# Daily variation of gene expression in diverse rat tissues

**DOI:** 10.1371/journal.pone.0197258

**Published:** 2018-05-10

**Authors:** Panteleimon D. Mavroudis, Debra C. DuBois, Richard R. Almon, William J. Jusko

**Affiliations:** 1 Department of Pharmaceutical Sciences, School of Pharmacy and Pharmaceutical Sciences, State University of New York at Buffalo, Buffalo, New York, United States of America; 2 Department of Biological Sciences, State University of New York at Buffalo, Buffalo, New York, United States of America; University of Texas Southwestern Medical Center, UNITED STATES

## Abstract

Circadian information is maintained in mammalian tissues by a cell-autonomous network of transcriptional feedback loops that have evolved to optimally regulate tissue-specific functions. An analysis of daily gene expression in different tissues, as well as an evaluation of inter-tissue circadian variability, is crucial for a systems-level understanding of this transcriptional circuitry. Affymetrix gene chip measurements of liver, muscle, adipose, and lung tissues were obtained from a rich time series light/dark experiment, involving 54 normal rats sacrificed at 18 time points within the 24-hr cycle. Our analysis revealed a high degree of circadian regulation with a variable distribution of phases among the four tissues. Interestingly, only a small number of common genes maintain circadian activity in all tissues, with many of them consisting of “core-clock” components with synchronous rhythms. Our results suggest that inter-tissue circadian variability is a critical component of homeostatic body function and is mediated by diverse signaling pathways that ultimately lead to highly tissue-specific transcription regulation.

## Introduction

Nearly all aspects of physiology and behavior such as sleep-wake cycles, body temperature and metabolism are influenced by cell-autonomous oscillators named circadian clocks [1]. In mammals, this circadian system retains a hierarchical coordination where at the top are molecular oscillators entrained to environmental cues (i.e. Zeitgebers) such as light and food, and subsequently orchestrate the rhythmicity of peripheral organs through hormonal signals, sympathetic innervation, or indirect cues (e.g. body temperature). Circadian rhythmicity of peripheral tissues persists for several days in the absence of environmental cues (i.e. light/dark cycles, eating/fasting activity) [2]. However, the temporal entrainment by the environment enables the physiological processes to be optimized with respect to daily changes and improves survival [3–5].

The molecular mechanism that drives circadian gene and protein expression is highly controlled by feedback interactions mediated by E-boxes, D-boxes, and Rev-Erba/ROR elements (RREs) [6–9]. In particular, the primary feedback loop consists of CLOCK and BMAL proteins that initiate transcription of target genes containing E-box sequences, including Period (*Per1*, *Per2*, *Per3*) and Cryptochrome (*Cry1* and *Cry2*). The PER and -CRY proteins heterodimerize, translocate into the nucleus, and inhibit their own transcription by preventing CLOCK:BMAL binding to E-box elements. In the ancillary feedback loop, REVERB and ROR proteins bind to RREs repressing and activating forward transcription respectively. Finally, DBP and NFIL3 bind to D-box elements ultimately forming a robust intracellular oscillator among core-clock genes that are present in almost all cells [10–13]. The circadian information generated by this small group of genes is expanded at the tissue level through regulation of clock-controlled genes (CCGs) expression, that eventually adjusts tissue specific rhythmic functions (e.g. metabolism, heart rate) [14, 15].

Numerous studies have underlined the importance of peripheral tissue clocks in several physiological processes such as glucose homeostasis, energy balance, xenobiotic/endobiotic detoxification, renal plasma flow, urine production, blood pressure and heart rate [16–25]. However, the main motives for peripheral circadian rhythmicity, as well as the extent of circadian regulation at the periphery, are not yet fully understood. One hypothesis is that peripheral organs maintain this rhythmicity in order to optimize the conservation of resources, while others propose that this rhythmicity may serve to optimize cellular physiology [26]. Interestingly, recent studies in mice show that different tissues maintain highly different rhythmic characteristics at least with respect to the phase of circadian expressed genes, with a very low overlap of common gene circadian expression in the different tissues [27–29]. These observations suggest a highly tissue-specific circadian regulation that probably serves different purposes for the different tissues. Furthermore, the relative contribution of cell-autonomous rhythms relative to regulation by systemic cues is still not clear [30]. One of the main current assumptions is that systemic cues entrain only the small group of core-clock genes (i.e. Per, Cry, Bmal1), and these subsequently regulate the distribution of clock-controlled gene phases through the tissue-specific interlocked clockwork circuitry [26]. Deciphering the mechanism that gives rise to circadian rhythmicity in peripheral tissues is of importance in order to understand systemic regulation of the body.

Liver, muscle, adipose tissue, and lung are organs playing a pivotal role in numerous body processes such as metabolism and transport of molecules, innate immune response, cell signaling, and others [31–34]. Due to their importance, many tissue functions are commonly targeted by drugs. Interestingly, the nature of the processes carried out by these tissues suggests that many of the expressed genes are under circadian control. In this regard, a circadian analysis of gene expression as well as a comparative investigation of common circadian gene characteristics among these tissues is a step towards a systematic understanding of inter-tissue dynamics resulting in homeostasis and well-being.

In this study, Affymetrix data from liver, muscle, adipose, and lung of Wistar rats were used to analyze circadian expression of genes in these tissues. By employing JTK_CYCLE non-parametric algorithm, genes that show circadian changes in expression over time were identified and compared among the tissues examined. Accordingly, inter-tissue rhythmic variability was evaluated. To assess rhythmicity of biological processes in the different tissues, genes that maintained robust oscillations were further categorized according to their biological function, and compared between tissues. This analysis provides a systematic evaluation of tissue-specific circadian characteristics, as well as a systems-level understanding of inter-tissue rhythmic variabilities that lead to homeostatic regulation.

## Materials and methods

### Animals

The research protocol adhered to the “Principles of Laboratory Animal Care” (NIH Pub. No. 85–23, revised 1985) and was approved by the State University of New York at Buffalo Institutional Animal Care and Use Committee. A detailed description of the animal experiments can be found in our previous published reports [31, 35]. In brief, our studies involved 54 normal male Wistar rats from Harlan Laboratories (Indianapolis, IN) that were allowed to acclimatize for 2 weeks in a constant 22°C environment equipped with a 12:12-hr light-dark cycle (full spectrum UV lighting) with free access to standard rat chow and drinking water. Animals were housed in single cages. Twenty-seven rats (Group I) were acclimatized for 2 weeks before study to a normal light/dark cycle, where lights went on at 8:00 AM and off at 8:00 PM. The onset of the light period was considered as time 0. The other 27 rats (Group II) were acclimatized for 2 weeks before study to a reversed light/dark cycle, where lights went on at 8:00 PM and off at 8:00 AM. Animals (8–9 weeks old) were anesthetized with a mixture of ketamine and xylazine (80 and 10 mg/kg) and killed by exsanguination through the abdominal aorta on three successive days at 0.25, 1, 2, 4, 6, 8, 10, 11, and 11.75 h after lights on for the light period time points, and at 12.25, 13, 14, 16, 18, 20, 22, 23, and 23.75 h after lights on for time points in the dark period. Time points were selected with extra weight given to light-dark transition periods, as it is likely that more dynamic changes would occur during such transitions. For animal manipulations in the dark period, lab personel utilized night vision goggles until surgical anesthesia was obtained in order to avoid animal exposure to light. Animals sacrificed at the same time on the three successive days were treated as triplicate measurements and further analysis was performed on this triplicate dataset. Livers, gastrocnemius muscles, abdominal fat pads, and lungs were excised and frozen in liquid nitrogen immediately after sacrifice and stored at -80°C until RNA preparation.

### Microarrays

Tissue samples from each animal were ground into a fine powder in a mortar cooled by liquid nitrogen. Total RNA was extracted in tri-reagent and further purified by passage through RNeasy mini-columns (Qiagen Inc, Carol Stream, IL) according to manufacturer’s directions for RNA clean-up. Final RNA preparations were suspended in RNase-free water and stored at -80 °C. The RNAs were quantified spectrophotometrically, and purity and integrity were assessed by formaldehyde-agarose gel electrophoresis. All samples used for arrays exhibited 260 nm to 280 nm absorbance ratios of ~ 2.0, and all showed intact ribosomal 28S and 18S RNA bands in an approximate ratio of 2:1, as visualized by ethidium bromide staining. Isolated RNA from each sample was used to prepare the target according to Affymetrix protocols. The biotinylated cRNAs were hybridized to 54 individual Affymetrix GeneChips Rat Genome 230A for liver and muscle and 230A_2 for adipose and lung (Affymetrix, Santa Clara, CA). The high reproducibility of in situ synthesis of oligonucleotide chips allows accurate comparison of signals generated by samples hybridized to separate arrays. These data were submitted to Gene Expression Omnibus (GEO) (GSE8988 for liver, GSE8989 for muscle, GSE20635 for adipose, and GSE25612 for lung).

### Data mining for circadian rhythmicity and phase calculation

Affymetrix Microarray Suite 5.0 (Affymetrix) was used for initial data acquisition and analysis. In order to enable inter-tissue analysis normalization was performed in two steps. Initially, the signal intensities were normalized for each chip with a distribution of all genes around the 50^th^ percentile for that chip. Next, using GeneSpring, the value of each probe set on each chip was normalized to the average of that probe set on all chips in that tissue set. As such, inter-tissue signal variabilities were taken into consideration and the expression pattern of all probe sets in all tissues oscillated approximately around 1. Microarray data for the individual tissues were published [31, 33–35]. Building on our previous efforts, in this report we provide a systematic analysis of all tissues jointly. In order to consider whether a ProbeSet maintains circadian rhythmicity, we used the JTK_CYCLE algorithm [36]. The algorithm evaluates whether data maintain a periodic pattern by using a non-parametric method based on the Jonckheere-Terpstra test for monotonic ordering, and Kendall’s τ test for association of measured quantities. Each algorithm evaluation is accompanied by a Bonferonni-adjusted p-value, and a Benjamini-Hochberg procedure q value that controls the false discovery rate (FDR) as well as evaluations of the amplitude (A) and phase (ϕ) of the oscillation. Similar to the work of [29], parameters of the JTK_CYCLE were set to fit the time series data to exactly 24 hr periodic waveforms, and a 5% FDR value was set for detection. The phase lag or phase difference Δϕ between the expression levels of the same gene in two tissues (i,j) was calculated as:

$${\Delta \phi }_{Ti/Tj}\;=\;|\phi_{Ti}-\phi_{Tj}|$$ (1)

where ϕ_*Ti*,*j*_ is the phase of the sinusoid resulting from fitting a certain gene in Tissue *i*,*j* and the vertical bar denotes the absolute value. Similarly, the maximum phase lag between the expression levels of the same genes in three (*i*,*j*,*k*) and four tissues (*i*,*j*,*k*,*l*) were:

$${max\Delta \phi }_{Ti/Tj/Tk}\;=\;\text{max}\;\left({\;\Delta \phi }_{\frac{Ti}{Tj}},{\;\Delta \phi }_{\frac{Ti}{Tk}},\;{\Delta \phi }_{\frac{Tj}{Tk}}\right),$$ (2)

$${max\Delta \phi }_{\frac{\frac{Ti}{Tj}}{Tk}/Τl}\;=\;max\;({\;\Delta \phi }_{\frac{Ti}{Tj}},\;{\;\Delta \phi }_{\frac{Ti}{Tk}}\;,{\Delta \phi }_{\frac{Ti}{Tl}},\;{\;\Delta \phi }_{\frac{Tj}{Tk}},\;{\;\Delta \phi }_{\frac{Tj}{Tl}},\;{\;\Delta \phi }_{\frac{Tk}{Tl}})$$ (3)

Throughout this work, 5the phase difference *Δ*ϕ is expressed in hours and has values from 0 h when two rhythms have the same phase and 12 h when rhythms are antiphasic in the tissues examined. For the case of individual phases, radian units are preferably used.

The percent amplitude difference between the expression levels of the same gene in two tissues was calculated as:

$${\Delta A}_{Ti/Tj}\;=\;\frac{A_{Tmax}-A_{Tmin}}{A_{Tmax}}*100$$ (4)

where *A*_*Tmax*_ is the larger JTK_CYCLE amplitude between tissues T_i_ and T_j_ and *A*_*Tmin*_ the lower. The maximum percent amplitude difference between the expression levels of the same genes in three and four tissues were:

$${max\;\Delta A}_{Ti/Tj/Tk}\;=\;\text{max}\;\left({\;\Delta A}_{\frac{Ti}{Tj}},{\;\Delta A}_{\frac{Ti}{Tk}},\;{\Delta A}_{\frac{Tj}{Tk}}\right),$$ (5)

$${max\Delta A}_{\frac{\frac{Ti}{Tj}}{Tk}/Τl}\;=\;max\;({\;\Delta A}_{\frac{Ti}{Tj}},\;{\;\Delta A}_{\frac{Ti}{Tk}}\;,{\Delta A}_{\frac{Ti}{Tl}},\;{\;\Delta A}_{\frac{Tj}{Tk}},\;{\;\Delta A}_{\frac{Tj}{Tl}},\;{\;\Delta A}_{\frac{Tk}{Tl}})$$ (6)

Percent amplitude values range from 0% when two genes retain the same amplitude and 100% when the lower amplitude is infinitesimally smaller than the greater one. For evaluating phase correlation, the circular correlation measure (r) was calculated as:

$$r\;=\;\frac{\sum_{k\;=\;1}^{n}\text{sin}\;\left(a_{1k}-T_{1,1}\right)sin(a_{2k}-T_{2,1})}{\sqrt{\sum_{k\;=\;1}^{n}{\text{sin}}^{2}\;\left(a_{1k}-T_{1,1}\right)\sum_{k\;=\;1}^{n}{\text{sin}}^{2}\;\left(a_{2k}-T_{2,1}\right)}}$$ (7)

where a_1k_ is the phase angle of gene k in tissue 1 and a_2k_ the phase angle of gene k in tissue 2. Similarly, T_1,1_ and T_2,2_ are the mean direction of the first and second circular variables [37]. For circular phase correlation calculations, the *circ*.*cor* function from *CircStats* package in R was used.

### Functional clustering

Official symbols corresponding to each of the genes showing rhythmic oscillations were analyzed using various online tools and databases, including Pubmed, Rat Nomenclature Guidelines (RGD), and GeneCards. From this information, an extensive literature search was performed as well as use of DAVID gene functional classification tool [38, 39] and PANTHER databases [40] to identify the functions and other relevant information for each gene. Detailed results from functional clustering are shown in supporting information Tables L-O in S1 Appendix.

### Comparison of circadian gene expression between rat and mouse

Circadian analysis results for rats were compared with a recent analyses in mice [29]. The GEO databases GSE8988 (liver), GSE8989 (muscle), GSE20635 (adipose), and GSE25612 (lung) were used for rat, and the GEO database GSE54652 (all-tissues) for mouse. For the case of mouse, the sub-series GSE54650 were used that contain gene expression data assayed by microarrays. For rats, the microarray chips used were Affymetrix GeneChips Rat Genome 230A for liver and muscle (15,967 probe sets) and 230A_2 for adipose and lung (>31,000 probe sets), whereas for the case of mouse the Affymetrix MoGene 1.0 ST arrays were used (>35,000 probe sets). Microarray data from both species were analyzed for circadian gene expression using JTK_CYCLE algorithm [36] with an FDR threshold of 5%.

## Results

**Fig 1:**
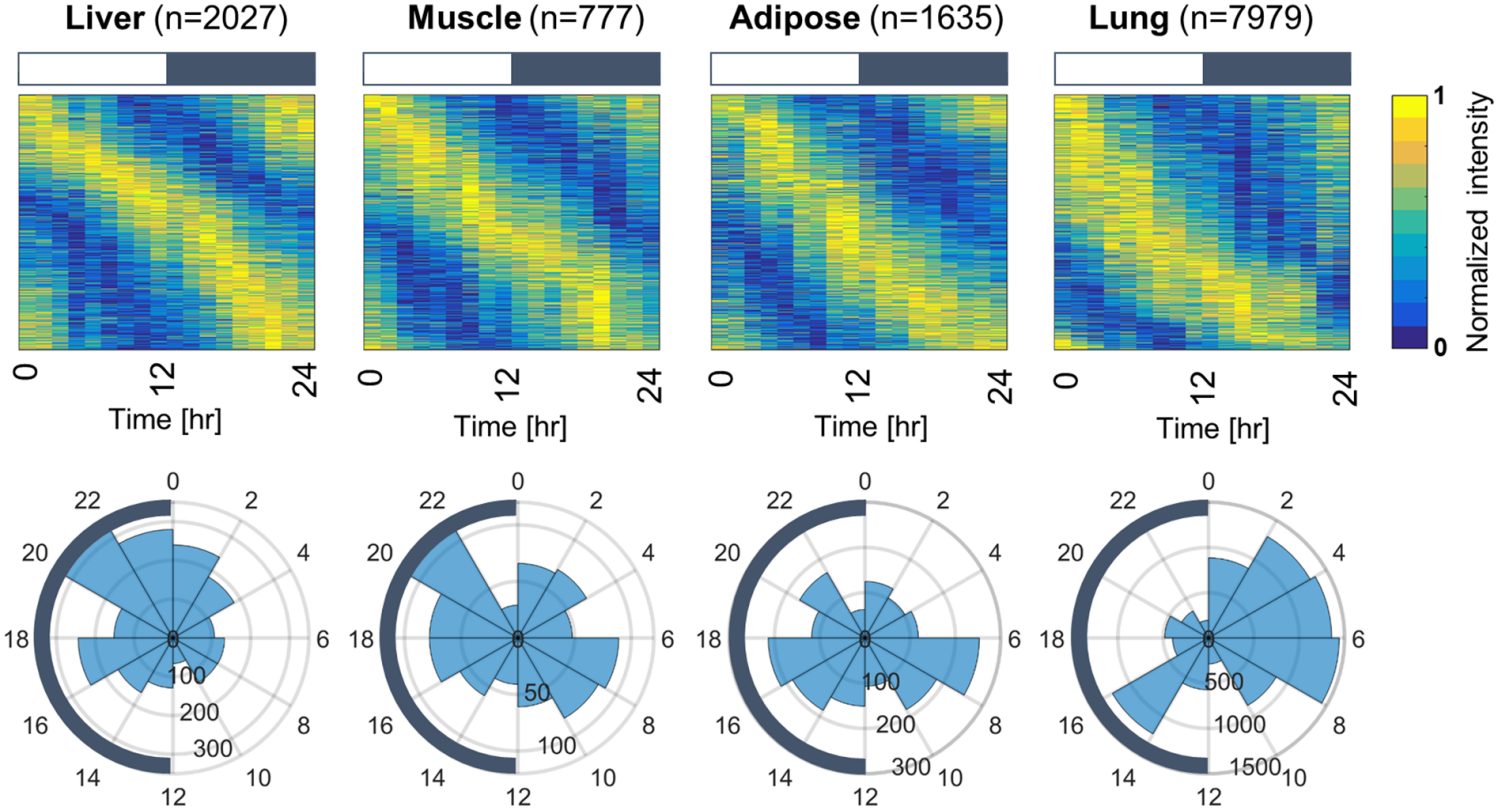
Temporal profiles of genes maintaining circadian rhythmicity in liver, muscle, adipose, and lung. Upper panel: Heatmaps showing mean expression data from 3 animals sacrificed at the same time point during three consecutive days. Rows represent the different genes, and columns the mean expression values at the different times of the day. From blue to yellow, the expression intensity is increasing. Ordering of genes in the different rows is based on their phase. Heatmap titles indicate the tissue, and the respective bar plots at the top of each subplot represent the 12 h light (white) 12 h dark (grey) periods. The n is for the number of genes found to retain circadian rhythmicity in each tissue Lower panel: Respective phase histograms for the tissues shown in the upper panel. Circular coordinates indicate the time of day and numbers on the nested circles the number of genes. Dark semicircles at the perimeter of the circles indicate the dark phase.

After normalization and mining, genes that maintain robust rhythmicities were assessed by using the JTK_CYCLE algorithm. Fig 1 shows expression profiles of the genes found to maintain circadian activity in the tissues examined. Heatmap subplots in the upper panel represent mean expression intensities (mean of three animals) of the different genes during the 24 h period, with the highest value normalized to 1 and lowest to 0 for visualization purposes. For the case of liver, the total genes that maintain circadian activity were 2027 with most maintaining highest expression intensities between 12 and 24 hours (in the dark period). Muscle with 777 and adipose with 1635 circadian genes present a more uniform expression distribution. In lung, the total number of circadian genes were 7979 and the majority maintain highest expression between 0 and 12 hours in the light/inactive phase. The lower panel presents phase histograms which further underline the distribution of phases for each tissue. In liver, most of the genes peak between 22 and 2 hours at the transition of dark/light phase while smaller clusters of peak times occur around the whole 24-hour day. Genes in muscle mainly peak at two periods in the 24 hr cycle, one at the middle of the dark period (between 16 and 22 hours) and a second between 6 and 10 hours at the light phase. Similarly, adipose circadian genes peak a few hours earlier in the dark period (between 12 and 18 hours) and at the light period between 6 and 10 hours. In contrast, most of genes oscillating in lung peak at the early light period between 0 and 8 hours.

**Fig 2:**
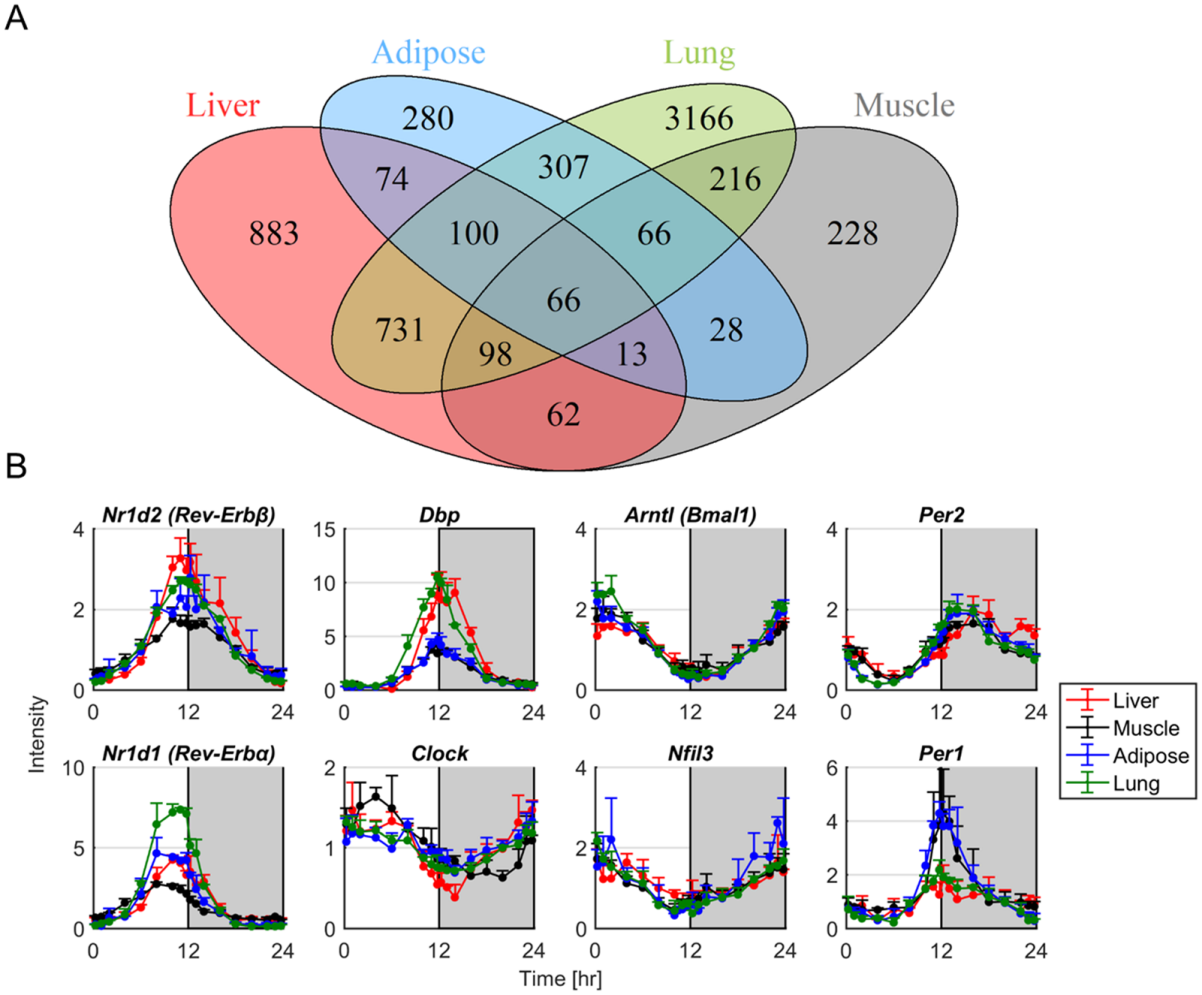
Common genes that maintain circadian rhythmicity in the four tissues. A: Venn diagram of the four tissues. B: Temporal profiles of core-clock genes that are oscillating in all four tissues. Dots represent the average expression values from 3 animals sacrificed at three consecutive days and error bars the standard deviation of these three replicates.

After assessing circadian rhythmicity, the resulting genes were compared among the different tissues examined. The 4 set Venn diagram of Fig 2A illustrates the number of common genes that are oscillating in all combinations of the four tissues. In total, 66 genes are commonly oscillating in all four tissues. Many of these genes belong to the core-clock gene family and maintain highly synchronous rhythmicity across tissues. Their profiles are shown in Fig 2B. The *Per2*, *Rev-Erbα*, *Rev-Erbβ*, and *Dbp* genes peak at late light/early dark period where the rat activity phase begins and maintain nearly antiphasic expression with *Nfil3* and *Bmal1* genes that retain highest expression at the early light period.

**Fig 3:**
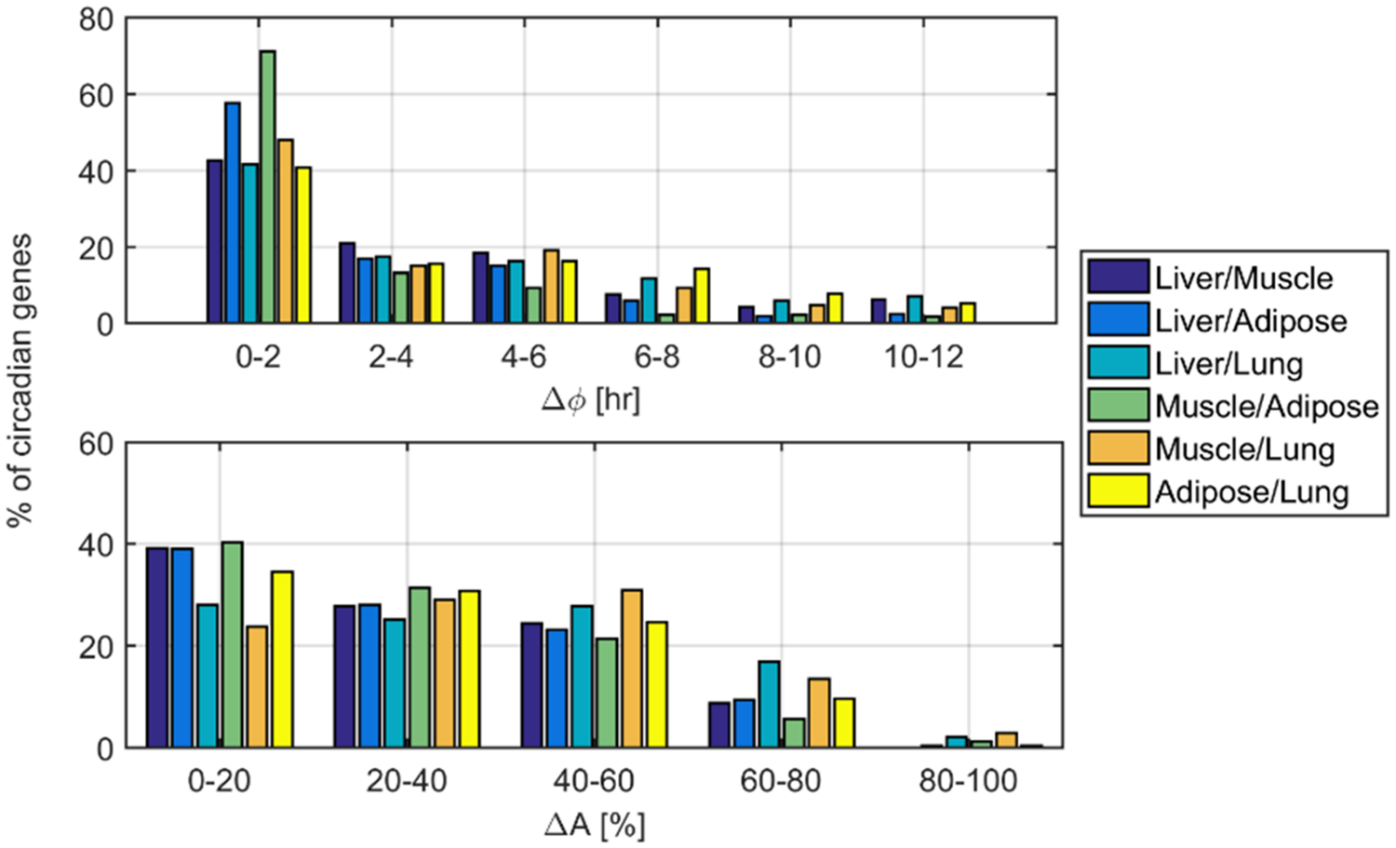
Phase and amplitude variabilities in genes oscillating commonly in all 2-tissue combinations. Upper panel: Histogram of phase lags of common genes in two tissue combinations. Different colors represent the various tissue combinations. Phase lags are separated into 6 groups on the x-axis, and represent genes that have a phase difference between 0 to 2, 2 to 4, 4 to 6, 6 to 8, 8 to 10, and 10 to 12 hours. The y-axis depicts the percentage of circadian genes retaining a certain phase lag for combinations of certain tissues where Δϕ represent the phase lag (phase difference) in hours. Lower panel: Histogram of % amplitude difference of common genes in two tissue combinations. Different colors represent the various tissue combinations. The % amplitude differences are separated in 5 groups on the x-axis, and represent genes that have a % amplitude difference 0 to 20, 20 to 40, 40 to 60, 60 to 80, and 80 to 100. The y-axis depicts the percentage of circadian genes retaining a certain amplitude difference for combinations of certain tissues where ΔA represent the amplitude difference in %.

Next, the variability of phases and amplitudes among genes commonly oscillating in two or more tissues was explored. In the following, detailed results for common genes in two tissues are described while results for 3- and 4- tissues are shown in the supporting information and only summarized here. Fig 3 subplots show phase lags (Δϕ) and amplitude differences (ΔA) of common genes in 2 tissues, represented as bar plots for the different combinations of tissues. For all 2-tissue combinations, the majority of the common genes maintain high synchronicity with a phase lag between 0 to 2 hours (Fig 3 upper subplot). Similarly, in 4 out of 6 combinations of tissues, most of the common genes maintain an amplitude difference of 0–20%. However, the amplitude variability presents an overall wider distribution compared to phase variability since percent amplitude differences among common genes in different tissues more uniformly occupy the range of values (Fig 3 lower subplot). Detailed phase and amplitude variabilities among common genes in 2 tissues are presented in supporting information Tables A-F in S1 Appendix. For 3- and 4-tissues detailed results are shown in Tables G-K in S1 Appendix.

**Fig 4:**
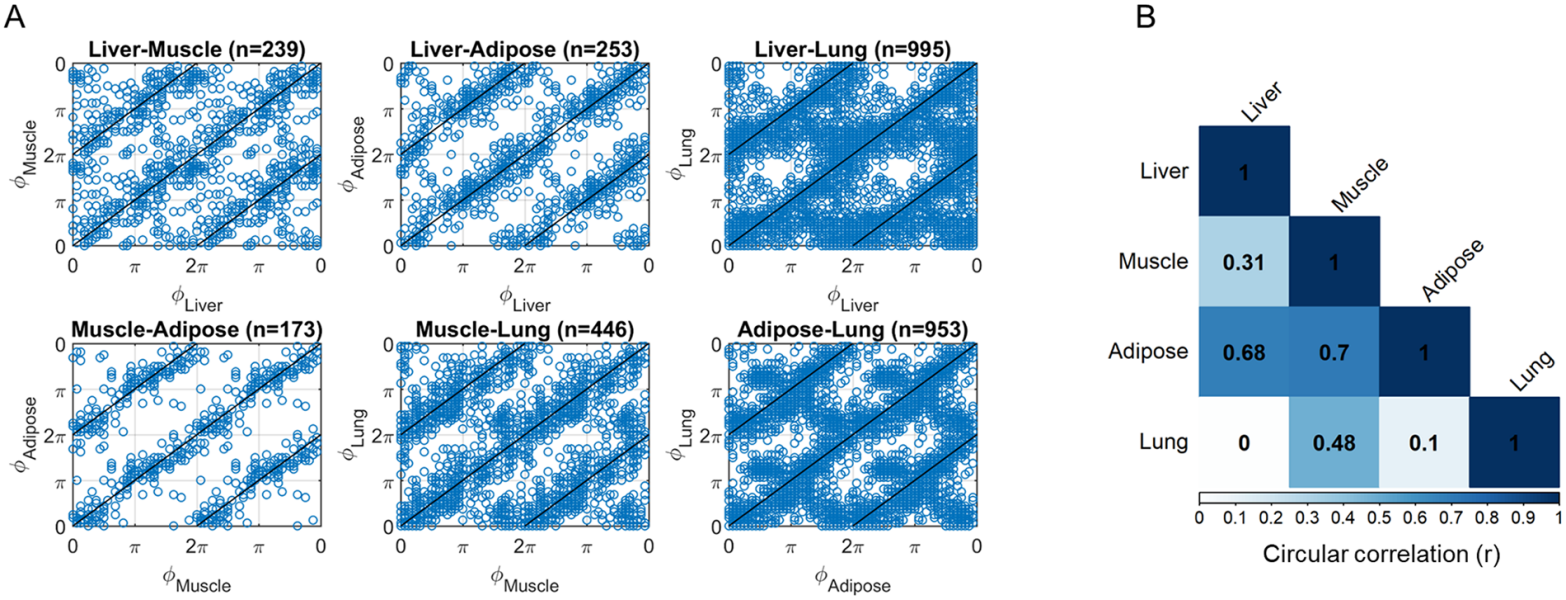
Correlation of phases (ϕ) between circadian genes oscillating in combination of 2 tissues. A: Scatterplots of phases (ϕ) in combinations of two tissues compared with the identity line. Phases for 4 periods were concantenated for visualization purposes B: Caclulated circular correlation coefficients (r) for the phases of circadian genes in the respective combinations of tissues.

To evaluate the combination of tissues that retain most genes that oscillate in synchrony, a circular version of Pearson’s correlation coefficient [37] was calculcated between the phases (ϕ) of common genes that oscillate in all combination of two tissues (Fig 4A). As shown in Fig 4B the circadian genes in muscle and adipose retain the most correlated phases (ϕ), followed closely by liver and adipose. Circadian genes in all other combinations retain correlation coefficients lower than 0.5. Lowest correlation was shown between the phases of circadian genes in liver and lung.

**Fig 5:**
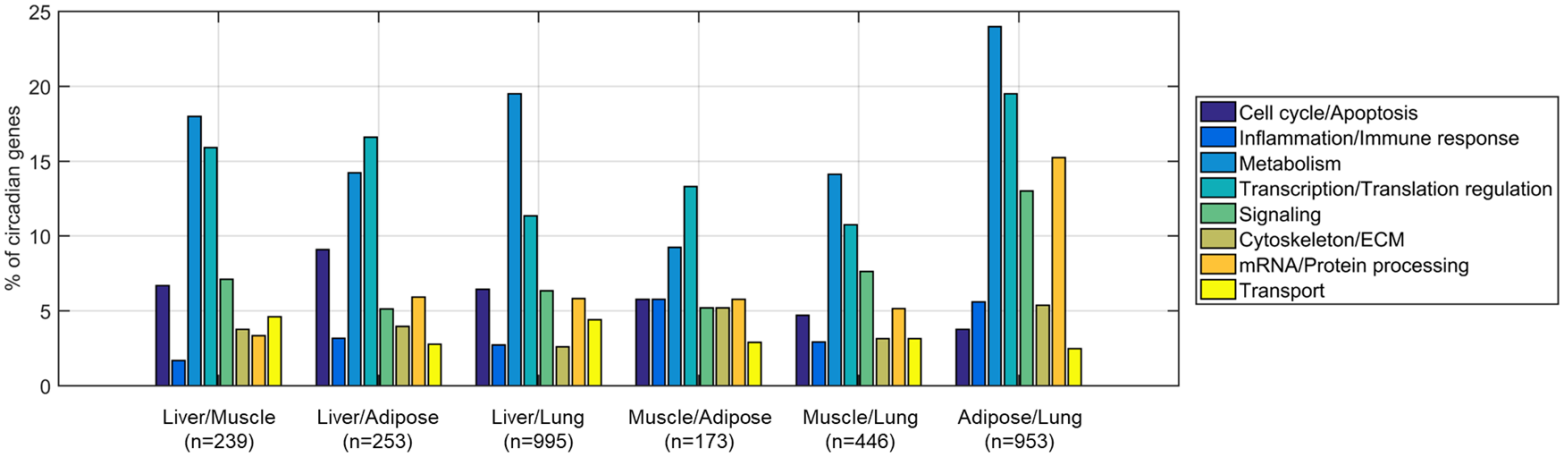
Bar plots showing the different functional groups of the genes for the various combinations of 2-tissues. Different colors indicate the different functional groups. The x-axis represents the different combination of tissues and the y-axis the percentage of circadian genes that belong to a certain functional group. The n is the number of genes found to maintain circadian oscillations in the respective combinations of tissues.

In addition, the functional groups to which the common genes belong were assessed. Overall, in line with previous efforts, 8 functional groups were identified: Cell Cycle/Apoptosis, Inflammation/Immune response, Metabolism, Transcription/Translation Regulation, Signaling, Cytoskeleton/Extracellular matrix (ECM), mRNA/Protein Processing, and Transport. Fig 5 shows the percent occupation of each functional group for the genes that are commonly expressed in 2 tissues. In all tissue combinations, the groups that have the highest occupancy are the ones that involve genes responsible for Transcription/Translation regulation and Metabolism. The genes that are commonly oscillating in liver/lung are represented largely in Metabolic Processes compared to Transcription/Translation.

**Fig 6:**
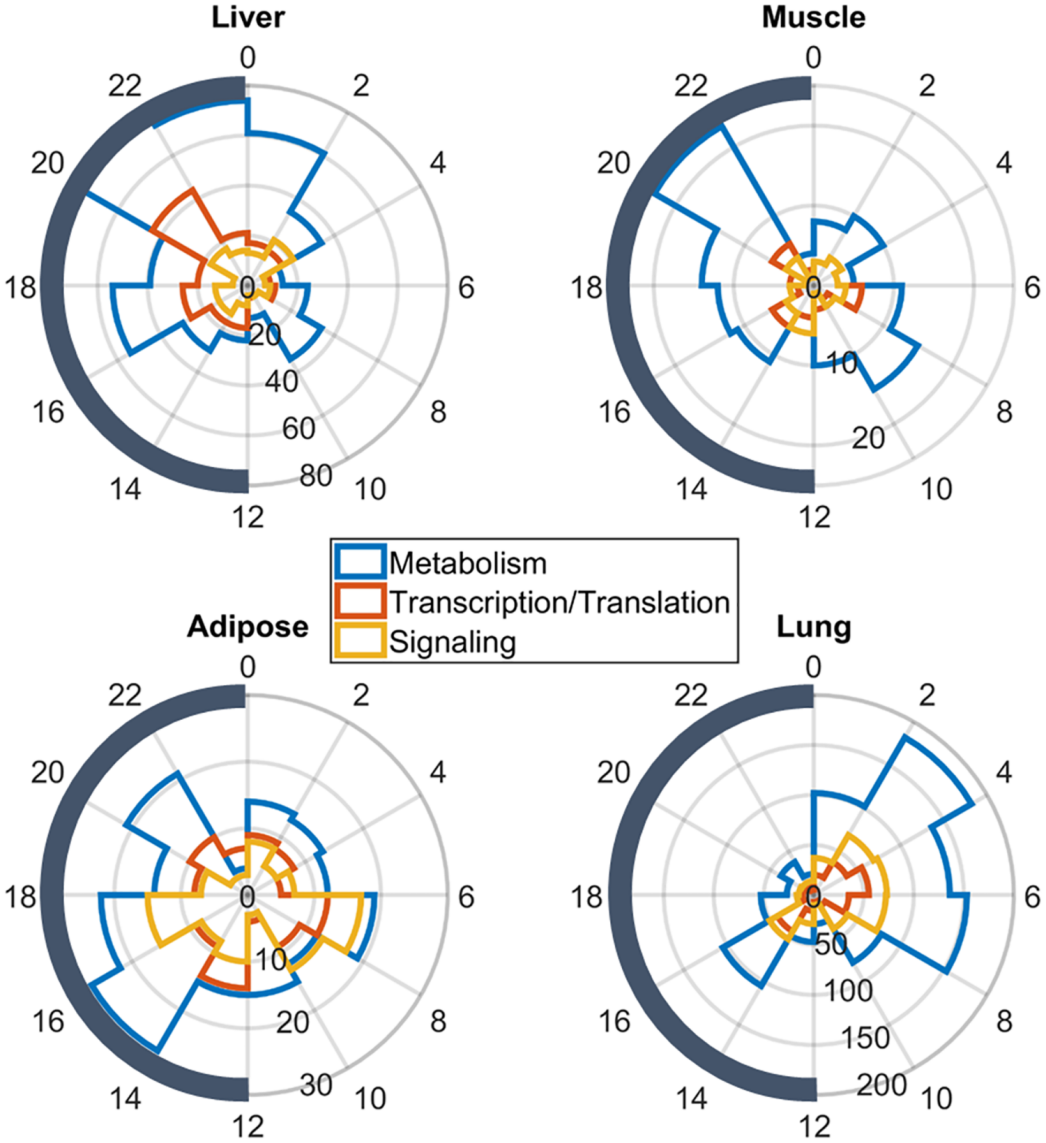
Phase histograms for the circadian genes in the individual tissues, relative to their three main biological functions. Circular coordinates indicate the time of day and numbers on the nested circles the numbers of genes. Different colors depict different functional groups.

The phases for the various specific biological functions were further evaluated across the tissues. Fig 6 shows the tissue-specific histograms of the three most highly populated functional groups on average (Transcription/Translation, Metabolic Processes, and Signaling). In liver most of the genes functionally categorized as participating in Metabolic Processes peaked at late dark early light period. In muscle there are two peaks, one in the late dark phase in addition to a peak in the late light phase. Similarly, adipose maintains a main peak of metabolic processes at early dark period as well as considerable amount of metabolic genes that peak at the light phase. However, in lung most genes participating in Metabolic processes peak near the middle of the light period. Genes that are categorized as participating in Transcription/Translation as well as signaling processes follow similar patterns. In liver, the vast majority of genes peak at the dark/light transition whether in muscle and adipose the distribution of peaks is more dispersed with significant amount of genes peaking in dark as well as light periods. For the case of lung, Transcription/Translation and signaling-related genes peak mainly in the light period. Genes that oscillate in the different tissues as well as their biological function are shown in Tables L-O in S1 Appendix.

**Fig 7:**
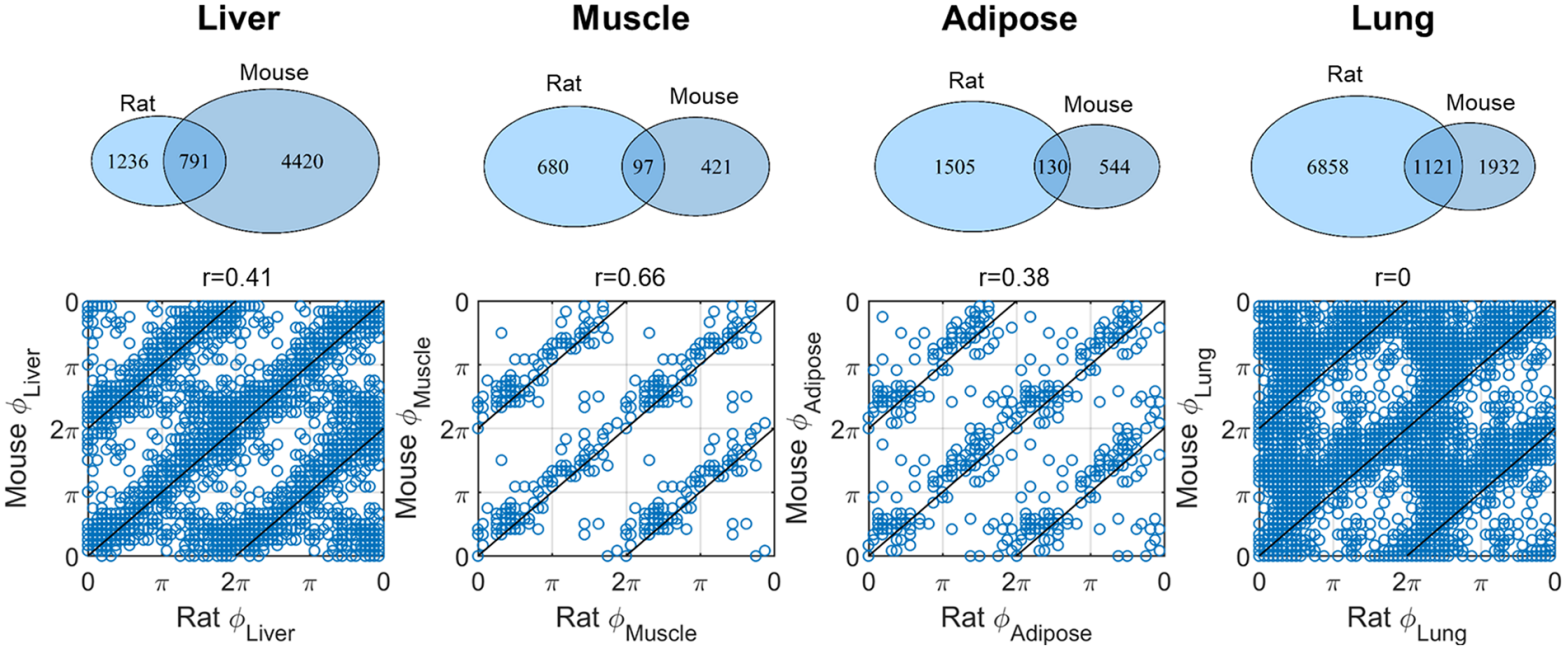
Conserved genes in mouse and rat for liver, muscle, adipose and lung. Upper panel: Venn diagrams showing number of genes in each tissue for mouse and rat as well as the number of overlapping genes. Lower panel: Scatterplots of phases (ϕ) in rat and mouse for the different tissues compared with the identity line. Phases for 4 periods were concantenated for visualization purposes. r values at the title of the graphs indicate the circular correlation coefficient.

Lastly, the extent of circadian gene conservation between mouse and rat was investigated. Interestingly, from the total 2027 genes found to maintain circadian rhythmicity in rats, 791 have been also found in mice showing a 39% overlap of the circadian genome of the two species in liver (Fig 7 upper panel, Liver). For muscle, from the total 777 circadian genes in rats, 97 were conserved between mouse and rat showing a 13% overlap (Fig 7 upper panel, Muscle). For adipose the observed overlap of circadian genome was 8% and for lung 14% (Fig 7 upper panel, Adipose and Lung). In Fig 7 lower panel, the phases of the conserved genes were plotted for the different tissues. The title of each subplot indicates the value of the circular correlation coefficient (same as Fig 4). The rat tissue that retains the most genes with phases correlated with mouse is muscle, followed by liver and adipose. Phases of genes in rat lung are not correlated with the same genes in mouse. Detailed gene names together with the calculated phases are shown in Tables P-S in S1 Appendix.

## Discussion

Homeostasis reflects an orchestrated integration of organs and tissues that functions to sustain health. The maintenance of biological stability is therefore highly dependent on the efficient coupling of interconnected tissues that often maintain autonomous rhythmicities [41, 42]. In order to elucidate the dynamics underlying homeostasis at the systemic level, we present a genome-wide analysis of circadian rhythms in mRNA expression in 4 tissues of the adult male rat.

This study was carried out in intact Wistar rats acclimated to a tightly controlled 12 hour light/12 hour dark cycle, but with the animals otherwise undisturbed. Microarray data from rat liver, muscle, adipose and lung were used for forward data analysis. A variety of algorithms have been proposed previously for the identification of rhythmic patterns in microarray data both in time as well as frequency domain. The most widely applied, COSOPT [43] method in time domain measures the goodness of fit between data and cosine curves of various phases and periods whereas Fisher’s *G*-test computes the data periodogram in the frequency domain and calculates the significance of the dominant frequency [44]. Recently, Hughes et al. [36] introduced a novel algorithm that applies Jonckheere-Terpstra (JT) test to the null distribution of Kendall’s tau correlations (JTK_CYCLE). In [45] the various methods of rhythm identification were compared and the strengths and weaknesses of the different methods were evaluated ultimately providing a recommendation graph based on the different experimental settings. One of the salient features of this study is the richness of our data set that incorporates denser sampling points at the light/dark and dark/light transition periods (0.25, 1, 2, 4, 6, 8, 10, 11, and 11.75 h after lights on for the light period time points, and at 12.25, 13, 14, 16, 18, 20, 22, 23, and 23.75 h after lights on for time points in the dark period). This extensive sampling of this study design encourages the use of the JTK_CYCLE algorithm for detecting circadian rhythmicity and calculating rhythm’s phase and amplitude. Similar to the work of [29] JTK_CYCLE was set up to detect exact 24 hr oscillations in transcript abundance. More complicated rhythms of different frequencies were out of the scope of the present analysis.

As shown in Fig 1, the majority of circadian genes in liver, muscle, and adipose maintain highest expression in the dark period, which is also the active period in these nocturnal rodents. Specifically, phase histograms show that although these tissues contain genes that are peaking throughout the 24-hour period, the most populated clusters reside in the dark phase. Especially in liver, the vast majority of the genes peak at late dark phase from (18hr to 0hr). For muscle and adipose there are two transcriptional surges: one during the light/inactive period and one during the dark/active period. However, in lung the phase distribution differs substantially from the other tissues since its circadian genes maintain highest expression in the light/inactive phase between 0 and 12 hr. Interestingly, similar results have been observed recently in mice where among 12 organs examined, rhythmicity in lung and heart differed significantly [29]. Overall, our results are in accordance with experiments conducted mainly in mice [28, 29, 46, 47] indicating the presence of “rush hours” preceding dawn and dusk when transcriptional activity is increased as the body anticipates the forthcoming light/dark phase change.

A limitation of our analysis is that, owing to development times, different gene chips were used for data acquisition between liver/muscle (Affymetrix GeneChips Rat Genome 230A), and adipose/lung (Affymetrix GeneChips Rat Genome 230_2). The Rat Genome 230A that was used for liver and muscle contains 15967 probe sets whereas the Rat Genome 230_2 that was used for lung and adipose contains >31000 probe sets. Therefore, absolute numbers of genes that maintain circadian activity is not directly comparable among all tissues (Fig 1, n values). However, from the total 15967 probe sets in liver and muscle almost 14% maintain circadian activity in liver and 5% in muscle, whereas from 31099 probe sets of adipose and lung 30% maintain circadian activity in lung, and 6% in adipose. Circadian activity in lung is therefore proportionally higher among all tissues, followed by liver, adipose and muscle. Previous work reports 8–16% circadian genes in liver, 12% in lung, and 4% in muscle and adipose tissue [28, 29]. In all tissues apart from lung the percentages of circadian genes are comparable to previous works. A significantly larger circadian regulation was found in lung. The diverse percentages observed may be due to the different experimental procedures used for mRNA quantification or different array systems (e.g. Rat Affymetrix GeneChips Rat Genome 230 vs Affymetrix MoGene 1.0 ST arrays). Furthermore, the design of our data sampling with extensive measurements at inflection points facilitates the mining of circadian patterns.

Our analysis reveals that the circadian regulation for the majority of genes is tissue specific and there are only a small number of common genes oscillating between any tissue combination (Fig 2, Tables A-F in S1 Appendix). Fig 2 compares only the probe sets that are present in all tissues examined. As illustrated by the Venn diagram (Fig 2A) there are (307+66+66+100) 539 genes that are commonly oscillating between adipose and lung, (74+100+66+13) 253 genes between liver and adipose, (731+98+66+100) 995 between liver and lung, (98+66+13+62) 239 between liver and muscle, (66+66+13+28) 173 between muscle and adipose, and (216+66+66+98) 446 between muscle and lung. Consequently, the two tissues that share the most commonly oscillating genes are liver and lung whereas adipose and muscle retain fewer commonly oscillating genes. The greater resemblance between liver and lung further underlines that both tissues take part in associated body functions such as metabolism (Fig 5).

Among all tissues examined, there are 66 genes that are commonly expressed with circadian rhythmicity. There are 8 out of 66 genes that are directly assigned to regulation of the tissue-specific clock. In particular, *Per1-2*, *Rev-Erbα*, and *Rev-Erbβ* take part in E-box-mediated transcription by repressing forward gene expressions (E-box transcriptional repressors), whereas *Clock* and *Bmal1* act as E-box transcriptional activator. Similarly, *Dbp* and *Nfil3* regulate *Dbox*-mediated transcription as activator and repressor respectively [14]. This interconnected network of molecular interactions leads to self-sustained oscillations in these tissues. Our analysis confirms the already established antiphasic relation between Ebox-mediated genes such as *Per1-2*, *Rev-Erbα*, *Rev-Erbβ* and *Dbp* that peak at the light/dark transition and RRE-mediated genes such as *Clock*, *Bmal1*, *Nfil3* that peak at the dark/light transition 12 hours later [48]. Apart from core-clock genes, our work reveals a number of additional genes that are commonly oscillating in all four tissues examined. Their biological functions are spread out to all eight functional groups investigated in this work. Detailed functions and names of the totality of these genes is shown in the Tables L-O in S1 Appendix.

The majority of the genes that are commonly oscillating in 2 or more tissues maintain a relative synchronicity. Fig 3 shows the phase lags (Δϕ), and amplitude differences (ΔA) of common genes in all combinations of two tissues. The majority of the common genes for any combination of two tissues have a phase lag between 0 and 2 hours. Despite this exceptional orchestration, there are a substantial number of genes with phase differences that are 2–4 hours apart (~20%) and can reach up to 12 hours. Similarly, the majority of genes in five out of six combination of 2-tissues retain amplitudes that deviate between 0–20%. Exception is genes commonly oscillating in muscle/lung, the majority of which retain an amplitude difference between 20–40%. Amplitude differences and phase lags are not correlated in common genes within two tissues. This further means that higher synchronicity of genes among tissues is not necessarily accompanied by similar amplitudes of those genes.

These results indicate that despite the fact that the different tissues receive the same systemic signals and therefore are entrained to the same humoral, systemic, and indirect cues of the body, there are a considerable number of genes that maintain variable phases among different tissues. Results shown in Fig 4 further supports this result by showing that genes in muscle/adipose and liver/adipose retain highly correlated phases (Fig 4B). The adipose-muscle axis and the importance of fat use as fuel for muscle contraction has been long appreciated. It is currently known that muscle contraction is in synchorny with increased expression of non-esterified fatty acids (NEFA) from adipose tissue that for the case of light exercise make up the oxidative fuel used by muscle [49]. Furthermore, chemokines such as MCP1, chemerin, and IL-6 were shown to be involved in a paracrine/endocrine cross-talk between muscle and adipose so to optimize inflammatory response [50]. The liver is the major metabolic organ responsible for energy utilization. The importance of liver-adipose tissue cross-talk was recently underlined through the fibroblast growth factor 21 (FGF21)-adiponectin axis that functions as a key mediator of energy consumption homeostasis and its disruption is an important contributor of cardio-metabolic syndrome [51, 52]. Our analysis points also towards a tight rhythmic relationship of liver and adipose tissue exemplified by the phase coherence of the circadian genes commonly oscillating in these two tissues. For the rest of the tissue combinations the circular phase correlations were lower, which further indicates that there is considerable inter-tissue variability with respect to “processing” systemic signals to the relative tissues.

The majority of common genes among 2-, 3-, and 4- tissues are participating mainly in processes related to Metabolism and Transcription/Translation regulation (Fig 5 for 2 tissues, and S3 and S4 Figs for 3 and 4 tissues). For the four out of six tissue combinations, Metabolism is the most populated functional group across tissues closely followed by Transcription/Translation. However, when comparing liver/lung, number of genes participating in Metabolic processes are significantly higher than genes in Transcription/Translation regulation processes. Interestingly, it is known that along with liver, the lung plays an important role in the metabolism of drugs administered either by inhalation or through systemic circulation, affecting their pharmacokinetics [53]. Furthermore, studies have shown that the pharmacokinetics of some drugs used for treating diseases including asthma, lung cancer and others, are time of day dependent, which could partially be linked to the oscillations of genes involved in Metabolic Processes [54].

Overall, the phases of different biological processes in the various tissues examined follow the rhythms observed when the whole transcriptome was analyzed (Fig 1). In liver, the three main biological functions namely Transcription/Translation, Metabolism, and Signaling maintain major peak times during the transition of active/dark phase and inactive/light phase (Fig 6). For the case of genes that participate in Metabolic Processes a second smaller peak is observed at the late light phase. Liver is the main site of metabolism of nutrients, endobiotics, and xenobiotics. Since rats are nocturnal animals and their feeding occurs in the dark/active phase, our study indicates that metabolic related genes are phased mainly to the appropriate time of their utilization but also to the light period where rodents are resting. Overall, biological processes in liver peak at the late dark early light phase. For the case of adipose and muscle, the bimodal phase distribution of genes participating in Metabolic, Transcription/Translation and Signaling processes is more characteristic. For the case of Metabolism and Transcription processes in muscle, they peak at late light and late dark phases whereas signaling processes maintain a peak at early light and early dark phases. In adipose, the Metabolic and Transcription/Translation processes peak at the late light phase nearly at the same time as muscle and atthe dark phase some hours earlier than that of muscle. Adipose signaling processes peak at the transition of light/dark phase. Finally, biological processes in lungs peak consistently at the early light phase. This suggests that Metabolism, Repair and Turnover processes mainly occur during the period when the organism is inactive and, therefore, has lower pulmonary demands, as the organism’s requirement for oxygen is minimal. For the case of liver, muscle, and adipose our study indicates two transcription surges, one in light and one in the dark period when the organism is getting prepared for the impending phase change.

Lastly, the extent of rhythmic genome conservation between mouse and rat was investigated. Mouse data were found in [29] for the tissues of interest. Our analysis indicated an extensive conservation of circadian genes especially for the case of genes maintaining circadian rhythmicity in rat liver with 39% of them present also in mouse liver. The phases of these overlapped genes between mouse and rat were moderately correlated with an r = 0.41. The next tissue with the highest percentage of conserved genes was lung with 14%. Interestingly, the phases of these genes did not correlate between the two species. Among all tissues explored, lung had the lowest circular correlation value (r = 0) and the highest correlation was in muscle (r = 0.66). Lastly, 8% of adipose circadian genes showed moderate species phase correlation. Due to the different microarray chips used for rat and mouse genome analysis and their different sensitivities (Materials and Methods-Comparison of circadian gene expression between rat and mouse), a direct comparison of the number of genes oscillating in the two species may be misleading.

In summary, our study presents a global analysis of the circadian pattern of transcription in four major tissues of rat. An extensive circadian regulation occurs in all tissues examined. In accordance with results in mice, our analysis reveals a highly tissue specific regulation of circadian genes with only a small group of genes oscillating in all tissues. Most of the genes oscillating in liver are peaking during the dark/active and light/inactive phase transition whereas in muscle and adipose genes exhibit more uniformly distributed peak times forming two main clusters during the light and dark phase likely anticipating the impending phases of the day. In lung, circadian genes peak mainly in the inactive periods of the rats. Intriguingly, genes that are commonly oscillating in 2 or more tissues present a high synchronicity with most of the genes retaining phase lags between 0 to 2 hours. Genes in liver and adipose maintain the highest correlated phases. Functional categorization of genes that are commonly oscillating in the tissues further indicates that most of these genes take part in Metabolic and Transcription/Translation processes. Concerning the total genome, most of the genes maintaining circadian rhythmicity participate in Transcription/Translation, Metabolic, and Signaling processes. Despite the variable rhythmicities of the different genes in the various tissues examined, they generally maintain two transcription peaks, one during the dark/active and one during the light/inactive phase of day that further indicate the anticipatory processes of the body to encounter the impending phase of the day.

## Supporting information

S1 Appendix Tables of genes maintaining circadian expression in individual and combination of the tissues examined, and comparison with circadian genes in mice. (DOCX)

S1 Fig Phase and amplitude variabilities in genes oscillating commonly in all 3 tissues combinations. A: Histogram of phase lags of common genes in 3 tissues combinations. Different colors represent the various tissue combinations. Phase lags are separated to 6 groups on the x-axis, and represent genes that have a phase difference between 0 to 2, 2 to 4, 4 to 6, 6 to 8, 8 to 10, and 10 to 12 hours. The y-axis shows the percentage of circadian genes retaining a certain phase lag for combinations of certain tissues. B: Histogram of % amplitude difference of common genes in 3 tissues combinations. Different colors reflect the various tissue combinations. The % amplitude differences are separated in 5 groups on the x-axis, and represent genes that have a % amplitude differences of 0 to 20, 20 to 40, 40 to 60, 60 to 80, 80 to 100. The y-axis depicts the percentage of circadian genes retaining a certain amplitude difference for combinations of certain tissues. C: Scatterplot of phase lag (x-axis) versus % amplitude difference (y-axis). Different colors show different combination of 3 tissues. Lower panel boxplots indicate distributions of phase lags in the different combinations of tissues. Upper panel boxplots indicate distributions of % amplitude difference in the different combination of tissues.
(TIF)

S2 Fig Phase and amplitude variabilities in genes oscillating commonly in all 4 tissues combinations. A: Histogram of phase lags of common genes in 4 tissues. Phase lags are separated to 6 groups on the x-axis, and represent genes that have a phase difference between 0 to 2, 2 to 4, 4 to 6, 6 to 8, 8 to 10, and 10 to 12 hours. The y-axis represents the percentage of circadian genes retaining a certain phase lag. B: Histogram of % amplitude difference of common genes in 4 tissues. % amplitude differences are separated in 5 groups on the x-axis, and represent genes that have a % amplitude different 0 to 20, 20 to 40, 40 to 60, 60 to 80, 80 to 100. The y-axis indicates the percentage of circadian genes retaining a certain amplitude difference. C: Scatterplot of phase lag (x-axis) versus % amplitude difference (y-axis). Lower panel boxplot shows the distribution of phase lags and upper panel boxplot the distribution of % amplitude.
(TIF)

S3 Fig Bar plots showing the different functional groups of the genes for the various combinations of 3-tissues. Different colors indicate the different functional groups. x-axis represents the different combination of tissues and the y-axis the percentage of circadian genes that belong to a certain functional group.
(TIF)

S4 Fig Bar plots showing the different functional groups of the genes for the various combinations of 4-tissues. Different colors indicate the different functional groups. x-axis represents the different combination of tissues and the y-axis the percentage of circadian genes that belong to a certain functional group.
(TIF)
