# Supplementary material for: Daily variation of gene expression in diverse rat tissues
Source: PLoS One. 2018 May 10;13(5):e0197258. doi: 10.1371/journal.pone.0197258 (PMC5945012; doi:10.1371/journal.pone.0197258)
Supplement: S1 Appendix — (DOCX) [file pone.0197258.s001.docx]

# Supporting information

## **S1** Appendix

### Table A: Common genes retaining circadian expression in Liver and Muscle. Δφ indicates their phase difference in hours and ΔΑ their amplitude difference in % change

| **ProbeID** | **Symbol** | **Gene Name** | **Δφ [hr]** | **ΔA [%]** |
| --- | --- | --- | --- | --- |
| 1371864_at | --- | --- | 2.25 | 50.54 |
| 1372964_at | --- | --- | 9.75 | 69.61 |
| 1389618_at | --- | --- | 2.25 | 27.51 |
| 1375760_at | --- | --- | 2.25 | 1.54 |
| 1372011_at | --- | --- | 5.25 | 0.42 |
| 1375781_at | --- | --- | 0.75 | 12.31 |
| 1377149_at | --- | --- | 3.75 | 1.01 |
| 1376341_at | --- | --- | 0.75 | 65.21 |
| 1372031_at | --- | --- | 2.25 | 38.63 |
| 1372136_at | --- | --- | 1.5 | 44.68 |
| 1389256_at | --- | --- | 0 | 26.33 |
| 1374060_at | --- | --- | 2.25 | 26.96 |
| 1375879_at | --- | --- | 3 | 53.51 |
| 1390215_at | --- | --- | 0.75 | 51.85 |
| 1372220_at | --- | --- | 3 | 42.96 |
| 1399082_at | --- | --- | 4.5 | 52.45 |
| 1388395_at | G0s2 | G0/G1switch 2 | 0.75 | 54.78 |
| 1387294_at | Sh3bp5 | SH3-domain binding protein 5 | 3.75 | 4.26 |
| 1370816_at | Nr1d1 | nuclear receptor subfamily 1, group D, member 1 | 1.5 | 48.81 |
| 1367850_at | Fcgr2a | low affinity immunoglobulin gamma Fc region receptor III-like | 1.5 | 34.01 |
| 1390430_at | Nr1d2 | nuclear receptor subfamily 1, group D, member 2 | 0.75 | 56.33 |
| 1368303_at | Per2 | period circadian clock 2 | 2.25 | 22.05 |
| 1398246_s_at | Fcgr2a | low affinity immunoglobulin gamma Fc region receptor III-like | 1.5 | 8.72 |
| 1387874_at | Dbp | D-box binding PAR bZIP transcription factor | 0 | 59.79 |
| 1367602_at | Cited2 | Cbp/p300-interacting transactivator Glu/ Asp-rich carboxy-term Dom 3 | 12 | 77.96 |
| 1370510_a_at | Arntl | aryl hydrocarbon receptor nuclear translocator-like | 0 | 2.79 |
| 1373866_at | Coq10b | coenzyme Q10B | 2.25 | 3.33 |
| 1368511_at | Bhlhe41 | Basic helix-loop-helix domain containing, class B, 3 | 1.5 | 42.32 |
| 1368249_at | Klf15 | Kruppel-like factor 15 | 4.5 | 13.66 |
| 1373108_at | Ppp1r3c | protein phosphatase 1, regulatory subunit 3C | 2.25 | 24.00 |
| 1389456_at | Clock | clock circadian regulator | 0.75 | 11.33 |
| 1390028_at | Dyrk2 | Dual-specificity tyrosine- (Y)-phosphorylation regulated kinase 2 | 1.5 | 74.95 |
| 1370847_at | Spon2 | spondin 2 | 0 | 29.39 |
| 1368488_at | Nfil3 | nuclear factor, interleukin 3 regulated | 0 | 16.48 |
| 1387703_a_at | Usp2 | Ubiquitin-specific peptidase 2 | 0 | 31.77 |
| 1388471_at | Tcp11l2 | t-complex 11 like 2 | 2.25 | 17.09 |
| 1387669_a_at | Ephx1 | Epoxide hydrolase 1, | 2.25 | 29.47 |
| 1374636_at | Jade1 | PHD finger protein 17 | 3.75 | 36.01 |
| 1386946_at | Cpt1a | Carnitine palmitoyltransferase I | 1.5 | 60.42 |
| 1369590_a_at | Ddit3 | DNA-damage-inducible transcript 3 | 0 | 65.74 |
| 1388426_at | Srebf1 | sterol regulatory element binding transcription factor 1 | 3.75 | 10.50 |
| 1373011_at | Fam134b | family with sequence similarity 134, member B | 12 | 10.64 |
| 1375677_at | Tob2 | transducer of ERBB2, 2 | 2.25 | 11.64 |
| 1370663_at | Wee1 | WEE1 G2 checkpoint kinase | 1.5 | 9.45 |
| 1387053_at | Fmo1 | Flavin-containing monooxygenase 1 | 0.75 | 33.47 |
| 1369973_at | Xdh | xanthine dehydrogenase(Xdh) | 3.75 | 26.24 |
| 1372752_at | Tspan4 | tetraspanin-4-like | 5.25 | 65.77 |
| 1369976_at | Dynll1 | Dynein, cytoplasmic, light chain 1 | 3 | 40.93 |
| 1372091_at | Mid1ip1 | MID1 interacting protein 1 | 2.25 | 16.19 |
| 1399005_at | Ppp2r5a | Protein phosphatase 2, regulatory subunit B | 3 | 20.22 |
| 1388686_at | Rcan1 | Regulator of calcineurin 1 | 8.25 | 24.23 |
| 1370019_at | Sult1a1 | sulfotransferase family 1A member 1 | 6 | 29.64 |
| 1369150_at | Pdk4 | pyruvate dehydrogenase kinase 4 | 6 | 32.46 |
| 1371953_at | Ccng2 | cyclin G2 | 3.75 | 46.23 |
| 1388901_at | Fkbp5 | FK506 binding protein 5 | 6.75 | 22.46 |
| 1371583_at | Rbm3 | RNA binding motif (RNP1, RRM) protein 3 | 0.75 | 17.57 |
| 1367802_at | Sgk1 | serum/glucocorticoid regulated kinase 1 | 9 | 14.67 |
| 1374855_at | Per1 | period circadian clock 1 | 0.75 | 58.59 |
| 1367568_a_at | Mgp | matrix Gla protein | 0.75 | 23.49 |
| 1386987_at | Il6r | Interleukin 6 receptor | 3 | 6.07 |
| 1374574_at | Fam214a | family with sequence similarity 214, member A | 9.75 | 33.66 |
| 1368549_at | Hbp1 | HMG-box transcription factor 1 | 2.25 | 25.75 |
| 1370209_at | Klf9 | Kruppel-like factor 9 | 3.75 | 43.06 |
| 1372347_at | Skil | SKI-like proto-oncogene | 7.5 | 20.39 |
| 1370912_at | Hspa1a | Heat shock 70kD protein 1B | 1.5 | 26.21 |
| 1379550_a_at | Gtf2ird1 | general transcription factor II-I repeat domain-containing protein 1-like | 2.25 | 6.35 |
| 1390117_at | Ypel2 | Yippee-like 2 | 1.5 | 30.14 |
| 1371754_at | Slc25a25 | solute carrier family 25 member 25 | 5.25 | 50.94 |
| 1390171_at | Fam76a | family with sequence similarity 76, member A | 2.25 | 23.03 |
| 1371150_at | Ccnd1 | cyclin D1 | 6.75 | 64.90 |
| 1376089_at | Ldlr | low density lipoprotein receptor | 6 | 44.33 |
| 1372452_at | Gpam | glycerol-3-phosphate acyltransferase, mitochondrial | 11.25 | 58.50 |
| 1372056_at | Cmtm6 | CKLF-like MARVEL transmembrane domain containing 6 | 3.75 | 32.23 |
| 1372701_at | Hsp90aa1 | heat shock protein 1, alpha-like | 3 | 60.62 |
| 1390042_at | Tmem140 | Transmembrane Protein 140 | 0 | 7.95 |
| 1376997_at | Fzd4 | frizzled class receptor 4 | 9 | 41.20 |
| 1367741_at | Herpud1 | homocysteine inducible ER protein with ubiquitin like domain 1 | 2.25 | 62.60 |
| 1388153_at | Acsl1 | acyl-CoA synthetase long-chain family member 1 | 7.5 | 50.65 |
| 1398913_at | Numa1 | nuclear mitotic apparatus protein 1 | 0.75 | 12.78 |
| 1373158_at | Gpr146 | G protein-coupled receptor 146 | 0.75 | 63.57 |
| 1367795_at | Ifrd1 | interferon-related developmental regulator 1 | 7.5 | 37.05 |
| 1368136_at | Tmpo | Thymopoietin | 2.25 | 0.32 |
| 1372390_at | Peg3 | paternally expressed 3 | 10.5 | 21.73 |
| 1376861_at | Mospd1 | Motile sperm domain containing 1 | 10.5 | 17.69 |
| 1388795_at | Tppp | tubulin polymerization promoting protein | 2.25 | 36.40 |
| 1388709_at | Wdr43 | WD repeat domain 43 | 6.75 | 22.56 |
| 1369919_at | Tef | TEF, PAR bZIP transcription factor | 3 | 58.22 |
| 1372164_at | Wbp1l | WW domain binding protein 1-like | 9.75 | 29.51 |
| 1376869_at | Tle4 | transducin-like enhancer of split 4 | 0.75 | 77.41 |
| 1388361_at | Ndufb10 | NADH dehydrogenase (ubiquinone) 1 beta subcomplex, 10 | 3.75 | 32.84 |
| 1390157_at | Ube2h | ubiquitin-conjugating enzyme E2H(Ube2h) Rattus norvegicus | 1.5 | 36.70 |
| 1368247_at | Hspa1a | heat shock 70kD protein 1A | 5.25 | 12.08 |
| 1372150_at | Usp10 | ubiquitin specific peptidase 10 | 10.5 | 44.04 |
| 1383169_at | LOC103691479 | Leukemia inhibitory factor receptor ? | 9 | 8.04 |
| 1373282_at | Slc25a33 | solute carrier family 25 member 33 | 2.25 | 25.52 |
| 1386864_at | Pgam1 | phosphoglycerate mutase 1 | 4.5 | 46.15 |
| 1375138_at | Timp3 | TIMP metallopeptidase inhibitor 3 | 0.75 | 30.79 |
| 1392490_at | Tmem57 | Transmembrane protein 57 | 3.75 | 14.45 |
| 1367609_at | Mif | Macrophage migration inhibitory factor | 3.75 | 13.91 |
| 1368283_at | Ehhadh | Enoyl-coenzyme A, hydratase/3- hydroxyacyl coenzyme A dehydrogenase | 12 | 25.25 |
| 1369959_at | Zfp36l1 | zinc finger protein 36, C3H type-like 1 | 2.25 | 13.76 |
| 1388364_at | Ndufs3 | NADH-ubiquinone oxidoreductase Fe-S protein 3 | 3 | 29.31 |
| 1389984_at | Jarid2 | jumonji and AT-rich interaction domain containing 2 | 1.5 | 16.67 |
| 1389064_at | Fem1c | fem-1 homolog C | 1.5 | 44.70 |
| 1367578_at | Prdx2 | peroxiredoxin 2 | 3.75 | 66.80 |
| 1374454_at | Pcmtd2 | protein-L-isoaspartate O-methyltransferase domain containing 2 | 1.5 | 0.00 |
| 1398868_at | Timm13 | Translocase of inner | 4.5 | 60.27 |
| 1387521_at | Pdcd4 | programmed cell death 4 | 2.25 | 14.96 |
| 1388136_at | Timm9 | Translocase of inner mitochondrial membrane 9 | 3.75 | 23.14 |
| 1399022_at | Clk1 | CDC-like kinase 1 | 7.5 | 3.88 |
| 1398819_at | Dnaja1 | DNAJ (Hsp40) homolog, subfamily A, member 1 | 3.75 | 6.09 |
| 1387024_at | Dusp6 | dual specificity phosphatase 6 | 9.75 | 38.42 |
| 1371780_at | Kdelr2 | KDEL endoplasmic reticulum protein retention receptor 2 | 12 | 6.51 |
| 1369467_a_at | Pfkfb1 | 6-Phosphofructo-2-kinase/fructose-2,6-bisphosphatase 1 | 0 | 58.18 |
| 1371976_at | Fam195a | Unknown | 0.75 | 46.99 |
| 1388850_at | Hsp90aa1 | heat shock protein 90, alpha (cytosolic), class A member 1 | 3 | 71.47 |
| 1389586_at | Ednrb | Endothelin Receptor Type B | 0.75 | 23.74 |
| 1376435_at | Loxl4 | Lysyl Oxidase Like 4 | 0 | 26.55 |
| 1368127_at | Neu2 | neuraminidase 2 | 4.5 | 61.21 |
| 1367708_a_at | Fasn | fatty acid synthase | 6.75 | 10.91 |
| 1388468_at | Cdc42se1 | CDC42 small effector 1 | 3 | 7.93 |
| 1388767_at | Pdcd6 | Programmed cell death | 0.75 | 63.68 |
| 1370381_at | Pnrc1 | Proline-rich nuclear receptor coactivator 1, F-box coactivator 1, F-box protein 11 | 0.75 | 62.22 |
| 1388343_at | Ndufb7 | NADH:ubiquinone oxidoreductase subunit B7 | 4.5 | 62.25 |
| 1371832_at | Leo1 | LEO1 homolog, Paf1/RNA polymerase II complex component | 3 | 12.39 |
| 1372069_at | Kank1 | KN motif and ankyrin repeat domains 1 | 2.25 | 7.20 |
| 1376636_at | Tgfbr1 | transforming growth factor, beta receptor 1 | 8.25 | 0.00 |
| 1389538_at | Nfkbia | NFKB inhibitor alpha | 0.75 | 9.75 |
| 1371246_at | Nutf2 | nuclear transport factor 2 | 4.5 | 63.18 |
| 1386981_at | Slc16a1 | solute carrier family 16 member 1 | 8.25 | 42.88 |
| 1389964_at | Ndufab1 | NADH dehydrogenase 1, alpha/beta subcomplex, 1 | 4.5 | 8.61 |
| 1371330_at | Rpl11 | ribosomal protein L11 | 3.75 | 60.06 |
| 1367463_at | Phb2 | prohibitin 2 | 2.25 | 12.37 |
| 1389088_at | Adnp | activity-dependent neuroprotector homeobox | 3.75 | 14.37 |
| 1374421_at | Baz1b | bromodomain adjacent to zinc finger domain, 1B | 4.5 | 49.28 |
| 1367670_at | Fh | Fumarate hydratase | 3 | 48.47 |
| 1374006_at | Ccbl2 | kynurenine aminotransferase 3 | 3 | 37.92 |
| 1371680_at | Gabarapl1 | GABA type A receptor associated protein like 1 | 6.75 | 10.80 |
| 1372409_at | Mad2l1bp | MAD2L1 binding protein | 3 | 63.30 |
| 1373542_at | Sphk2 | sphingosine kinase 2 | 2.25 | 24.93 |
| 1386885_at | Ech1 | enoyl-CoA hydratase 1 | 3.75 | 19.32 |
| 1372085_at | Atl2 | ADP-ribosylation factor-like 6-interacting protein 2 | 0.75 | 45.64 |
| 1374752_at | Mdfic | Myod family inhibitor domain containing | 2.25 | 26.35 |
| 1367894_at | Insig1 | insulin induced gene 1 | 2.25 | 21.70 |
| 1389003_at | Rhobtb3 | Rho-related BTB domain containing 3 | 4.5 | 7.95 |
| 1374425_at | Tle1 | transducin like enhancer of split 1 | 10.5 | 0.87 |
| 1398998_at | RGD1309748 | -- | 2.25 | 82.55 |
| 1371519_at | Etfdh | Electron-transferring-flavoprotein dehydrogenase | 11.25 | 67.46 |
| 1367577_at | Hspb1 | Heat shock 27kda protein 1 | 1.5 | 40.27 |
| 1375220_at | Ndufa11 | NADH:ubiquinone oxidoreductase subunit A11 | 6.75 | 53.06 |
| 1387244_at | Cgrrf1 | Cgr19 | 2.25 | 35.31 |
| 1389000_at | Kdm3b | lysine demethylase 3B | 3.75 | 17.22 |
| 1375658_at | Sash1 | SAM and SH3 domain containing 1 | 3 | 21.36 |
| 1388380_at | Samm50 | SAMM50 sorting and assembly machinery component | 3.75 | 31.67 |
| 1371316_at | Fau | ubiquitin-like protein fubi and ribosomal protein S30-like | 3 | 43.87 |
| 1377334_at | RT1-Ba | RT1 class II, locus Ba | 0.75 | 71.30 |
| 1371041_at | Ndufv2 | NADH:ubiquinone oxidoreductase core subunit V2 | 3.75 | 53.63 |
| 1371729_at | Ypel5 | yippee-like 5 | 4.5 | 39.73 |
| 1373240_at | Dhrs3 | Dehydrogenase/reductase 3 | 9.75 | 61.98 |
| 1374798_at | Tor1aip2 | torsin 1A interacting protein 2 | 2.25 | 46.85 |
| 1367839_at | Fdft1 | farnesyl diphosphate farnesyl transferase 1 | 3 | 8.62 |
| 1389393_at | Abhd17c | abhydrolase domain containing 17C | 6 | 54.55 |
| 1370336_at | Osgin1 | oxidative stress induced growth inhibitor 1 | 11.25 | 10.61 |
| 1389301_at | Mbnl2 | Muscle blind-like 2 isoform 1 | 7.5 | 55.10 |
| 1370824_at | Slc38a3 | solute carrier family 38, member 3 | 5.25 | 46.49 |
| 1376646_at | Popdc2 | Popeye domain containing 2 | 0 | 36.42 |
| 1368863_at | Nme3 | NME/NM23 nucleoside diphosphate kinase 3 | 1.5 | 68.10 |
| 1376700_at | Lima1 | LIM domain and actin binding 1 | 3 | 49.95 |
| 1388499_at | Timmdc1 | translocase of inner mitochondrial membrane domain containing 1 | 1.5 | 37.33 |
| 1370215_at | C1qb | complement C1q B chain | 3 | 53.05 |
| 1371955_at | Mrpl35 | mitochondrial ribosomal protein L35 | 4.5 | 51.25 |
| 1368227_at | Slc28a2 | Solute carrier family 28 (sodium- coupled nucleoside transporter) a2 | 6 | 20.67 |
| 1367771_at | Gilz | Glucocorticoid-induced leucine zipper | 1.5 | 36.42 |
| 1371249_at | Xbp1 | X-box binding protein 1 | 5.25 | 73.54 |
| 1376621_at | Wdr26 | WD repeat domain 26 | 0.75 | 27.69 |
| 1372650_at | Dnmbp | dynamin binding protein | 2.25 | 50.71 |
| 1374674_at | Atxn3 | ataxin 3 | 5.25 | 30.57 |
| 1372185_at | Ntmt1 | N-terminal Xaa-Pro-Lys N-methyltransferase 1 | 11.25 | 38.99 |
| 1388519_at | Sec61b | Sec61 β subunit | 5.25 | 54.01 |
| 1374914_at | Ppard | peroxisome proliferator-activated receptor delta | 4.5 | 77.57 |
| 1386990_at | Ebp | emopamil binding protein | 0 | 2.03 |
| 1370172_at | Sod2 | superoxide dismutase 2 | 4.5 | 0.42 |
| 1377021_at | Trmt6 | tRNA methyltransferase 6 | 3.75 | 39.59 |
| 1386937_at | Atp1b1 | Atpase, na+/k+ transporting, β 1 | 6.75 | 53.13 |
| 1386944_a_at | G6pc | glucose-6-phosphatase, catalytic subunit | 6 | 84.88 |
| 1368304_at | Fmo3 | flavin containing monooxygenase 3 | 4.5 | 68.09 |
| 1387856_at | Cnn3 | Calponin 3, acidic | 1.5 | 28.98 |
| 1370298_at | Coa3 | cytochrome C oxidase assembly factor 3 | 5.25 | 25.60 |
| 1370570_at | Nrp1 | Neuropilin 1 | 4.5 | 44.70 |
| 1388408_at | Prr13 | Proline rich 13 | 1.5 | 29.16 |
| 1398891_at | Mrpl15 | Mitochondrial ribosomal protein L15 | 4.5 | 25.00 |
| 1368087_a_at | Ptpn21 | protein tyrosine phosphatase, non-receptor type 21 | 10.5 | 77.11 |
| 1389459_at | Yae1d1 | Yae1 domain containing 1 | 1.5 | 20.35 |
| 1398752_at | 42993 | selenoprotein 15 | 0 | 12.09 |
| 1374750_at | Fnip1 | Folliculin Interacting Protein 1 | 2.25 | 13.18 |
| 1370283_at | Hspa5 | heat shock protein family A member 5 | 5.25 | 48.19 |
| 1370928_at | Litaf | lipopolysaccharide-induced TNF factor | 1.5 | 69.69 |
| 1370575_a_at | Azin1 | antizyme inhibitor 1 | 10.5 | 23.21 |
| 1372004_at | Hebp1 | Heme binding protein 1 | 6.75 | 41.41 |
| 1371254_at | Uqcrfs1 | ubiquinol-cytochrome c reductase, Rieske iron-sulfur polypeptide 1 | 3 | 27.97 |
| 1370374_at | Steap3 | STEAP family member 3 | 5.25 | 61.39 |
| 1375215_x_at | Pgpep1 | Pyroglutamyl-peptidase I | 5.25 | 50.40 |
| 1374395_at | Xpo1 | exportin 1 | 5.25 | 57.02 |
| 1387870_at | Zfp36 | zinc finger protein 36 | 0 | 40.79 |
| 1376927_at | Lrrc14b | leucine rich repeat containing 14B | 8.25 | 48.52 |
| 1367902_at | Gng11 | Guanine nucleotide binding protein , γ 11 | 2.25 | 4.64 |
| 1370370_at | Hyal2 | hyaluronoglucosaminidase 2 | 3.75 | 28.78 |
| 1367980_at | Rabep1 | rabaptin, RAB GTPase binding effector protein 1 | 0.75 | 20.91 |
| 1369092_at | Sec22a | Sec22 vesicle trafficking protein homolog a | 2.25 | 1.20 |
| 1368552_at | Grpel1 | GrpE-like 1, mitochondrial | 2.25 | 37.90 |
| 1367662_at | Hsd17b10 | hydroxysteroid dehydrogenase 10 | 6 | 22.14 |
| 1399077_at | Mtx1 | Metaxin 1 | 3.75 | 0.31 |
| 1371379_at | Mpc2 | mitochondrial pyruvate carrier 2 | 2.25 | 16.89 |
| 1367618_a_at | Gnb2l1 | Receptor For Activated C Kinase 1 | 2.25 | 41.24 |
| 1367629_at | Cox7a2 /// Cox7a2l2 | cytochrome c oxidase subunit VIIa polypeptide 2 | 3.75 | 47.63 |
| 1371900_at | Celf1 | CUGBP, Elav-like family member 1 | 3 | 5.18 |
| 1388819_at | Scamp1 | secretory carrier membrane protein 1 | 6 | 10.45 |
| 1369200_at | Nt5e | 5' nucleotidase, ecto | 0.75 | 25.81 |
| 1387116_at | Dnajb9 | DnaJ heat shock protein family member B9 | 0.75 | 15.94 |
| 1368016_at | Pecr | peroxisomal trans-2-enoyl-CoA reductase | 3 | 6.52 |
| 1376187_at | Slc35d1 | Solute Carrier Family 35 Member D1 | 5.25 | 5.56 |
| 1371329_at | Eif5a | eukaryotic translation initiation factor 5A | 3.75 | 9.33 |
| 1399154_at | Kdm2a | lysine demethylase 2A | 4.5 | 41.57 |
| 1388365_at | Atp6v0d1 | ATPase H+ transporting V0 subunit D1 | 3 | 19.06 |
| 1374449_at | Cdca3 | cell division cycle associated 3 | 7.5 | 45.30 |
| 1370277_at | Slc25a3 | solute carrier family 25 member 3 | 1.5 | 65.53 |
| 1387109_at | Por | P450 (cytochrome) oxidoreductase | 5.25 | 54.18 |
| 1367933_at | Amd1 | adenosylmethionine decarboxylase 1 | 12 | 15.38 |
| 1399159_a_at | Vamp3 | vesicle-associated membrane protein 3 | 6 | 59.29 |
| 1376796_at | Rab14 | RAB14, member RAS oncogene family | 9.75 | 12.10 |
| 1388734_at | LOC102546572 | zinc finger protein 709-like | 9 | 51.55 |
| 1398431_at | Car8 | Carbonic anhydrase viii | 2.25 | 32.60 |
| 1373270_at | Wipi1 | WD repeat domain, phosphoinositide interacting 1 | 6 | 3.59 |
| 1367597_at | Rps8 | ribosomal protein S8 | 3 | 1.21 |
| 1369943_at | Tgm2 | Transglutaminase 2 | 0 | 61.14 |
| 1371237_a_at | Mt1a /// Ttr | metallothionein 1 | 1.5 | 78.13 |
| 1389866_at | LOC100912557 | 6.8 kDa mitochondrial proteolipid | 3 | 43.73 |
| 1367755_at | Cdo1 | Cysteine dioxygenase, type i | 5.25 | 21.63 |
| 1389450_at | Wbscr22 | Williams Beuren syndrome chromosome region 22 | 2.25 | 31.96 |

### Table B: Common genes retaining circadian expression in Liver and Adipose. Δφ indicates their phase difference in hours and ΔΑ their amplitude difference in % change

| **ProbeID** | **Symbol** | **Gene Name** | **Δφ [hr]** | **ΔA [%]** |
| --- | --- | --- | --- | --- |
| 1371864_at | --- | -- | 1.5 | 42.37 |
| 1375760_at | --- | -- | 0 | 16.20 |
| 1376341_at | --- | -- | 0.75 | 7.37 |
| 1389412_at | --- | -- | 4.5 | 37.32 |
| 1388509_at | --- | -- | 0 | 28.86 |
| 1373736_at | --- | -- | 3 | 40.73 |
| 1375879_at | --- | --- | 1.5 | 52.92 |
| 1372136_at | --- | --- | 0.75 | 45.99 |
| 1371854_at | --- | -- | 3 | 52.26 |
| 1388786_at | --- | -- | 2.25 | 51.02 |
| 1399082_at | --- | -- | 0.75 | 57.42 |
| 1375781_at | --- | -- | 3 | 54.99 |
| 1372949_at | --- | -- | 3.75 | 6.83 |
| 1368303_at | Per2 | period circadian clock 2 | 2.25 | 28.26 |
| 1370510_a_at | Arntl | aryl hydrocarbon receptor nuclear translocator-like | 0.75 | 13.43 |
| 1370816_at | Nr1d1 | nuclear receptor subfamily 1, group D, member 1 | 0.75 | 13.35 |
| 1387874_at | Dbp | D-box binding PAR bZIP transcription factor | 0.75 | 52.86 |
| 1370541_at | Nr1d2 | nuclear receptor subfamily 1, group D, member 2 | 0 | 12.42 |
| 1367850_at | Fcgr2a | low affinity immunoglobulin gamma Fc region receptor III-like | 2.25 | 31.51 |
| 1398246_s_at | Fcgr2a | low affinity immunoglobulin gamma Fc region receptor III-like | 0.75 | 43.34 |
| 1370912_at | Hspa1a | Heat shock 70kD protein 1B | 0.75 | 73.83 |
| 1373114_at | Dtx4 | deltex E3 ubiquitin ligase 4 | 0 | 70.23 |
| 1373542_at | Sphk2 | sphingosine kinase 2 | 2.25 | 21.03 |
| 1367771_at | Tsc22d3 | Glucocorticoid-induced leucine zipper | 0.75 | 29.67 |
| 1374855_at | Per1 | period circadian clock 1 | 1.5 | 74.07 |
| 1370209_at | Klf9 | Kruppel-like factor 9 | 1.5 | 31.33 |
| 1388901_at | Fkbp5 | FK506 binding protein 5 | 2.25 | 54.40 |
| 1388898_at | Hsph1 | heat shock protein family H (Hsp110) member 1 | 3 | 23.38 |
| 1373195_at | Fus | Fused in sarcoma | 0 | 45.17 |
| 1368304_at | Fmo3 | flavin containing monooxygenase 3 | 3.75 | 59.19 |
| 1368247_at | Hspa1a | heat shock 70kD protein 1A | 1.5 | 58.15 |
| 1389199_at | RGD1309079 | similar to Ab2-095 | 3 | 69.29 |
| 1368488_at | Nfil3 | nuclear factor, interleukin 3 regulated | 0 | 43.84 |
| 1368177_at | Acsl3 | acyl-CoA synthetase long-chain family member 3 | 3 | 44.17 |
| 1370847_at | Spon2 | spondin 2 | 0.75 | 61.43 |
| 1374531_at | Slc6a6 | solute carrier family 6 member 6 | 11.25 | 74.97 |
| 1373158_at | Gpr146 | G protein-coupled receptor 146 | 0.75 | 3.17 |
| 1373093_at | Errfi1 | ERBB receptor feedback inhibitor 1 | 1.5 | 49.35 |
| 1371832_at | Leo1 | LEO1 homolog, Paf1/RNA polymerase II complex component | 0 | 10.62 |
| 1375677_at | Tob2 | transducer of ERBB2, 2 | 0.75 | 19.24 |
| 1369919_at | Tef | TEF, PAR bZIP transcription factor | 4.5 | 56.85 |
| 1398877_at | Stip1 | stress-induced phosphoprotein 1 | 3 | 27.55 |
| 1389456_at | Clock | clock circadian regulator | 0 | 7.14 |
| 1368021_at | Adh1 | alcohol dehydrogenase 1 | 8.25 | 74.22 |
| 1368486_at | Irs3 | insulin receptor substrate 3 | 3 | 3.07 |
| 1388271_at | Mt2A | metallothionein 2A | 0.75 | 76.61 |
| 1371505_at | Hnrnpc | heterogeneous nuclear ribonucleoproteins C1/C2-like | 1.5 | 31.28 |
| 1370570_at | Nrp1 | Neuropilin 1 | 4.5 | 73.14 |
| 1371583_at | Rbm3 | RNA binding motif (RNP1, RRM) protein 3 | 0.75 | 9.94 |
| 1368511_at | Bhlhe41 | Basic helix-loop-helix domain containing, class B, 3 | 0 | 46.53 |
| 1372390_at | Peg3 | paternally expressed 3 | 4.5 | 46.70 |
| 1367725_at | Pim3 | Pim-3 proto-oncogene, serine/threonine kinase | 2.25 | 59.43 |
| 1376435_at | Loxl4 | Lysyl Oxidase Like 4 | 1.5 | 35.19 |
| 1370991_at | Cml3 | probable N-acetyltransferase | 3.75 | 42.04 |
| 1373718_at | Tubb2a | tubulin, beta 2A class 2a | 1.5 | 73.01 |
| 1372091_at | Mid1ip1 | MID1 interacting protein 1 | 2.25 | 45.55 |
| 1373866_at | Coq10b | coenzyme Q10B | 0 | 69.19 |
| 1368549_at | Hbp1 | HMG-box transcription factor 1 | 0.75 | 46.71 |
| 1398819_at | Dnaja1 | DNAJ (Hsp40) homolog, subfamily A, member 1 | 3 | 32.65 |
| 1376788_at | Dapk1 | Death-associated protein kinase 1 | 0.75 | 31.10 |
| 1368129_at | Sfmbt1 | Scm-like with four mbt domains 1 | 3.75 | 11.96 |
| 1388239_at | Per3 | period circadian clock 3 | 0 | 77.12 |
| 1390383_at | Plin2 | perilipin 2 | 2.25 | 21.20 |
| 1374936_at | Amdhd2 | amidohydrolase domain containing 2 | 4.5 | 16.84 |
| 1368249_at | Klf15 | Kruppel-like factor 15 | 2.25 | 28.28 |
| 1374709_at | Hlf | Hepatic leukemia factor | 1.5 | 30.29 |
| 1367568_a_at | Mgp | matrix Gla protein | 0 | 3.13 |
| 1389844_at | Fkbp4 | FK506 binding protein 4 | 0.75 | 25.93 |
| 1372213_at | LOC500300 | -- | 6 | 3.75 |
| 1388674_at | Cdkn1a | cyclin-dependent kinase inhibitor 1A | 2.25 | 45.26 |
| 1370283_at | Hspa5 | heat shock protein family A member 5 | 2.25 | 37.22 |
| 1371986_at | Anp32a | Acidic Nuclear Phosphoprotein 32 Family Member A | 3.75 | 1.07 |
| 1373106_at | Zfp36l2 | zinc finger protein 36, C3H type-like 2 | 2.25 | 17.76 |
| 1387669_a_at | Ephx1 | Epoxide hydrolase 1, | 3.75 | 38.61 |
| 1371237_a_at | Mt1a | metallothionein 1 | 0.75 | 88.44 |
| 1388752_at | Bclaf1 | BCL2-associated transcription factor 1 | 1.5 | 23.51 |
| 1377287_at | Mars2 | methionine--tRNA ligase, mitochondrial-like | 3 | 13.04 |
| 1389355_at | Ier5 | immediate early response 5 | 0.75 | 33.12 |
| 1373014_at | B3gat3 | beta-1,3-glucuronyltransferase 3 | 4.5 | 57.55 |
| 1390171_at | Fam76a | family with sequence similarity 76, member A | 1.5 | 11.01 |
| 1388722_at | Dnajb1 | DnaJ heat shock protein family member B1 | 2.25 | 10.76 |
| 1372223_at | Cpeb4 | Cytoplasmic Polyadenylation Element Binding Protein 4 | 3 | 34.18 |
| 1383539_at | Prrg1 | proline rich and Gla domain 1 | 3.75 | 3.79 |
| 1367802_at | Sgk1 | serum/glucocorticoid regulated kinase 1 | 9.75 | 10.23 |
| 1367946_at | Pdlim1 | PDZ and LIM domain 1 | 5.25 | 27.14 |
| 1398431_at | Car8 | Carbonic anhydrase viii | 3.75 | 18.23 |
| 1372854_at | Ttc17 | tetratricopeptide repeat domain 17 | 2.25 | 15.53 |
| 1371929_at | Mlx | MLX, MAX dimerization protein | 3 | 4.31 |
| 1370381_at | Pnrc1 | Proline-rich nuclear receptor coactivator 1, F-box coactivator 1, F-box protein 11 | 0 | 63.78 |
| 1374522_at | Mia3 | MIA family member 3, ER export factor | 6 | 45.90 |
| 1387703_a_at | Usp2 | Ubiquitin-specific peptidase 2 | 0.75 | 2.14 |
| 1383160_at | Chordc1 | Cysteine and histidine-rich domain (CHORD)-containing, zinc-bp1 | 2.25 | 43.76 |
| 1390104_at | Irgq | immunity-related GTPase Q | 0.75 | 10.75 |
| 1398998_at | RGD1309748 | -- | 0 | 22.58 |
| 1398750_at | Calr | calreticulin | 0 | 25.31 |
| 1376936_at | Slc8b1 | solute carrier family 8 member B1 | 0.75 | 7.76 |
| 1390602_a_at | Tprn | taperin | 7.5 | 7.59 |
| 1374349_at | Ctdspl | Ctd small phosphatase-like | 0.75 | 68.97 |
| 1375892_at | Elavl1 | ELAV like RNA binding protein 1 | 3 | 26.44 |
| 1377000_at | Whamm | WAS protein homolog associated with actin, golgi membranes and microtubules | 5.25 | 33.33 |
| 1374636_at | Jade1 | PHD finger protein 17 | 2.25 | 53.77 |
| 1377016_at | Creld2 | cysteine-rich with EGF-like domains 2 | 1.5 | 34.64 |
| 1374036_at | Mcm2 | Minichromosome maintenance deficient 2 mitotin | 0.75 | 66.17 |
| 1388528_at | Fbl | Fibrillarin | 0 | 24.98 |
| 1375658_at | Sash1 | SAM and SH3 domain containing 1 | 1.5 | 3.65 |
| 1372878_at | Zfr | zinc finger RNA binding protein | 0.75 | 36.66 |
| 1389318_at | Daam1 | dishevelled associated activator of morphogenesis 1 | 3 | 13.84 |
| 1390381_at | Xpc | Xeroderma pigmentosum, | 3.75 | 11.07 |
| 1374947_at | Bcar3 | breast cancer anti-estrogen resistance 3 | 0 | 8.54 |
| 1388795_at | Tppp | tubulin polymerization promoting protein | 3.75 | 7.99 |
| 1367932_at | Hmgcs1 | 3-hydroxy-3-methylglutaryl-CoA synthase 1 | 6 | 4.92 |
| 1371350_at | Mat2a | methionine adenosyltransferase 2A | 3.75 | 43.25 |
| 1372124_at | Eif4b | eukaryotic translation initiation factor 4B | 0.75 | 3.51 |
| 1375858_at | Nprl2 | NPR2-like, GATOR1 complex subunit | 2.25 | 13.59 |
| 1368862_at | Akt1 | v akt murine thymoma viral oncogene homolog 1 | 0 | 13.96 |
| 1398960_at | Cct6a | chaperonin containing TCP1 subunit 6A | 0.75 | 54.37 |
| 1367741_at | Herpud1 | homocysteine inducible ER protein with ubiquitin like domain 1 | 0 | 53.52 |
| 1373668_at | Polr2i | Polymerase (RNA) II polypeptide I | 3 | 26.00 |
| 1370215_at | C1qb | complement C1q B chain | 4.5 | 48.13 |
| 1388945_at | Pxdc1 | PX domain containing 1 | 0 | 52.54 |
| 1372069_at | Kank1 | KN motif and ankyrin repeat domains 1 | 1.5 | 41.25 |
| 1398240_at | Hspa8 | Heat shock protein 70 | 2.25 | 56.88 |
| 1375138_at | Timp3 | TIMP metallopeptidase inhibitor 3 | 0.75 | 50.88 |
| 1377474_at | Abhd13 | abhydrolase domain containing 13 | 1.5 | 7.43 |
| 1371403_at | Cct3 | Chaperonin subunit 3 (gamma) | 0.75 | 71.38 |
| 1388582_at | Psme3 | glucose-6-phosphatase, catalytic subunit | 5.25 | 5.97 |
| 1375423_at | LOC689959 | hypothetical protein LOC689959 | 2.25 | 23.01 |
| 1371356_at | Tns2 | tensin 2 | 1.5 | 56.98 |
| 1388542_at | Use1 | unconventional SNARE in the ER 1 | 1.5 | 49.11 |
| 1372783_at | Larp4b | La ribonucleoprotein domain family, member 4B | 2.25 | 47.59 |
| 1392490_at | Tmem57 | Transmembrane protein 57 | 4.5 | 52.18 |
| 1398788_at | Pdia3 | protein disulfide isomerase family A, member 3 | 3 | 53.01 |
| 1370871_at | Hnrnpa3 | heterogeneous nuclear ribonucleoprotein A3 | 3.75 | 30.43 |
| 1369902_at | Bmf | Bcl-2 modifying factor | 2.25 | 10.29 |
| 1369200_at | Nt5e | 5' nucleotidase, ecto | 8.25 | 12.14 |
| 1372264_at | Pck1 | phosphoenolpyruvate carboxykinase 1 | 3.75 | 18.71 |
| 1372030_at | Zfyve21 | zinc finger FYVE-type containing 21 | 2.25 | 10.98 |
| 1388686_at | Rcan1 | Regulator of calcineurin 1 | 7.5 | 45.76 |
| 1375916_at | Pcmtd2 | protein-L-isoaspartate O-methyltransferase domain containing 2 | 1.5 | 17.52 |
| 1371839_at | Srsf2 | serine and arginine rich splicing factor 2 | 1.5 | 56.39 |
| 1373055_at | Tbcel | tubulin folding cofactor E-like | 0.75 | 7.48 |
| 1371693_at | Ahsa1 | Activator of heat shock 90-kDa protein ATPase homolog 1 | 1.5 | 54.91 |
| 1372024_at | Mafg | MAF bZIP transcription factor G | 0.75 | 57.08 |
| 1391078_at | Rfc1 | replication factor C subunit 1 | 3 | 6.59 |
| 1375898_at | Rbpms | RNA Binding Protein With Multiple Splicing | 4.5 | 32.53 |
| 1386987_at | Il6r | Interleukin 6 receptor | 0 | 65.18 |
| 1372844_at | Efna1 | Ephrin A1 | 6 | 79.47 |
| 1367657_at | Btg1 | BTG anti-proliferation factor 1 | 0.75 | 44.13 |
| 1376588_at | Mtmr12 | myotubularin related protein 12 | 6 | 10.62 |
| 1371754_at | Slc25a25 | solute carrier family 25 member 25 | 9 | 15.93 |
| 1376118_at | Otub2 | OTU deubiquitinase, ubiquitin aldehyde binding 2 | 5.25 | 4.80 |
| 1387805_at | Bnip3 | BCL2/adenovirus E1B 19 kDa- | 0.75 | 33.12 |
| 1371150_at | Ccnd1 | cyclin D1 | 7.5 | 46.63 |
| 1370327_at | Commd5 /// LOC100910472 | COMM domain containing 5 | 0.75 | 19.82 |
| 1371819_at | Hdac5 | histone deacetylase 5 | 2.25 | 32.41 |
| 1374932_at | Angptl8 | angiopoietin-like 8 | 2.25 | 2.08 |
| 1389988_at | Kctd2 | potassium channel tetramerization domain containing 2 | 5.25 | 33.89 |
| 1373309_at | Tmem86a | transmembrane protein 86A | 0.75 | 18.42 |
| 1367826_at | Nfe2l2 | nuclear factor, erythroid 2-like 2 | 12 | 1.67 |
| 1390272_at | Dph5 | diphthamide biosynthesis 5 | 2.25 | 49.11 |
| 1369664_at | Avpr1a | arginine vasopressin receptor 1A | 10.5 | 7.69 |
| 1373178_at | LOC103691479 | Leukemia inhibitory factor receptor ? | 0.75 | 35.03 |
| 1368223_at | Adamts1 | Adam metallopeptidase with thrombospondin 1 motif, 15 | 5.25 | 35.31 |
| 1376621_at | Wdr26 | WD repeat domain 26 | 3 | 11.90 |
| 1370336_at | Osgin1 | oxidative stress induced growth inhibitor 1 | 6.75 | 34.30 |
| 1372827_at | Ppid | peptidylprolyl isomerase D | 0.75 | 56.08 |
| 1386971_at | Ppp1r10 | protein phosphatase 1, regulatory subunit 10 | 1.5 | 33.02 |
| 1373605_at | Trak1 | trafficking kinesin protein 1 | 6 | 40.38 |
| 1377156_at | Tcf7l2 | transcription factor 7 like 2 | 4.5 | 50.78 |
| 1387294_at | Sh3bp5 | SH3-domain binding protein 5 | 9.75 | 80.83 |
| 1375335_at | Hsp90ab1 | Heat shock 90-kDa protein 1, beta | 7.5 | 30.73 |
| 1377014_at | Nim1k | NIM1 serine/threonine protein kinase | 6.75 | 54.67 |
| 1388799_at | Klhl7 | kelch-like family member 7 | 4.5 | 17.26 |
| 1373824_at | Cfdp1 | Flavin | 8.25 | 18.98 |
| 1368283_at | Ehhadh | Enoyl-coenzyme A, hydratase/3- hydroxyacyl coenzyme A dehydrogenase | 10.5 | 52.27 |
| 1388171_at | Cdk7 | cyclin-dependent kinase 7 | 10.5 | 0.38 |
| 1373302_at | Acer2 | alkaline ceramidase 2 | 5.25 | 56.00 |
| 1389685_at | LOC103690102 /// Zfp655 | zinc finger protein 655 | 0.75 | 43.22 |
| 1367539_at | Gtf2a1 | General Transcription Factor IIA Subunit 1 | 0 | 60.40 |
| 1390692_at | Ctps1 | CTP synthase 1 | 0.75 | 64.40 |
| 1398886_at | RGD1563348 | -- | 4.5 | 21.72 |
| 1368173_at | Nop58 | NOP58 ribonucleoprotein | 1.5 | 4.07 |
| 1389128_at | Wdfy3 | WD repeat and FYVE domain containing 3 | 4.5 | 2.13 |
| 1389493_at | Abtb1 | ankyrin repeat and BTB domain containing 1 | 0.75 | 53.58 |
| 1373147_at | Fbxl3 | F-box and leucine-rich repeat protein 3 | 1.5 | 36.11 |
| 1398327_at | Fermt2 | fermitin family member 2 | 3.75 | 27.78 |
| 1376101_at | Lrp6 | LDL Receptor Related Protein 6 | 2.25 | 26.06 |
| 1387836_at | Ykt6 | YKT6 v-SNARE homolog | 4.5 | 37.82 |
| 1375951_at | Thbd | thrombomodulin | 10.5 | 6.13 |
| 1372120_at | Uba5 | ubiquitin-like modifier activating enzyme 5 | 4.5 | 62.25 |
| 1373376_at | Sun1 | Sad1 and UNC84 domain containing 1 | 2.25 | 21.25 |
| 1371684_at | Pelo | pelota mRNA surveillance and ribosome rescue factor | 3 | 41.22 |
| 1376275_at | Arl5b | ADP Ribosylation Factor Like GTPase 5B | 1.5 | 11.03 |
| 1390042_at | Tmem140 | Transmembrane Protein 140 | 2.25 | 29.98 |
| 1389538_at | Nfkbia | NFKB inhibitor alpha | 2.25 | 32.20 |
| 1374568_at | Cipc | CLOCK-interacting pacemaker | 0.75 | 58.65 |
| 1367982_at | Alas1 | Aminolevulinic acid synthase 1 | 8.25 | 69.47 |
| 1368947_at | Gadd45a | growth arrest and DNA-damage-inducible, alpha | 7.5 | 36.95 |
| 1389681_at | Pvrl2 | Poliovirus receptor-related 2 | 4.5 | 56.69 |
| 1389587_at | Umps | uridine monophosphate synthetase | 3.75 | 27.93 |
| 1368321_at | Egr1 | early growth response 1 | 5.25 | 0.15 |
| 1368275_at | Msmo1 | Sterol-C4-methyl oxidase-like | 5.25 | 62.91 |
| 1376676_a_at | Mphosph8 | M-phase phosphoprotein 8 | 3.75 | 6.78 |
| 1387024_at | Dusp6 | dual specificity phosphatase 6 | 0.75 | 18.79 |
| 1372308_at | St3gal1 | ST3 beta-galactoside alpha-2,3-sialyltransferase 1 | 1.5 | 12.01 |
| 1372919_at | Gpat4 | glycerol-3-phosphate acyltransferase 4 | 0 | 2.35 |
| 1375536_at | Numb | NUMB, endocytic adaptor protein | 4.5 | 35.59 |
| 1387521_at | Pdcd4 | programmed cell death 4 | 2.25 | 17.30 |
| 1388135_at | Rpa2 | replication protein A2 | 3 | 51.60 |
| 1392607_at | Epb4.1 | erythrocyte membrane protein band 4.1 | 0 | 6.84 |
| 1370188_at | Tra2b | transformer 2 beta homolog | 7.5 | 4.49 |
| 1390097_at | Tspyl4 | TSPY-like 4 | 5.25 | 38.84 |
| 1369560_at | Gpd1 | glycerol-3-phosphate dehydrogenase 1 | 6.75 | 6.05 |
| 1389090_at | Wrnip1 | Werner helicase interacting protein 1 | 5.25 | 3.88 |
| 1388406_at | Naa60 | N-acetyltransferase 60, NatF catalytic subunit | 0 | 15.14 |
| 1372701_at | Hsp90aa1 /// LOC100362895 /// LOC103692716 | heat shock protein 1, alpha-like | 6 | 80.14 |
| 1387267_at | Ntf3 | neurotrophin 3 | 3 | 2.69 |
| 1368698_at | Atp2b2 | ATPase plasma membrane Ca2+ transporting 2 | 1.5 | 30.11 |
| 1369636_at | Sord | sorbitol dehydrogenase | 9 | 29.37 |
| 1398979_at | Angel2 | Angel Homolog 2 | 2.25 | 83.78 |
| 1374228_at | Trim47 | tripartite motif-containing 47 | 3.75 | 32.83 |
| 1374421_at | Baz1b | bromodomain adjacent to zinc finger domain, 1B | 0.75 | 30.72 |
| 1369644_at | Adgrl2 | adhesion G protein-coupled receptor L2 | 6 | 45.72 |
| 1375367_at | Pdlim2 | PDZ and LIM domain 2 | 2.25 | 13.88 |
| 1388132_at | Sfpq | splicing factor proline and glutamine rich | 1.5 | 38.46 |
| 1374676_at | Sgms1 | Sphingomyelin Synthase 1 | 1.5 | 44.60 |
| 1372116_at | Mrps2 | mitochondrial ribosomal protein S2 | 3 | 20.64 |
| 1370066_at | Keap1 | Kelch-like ECH-associated protein 1 | 6 | 17.58 |
| 1368032_at | Nolc1 | nucleolar and coiled-body phosphoprotein 1 | 1.5 | 24.91 |
| 1383013_at | Klf13 | Kruppel-like factor 13 | 1.5 | 40.67 |
| 1390107_at | Sytl2 | Synaptotagmin-like 2 | 1.5 | 45.57 |
| 1371813_at | Hirip3 | HIRA interacting protein 3 | 2.25 | 32.01 |
| 1377334_at | RT1-Ba | RT1 class II, locus Ba | 0.75 | 73.90 |
| 1398930_at | Atp6v0b | ATPase H+ transporting V0 subunit B | 7.5 | 18.86 |
| 1372340_at | Mat2b | methionine adenosyltransferase 2B | 2.25 | 15.32 |
| 1375964_at | Psph | phosphoserine phosphatase | 3 | 68.46 |
| 1388516_at | Cdv3 | carnitine deficiency-associated gene expressed in ventricle 3 | 0.75 | 35.64 |
| 1389384_at | Ndufaf4 | Hormone-regulated proliferation-associated protein 20 | 1.5 | 66.38 |
| 1370909_at | Nup62 | nucleoporin 62 | 1.5 | 29.17 |
| 1373240_at | Dhrs3 | Dehydrogenase/reductase 3 | 6 | 58.22 |
| 1369943_at | Tgm2 | Transglutaminase 2 | 2.25 | 21.38 |
| 1374945_at | LOC103692719 /// Trmt61a | tRNA methyltransferase 61A | 2.25 | 21.46 |
| 1399022_at | Clk1 | CDC-like kinase 1 | 3.75 | 24.11 |
| 1383347_at | Rev1 | REV1, DNA directed polymerase | 0 | 42.61 |
| 1387116_at | Dnajb9 | DnaJ heat shock protein family member B9 | 0.75 | 38.65 |
| 1373044_at | Eif3m | eukaryotic translation initiation factor 3, subunit M | 3 | 42.11 |
| 1377353_a_at | Tnfsf13 | TNF superfamily member 13 | 3.75 | 35.52 |
| 1367492_at | Dnajc8 | DnaJ heat shock protein family member C8 | 1.5 | 23.69 |
| 1374007_at | Slu7 | SLU7 Homolog, Splicing Factor | 0.75 | 45.56 |
| 1373826_at | Ypel5 | yippee-like 5 | 1.5 | 19.33 |
| 1387914_at | Cyp27a1 | cytochrome P450, family 27, subfamily a, polypeptide 1 | 6.75 | 10.94 |
| 1375080_at | Slc25a45 | solute carrier family 25, member 45 | 9.75 | 9.78 |
| 1387780_at | Dnaja2 | DNAJ (Hsp40) homolog, subfamily A, member 2 | 0.75 | 23.76 |

### Table C: Common genes retaining circadian expression in Liver and Lung. Δφ indicates their phase difference in hours and ΔΑ their amplitude difference in % change

| **ProbeID** | **Symbol** | **Gene Name** | **Δφ [hr]** | **ΔA [%]** |
| --- | --- | --- | --- | --- |
| 1371864_at | --- | --- | 1.5 | 32.94 |
| 1376341_at | --- | --- | 0.75 | 16.48 |
| 1398343_at | --- | --- | 2.25 | 10.71 |
| 1373736_at | --- | --- | 3 | 4.73 |
| 1372103_at | --- | --- | 2.25 | 9.76 |
| 1388146_at | --- | --- | 2.25 | 24.48 |
| 1374748_at | --- | --- | 1.5 | 0.66 |
| 1374611_at | --- | --- | 0.75 | 26.36 |
| 1375088_at | --- | --- | 3 | 29.19 |
| 1372011_at | --- | --- | 4.5 | 53.46 |
| 1388985_at | --- | --- | 5.25 | 8.02 |
| 1372505_at | --- | --- | 1.5 | 58.81 |
| 1399082_at | --- | --- | 3 | 72.02 |
| 1371903_at | --- | --- | 11.25 | 16.06 |
| 1373626_at | --- | --- | 0 | 24.95 |
| 1371595_at | --- | --- | 8.25 | 51.55 |
| 1371796_at | --- | --- | 2.25 | 62.50 |
| 1377149_at | --- | --- | 6 | 27.33 |
| 1388966_at | --- | --- | 1.5 | 24.09 |
| 1388942_at | --- | --- | 0.75 | 69.34 |
| 1373513_at | --- | --- | 11.25 | 65.56 |
| 1389618_at | --- | -- | 6 | 75.15 |
| 1390215_at | --- | --- | 1.5 | 42.97 |
| 1372261_at | --- | --- | 2.25 | 11.94 |
| 1368046_at | --- | --- | 6.75 | 46.74 |
| 1372136_at | --- | --- | 2.25 | 61.37 |
| 1390457_at | --- | --- | 8.25 | 41.59 |
| 1385273_at | --- | --- | 0.75 | 17.17 |
| 1373740_at | --- | --- | 5.25 | 70.63 |
| 1382644_at | --- | --- | 1.5 | 10.20 |
| 1374241_at | --- | --- | 8.25 | 77.29 |
| 1372031_at | --- | --- | 2.25 | 68.41 |
| 1372220_at | --- | --- | 3 | 17.56 |
| 1372949_at | --- | --- | 3 | 44.41 |
| 1374731_at | --- | --- | 0 | 19.54 |
| 1372199_at | --- | --- | 0.75 | 61.73 |
| 1370173_at | --- | --- | 3 | 63.63 |
| 1373703_at | --- | --- | 4.5 | 54.52 |
| 1371699_at | --- | --- | 9 | 85.87 |
| 1372022_at | --- | --- | 1.5 | 67.37 |
| 1372642_at | --- | --- | 6.75 | 74.99 |
| 1382516_at | --- | --- | 6 | 64.40 |
| AFFX_Rat_beta-actin_3_at | --- | --- | 2.25 | 77.07 |
| 1389412_at | --- | --- | 0.75 | 65.55 |
| 1373854_at | --- | --- | 3 | 63.10 |
| 1390430_at | Nr1d2 | nuclear receptor subfamily 1, group D, member 2 | 0.75 | 15.18 |
| 1367771_at | Tsc22d3 | Glucocorticoid-induced leucine zipper | 1.5 | 44.71 |
| 1368303_at | Per2 | period circadian clock 2 | 3 | 24.96 |
| 1368304_at | Fmo3 | flavin containing monooxygenase 3 | 3.75 | 80.60 |
| 1370816_at | Nr1d1 | nuclear receptor subfamily 1, group D, member 1 | 0.75 | 48.24 |
| 1370510_a_at | Arntl | aryl hydrocarbon receptor nuclear translocator-like | 0.75 | 27.08 |
| 1370847_at | Spon2 | spondin 2 | 0.75 | 83.83 |
| 1367982_at | Alas1 | Aminolevulinic acid synthase 1 | 3 | 15.76 |
| 1369919_at | Tef | TEF, PAR bZIP transcription factor | 1.5 | 62.81 |
| 1370209_at | Klf9 | Kruppel-like factor 9 | 2.25 | 27.76 |
| 1373114_at | Dtx4 | deltex E3 ubiquitin ligase 4 | 1.5 | 60.81 |
| 1387874_at | Dbp | D-box binding PAR bZIP transcription factor | 0.75 | 17.05 |
| 1367741_at | Herpud1 | homocysteine inducible ER protein with ubiquitin like domain 1 | 0 | 26.31 |
| 1375138_at | Timp3 | TIMP metallopeptidase inhibitor 3 | 2.25 | 12.56 |
| 1367577_at | Hspb1 | Heat shock 27kda protein 1 | 3 | 42.29 |
| 1374709_at | Hlf | Hepatic leukemia factor | 3.75 | 37.30 |
| 1368249_at | Klf15 | Kruppel-like factor 15 | 1.5 | 44.65 |
| 1372752_at | Tspan4 | tetraspanin-4-like | 5.25 | 30.59 |
| 1388901_at | Fkbp5 | FK506 binding protein 5 | 0.75 | 58.31 |
| 1371583_at | Rbm3 | RNA binding motif (RNP1, RRM) protein 3 | 1.5 | 18.35 |
| 1370283_at | Hspa5 | heat shock protein family A member 5 | 2.25 | 9.31 |
| 1368778_at | Slc6a6 | solute carrier family 6 member 6 | 8.25 | 75.78 |
| 1367814_at | Atp1b1 | Atpase, na+/k+ transporting, β 1 | 6 | 47.32 |
| 1367905_at | Enpp3 | Ectonucleotide pyrophosphatase/phosphodiesterase 3 | 1.5 | 67.00 |
| 1368488_at | Nfil3 | nuclear factor, interleukin 3 regulated | 0 | 35.11 |
| 1367755_at | Cdo1 | Cysteine dioxygenase, type i | 6 | 76.77 |
| 1390199_at | Clock | clock circadian regulator | 0.75 | 40.55 |
| 1372101_at | Plpp3 | phospholipid phosphatase 3 | 6 | 48.76 |
| 1368422_at | Meox2 | Mesenchyme homeobox 2 | 4.5 | 32.56 |
| 1371832_at | Leo1 | LEO1 homolog, Paf1/RNA polymerase II complex component | 0 | 9.40 |
| 1374855_at | Per1 | period circadian clock 1 | 1.5 | 43.14 |
| 1369943_at | Tgm2 | Transglutaminase 2 | 2.25 | 2.82 |
| 1387109_at | Por | P450 (cytochrome) oxidoreductase | 3 | 67.53 |
| 1370019_at | Sult1a1 | sulfotransferase family 1A member 1 | 5.25 | 41.41 |
| 1398431_at | Car8 | Carbonic anhydrase viii | 6.75 | 23.76 |
| 1374752_at | Mdfic | Myod family inhibitor domain containing | 0.75 | 8.67 |
| 1372755_at | Mal2 | mal, T-cell differentiation protein 2 | 1.5 | 47.61 |
| 1387703_a_at | Usp2 | Ubiquitin-specific peptidase 2 | 0 | 44.10 |
| 1390255_at | Ablim3 | Actin binding lim protein family, 3 | 11.25 | 9.89 |
| 1398750_at | Calr | calreticulin | 0 | 31.57 |
| 1368486_at | Irs3 | insulin receptor substrate 3 | 2.25 | 2.33 |
| 1369636_at | Sord | sorbitol dehydrogenase | 6 | 4.76 |
| 1373195_at | Fus | Fused in sarcoma | 1.5 | 15.11 |
| 1374798_at | Tor1aip2 | torsin 1A interacting protein 2 | 3.75 | 3.69 |
| 1371774_at | Sat1 | spermidine/spermine N1-acetyl transferase 1 | 3.75 | 20.62 |
| 1386946_at | Cpt1a | Carnitine palmitoyltransferase I | 2.25 | 73.27 |
| 1390731_at | Rasgef1b | Rasgef domain family, 1b | 0 | 42.70 |
| 1388686_at | Rcan1 | Regulator of calcineurin 1 | 7.5 | 28.91 |
| 1388331_at | Hsp90b1 | Heat shock protein 90-kDa beta | 0.75 | 30.74 |
| 1372213_at | LOC500300 | -- | 9 | 39.13 |
| 1368247_at | Hspa1a | heat shock 70kD protein 1A | 0 | 55.86 |
| 1369725_at | Adap2 | Arfgap with dual ph domains 2 | 2.25 | 20.21 |
| 1372308_at | St3gal1 | ST3 beta-galactoside alpha-2,3-sialyltransferase 1 | 5.25 | 7.76 |
| 1387294_at | Sh3bp5 | SH3-domain binding protein 5 | 6 | 59.09 |
| 1389355_at | Ier5 | immediate early response 5 | 1.5 | 37.01 |
| 1388898_at | Hsph1 | heat shock protein family H (Hsp110) member 1 | 0.75 | 31.67 |
| 1386895_at | Maged1 | MAGE family member D1 | 5.25 | 34.37 |
| 1373093_at | Errfi1 | ERBB receptor feedback inhibitor 1 | 0.75 | 26.10 |
| 1375336_at | Hsp90ab1 | Heat shock 90-kDa protein 1, beta | 0.75 | 13.01 |
| 1370912_at | Hspa1a | Heat shock 70kD protein 1B | 1.5 | 64.47 |
| 1372512_at | Stx18 | Syntaxin 18 | 3.75 | 25.74 |
| 1388395_at | G0s2 | G0/G1switch 2 | 1.5 | 19.52 |
| 1388426_at | Srebf1 | sterol regulatory element binding transcription factor 1 | 0.75 | 28.26 |
| 1370991_at | Cml3 | probable N-acetyltransferase | 3.75 | 26.85 |
| 1371505_at | Hnrnpc | heterogeneous nuclear ribonucleoproteins C1/C2-like | 3 | 59.61 |
| 1372364_a_at | Ntan1 | N-terminal asparagine amidase | 0 | 21.86 |
| 1389319_at | Ergic1 | Endoplasmic reticulum-golgi intermediate compartment 1 | 0.75 | 57.08 |
| 1376813_at | Lpcat3 | lysophosphatidylcholine acyltransferase 3 | 1.5 | 2.68 |
| 1388850_at | Hsp90aa1 | heat shock protein 90, alpha (cytosolic), class A member 1 | 1.5 | 68.25 |
| 1372701_at | Hsp90aa1 | heat shock protein 1, alpha-like | 1.5 | 61.64 |
| 1388422_at | Lims2 | LIM and senescent cell antigen like domains 2 | 11.25 | 27.10 |
| 1372352_at | Manf | mesencephalic astrocyte-derived neurotrophic factor | 0.75 | 16.13 |
| 1388662_at | Rora | RAR related orphan receptor a | 9 | 44.36 |
| 1377287_at | Mars2 | methionine--tRNA ligase, mitochondrial-like | 3 | 21.67 |
| 1388271_at | Mt2A | metallothionein 2A | 2.25 | 80.22 |
| 1390107_at | Sytl2 | Synaptotagmin-like 2 | 3 | 14.15 |
| 1374591_at | Ptprd | Protein tyrosine phosphatase, receptor type, d | 6.75 | 28.87 |
| 1372612_at | Dynll2 | dynein light chain LC8-type 2 | 5.25 | 31.69 |
| 1398240_at | Hspa8 | Heat shock protein 70 | 0 | 52.72 |
| 1371237_a_at | Mt1a /// Ttr | metallothionein 1 | 0 | 94.94 |
| 1388640_at | Sumo3 | Smt3 suppressor of mif two 3 homolog 3 | 1.5 | 31.44 |
| 1374349_at | Ctdspl | Ctd small phosphatase-like | 0 | 47.06 |
| 1373147_at | Fbxl3 | F-box and leucine-rich repeat protein 3 | 0.75 | 31.35 |
| 1374251_at | Kcnj15 | potassium voltage-gated channel subfamily J member 15 | 0.75 | 67.76 |
| 1379361_at | Pex11a | Peroxisomal biogenesis factor 11 α | 0 | 19.93 |
| 1371014_at | Plcb1 | Phospholipase c, β 1 | 9.75 | 73.69 |
| 1373243_at | Pmvk | phosphomevalonate kinase | 3.75 | 39.18 |
| 1373312_at | Pnkd | paroxysmal nonkinesigenic dyskinesia | 0 | 50.77 |
| 1387214_at | Ces1e | Carboxylesterase 1 | 3 | 70.95 |
| 1373282_at | Slc25a33 | solute carrier family 25 member 33 | 3 | 25.41 |
| 1372522_at | Arglu1 | arginine and glutamate rich 1 | 8.25 | 1.67 |
| 1389521_at | Ivns1abp | Influenza virus ns1a binding protein | 2.25 | 27.88 |
| 1376860_at | Slc22a23 | solute carrier family 22, member 23 | 8.25 | 2.01 |
| 1390697_at | Gemin8 | Gem Nuclear Organelle Associated Protein 8 | 2.25 | 37.22 |
| 1367586_at | Ldha | lactate dehydrogenase A | 3.75 | 3.16 |
| 1373829_at | Fgfr2 | Fibroblast growth factor receptor 2 | 3 | 63.67 |
| 1372878_at | Zfr | zinc finger RNA binding protein | 3.75 | 52.79 |
| 1372004_at | Hebp1 | Heme binding protein 1 | 6 | 37.61 |
| 1373542_at | Sphk2 | sphingosine kinase 2 | 1.5 | 19.12 |
| 1367609_at | Mif | Macrophage migration inhibitory factor | 3 | 35.38 |
| 1377194_a_at | Ccdc90b | Coiled-coil domain containing 90b | 6 | 37.20 |
| 1370642_s_at | Pdgfrb | platelet derived growth factor receptor beta | 0 | 22.15 |
| 1368511_at | Bhlhe41 | Basic helix-loop-helix domain containing, class B, 3 | 0 | 26.17 |
| 1387896_at | Scp2 | Sterol carrier protein 2 | 0.75 | 13.88 |
| 1370928_at | Litaf | lipopolysaccharide-induced TNF factor | 0.75 | 59.97 |
| 1369467_a_at | Pfkfb1 | 6-Phosphofructo-2-kinase/fructose-2,6-bisphosphatase 1 | 4.5 | 28.99 |
| 1373866_at | Coq10b | coenzyme Q10B | 0 | 71.22 |
| 1398795_at | Dars | aspartyl-tRNA synthetase | 8.25 | 16.06 |
| 1372056_at | Cmtm6 | CKLF-like MARVEL transmembrane domain containing 6 | 0.75 | 70.67 |
| 1374065_at | Met | Met proto-oncogene | 1.5 | 41.68 |
| 1372390_at | Peg3 | paternally expressed 3 | 9 | 13.47 |
| 1389587_at | Umps | uridine monophosphate synthetase | 5.25 | 15.92 |
| 1368016_at | Pecr | peroxisomal trans-2-enoyl-CoA reductase | 4.5 | 28.67 |
| 1371310_s_at | Serpinh1 | serpin family H member 1 | 2.25 | 0.52 |
| 1389393_at | Abhd17c | abhydrolase domain containing 17C | 4.5 | 54.41 |
| 1373842_at | Wasl | Wiskott-Aldrich syndrome-like | 5.25 | 61.20 |
| 1373043_at | Sdf2l1 | stromal cell-derived factor 2-like 1 | 2.25 | 41.54 |
| 1390042_at | Tmem140 | Transmembrane Protein 140 | 1.5 | 18.31 |
| 1370336_at | Osgin1 | oxidative stress induced growth inhibitor 1 | 11.25 | 58.90 |
| 1371544_at | Erh | enhancer of rudimentary homolog (Drosophila) | 7.5 | 0.83 |
| 1398877_at | Stip1 | stress-induced phosphoprotein 1 | 1.5 | 32.54 |
| 1390383_at | Plin2 | perilipin 2 | 2.25 | 53.08 |
| 1388408_at | Prr13 | Proline rich 13 | 0 | 17.62 |
| 1388858_at | Map2k3 | mitogen activated protein kinase kinase 3 | 3 | 35.78 |
| 1373185_at | Ssr2 | signal sequence receptor subunit 2 | 3 | 8.48 |
| 1389199_at | RGD1309079 | similar to Ab2-095 | 0.75 | 85.82 |
| 1390021_at | Hist1h2bcl1 | histone cluster 1, H2bh | 0.75 | 0.92 |
| 1389287_at | Tead1 | Tea domain family 1 | 6.75 | 71.43 |
| 1392490_at | Tmem57 | Transmembrane protein 57 | 6 | 35.21 |
| 1389308_at | Dnajb11 | DnaJ heat shock protein family (Hsp40) member B11 | 0 | 9.73 |
| 1375645_at | Szrd1 | SUZ RNA Binding Domain Containing 1 | 1.5 | 67.71 |
| 1377143_at | Slc35b1 | solute carrier family 35, member B1 | 0.75 | 14.16 |
| 1368127_at | Neu2 | neuraminidase 2 | 0.75 | 61.86 |
| 1377016_at | Creld2 | cysteine-rich with EGF-like domains 2 | 0 | 33.52 |
| 1389519_at | Psmd8 | Proteasome 26s subunit, non-atpase, 8 | 2.25 | 4.90 |
| 1371729_at | Ypel5 | yippee-like 5 | 0.75 | 42.80 |
| 1373864_at | Map4k4 | mitogen-activated protein kinase kinase kinase kinase 4(Map4k4) Rattus | 1.5 | 73.87 |
| 1375965_at | Ube2k | Ubiquitin-conjugating enzyme e2k | 6.75 | 13.79 |
| 1376880_at | Tbk1 | Tank-binding kinase 1 | 0.75 | 12.37 |
| 1371791_at | Surf4 | Surfeit 4 | 1.5 | 57.48 |
| 1371622_at | Dph1 | diphthamide biosynthesis 1 | 9.75 | 26.78 |
| 1389844_at | Fkbp4 | FK506 binding protein 4 | 0.75 | 15.07 |
| 1387395_at | Adora2b | Adenosine a2b receptor | 2.25 | 60.07 |
| 1373719_at | Map4k3 | mitogen-activated protein kinase kinase kinase kinase 3 | 1.5 | 0.99 |
| 1375612_at | Hnrnpa1 | Heterogeneous nuclear ribonucleoprotein a1 | 6.75 | 14.98 |
| 1368992_a_at | Srsf5 | serine and arginine rich splicing factor 5 | 0.75 | 9.22 |
| 1389965_at | Tgoln2 | Trans-Golgi Network Protein 2 | 2.25 | 0.84 |
| 1374903_at | Gcnt2 | beta-1,6- Golgi, glycoprotein synthesis Acetylglucosaminyltransferase family polypeptide 3 | 3.75 | 88.32 |
| 1387145_at | Gjb1 | Gap junction protein, β 1, 32kda | 5.25 | 68.16 |
| 1390171_at | Fam76a | family with sequence similarity 76, member A | 3 | 16.45 |
| 1390274_at | Plxna2 | Plexin a2 | 8.25 | 8.11 |
| 1371629_at | Cxxc5 | CXXC finger protein 5 | 3 | 14.25 |
| 1368177_at | Acsl3 | acyl-CoA synthetase long-chain family member 3 | 0.75 | 46.27 |
| 1374346_at | Aifm2 | Apoptosis-inducing factor, mitochondrion-associated, 2 | 3.75 | 57.19 |
| 1383169_at | LOC103691479 | Leukemia inhibitory factor receptor ? | 3 | 60.56 |
| 1388795_at | Tppp | tubulin polymerization promoting protein | 2.25 | 6.07 |
| 1367902_at | Gng11 | Guanine nucleotide binding protein , γ 11 | 2.25 | 29.87 |
| 1372027_at | Spata13 | spermatogenesis associated 13 | 0.75 | 11.64 |
| 1398894_at | Commd3 | Comm domain containing 3 | 4.5 | 30.16 |
| 1371730_at | Lonp2 | lon peptidase 2, peroxisomal | 0.75 | 58.30 |
| 1368223_at | Adamts1 | Adam metallopeptidase with thrombospondin 1 motif, 15 | 3 | 39.93 |
| 1368596_at | Sik1 | Salt-inducible kinase 1 | 5.25 | 40.50 |
| 1371249_at | Xbp1 | X-box binding protein 1 | 8.25 | 60.09 |
| 1368549_at | Hbp1 | HMG-box transcription factor 1 | 0 | 67.07 |
| 1389402_at | Csrnp1 | cysteine and serine rich nuclear protein 1 | 1.5 | 5.60 |
| 1388239_at | Per3 | period circadian clock 3 | 0.75 | 58.47 |
| 1370964_at | Ass1 | argininosuccinate synthase 1 | 1.5 | 54.47 |
| 1376623_at | Tmem204 | Transmembrane protein 204 | 5.25 | 10.69 |
| 1371636_at | Ankrd13c | ankyrin repeat domain 13C | 1.5 | 42.79 |
| 1367568_a_at | Mgp | matrix Gla protein | 0.75 | 40.47 |
| 1388883_at | Pold4 | Polymerase , δ 4 | 9.75 | 26.84 |
| 1375916_at | Pcmtd2 | protein-L-isoaspartate O-methyltransferase domain containing 2 | 2.25 | 60.75 |
| 1372613_at | Bdh2 | 3-hydroxybutyrate dehydrogenase, type 2 | 5.25 | 43.25 |
| 1371929_at | Mlx | MLX, MAX dimerization protein | 6 | 33.98 |
| 1375898_at | Rbpms | RNA Binding Protein With Multiple Splicing | 7.5 | 26.20 |
| 1372901_at | Nhlrc3 | NHL repeat containing 3 | 0.75 | 52.16 |
| 1376646_at | Popdc2 | Popeye domain containing 2 | 3 | 11.87 |
| 1379550_a_at | Gtf2ird1 | general transcription factor II-I repeat domain-containing protein 1-like | 0.75 | 47.13 |
| 1375663_at | Ube2f | Ubiquitin-conjugating enzyme e2f | 4.5 | 0.93 |
| 1387669_a_at | Ephx1 | Epoxide hydrolase 1, | 3.75 | 10.02 |
| 1398998_at | RGD1309748 | -- | 0 | 54.40 |
| 1368871_at | Map3k1 | mitogen-activated protein kinase kinase kinase 1-like | 3 | 56.56 |
| 1376662_at | Ppp1r12a | protein phosphatase 1, regulatory subunit 12A | 0.75 | 38.13 |
| 1367723_a_at | Sh2b3 | SH2B adaptor protein 3 | 0 | 53.44 |
| 1386864_at | Pgam1 | phosphoglycerate mutase 1 | 0.75 | 49.04 |
| 1369654_at | Prkaa2 | protein kinase AMP-activated catalytic subunit alpha 2 | 0 | 21.85 |
| 1370180_at | Nudt4 | nudix hydrolase 4 | 12 | 0.00 |
| 1390117_at | Ypel2 | Yippee-like 2 | 1.5 | 61.05 |
| 1373240_at | Dhrs3 | Dehydrogenase/reductase 3 | 9 | 46.13 |
| 1399143_at | Ube2n | ubiquitin-conjugating enzyme E2N | 5.25 | 15.02 |
| 1388857_at | Sec23b | Sec23 homolog B, coat complex II component | 7.5 | 29.15 |
| 1374568_at | Cipc | CLOCK-interacting pacemaker | 3 | 62.59 |
| 1370414_at | Rab38 | RAB38, member RAS oncogene family | 1.5 | 56.89 |
| 1368173_at | Nop58 | NOP58 ribonucleoprotein | 9 | 54.30 |
| 1398946_at | Mrps16 | mitochondrial ribosomal protein S16 | 1.5 | 34.66 |
| 1373055_at | Tbcel | tubulin folding cofactor E-like | 0.75 | 12.59 |
| 1374335_at | Gata6 | GATA Binding Protein 6 | 1.5 | 52.94 |
| 1370057_at | Csrp1 | cysteine and glycine-rich protein 1 | 2.25 | 13.04 |
| 1383080_at | Lamp2 | lysosomal-associated membrane protein 2 | 2.25 | 49.02 |
| 1368021_at | Adh1 | alcohol dehydrogenase 1 | 10.5 | 14.72 |
| 1372290_at | Nelfe | negative elongation factor complex member E | 0.75 | 61.33 |
| 1367591_at | Prdx3 | peroxiredoxin 3 | 0.75 | 23.01 |
| 1378016_at | Eml4 | echinoderm microtubule associated protein like 4 | 3 | 4.79 |
| 1374505_at | Lyrm5 | electron transfer flavoprotein regulatory factor 1 | 11.25 | 39.20 |
| 1369959_at | Zfp36l1 | zinc finger protein 36, C3H type-like 1 | 2.25 | 45.68 |
| 1372160_at | Blcap | Bladder cancer associated protein | 6.75 | 68.95 |
| 1373901_at | Cdk2ap2 | cyclin-dependent kinase 2 associated protein 2 | 1.5 | 17.33 |
| 1376861_at | Mospd1 | Motile sperm domain containing 1 | 9.75 | 44.63 |
| 1371953_at | Ccng2 | cyclin G2 | 0.75 | 66.84 |
| 1389003_at | Rhobtb3 | Rho-related BTB domain containing 3 | 0.75 | 34.52 |
| 1374416_at | Coa4 | Coiled-coil-helix-coiled-coil-helix domain containing 8 | 11.25 | 13.34 |
| 1373777_at | Rgs16 | Regulator of G-protein signaling 16 | 0 | 56.05 |
| 1368436_at | Nudc | nuclear migration protein nudC-like | 2.25 | 26.44 |
| 1376214_at | Mmachc | Methylmalonic aciduria cblc type, with homocystinuria | 6 | 28.94 |
| 1377060_at | Mccc2 | Methylcrotonoyl-coenzyme a carboxylase 2 | 9.75 | 1.40 |
| 1372920_at | Prodh | proline dehydrogenase 1 | 0 | 31.81 |
| 1370045_at | Polg | DNA polymerase gamma, catalytic subunit | 9.75 | 59.60 |
| 1373970_at | Il33 | interleukin 33 | 4.5 | 22.77 |
| 1370570_at | Nrp1 | Neuropilin 1 | 3.75 | 55.29 |
| 1398327_at | Fermt2 | fermitin family member 2 | 0 | 10.06 |
| 1371693_at | Ahsa1 | Activator of heat shock 90-kDa protein ATPase homolog 1 | 3 | 44.58 |
| 1370295_at | Nme1 | NME/NM23 nucleoside diphosphate kinase 1 | 5.25 | 12.63 |
| 1367627_at | Gatm | glycine amidinotransferase | 4.5 | 44.04 |
| 1372774_at | Entpd5 | ectonucleoside triphosphate diphosphohydrolase 5 | 2.25 | 16.04 |
| 1387361_s_at | Pgk1 | Phosphoglycerate kinase 1 | 2.25 | 74.25 |
| 1368563_at | Aspa | Aspartoacylase | 3.75 | 51.96 |
| 1370946_at | Nfix | nuclear factor 1 X | 1.5 | 85.28 |
| 1374156_at | Mpp5 | Membrane protein, palmitoylated 5 | 0.75 | 43.75 |
| 1388410_at | Ugp2 | UDP-glucose pyrophosphorylase 2 | 2.25 | 81.93 |
| 1388338_at | Ppp2r4 | protein phosphatase 2 phosphatase activator | 0 | 21.17 |
| 1373140_at | Il6st | interleukin 6 signal transducer | 7.5 | 36.19 |
| 1398950_at | Mlec | Sciellin | 3.75 | 45.00 |
| 1371856_at | Pnrc2 | proline-rich nuclear receptor coactivator 2 | 0 | 48.97 |
| 1371798_at | Gna12 | G protein subunit alpha 12 | 2.25 | 15.06 |
| 1398886_at | RGD1563348 | -- | 6.75 | 27.18 |
| 1369415_at | Bhlhe40 | basic helix-loop-helix family, member e40 | 3.75 | 58.80 |
| 1375056_at | Ociad2 | OCIA Domain Containing 2 | 9.75 | 11.88 |
| 1389064_at | Fem1c | fem-1 homolog C | 3 | 43.41 |
| 1371822_at | Polr3d | RNA polymerase III subunit D | 8.25 | 57.22 |
| 1388758_at | Ogt | O-linked N-acetylglucosamine (GlcNAc) transferase | 3.75 | 40.70 |
| 1376837_at | H6pd | Hexose-6-Phosphate Dehydrogenase/Glucose 1-Dehydrogenase | 2.25 | 23.55 |
| 1386987_at | Il6r | Interleukin 6 receptor | 3.75 | 71.76 |
| 1371809_at | Mrps18b | mitochondrial ribosomal protein S18B | 1.5 | 19.73 |
| 1369976_at | Dynll1 | Dynein, cytoplasmic, light chain 1 | 3.75 | 65.66 |
| 1368038_at | Synj2bp | synaptojanin 2 binding protein | 2.25 | 10.81 |
| 1389010_at | Lta4h | leukotriene A4 hydrolase | 3 | 30.05 |
| 1372704_at | Ostc | Oligosaccharyltransferase complex subunit | 2.25 | 0.79 |
| 1369092_at | Sec22a | Sec22 vesicle trafficking protein homolog a | 3 | 7.71 |
| 1372681_at | Stxbp6 | syntaxin binding protein 6 | 6.75 | 14.50 |
| 1371840_at | S1pr1 | sphingosine-1-phosphate receptor 1 | 12 | 70.76 |
| 1390373_at | Smad5 | SMAD family member 5 | 3.75 | 17.11 |
| 1367602_at | Cited2 | Cbp/p300-interacting transactivator Glu/ Asp-rich carboxy-term Dom 3 | 12 | 7.73 |
| 1373269_at | Smim13 | Small Integral Membrane Protein 13 | 8.25 | 42.35 |
| 1388874_at | Mtss1 | Metastasis suppressor 1 | 8.25 | 71.12 |
| 1393516_at | Slc16a12 | Solute carrier family 16, member | 2.25 | 6.94 |
| 1374105_at | Higd1a | HIG1 domain family, member 1A | 2.25 | 51.07 |
| 1383013_at | Klf13 | Kruppel-like factor 13 | 1.5 | 70.53 |
| 1372582_at | Csnk1g3 | casein kinase 1, gamma 3 | 9.75 | 34.07 |
| 1368136_at | Tmpo | Thymopoietin | 2.25 | 54.84 |
| 1367539_at | Gtf2a1 | General Transcription Factor IIA Subunit 1 | 2.25 | 53.22 |
| 1367695_at | Qdpr | quinoid dihydropteridine reductase | 5.25 | 12.34 |
| 1398913_at | Numa1 | nuclear mitotic apparatus protein 1 | 3.75 | 47.98 |
| 1371684_at | Pelo | pelota mRNA surveillance and ribosome rescue factor | 7.5 | 50.94 |
| 1372619_at | Mrpl49 | mitochondrial ribosomal protein L49 | 3.75 | 54.82 |
| 1370563_at | Akr1c14 | aldo-keto reductase family 1, member C14 | 10.5 | 19.87 |
| 1368073_at | Irf1 | interferon regulatory factor 1 | 0 | 46.88 |
| 1389303_at | Mif4gd | MIF4G domain containing | 0.75 | 17.52 |
| 1373047_at | Prkci | protein kinase C, iota | 6 | 36.31 |
| 1388442_at | Canx | calnexin | 1.5 | 42.50 |
| 1375870_a_at | Rbms1 | RNA binding motif, single stranded interacting protein 1 | 6 | 28.26 |
| 1387855_at | Gdi1 | GDP dissociation inhibitor 1 | 4.5 | 28.03 |
| 1375367_at | Pdlim2 | PDZ and LIM domain 2 | 2.25 | 50.16 |
| 1373104_at | Specc1l | sperm antigen with calponin homology and coiled-coil domains 1-like | 3.75 | 33.12 |
| 1388722_at | Dnajb1 | DnaJ heat shock protein family member B1 | 3 | 33.01 |
| 1373501_at | Nek7 | NIMA-related kinase 7 | 4.5 | 34.73 |
| 1368573_at | Kpnb1 | karyopherin subunit beta 1 | 9 | 9.68 |
| 1368490_at | Cd14 | CD14 molecule | 5.25 | 47.66 |
| 1398296_at | Gde1 | glycerophosphodiester phosphodiesterase 1 | 2.25 | 61.27 |
| 1372468_at | Adgre5 | adhesion G protein-coupled receptor E5 | 0.75 | 10.42 |
| 1398788_at | Pdia3 | protein disulfide isomerase family A, member 3 | 2.25 | 21.65 |
| 1370381_at | Pnrc1 | Proline-rich nuclear receptor coactivator 1, F-box coactivator 1, F-box protein 11 | 0.75 | 74.44 |
| 1372197_at | Rictor | RPTOR independent companion of MTOR, complex 2 | 2.25 | 15.11 |
| 1389245_at | Psmd7 | proteasome 26S subunit, non-ATPase 7 | 3.75 | 2.47 |
| 1399022_at | Clk1 | CDC-like kinase 1 | 5.25 | 66.46 |
| 1371455_at | Pmm1 | phosphomannomutase 1 | 7.5 | 1.03 |
| 1372722_at | Dnajb4 | DnaJ heat shock protein family member B4 | 0.75 | 40.12 |
| 1371548_at | Mrps25 | mitochondrial ribosomal protein S25 | 3 | 19.35 |
| 1375185_at | Ipo7 | importin 7 | 3.75 | 65.73 |
| 1375922_at | Cox15 | COX15 cytochrome c oxidase assembly homolog | 2.25 | 10.57 |
| 1368228_at | Zranb2 | Zinc finger, ran-binding domain containing 2 | 6 | 24.56 |
| 1373870_at | Fam98a | Family with sequence similarity 98, member A | 6 | 65.19 |
| 1373718_at | Tubb2a | tubulin, beta 2A class 2a | 4.5 | 94.33 |
| 1374493_at | Bmf | Bcl2 Modifying Factor | 1.5 | 42.77 |
| 1371444_at | Cers2 | ceramide synthase 2 | 12 | 15.61 |
| 1376847_at | Marc1 | mitochondrial amidoxime reducing component 1 | 0 | 39.44 |
| 1375892_at | Elavl1 | ELAV like RNA binding protein 1 | 2.25 | 4.89 |
| 1373738_at | Mphosph6 | M phase phosphoprotein 6 | 5.25 | 3.06 |
| 1367698_a_at | Sept9 | septin 9 | 0.75 | 0.66 |
| 1374747_at | Cdk14 | PFTAIRE protein kinase 1 | 1.5 | 45.24 |
| 1373824_at | Cfdp1 | Flavin | 0.75 | 10.95 |
| 1372908_at | Spsb3 | splA/ryanodine receptor domain and SOCS box containing 3 | 0.75 | 14.78 |
| 1373024_at | Ap3s1 | Adaptor-related protein complex 3, sigma 1 subunit | 5.25 | 36.72 |
| 1390115_at | Sec63 | SEC63 homolog, protein translocation regulator | 0.75 | 1.43 |
| 1398819_at | Dnaja1 | DNAJ (Hsp40) homolog, subfamily A, member 1 | 3 | 6.43 |
| 1370663_at | Wee1 | WEE1 G2 checkpoint kinase | 2.25 | 35.64 |
| 1373158_at | Gpr146 | G protein-coupled receptor 146 | 3 | 52.15 |
| 1371463_at | Phf5a | PHD finger protein 5A | 11.25 | 4.95 |
| 1369973_at | Xdh | xanthine dehydrogenase(Xdh) | 4.5 | 20.94 |
| 1376100_at | Tubb6 | tubulin, beta 6 class V | 1.5 | 85.41 |
| 1372741_at | Sccpdh | saccharopine dehydrogenase | 0.75 | 39.72 |
| 1374625_at | Hes6 | hes family bHLH transcription factor 6 | 5.25 | 26.62 |
| 1388471_at | Tcp11l2 | t-complex 11 like 2 | 0.75 | 57.04 |
| 1388519_at | Sec61b | Sec61 β subunit | 0 | 12.10 |
| 1389832_at | Gsto1 | glutathione S-transferase omega 1 | 10.5 | 0.93 |
| 1398874_at | Atxn10 | Ataxin 10 | 3 | 21.62 |
| 1374945_at | Trmt61a | tRNA methyltransferase 61A | 9 | 35.43 |
| 1389032_at | Mcoln1 | mucolipin 1 | 0.75 | 11.48 |
| 1388401_at | Flnb | filamin-B-like | 3 | 39.34 |
| 1372661_at | Tbl3 | transducin -like 3 | 3.75 | 20.18 |
| 1398763_at | Timm23 | translocase of inner mitochondrial membrane 23 | 1.5 | 11.98 |
| 1387659_at | Gda | Guanine deaminase; EC 3.50.4.3 | 3 | 42.61 |
| 1373014_at | B3gat3 | beta-1,3-glucuronyltransferase 3 | 3.75 | 46.66 |
| 1374947_at | Bcar3 | breast cancer anti-estrogen resistance 3 | 0 | 51.00 |
| 1377192_a_at | Clpx | Caseinolytic peptidase X | 0.75 | 87.30 |
| 1371969_at | Cald1 | Caldesmon 1 | 0.75 | 52.60 |
| 1388789_at | Tmem263 | transmembrane protein 263 | 2.25 | 50.31 |
| 1398799_at | Eif4e | Eukaryotic translation initiation factor 4e | 3 | 59.28 |
| 1368102_at | Hsd11b2 | hydroxysteroid 11-beta dehydrogenase 2 | 8.25 | 13.64 |
| 1374070_at | Gpx2 | glutathione peroxidase 2 | 6 | 33.33 |
| 1373039_at | Tomm70a | translocase of outer mitochondrial membrane 70 | 8.25 | 31.58 |
| 1377051_at | LOC100362572 | Mpv17 transgene, kidney disease mutant-like -like | 0.75 | 19.12 |
| 1368870_at | Id2 | inhibitor of DNA binding 2, HLH protein | 2.25 | 46.95 |
| 1372260_at | Rogdi | rogdi homolog | 6 | 18.55 |
| 1373248_at | Vsig10 | V-set & immunoglobulin domain containing 10 | 1.5 | 33.01 |
| 1389617_at | Elk3 | ELK3, ETS-domain protein | 0.75 | 10.99 |
| 1371986_at | Anp32a | Acidic Nuclear Phosphoprotein 32 Family Member A | 3.75 | 39.19 |
| 1376089_at | Ldlr | low density lipoprotein receptor | 9.75 | 35.62 |
| 1375080_at | Slc25a45 | solute carrier family 25, member 45 | 6 | 19.84 |
| 1398273_at | Efna1 | Ephrin A1 | 0 | 58.49 |
| 1388523_at | Txndc12 | thioredoxin domain containing 12 | 1.5 | 38.29 |
| 1371485_at | Sh3pxd2a | SH3 And PX Domains 2A | 1.5 | 15.47 |
| 1389648_at | Ripk4 | receptor-interacting serine-threonine kinase 4 | 2.25 | 50.27 |
| 1375895_at | Tsr1 | TSR1, ribosome maturation factor | 9 | 70.94 |
| 1388721_at | Hspb8 | heat shock protein family B member 8 | 0.75 | 12.39 |
| 1372562_at | MGC94207 | similar to RIKEN cDNA C030006K11 | 9.75 | 52.06 |
| 1387365_at | Nr1h3 | nuclear receptor subfamily 1, group H, member 3 | 2.25 | 27.81 |
| 1373533_at | RGD1562037 | similar to OTTHUMP00000046255 | 0 | 24.56 |
| 1389538_at | Nfkbia | NFKB inhibitor alpha | 2.25 | 44.43 |
| 1372602_at | Stbd1 | Genethonin 1 | 4.5 | 64.21 |
| 1388976_at | Bola3 | bolA family member 3 | 0 | 58.85 |
| 1372533_at | Edem1 | ER degradation enhancer mannosidase alpha-like 1 | 5.25 | 75.44 |
| 1367894_at | Insig1 | insulin induced gene 1 | 1.5 | 42.38 |
| 1387905_at | Dnajc12 | DnaJ heat shock protein family member C12 | 1.5 | 18.68 |
| 1374601_at | Ifngr2 | interferon gamma receptor 2 | 5.25 | 50.82 |
| 1377474_at | Abhd13 | abhydrolase domain containing 13 | 2.25 | 0.92 |
| 1371957_at | Imp4 | IMP4, U3 small nucleolar ribonucleoprotein | 4.5 | 38.90 |
| 1392900_at | Capza1 | Capping protein muscle z-line, α 1 | 6 | 39.11 |
| 1369630_at | Adk | adenosine kinase | 9 | 27.53 |
| 1379252_at | LOC102556753 | -- | 8.25 | 57.66 |
| 1390392_at | Tmem185b | transmembrane protein 185B | 8.25 | 27.63 |
| 1370353_at | Timm22 | translocase of inner mitochondrial membrane 22 | 7.5 | 39.20 |
| 1372331_at | Eif1ax | eukaryotic translation initiation factor 1A, X-linked | 3 | 54.44 |
| 1388684_at | Fnbp4 | formin binding protein 4 | 3 | 23.92 |
| 1371547_at | Grcc10 | gene rich cluster, C10 gene | 0 | 20.93 |
| 1373625_at | Shmt1 | Serine hydroxymethyl transferase 1 (soluble) | 3.75 | 33.68 |
| 1371945_at | Ube2l3 | ubiquitin-conjugating enzyme E2L 3 | 5.25 | 31.59 |
| 1367795_at | Ifrd1 | interferon-related developmental regulator 1 | 8.25 | 85.06 |
| 1386954_at | Ak2 | Adenylate kinase 2 | 5.25 | 30.17 |
| 1367850_at | Fcgr2a | low affinity immunoglobulin gamma Fc region receptor III-like | 0.75 | 76.52 |
| 1374159_at | Als2cl | ALS2 C-terminal like | 2.25 | 51.34 |
| 1373447_at | Hn1l | hematological and neurological expressed 1-like | 5.25 | 42.44 |
| 1368033_at | Nolc1 | nucleolar and coiled-body phosphoprotein 1 | 3.75 | 46.04 |
| 1371752_at | Rangrf | RAN guanine nucleotide release factor | 3 | 25.55 |
| 1367870_at | Glrx3 | glutaredoxin 3 | 3.75 | 45.96 |
| 1371428_at | Sec61g | Sec61 translocon gamma subunit | 3 | 28.64 |
| 1389654_at | Pls1 | plastin 1 | 9.75 | 10.31 |
| 1368522_at | Timeless | timeless circadian clock | 10.5 | 32.89 |
| 1389014_at | Nampt | nicotinamide phosphoribosyltransferase | 3 | 76.43 |
| 1373967_at | Sub1 | SUB1 homolog, transcriptional regulator | 6.75 | 49.75 |
| 1390728_at | Limd1 | LIM domains containing 1 | 2.25 | 42.56 |
| 1371592_at | Csk | c-src tyrosine kinase | 3.75 | 12.48 |
| 1398960_at | Cct6a | chaperonin containing TCP1 subunit 6A | 3 | 86.03 |
| 1389017_at | Antxr2 | Anthrax toxin receptor 2 | 11.25 | 1.68 |
| 1367461_at | Copb1 | coatomer protein complex, subunit beta 1 | 11.25 | 6.93 |
| 1388639_at | Bcas3 | BCAS3, microtubule associated cell migration factor | 6.75 | 11.02 |
| 1383160_at | Chordc1 | Cysteine and histidine-rich domain (CHORD)-containing, zinc-bp1 | 0.75 | 65.28 |
| 1376168_at | Mmgt1 | membrane magnesium transporter 1 | 6 | 72.40 |
| 1394292_at | Mrpl37 | mitochondrial ribosomal protein L37 | 1.5 | 69.78 |
| 1371535_at | Pmm2 | phosphomannomutase 2 | 6.75 | 27.42 |
| AFFX_Rat_GAPDH_5_at | Gapdh | glyceraldehyde-3-phosphate dehydrogenase | 0.75 | 34.89 |
| 1374487_at | Fam96a | family with sequence similarity 96, member A | 3.75 | 7.44 |
| 1370243_a_at | LOC100359583 | hypothetical protein LOC100359583 | 3.75 | 54.36 |
| 1370370_at | Hyal2 | hyaluronoglucosaminidase 2 | 0 | 16.57 |
| 1388387_at | Ubac1 | UBA domain containing 1 | 1.5 | 26.18 |
| 1387962_at | Zfp180 | zinc finger protein 180 | 4.5 | 12.25 |
| 1372450_at | Cmc2 | C-x-C motif containing 2 | 0.75 | 51.02 |
| 1389733_at | Mars | methionyl-tRNA synthetase | 9.75 | 42.37 |
| 1370319_at | Ppif | peptidylprolyl isomerase F | 5.25 | 9.45 |
| 1371972_at | Sun2 | Sad1 and UNC84 domain containing 2 | 3.75 | 52.13 |
| 1371454_at | Emc6 | ER membrane protein complex subunit 6 | 2.25 | 55.81 |
| 1387912_at | Ddx46 | DEAD-box helicase 46 | 2.25 | 15.87 |
| 1398798_at | Metap2 | similar to Methionine aminopeptidase 2 | 9 | 30.84 |
| 1387053_at | Fmo1 | Flavin-containing monooxygenase 1 | 11.25 | 21.36 |
| 1373302_at | Acer2 | alkaline ceramidase 2 | 0.75 | 25.11 |
| 1390412_at | Slc40a1 | solute carrier family 40 member 1 | 11.25 | 35.03 |
| 1367454_at | Copb2 | coatomer protein complex subunit beta 2 | 10.5 | 18.41 |
| 1386978_at | Bnip3l | BCL2/adenovirus E1B interacting protein 3-like | 0 | 60.08 |
| 1372715_at | Sfxn1 | Liver tricarboxylate carrier, mitochondrial | 2.25 | 55.50 |
| 1371467_at | l7Rn6 | Hikeshi, heat shock protein nuclear import factor | 1.5 | 73.49 |
| 1372001_at | Fam96b | family with sequence similarity 96, member B | 4.5 | 49.96 |
| 1386923_at | Ube2b | ubiquitin-conjugating enzyme E2B | 0.75 | 42.17 |
| 1367724_a_at | Atp6v0e1 | ATPase H+ transporting V0 subunit e1 | 7.5 | 37.67 |
| 1390536_at | Cdon | Cell Adhesion Associated, Oncogene Regulated | 9.75 | 60.00 |
| 1398875_at | Polr3k | RNA polymerase III subunit K | 3.75 | 51.26 |
| 1368428_at | Xpnpep2 | X-prolyl aminopeptidase 2, membrane-bound | 5.25 | 12.21 |
| 1390035_at | Diexf | digestive organ expansion factor homolog | 6 | 52.41 |
| 1374513_at | septin-7-like | septin-7-like | 5.25 | 56.01 |
| 1371738_at | Ano10 | anoctamin 10 | 8.25 | 24.32 |
| 1388537_at | Nipsnap1 | nipsnap homolog 1 | 2.25 | 24.56 |
| 1390893_at | Tmem69 | transmembrane protein 69 | 7.5 | 45.62 |
| 1398896_at | Arcn1 | Archain 1 | 9.75 | 25.90 |
| 1371372_at | Ptges3 | Prostaglandin E synthase 3 | 3 | 62.39 |
| 1374169_at | Cdip1 | cell death-inducing p53 target 1 | 1.5 | 75.00 |
| 1389096_at | Mical2 | microtubule associated monooxygenase, calponin and LIM domain containing 2 | 3 | 49.17 |
| 1388136_at | Timm9 | Translocase of inner mitochondrial membrane 9 | 9 | 38.85 |
| 1367728_at | Tsn | translin | 5.25 | 57.48 |
| 1367820_at | Banf1 | barrier to autointegration factor 1 | 3 | 32.30 |
| 1368091_at | Oplah | 5-oxoprolinase (ATP-hydrolysing) | 8.25 | 59.15 |
| 1374425_at | Tle1 | transducin like enhancer of split 1 | 3 | 57.71 |
| 1390381_at | Xpc | Xeroderma pigmentosum, | 4.5 | 65.33 |
| 1372558_at | Naa15 | N-acetyltransferase 15, NatA auxiliary subunit | 3.75 | 66.11 |
| 1387203_at | Gckr | Glucokinase regulatory protein | 0.75 | 51.94 |
| 1371763_at | RGD1309534 | -- | 9.75 | 63.19 |
| 1369969_at | Parp1 | poly polymerase 1 | 0.75 | 9.42 |
| 1373677_at | Slc39a10 | solute carrier family 39 member 10 | 0.75 | 76.94 |
| 1370575_a_at | Azin1 | antizyme inhibitor 1 | 8.25 | 65.11 |
| 1373108_at | Ppp1r3c | protein phosphatase 1, regulatory subunit 3C | 0.75 | 73.17 |
| 1372413_at | Mcrs1 | microspherule protein 1 | 6 | 39.47 |
| 1372607_at | Nubp2 | nucleotide binding protein 2 | 6.75 | 9.03 |
| 1374876_at | Leprotl1 | leptin receptor overlapping transcript-like 1 | 6.75 | 57.32 |
| 1373372_at | Mydgf | myeloid-derived growth factor | 0.75 | 54.29 |
| 1386976_at | Cd82 | Cd82 molecule | 2.25 | 62.63 |
| 1376501_at | Arhgap8 | Rho GTPase activating protein 8 | 9.75 | 70.72 |
| 1367867_at | Gfer | Augmenter of liver regeneration | 0.75 | 64.40 |
| 1398908_at | Stoml2 | stomatin like 2 | 2.25 | 37.68 |
| 1369644_at | Adgrl2 | adhesion G protein-coupled receptor L2 | 10.5 | 59.88 |
| 1376252_at | Srsf3 | Splicing factor, arginine/serine-rich 3 (SRp20)(Sfrs3) | 12 | 36.08 |
| 1375247_at | Mgll | monoglyceride lipase | 6 | 24.31 |
| 1367849_at | Sdc1 | syndecan 1 | 6 | 2.76 |
| 1367854_at | Acly | ATP citrate lyase | 7.5 | 23.54 |
| 1374987_at | Pank3 | pantothenate kinase 3 | 6 | 45.53 |
| 1373441_at | Dctn5 | dynactin subunit 5 | 9 | 57.42 |
| 1372827_at | Ppid | peptidylprolyl isomerase D | 0.75 | 83.15 |
| 1371634_at | Tmem126a | transmembrane protein 126A | 7.5 | 34.18 |
| 1371820_at | Mesdc2 | Mesoderm Development LRP Chaperone | 7.5 | 63.56 |
| 1371843_at | Yipf5 | Yip1 domain family, member 5 | 10.5 | 48.34 |
| 1370281_at | Fabp5 | fatty acid binding protein 5, epidermal | 5.25 | 34.27 |
| 1367969_at | Prdx6 | peroxiredoxin 6 | 1.5 | 0.62 |
| 1388819_at | Scamp1 | secretory carrier membrane protein 1 | 5.25 | 22.46 |
| 1389006_at | Mpeg1 | macrophage expressed 1 | 4.5 | 35.16 |
| 1389197_at | Poglut1 | protein O-glucosyltransferase 1 | 8.25 | 33.24 |
| 1388469_at | Igf1 | Insulin Like Growth Factor 1 | 1.5 | 4.70 |
| 1368847_at | Rab10 | RAB10, member RAS oncogene family | 5.25 | 10.09 |
| 1372274_at | Kmt2e | lysine methyltransferase 2E | 1.5 | 66.38 |
| 1388650_at | Top2a | topoisomerase (DNA) II alpha | 6.75 | 36.94 |
| 1379604_at | Apol3 | apolipoprotein L, 3 | 2.25 | 45.64 |
| 1373632_at | Ak6 | adenylate kinase 6 | 3 | 43.19 |
| 1376492_at | Unkl | unkempt family like zinc finger | 1.5 | 68.50 |
| 1371480_at | Cks1b | CDC28 protein kinase regulatory subunit 1B | 5.25 | 35.74 |
| 1374161_at | Ipo11 | importin 11 | 4.5 | 48.74 |
| 1374750_at | Fnip1 | Folliculin Interacting Protein 1 | 0 | 55.70 |
| 1372953_at | Ncald | neurocalcin delta | 3 | 60.27 |
| 1388810_at | Abce1 | ATP binding cassette subfamily E member 1 | 6.75 | 20.51 |
| 1377042_at | Pcgf5 | Polycomb group ring finger 5 | 4.5 | 69.41 |
| 1372857_at | Pacsin2 | protein kinase C and casein kinase substrate in neurons 2 | 9.75 | 16.55 |
| 1376660_at | Manea | mannosidase, endo-alpha | 7.5 | 21.29 |
| 1371848_at | Snrpd3 | small nuclear ribonucleoprotein D3 polypeptide | 5.25 | 52.13 |
| 1376765_at | Mro | maestro | 7.5 | 41.84 |
| 1371707_at | Tnpo2 | transportin 2 | 4.5 | 0.28 |
| 1367932_at | Hmgcs1 | 3-hydroxy-3-methylglutaryl-CoA synthase 1 | 11.25 | 41.99 |
| 1372221_at | Ube2h | ubiquitin-conjugating enzyme E2H | 2.25 | 43.44 |
| 1398945_at | Ddx6 | DEAD-box helicase 6 | 1.5 | 66.03 |
| 1369962_at | Atic | bifunctional purine biosynthesis protein PURH-like | 11.25 | 50.11 |
| 1389612_at | Rcbtb2 | RCC1 and BTB domain containing protein 2 | 2.25 | 27.41 |
| 1389734_x_at | RT1-T24-4 | RT1 class I, locus T24, gene 4 | 10.5 | 58.70 |
| 1367826_at | Nfe2l2 | nuclear factor, erythroid 2-like 2 | 11.25 | 31.64 |
| 1370803_at | Zwint | ZW10 interacting kinetochore protein | 4.5 | 40.81 |
| 1375658_at | Sash1 | SAM and SH3 domain containing 1 | 1.5 | 48.86 |
| 1392484_at | LOC100233176 | GON7, KEOPS complex subunit homolog | 10.5 | 40.41 |
| 1376570_at | Cct5 | chaperonin containing TCP1 subunit 5 | 0.75 | 71.99 |
| 1370314_at | Slc20a1 | solute carrier family 20 member 1 | 0 | 72.15 |
| 1371089_at | Gsta3 | glutathione S-transferase alpha 3 | 9 | 28.55 |
| 1388790_at | Coq5 | coenzyme Q5, methyltransferase | 1.5 | 26.11 |
| 1370975_at | Kdm3a | lysine demethylase 3A | 1.5 | 28.23 |
| 1367767_at | Hmgcl | 3-hydroxymethyl-3-methylglutaryl-CoA lyase | 6 | 11.70 |
| 1388454_at | Oaz2 | ornithine decarboxylase antizyme 2 | 3 | 43.35 |
| 1388720_at | Rnf144b | ring finger protein 144B | 10.5 | 61.97 |
| 1372531_at | Ppfibp2 | PPFIA binding protein 2 | 1.5 | 0.38 |
| 1375887_at | LOC100910807 | -- | 3 | 14.59 |
| 1387336_at | Nat8 | N-Acetyltransferase Camello 4 | 3.75 | 38.69 |
| 1374897_at | Alyref | Aly/REF export factor | 5.25 | 45.93 |
| 1370940_at | Tjp2 | Tight junction protein 2 (zona occludens 2) | 1.5 | 54.71 |
| 1367761_at | Ndel1 | nudE neurodevelopment protein 1-like 1 | 10.5 | 23.13 |
| 1373479_at | Ppp3ca | protein phosphatase 3 catalytic subunit alpha | 8.25 | 55.32 |
| 1376437_at | Derl2 | derlin 2 | 10.5 | 16.40 |
| 1367641_at | Sod1 | superoxide dismutase 1, soluble | 0 | 32.77 |
| 1386885_at | Ech1 | enoyl-CoA hydratase 1 | 9.75 | 6.58 |
| 1372218_at | Wdr12 | WD repeat domain 12 | 5.25 | 21.33 |
| 1380547_at | Clcn3 | chloride voltage-gated channel 3 | 6 | 62.39 |
| 1375453_at | Gpatch8 | G patch domain containing 8 | 1.5 | 29.77 |
| 1388484_at | Ube2c | Ubiquitin-conjugating enzyme E2C | 6 | 47.32 |
| 1389200_at | Bysl | bystin-like | 4.5 | 29.97 |
| 1372769_at | Eif2d | eukaryotic translation initiation factor 2D | 0.75 | 23.17 |
| 1371656_at | Cct4 | chaperonin containing TCP1 subunit 4p | 3 | 60.22 |
| 1371403_at | Cct3 | Chaperonin subunit 3 (gamma) | 2.25 | 78.09 |
| 1370184_at | Cfl1 | cofilin 1 | 2.25 | 16.50 |
| 1390326_at | Ang | angiogenin | 8.25 | 64.20 |
| 1398295_at | Slc29a1 | solute carrier family 29 member 1 | 3 | 8.68 |
| 1390028_at | Dyrk2 | Dual-specificity tyrosine- (Y)-phosphorylation regulated kinase 2 | 1.5 | 23.60 |
| 1373152_at | Prss23 | protease, serine, 23 | 4.5 | 0.34 |
| 1390421_at | Hoga1 | 4-hydroxy-2-oxoglutarate aldolase 1 | 2.25 | 24.68 |
| 1370215_at | C1qb | complement C1q B chain | 2.25 | 48.56 |
| 1388565_at | Spg21 | spastic paraplegia 21 homolog | 3.75 | 16.40 |
| 1386894_at | Hspd1 | heat shock protein family D member 1 | 1.5 | 70.76 |
| 1371422_at | Morf4l2 | mortality factor 4 like 2 | 3.75 | 38.52 |
| 1371352_at | Hmgn2 | high mobility group nucleosomal binding domain 2 | 9.75 | 52.05 |
| 1370163_at | Odc1 | Ornithine decarboxylase 1 | 9.75 | 71.89 |
| 1370237_at | Hadh | hydroxyacyl-CoA dehydrogenase | 1.5 | 22.18 |
| 1367834_at | Srm | spermidine synthase | 3 | 68.98 |
| 1376222_at | Sdr39u1 | short chain dehydrogenase/reductase family 39U, member 1 | 1.5 | 86.65 |
| 1367718_at | Chkb | Choline kinase-like | 2.25 | 54.12 |
| 1375432_at | Exosc1 | exosome component 1 | 5.25 | 12.79 |
| 1387805_at | Bnip3 | BCL2/adenovirus E1B 19 kDa- | 0.75 | 29.48 |
| 1371853_at | LOC103691922 /// Mrpl42 | mitochondrial ribosomal protein L42 | 1.5 | 63.60 |
| 1377155_at | LOC102549803 /// LOC102556148 | probable N-acetyltransferase CML2-like | 3.75 | 27.55 |
| 1386898_at | Hspe1 | heat shock protein family E member 1 | 0 | 73.58 |
| 1389046_at | Cnnm4 | cyclin and CBS domain divalent metal cation transport mediator 4 | 6 | 71.06 |
| 1387024_at | Dusp6 | dual specificity phosphatase 6 | 5.25 | 12.79 |
| 1367465_at | Dad1 | defender against cell death 1 | 3.75 | 2.39 |
| 1371581_at | Srsf1 | serine and arginine rich splicing factor 1 | 8.25 | 48.37 |
| 1371373_at | Rnf181 | ring finger protein 181 | 5.25 | 65.03 |
| 1374176_at | Lurap1l | Leucine Rich Adaptor Protein 1 Like | 3.75 | 61.59 |
| 1367686_at | Serp1 | Ribosome-associated membrane protein 4 | 0.75 | 55.39 |
| 1372517_at | Ppil1 | peptidylprolyl isomerase like 1 | 4.5 | 17.74 |
| 1376737_at | LOC100912041 | uncharacterized LOC100912041 | 11.25 | 15.65 |
| 1388960_at | Sar1a | secretion associated, Ras related GTPase 1A | 0 | 51.27 |
| 1372588_at | Bles03 | basophilic leukemia expressed protein BLES03 | 6 | 45.64 |
| 1371076_at | Cyp2b1 | cytochrome P450, family 2, subfamily b, polypeptide 1 | 4.5 | 78.38 |
| 1371652_at | Ccdc50 | coiled-coil domain containing 50 | 0.75 | 69.55 |
| 1379853_at | Hspb11 | heat shock protein family B , member 11 | 8.25 | 18.79 |
| 1374753_at | Papd4 | PAP-associated domain containing 4 | 3.75 | 56.87 |
| 1368536_at | Enpp2 | ectonucleotide pyrophosphatase/phosphodiesterase 2 | 8.25 | 82.85 |
| 1389941_at | Arl2bp | ADP-ribosylation factor like GTPase 2 binding protein | 8.25 | 42.25 |
| 1388103_at | Tmem37 | transmembrane protein 37 | 9.75 | 70.11 |
| 1387063_at | Ip6k2 | inositol hexakisphosphate kinase 2 | 6.75 | 5.07 |
| 1370530_a_at | Pld1 | phospholipase D1 | 3 | 23.73 |
| 1371549_at | Dcaf11 | DDB1 and CUL4 associated factor 11 | 0 | 50.39 |
| 1371332_at | LOC684681 | -- | 1.5 | 57.77 |
| 1374067_at | Cacul1 | CDK2-associated, cullin domain 1 | 0.75 | 34.30 |
| 1387045_at | Atp6v0a1 | ATPase H+ transporting V0 subunit a1 | 2.25 | 7.12 |
| 1375374_at | LOC102555400 | -- | 2.25 | 67.56 |
| 1388820_at | Sltm | SAFB-like, transcription modulator | 0.75 | 52.38 |
| 1367946_at | Pdlim1 | PDZ and LIM domain 1 | 1.5 | 2.85 |
| 1374693_at | Parp16 | poly polymerase family, member 16 | 2.25 | 4.56 |
| 1388341_at | Rangap1 | RAN GTPase activating protein 1 | 0.75 | 3.24 |
| 1390455_at | Abhd2 | abhydrolase domain containing 2 | 12 | 65.19 |
| 1372302_at | Rmdn3 | regulator of microtubule dynamics protein 3-like | 6 | 61.15 |
| 1388450_at | Synrg | Synergin Gamma | 7.5 | 6.37 |
| 1383126_at | Akt1 | AKT Serine/Threonine Kinase 1 | 11.25 | 27.50 |
| 1374932_at | Angptl8 | angiopoietin-like 8 | 0 | 27.88 |
| 1372601_at | Atf5 | activating transcription factor 5 | 2.25 | 29.88 |
| 1368608_at | Cyp2f4 | cytochrome P450, family 2, subfamily f, polypeptide 4 | 12 | 68.41 |
| 1376807_at | Rabl3 | RAB, member of RAS oncogene family-like 3 | 4.5 | 22.87 |
| 1389681_at | Pvrl2 | Poliovirus receptor-related 2 | 6 | 64.85 |
| 1372568_at | Epm2aip1 | EPM2A interacting protein 1 | 2.25 | 42.63 |
| 1374943_at | Fam210b | family with sequence similarity 210, member B | 7.5 | 35.97 |
| 1374441_at | Spryd4 | SPRY domain containing 4 | 10.5 | 27.73 |
| 1370298_at | Coa3 | cytochrome C oxidase assembly factor 3 | 2.25 | 44.77 |
| 1371074_a_at | Mcm6 | minichromosome maintenance complex component 6 | 7.5 | 85.76 |
| 1373387_at | Slirp | SRA stem-loop interacting RNA binding protein | 3 | 55.98 |
| 1375186_at | Dph3 | diphthamide biosynthesis 3 | 6.75 | 30.89 |
| 1372521_at | Rnd2 | Rho family GTPase 2 | 8.25 | 21.69 |
| 1388695_at | Shmt2 | serine hydroxymethyltransferase 2 | 2.25 | 38.99 |
| 1370200_at | Glud1 | glutamate dehydrogenase 1 | 3 | 58.37 |
| 1388164_at | RT1-S3 | RT1 class Ib, locus S3 | 5.25 | 0.30 |
| 1373071_at | Lin37 | lin-37 DREAM MuvB core complex component | 0.75 | 49.26 |
| 1369690_at | Nsf | N-ethylmaleimide sensitive factor, vesicle fusing ATPase | 3 | 24.36 |
| 1369150_at | Pdk4 | pyruvate dehydrogenase kinase 4 | 10.5 | 25.11 |
| 1371839_at | Srsf2 | serine and arginine rich splicing factor 2 | 10.5 | 8.29 |
| 1371855_at | Camk2g | Calcium/Calmodulin Dependent Protein Kinase II Gamma | 1.5 | 24.16 |
| 1369590_a_at | Ddit3 | DNA-damage-inducible transcript 3 | 3.75 | 0.75 |
| 1373309_at | Tmem86a | transmembrane protein 86A | 1.5 | 73.67 |
| 1388321_at | Imp3 | IMP3, U3 small nucleolar ribonucleoprotein | 11.25 | 36.47 |
| 1370290_at | Tubb5 | tubulin, beta 5 class I | 0 | 69.50 |
| 1390027_at | Usp8 | ubiquitin specific peptidase 8 | 4.5 | 49.43 |
| 1367725_at | Pim3 | Pim-3 proto-oncogene, serine/threonine kinase | 2.25 | 63.60 |
| 1377166_at | Als2 | ALS2, Alsin Rho Guanine Nucleotide Exchange Factor | 2.25 | 49.80 |
| 1370909_at | Nup62 | nucleoporin 62 | 1.5 | 66.41 |
| 1368378_at | Aldh1l1 | aldehyde dehydrogenase 1 family, member L1 | 3.75 | 8.53 |
| 1372868_at | Tor3a | torsin family 3, member A | 9.75 | 75.91 |
| 1382028_at | Pex19 | peroxisomal biogenesis factor 19 | 0.75 | 44.09 |
| 1370001_at | Rab8a | RAB8A, member RAS oncogene family | 9 | 25.35 |
| 1375161_at | Mrpl55 | mitochondrial ribosomal protein L55 | 2.25 | 62.91 |
| 1372116_at | Mrps2 | mitochondrial ribosomal protein S2 | 4.5 | 11.72 |
| 1377300_at | Dusp3 | dual specificity phosphatase 3 -like | 3.75 | 38.11 |
| 1372739_at | Tspan31 | tetraspanin 31 | 6 | 79.94 |
| 1384240_at | Agtr1a | angiotensin II receptor, type 1a | 3 | 19.71 |
| 1373867_at | Kdm5a | lysine demethylase 5A | 1.5 | 37.73 |
| 1367579_a_at | Tuba1c | tubulin alpha-1C chain-like | 0 | 58.83 |
| 1371543_at | Mtmr2 | myotubularin related protein 2 | 10.5 | 23.67 |
| 1368051_at | Hsd17b12 | hydroxysteroid (17-beta) dehydrogenase 12 | 1.5 | 65.97 |
| 1371565_at | Kti12 | KTI12 chromatin associated homolog | 8.25 | 54.77 |
| 1375974_at | G3bp2 | G3BP stress granule assembly factor 2 | 3 | 31.09 |
| 1399159_a_at | Vamp3 | vesicle-associated membrane protein 3 | 1.5 | 75.40 |
| 1389088_at | Adnp | activity-dependent neuroprotector homeobox | 1.5 | 36.51 |
| 1376435_at | Loxl4 | Lysyl Oxidase Like 4 | 0.75 | 30.11 |
| 1389391_at | Slc35e3 | solute carrier family 35, member E3 | 9.75 | 58.18 |
| 1399034_at | Pcnx | pecanex homolog 1 | 5.25 | 63.77 |
| 1367463_at | Phb2 | prohibitin 2 | 5.25 | 55.18 |
| 1367839_at | Fdft1 | farnesyl diphosphate farnesyl transferase 1 | 10.5 | 7.83 |
| 1374318_at | Brcc3 | BRCA1/BRCA2-containing complex, subunit 3 | 7.5 | 63.61 |
| 1373274_at | Txnrd3 | thioredoxin reductase 3 | 0.75 | 57.91 |
| 1377102_at | Tmem63a | transmembrane protein 63a | 2.25 | 33.62 |
| 1374396_at | Atp6v1c1 | ATPase H+ transporting V1 subunit C1 | 10.5 | 28.57 |
| 1389639_at | Shb | Protocadherin 1 (cadherin-like 1) | 7.5 | 53.91 |
| 1372720_at | Btbd1 | BTB domain containing 1 | 6 | 66.43 |
| 1374303_at | Alkbh2 | alkB homolog 2, alpha-ketoglutarate-dependent dioxygenase | 4.5 | 67.94 |
| 1367583_at | Tpt1 | tumor protein, translationally-controlled 1 | 3.75 | 32.43 |
| 1367711_at | Psmc2 | proteasome 26S subunit, ATPase 2 | 8.25 | 22.43 |
| 1373984_at | Slc39a14 | solute carrier family 39 member 14 | 5.25 | 25.19 |
| 1389969_at | Tomm40 | Translocase Of Outer Mitochondrial Membrane 40 | 6 | 45.15 |
| 1387773_at | Cycs | Cytochrome C, Somatic | 1.5 | 49.08 |
| 1369679_a_at | Nfia | nuclear factor I/A | 4.5 | 73.82 |
| 1368158_at | Scfd1 | sec1 family domain containing 1 | 9 | 45.76 |
| 1388370_at | Ccni | cyclin I | 4.5 | 51.26 |
| 1375451_at | Pcnxl3 | pecanex homolog 3 | 2.25 | 33.60 |
| 1372709_at | Bcap29 | B-cell receptor-associated protein 29 | 1.5 | 23.17 |
| 1398912_at | mrpl9 | mitochondrial ribosomal protein L9 | 6.75 | 47.24 |
| 1390387_at | Sh3d19 | SH3 domain containing 19 | 9.75 | 61.98 |
| 1367838_at | Cth | CTL target antigen (Cth) | 3.75 | 72.08 |
| 1370304_at | Timm17a | Translocase of inner mitochondrial membrane 17a | 7.5 | 19.62 |
| 1373006_at | Tmem171 | transmembrane protein 171 | 0.75 | 10.18 |
| 1389493_at | Abtb1 | ankyrin repeat and BTB domain containing 1 | 5.25 | 25.77 |
| 1375941_at | Baiap2l1 | BAI1-associated protein 2-like 1 | 7.5 | 91.43 |
| 1370193_at | Ptp4a1 | protein tyrosine phosphatase 4a1-like | 1.5 | 75.48 |
| 1391510_at | Ebag9 | estrogen receptor binding site associated, antigen, 9 | 12 | 41.45 |
| 1389420_at | Stap2 | Signal-transducing adaptor protein 2 | 9.75 | 17.02 |
| 1373685_at | Ankrd37 | ankyrin repeat domain 37 | 0 | 80.69 |
| 1373868_at | Bclaf1 | BCL2-associated transcription factor 1 | 3 | 45.11 |
| 1371410_at | Wdr83os | WD repeat domain 83 opposite strand | 3.75 | 9.65 |
| 1373955_at | Ipo5 | importin 5 | 3.75 | 44.89 |
| 1376849_at | Usp48 | Ubiquitin-specific protease 48 | 4.5 | 74.32 |
| 1398930_at | Atp6v0b | ATPase H+ transporting V0 subunit B | 7.5 | 61.57 |
| 1388150_at | Xpo1 | exportin 1 | 11.25 | 47.26 |
| 1371754_at | Slc25a25 | solute carrier family 25 member 25 | 9 | 60.18 |
| 1372830_at | Smg1 | SMG1, Nonsense Mediated MRNA Decay Associated PI3K Related Kinase | 5.25 | 30.43 |
| 1372340_at | Mat2b | methionine adenosyltransferase 2B | 4.5 | 43.76 |
| 1388674_at | Cdkn1a | cyclin-dependent kinase inhibitor 1A | 0.75 | 73.63 |
| 1377821_at | Erbb3 | erb-b2 receptor tyrosine kinase 3 | 0.75 | 72.12 |
| 1374652_at | Lrig1 | leucine-rich repeats and immunoglobulin-like domains 1 | 8.25 | 57.08 |
| 1387244_at | Cgrrf1 | Cgr19 | 0 | 63.16 |
| 1382048_at | Atp6v1a | Myosin ID | 6.75 | 42.13 |
| 1389013_at | Rpl7l1 | ribosomal protein L7-like 1 | 8.25 | 53.46 |
| 1367856_at | G6pd | glucose-6-phosphate dehydrogenase | 2.25 | 52.59 |
| 1374421_at | Baz1b | bromodomain adjacent to zinc finger domain, 1B | 2.25 | 59.59 |
| 1390124_at | RGD1560464 | similar to hypothetical protein FLJ38426 | 8.25 | 17.21 |
| 1371745_at | Cwc15 | CWC15 spliceosome-associated protein | 3.75 | 40.27 |
| 1390497_at | Txlng | taxilin gamma | 0.75 | 48.80 |
| 1368671_at | Srpx | sushi-repeat-containing protein, X-linked | 2.25 | 48.06 |
| 1387221_at | Gch1 | GTP cyclohydrolase 1 | 11.25 | 65.19 |
| 1390577_at | Ranbp10 | RAN binding protein 10 | 11.25 | 56.91 |
| 1371967_at | Mrpl16 | mitochondrial ribosomal protein L16 | 5.25 | 38.17 |
| 1389430_at | Hsd17b7 | Hydroxysteroid (17-beta) dehydrogenase 7 | 8.25 | 68.42 |
| 1389170_at | Casp7 | caspase 7 | 8.25 | 19.26 |
| 1369937_at | Calm1 | calmodulin 1 | 2.25 | 26.49 |
| 1372156_at | Tmem97 | transmembrane protein 97 | 6 | 58.02 |
| 1374449_at | Cdca3 | cell division cycle associated 3 | 6.75 | 43.81 |
| 1371660_at | Znhit1 | zinc finger, HIT-type containing 1 | 0.75 | 13.09 |
| 1388163_at | Slc25a5 | ADP/ATP translocase 3-like | 1.5 | 9.37 |
| 1370348_at | Ninj1 | ninjurin 1 | 0.75 | 17.41 |
| 1374914_at | Ppard | peroxisome proliferator-activated receptor delta | 1.5 | 79.38 |
| 1372170_at | Acy1 | aminoacylase 1 | 9 | 77.61 |
| 1373815_at | Lman2 | lectin, mannose-binding 2 | 1.5 | 32.42 |
| 1372108_at | Ptcd3 | Pentatricopeptide repeat domain 3 | 4.5 | 40.46 |
| 1388528_at | Fbl | Fibrillarin | 6.75 | 67.95 |
| 1370426_a_at | Atp2a2 | ATPase sarcoplasmic/endoplasmic reticulum Ca2+ transporting 2 | 3 | 53.33 |
| 1367521_at | Edc3 | enhancer of mRNA decapping 3 | 0 | 74.81 |
| 1371971_at | Txnl4a | thioredoxin like 4A | 0 | 47.85 |
| 1376272_s_at | Tmem167a | transmembrane protein 167A | 2.25 | 25.00 |
| 1372526_at | Flcn | folliculin | 1.5 | 45.85 |
| 1373557_at | Mcm4 | minichromosome maintenance complex component 4 | 8.25 | 57.51 |
| 1374235_at | Rcan2 | regulator of calcineurin 2 | 5.25 | 22.98 |
| 1367484_at | Ube2e1 | ubiquitin-conjugating enzyme E2E 1 | 7.5 | 0.25 |
| 1372536_at | Adck3 | Chaperone, ABC1 activity of bc1 complex like | 11.25 | 33.01 |
| 1374677_at | Adssl1 | adenylosuccinate synthase like 1 | 4.5 | 72.06 |
| 1367775_at | Amacr | alpha-methylacyl-CoA racemase | 4.5 | 27.84 |
| 1372028_at | Nop16 | NOP16 nucleolar protein | 1.5 | 63.36 |
| 1368692_a_at | Chka | choline kinase alpha | 1.5 | 90.58 |
| 1388741_at | Cmya5 | cardiomyopathy associated 5 | 6 | 81.76 |
| 1376627_at | C1galt1 | core 1 synthase, glycoprotein-N-acetylgalactosamine 3-beta-galactosyltransferase, 1 | 12 | 21.00 |
| 1373026_at | Spc24 | SPC24, NDC80 kinetochore complex component | 6 | 77.81 |
| 1371888_at | mrpl24 | mitochondrial ribosomal protein L24 | 3.75 | 21.05 |
| 1371987_at | Papd7 | poly RNA polymerase D7, non-canonical | 0.75 | 73.02 |
| 1373079_at | Smim14 | Small Integral Membrane Protein 14 | 11.25 | 69.92 |
| 1370244_at | Ctsl | cathepsin L | 9.75 | 0.00 |
| 1374486_at | Arhgef11 | Rho Guanine Nucleotide Exchange Factor 11 | 7.5 | 20.45 |
| 1398844_at | Txn2 | thioredoxin 2 | 3.75 | 10.22 |
| 1367536_at | Apip | APAF1 interacting protein | 9 | 50.94 |
| 1398883_at | Hnrnpa2b1 | heterogeneous nuclear ribonucleoprotein A2/B1 | 0.75 | 70.34 |
| 1376140_at | Abcd1 | ATP binding cassette subfamily D member 1 | 1.5 | 4.11 |
| 1370098_at | Vamp7 | vesicle-associated membrane protein 7 | 10.5 | 5.26 |
| 1367833_at | Psmc5 | proteasome 26S subunit, ATPase 5 | 3 | 38.78 |
| 1373270_at | Wipi1 | WD repeat domain, phosphoinositide interacting 1 | 7.5 | 9.51 |
| 1389297_at | Ero1a | endoplasmic reticulum oxidoreductase 1 alpha | 8.25 | 0.49 |
| 1371327_a_at | Actg1 | actin, gamma 1 | 3 | 78.65 |
| 1388681_at | Sar1b | secretion associated, Ras related GTPase 1B | 7.5 | 45.18 |
| 1383618_at | Mrpl38 | mitochondrial ribosomal protein L38 | 1.5 | 7.45 |
| 1371709_at | Mrpl3 | mitochondrial ribosomal protein L3 | 7.5 | 23.81 |
| 1376187_at | Slc35d1 | Solute Carrier Family 35 Member D1 | 3.75 | 43.98 |
| 1372523_at | Gclc | glutamate-cysteine ligase, catalytic subunit | 1.5 | 69.36 |
| 1373515_at | Lgalsl | Galectin-5 (RL-18) | 4.5 | 91.30 |
| 1367707_at | Fasn | fatty acid synthase | 3.75 | 9.84 |
| 1389113_at | Slc45a3 | solute carrier family 45, member 3 | 1.5 | 53.71 |
| 1372140_at | Ccdc28a | coiled-coil domain containing 28A | 2.25 | 68.84 |
| 1388534_at | Slc31a1 | solute carrier family 34 member 1 | 12 | 46.07 |
| 1374676_at | Sgms1 | Sphingomyelin Synthase 1 | 3 | 83.45 |
| 1374387_at | Arl6ip5 | ADP-ribosylation factor like GTPase 6 interacting protein 5 | 5.25 | 66.01 |
| 1373746_at | Wdr91 | WD repeat domain 91 | 3.75 | 46.18 |
| 1388742_at | Bcl2l11 | BCL2 like 11 | 0.75 | 58.90 |
| 1374420_at | Apmap | adipocyte plasma membrane associated protein | 8.25 | 56.94 |
| 1373983_at | Rsrc2 | arginine and serine rich coiled-coil 2 | 1.5 | 27.63 |
| 1373383_at | Mterf3 | MTERF domain containing 1 | 0.75 | 65.32 |
| 1373433_at | Hmgn5 /// Hmgn5b | high mobility group nucleosome binding domain 5 | 3.75 | 74.84 |
| 1371819_at | Hdac5 | histone deacetylase 5 | 3 | 8.22 |
| 1372506_at | Psme3 | glucose-6-phosphatase, catalytic subunit | 2.25 | 57.10 |
| 1375852_at | Hmgcr | 3-Hydroxy-3-methylglutaryl- coenzyme A reductase | 8.25 | 93.79 |
| 1367604_at | Crip2 | cysteine-rich protein 2 | 2.25 | 4.77 |
| 1388487_at | Add1 | adducin 1 | 12 | 6.94 |
| 1373452_at | Rcl1 | RNA terminal phosphate cyclase-like 1 | 5.25 | 20.60 |
| 1386956_at | Scarb1 | scavenger receptor class B, member 1 | 3 | 62.77 |
| 1399109_at | Nhlrc2 | NHL repeat containing 2 | 0.75 | 53.11 |
| 1374036_at | Mcm2 | Minichromosome maintenance deficient 2 mitotin | 5.25 | 89.93 |
| 1377021_at | Trmt6 | tRNA methyltransferase 6 | 6 | 66.76 |
| 1377334_at | RT1-Ba | RT1 class II, locus Ba | 2.25 | 83.00 |
| 1387267_at | Ntf3 | neurotrophin 3 | 1.5 | 17.66 |
| 1387312_a_at | Gck | glucokinase | 3 | 77.33 |
| 1367813_at | Ppp1r14a | protein phosphatase 1, regulatory subunit 14A | 6.75 | 64.16 |
| 1373835_at | Fbxo8 | F-box protein 8 | 2.25 | 64.14 |
| 1367789_at | Slc27a1 | solute carrier family 27 member 1 | 6.75 | 38.98 |
| 1367603_at | Tpi1 | triosephosphate isomerase 1 | 0 | 60.49 |
| 1371329_at | Eif5a | eukaryotic translation initiation factor 5A | 0 | 11.50 |
| 1376347_at | Kdm5b | lysine demethylase 5B | 7.5 | 79.90 |
| 1372966_at | Mfsd2a | major facilitator superfamily domain containing 2A | 2.25 | 64.97 |
| 1391572_at | Cars | cysteinyl-tRNA synthetase | 5.25 | 10.82 |
| 1374953_at | Tstd3 | thiosulfate sulfurtransferase (rhodanese)-like domain containing 3 | 4.5 | 63.16 |
| 1398902_at | Cluh | MKIAA0664 protein | 3 | 20.41 |
| 1375673_at | LOC100912399 | mitogen-activated protein kinase kinase kinase 1-like | 3 | 38.20 |
| 1376376_at | Zbtb44 | Zinc Finger And BTB Domain Containing 44 | 1.5 | 65.91 |
| 1372437_at | Skp1 | S-phase kinase-associated protein 1 | 0.75 | 51.77 |
| 1369950_at | Cdk4 | cyclin-dependent kinase 4 | 11.25 | 29.38 |
| 1388406_at | Naa60 | N-acetyltransferase 60, NatF catalytic subunit | 0 | 29.29 |
| 1373249_at | Ubl4a | ubiquitin-like 4A | 3.75 | 59.65 |
| 1398333_at | Epas1 | endothelial PAS domain protein 1 | 4.5 | 33.12 |
| 1373015_at | Rnf11 | Ring finger protein 11 | 0.75 | 80.20 |
| 1369200_at | Nt5e | 5' nucleotidase, ecto | 0.75 | 59.65 |
| 1388492_at | Tnip1 | TNFAIP3 interacting protein 1 | 0.75 | 13.04 |
| 1371399_at | Tomm6 | translocase of outer mitochondrial membrane 6 | 3 | 9.30 |
| 1372431_at | Mrpl12 | mitochondrial ribosomal protein L12 | 1.5 | 64.99 |
| 1368036_at | Ptprf | Protein Tyrosine Phosphatase, Receptor Type F | 0 | 59.49 |
| 1374854_at | Pinx1 | PIN2/TERF1 interacting, telomerase inhibitor 1 | 3 | 35.13 |
| 1375425_at | Ift20 | intraflagellar transport 20 | 4.5 | 56.99 |
| 1374612_at | Papd5 | poly RNA polymerase D5, non-canonical | 1.5 | 56.05 |
| 1398891_at | Mrpl15 | Mitochondrial ribosomal protein L15 | 0 | 33.93 |
| 1374890_at | Evi5 | ecotropic viral integration site 5 | 3 | 74.57 |
| 1370172_at | Sod2 | superoxide dismutase 2 | 0 | 1.45 |
| 1388716_at | Ehmt2 | euchromatic histone lysine methyltransferase 2 | 7.5 | 18.26 |
| 1371976_at | Fam195a | MAPK Regulated Corepressor Interacting Protein 2 | 1.5 | 74.78 |
| 1389156_at | Kxd1 | Hypothetical protein LOC498606 | 2.25 | 38.10 |
| 1370313_at | Acot7 | acyl-CoA thioesterase 7 | 1.5 | 31.53 |
| 1374109_at | Dgkq | diacylglycerol kinase, theta | 0.75 | 18.90 |
| 1371764_at | Ube2j2 | ubiquitin-conjugating enzyme E2, J2 | 9 | 17.20 |
| 1372187_at | Prkd3 | protein kinase D3 | 1.5 | 17.36 |
| 1388642_at | Ei24 | Progesterone receptor membrane component 2 | 1.5 | 81.97 |
| 1371955_at | Mrpl35 | mitochondrial ribosomal protein L35 | 3 | 47.65 |
| 1372779_at | B3gnt2 | UDP-GlcNAc:betaGal beta-1,3-N-acetylglucosaminyltransferase 2 | 3.75 | 2.53 |
| 1376936_at | Slc8b1 | solute carrier family 8 member B1 | 3.75 | 50.86 |
| 1388414_at | LOC100363268 /// Ndufs5 | rCG31129-like | 0.75 | 23.08 |
| 1391078_at | Rfc1 | replication factor C subunit 1 | 5.25 | 78.42 |
| 1374333_at | Card19 | caspase recruitment domain family, member 19 | 4.5 | 76.23 |
| 1371939_at | Caprin1 | cell cycle associated protein 1 | 7.5 | 5.16 |
| 1387914_at | Cyp27a1 | cytochrome P450, family 27, subfamily a, polypeptide 1 | 11.25 | 19.62 |
| 1371378_at | Eif1 | eukaryotic translation initiation factor 1 | 5.25 | 38.93 |
| 1387867_at | Aldh9a1 | aldehyde dehydrogenase 9 family, member A1 | 3.75 | 46.50 |
| 1370375_at | Gls2 | glutaminase 2 | 3 | 20.97 |
| 1372334_at | Opa1 | OPA1, Mitochondrial Dynamin Like GTPase | 6 | 25.73 |
| 1368654_at | Npap60 | nucleoporin 50 | 3 | 39.39 |
| 1368275_at | Msmo1 | Sterol-C4-methyl oxidase-like | 11.25 | 76.41 |
| 1388882_at | Fkbp3 | FK506 binding protein 3 | 1.5 | 57.14 |
| 1372036_at | Cd2bp2 | Cd2 binding protein 2 | 9 | 48.66 |
| 1374848_at | Gtf3c6 | general transcription factor 3C subunit 6 | 2.25 | 29.59 |
| 1374644_at | Gpr180 | G Protein-Coupled Receptor 180 | 6.75 | 60.56 |
| 1399077_at | Mtx1 | Metaxin 1 | 3.75 | 32.92 |
| 1372871_at | Cnppd1 | cyclin Pas1/PHO80 domain containing 1 | 0.75 | 69.09 |
| 1390343_at | Ccnc | cyclin C | 8.25 | 67.93 |
| 1388752_at | Bclaf1 | BCL2-associated transcription factor 1 | 2.25 | 62.20 |
| 1389000_at | Kdm3b | lysine demethylase 3B | 0.75 | 39.56 |
| 1390101_at | Ccdc107 | coiled-coil domain containing 107 | 3.75 | 14.39 |
| 1374642_at | Zfp64 | zinc finger protein 64 | 2.25 | 52.05 |
| 1398832_at | Ncl | Nucleolin | 11.25 | 58.64 |
| 1371418_at | Cct2 | Chaperonin containing TCP1, subunit 2 (beta) | 8.25 | 74.70 |
| 1398938_at | Acp1 /// LOC102549052 | acid phosphatase 1, soluble | 6.75 | 10.44 |
| 1387185_at | Apbb3 | amyloid beta precursor protein binding family B member 3 | 9.75 | 42.70 |
| 1369669_at | Nln | neurolysin | 10.5 | 51.34 |
| 1367807_at | Plod1 | procollagen-lysine, 2-oxoglutarate 5-dioxygenase 1 | 1.5 | 41.29 |
| 1388682_at | Cnih1 | cornichon family AMPA receptor auxiliary protein 1 | 3.75 | 54.48 |
| 1398348_at | Ddah1 | Dimethylarginine Dimethylaminohydrolase 1 | 3 | 52.51 |
| 1367790_at | Snd1 | staphylococcal nuclease and tudor domain containing 1 | 6 | 45.79 |
| 1389229_at | Pxylp1 | 2-phosphoxylose phosphatase 1 | 6 | 57.78 |
| 1399031_at | Dda1 | similar to CG31855-PA | 6.75 | 47.65 |
| 1374006_at | Ccbl2 | kynurenine aminotransferase 3 | 0.75 | 46.11 |
| 1371768_at | Ssu72 | SSU72 homolog, RNA polymerase II CTD phosphatase | 0.75 | 47.85 |
| 1374010_at | Lig3 | DNA ligase 3 | 3 | 65.85 |
| 1398300_at | Atp1b3 | ATPase Na+/K+ transporting subunit beta 3 | 3.75 | 23.04 |
| 1389351_at | Lrrfip1 | LRR binding FLII interacting protein 1 | 11.25 | 56.46 |
| 1373730_at | Rbm33 | RNA binding motif protein 33 | 9 | 77.65 |
| 1390385_at | Glce | glucuronic acid epimerase | 0.75 | 70.99 |
| 1375506_at | Adck5 | aarF domain containing kinase 5 | 3 | 3.94 |
| 1370462_at | Hmmr | hyaluronan-mediated motility receptor | 2.25 | 35.69 |
| 1373653_at | Mtpap | mitochondrial poly polymerase | 6 | 47.31 |
| 1398341_at | Cisd3 | CDGSH iron sulfur domain 3 | 6 | 37.06 |
| 1367793_at | Ddt | D-dopachrome tautomerase | 2.25 | 2.67 |
| 1371980_at | Atad3a | ATPase family, AAA domain containing 3A | 4.5 | 24.84 |
| 1389625_at | Chchd4 /// LOC100361898 | coiled-coil-helix-coiled-coil-helix domain containing 4 | 5.25 | 73.45 |
| 1367706_at | Vdac1 | similar to voltage-dependent anion channel 1 | 0.75 | 33.49 |
| 1374842_at | Cebpz | CCAAT/enhancer binding protein zeta | 9.75 | 55.63 |
| 1367525_at | Thrap3 | thyroid hormone receptor associated protein 3 | 3 | 62.16 |
| 1392731_at | Tnfrsf1b | TNF receptor superfamily member 1B | 4.5 | 82.73 |
| 1374674_at | Atxn3 | ataxin 3 | 1.5 | 40.83 |
| 1383654_a_at | Fn3k | fructosamine 3 kinase | 1.5 | 22.14 |
| 1376098_a_at | LOC102547700 | uncharacterized LOC102547700 | 3.75 | 68.25 |
| 1367537_at | Eif4enif1 | Eukaryotic translation initiation | 0.75 | 27.42 |
| 1387105_at | Zfp422 | zinc finger protein 422 | 3 | 68.04 |
| 1373603_at | Tsr3 | TSR3, 20S rRNA accumulation | 9 | 45.82 |
| 1367578_at | Prdx2 | peroxiredoxin 2 | 2.25 | 15.37 |
| 1368232_at | Mvk | mevalonate kinase | 11.25 | 36.63 |
| 1367903_at | Hmox2 | heme oxygenase 2 | 1.5 | 23.40 |
| 1371301_at | LOC100360449 /// LOC100364457 /// Rpl9 | ribosomal protein L9-like | 3 | 27.13 |
| 1370881_at | Tst | thiosulfate sulfurtransferase | 6.75 | 16.82 |
| 1372115_at | Ubr2 | ubiquitin protein ligase E3 component n-recognin 2 | 4.5 | 58.42 |
| 1388978_at | Tmem30a | Transmembrane Protein 30A | 7.5 | 60.12 |
| 1398326_at | Chchd10 /// LOC103694872 | coiled-coil-helix-coiled-coil-helix domain containing 10 | 7.5 | 54.71 |
| 1372832_at | Stx2 | syntaxin 2 | 2.25 | 60.66 |
| 1372088_at | Ppfibp1 | PPFIA binding protein 1 | 1.5 | 52.76 |
| 1371632_at | Coro1c | Coronin, actin-binding protein 1C | 8.25 | 74.93 |
| 1376927_at | Lrrc14b | leucine rich repeat containing 14B | 9 | 1.22 |
| 1368244_at | As3mt | arsenite methyltransferase | 6.75 | 37.98 |
| 1388102_at | Ptgr1 | Dithiolethione-inducible gene-1 | 4.5 | 70.47 |
| 1369664_at | Avpr1a | arginine vasopressin receptor 1A | 10.5 | 13.60 |
| 1398756_at | LOC300303 /// Npm1 | nucleophosmin 1 | 8.25 | 34.29 |
| 1373859_at | Yipf6 | Yip1 Domain Family Member 6 | 5.25 | 45.27 |
| 1376700_at | Lima1 | LIM domain and actin binding 1 | 1.5 | 73.36 |
| 1371705_at | Vps26a | VPS26 retromer complex component A | 3.75 | 74.93 |
| 1372347_at | Skil | SKI-like proto-oncogene | 3.75 | 69.00 |
| 1368458_at | Cyp7a1 | Cytochrome P450 (cholesterol hydroxylase 7 alpha) | 3 | 66.23 |
| 1388153_at | Acsl1 | acyl-CoA synthetase long-chain family member 1 | 12 | 17.45 |
| 1369934_at | Ppib | peptidylprolyl isomerase B | 3 | 52.38 |
| 1368062_at | Ap3m1 | adaptor-related protein complex 3, mu 1 subunit | 10.5 | 71.37 |
| 1374912_at | Kif2c | kinesin family member 2C | 0.75 | 82.24 |
| 1373929_at | Mrps7 | mitochondrial ribosomal protein S7 | 0.75 | 52.49 |
| 1371833_at | Bri3 | brain protein I3 | 4.5 | 84.49 |
| 1390224_at | Ikbkg | Inhibitor Of Nuclear Factor Kappa B Kinase Subunit Gamma | 2.25 | 52.82 |
| 1374805_at | Dscc1 | DNA replication and sister chromatid cohesion 1 | 9.75 | 84.64 |
| 1376496_at | Apol9a | apolipoprotein L 9a | 1.5 | 56.38 |
| 1375412_at | Arsb | arylsulfatase B | 10.5 | 11.61 |
| 1372024_at | Mafg | MAF bZIP transcription factor G | 3 | 53.44 |
| 1371355_at | Ndufa8 | NADH:ubiquinone oxidoreductase subunit A8 | 2.25 | 30.07 |
| 1371992_at | Babam1 | BRISC and BRCA1 A complex member 1 | 3.75 | 35.61 |
| 1373898_at | Pan3 | PAN3 Poly(A) Specific Ribonuclease Subunit | 0.75 | 68.64 |
| 1371580_at | Erlin1 | SPFH domain family, member 1 | 7.5 | 70.34 |
| 1376567_at | Mad1l1 | MAD1 mitotic arrest deficient like 1 | 7.5 | 88.90 |
| 1376340_a_at | Tssc4 | tumor suppressing subtransferable candidate 4 | 0.75 | 47.84 |
| 1388365_at | Atp6v0d1 | ATPase H+ transporting V0 subunit D1 | 7.5 | 29.15 |
| 1371585_at | Gspt1 /// LOC100911685 | G1 to S phase transition 1 | 6 | 42.58 |
| 1377937_at | Mrps14 | mitochondrial ribosomal protein S14 | 8.25 | 74.52 |
| 1390026_at | Bag3 | Bcl2-associated athanogene 3 | 1.5 | 42.16 |
| 1376585_at | Mrpl50 | mitochondrial ribosomal protein L50 | 6 | 38.05 |
| 1388587_at | Ier3 | immediate early response 3 | 1.5 | 75.65 |
| 1389336_at | Pop5 | POP5 homolog, ribonuclease P/MRP subunit | 3.75 | 54.86 |
| 1389984_at | Jarid2 | jumonji and AT-rich interaction domain containing 2 | 3.75 | 81.13 |
| 1372012_at | Dhcr24 | 24-Dehydrocholesterol Reductase | 4.5 | 55.28 |
| 1372401_at | Nans | N-acetylneuraminate synthase | 3.75 | 70.16 |
| 1368662_at | Rnf39 | ring finger protein 39 | 10.5 | 17.49 |
| 1371489_at | Rnf4 | ring finger protein 4 | 2.25 | 46.31 |
| 1386944_a_at | G6pc | glucose-6-phosphatase, catalytic subunit | 8.25 | 77.75 |
| 1388566_at | Lasp1 | LIM and SH3 protein 1 | 2.25 | 6.03 |
| 1386899_at | Ctsh | cathepsin H | 4.5 | 39.25 |
| 1390109_at | LOC100910069 /// LOC102554001 /// LOC680724 | uncharacterized LOC100910069 | 6.75 | 78.81 |
| 1373784_at | Cct8 | chaperonin containing TCP1 subunit 8 | 9.75 | 47.36 |
| 1389033_at | Uqcc2 | ubiquinol-cytochrome c reductase complex assembly factor 2 | 0 | 58.05 |
| 1374574_at | Fam214a | family with sequence similarity 214, member A | 4.5 | 42.86 |
| 1373244_at | Tmub2 | transmembrane and ubiquitin-like domain containing 2 | 5.25 | 42.95 |
| 1398760_at | LOC100359498 /// LOC103690996 /// Rpl35a | ribosomal protein L35a-like | 3 | 61.09 |
| 1371765_at | Hist3h2a | histone cluster 3, H2a | 12 | 68.38 |
| 1388739_at | Ubr7 | ubiquitin protein ligase E3 component n-recognin 7 | 2.25 | 70.14 |
| 1389586_at | Ednrb | Endothelin Receptor Type B | 7.5 | 53.07 |
| 1372223_at | Cpeb4 | Cytoplasmic Polyadenylation Element Binding Protein 4 | 4.5 | 50.32 |
| 1375536_at | Numb | NUMB, endocytic adaptor protein | 4.5 | 68.02 |
| 1389549_at | Prosc | proline synthetase co-transcribed homolog | 3.75 | 46.23 |
| 1370376_a_at | Ybx3 | Y box binding protein 3 | 2.25 | 50.72 |
| 1386871_at | Gpx4 | glutathione peroxidase 4 | 1.5 | 17.53 |
| 1388511_at | Arap1 | ArfGAP with RhoGAP domain, ankyrin repeat and PH domain 1 | 1.5 | 51.38 |
| 1375726_at | Lmo7 | LIM domain 7 | 5.25 | 69.17 |
| 1373037_at | Ube2l6 | ubiquitin-conjugating enzyme E2L 6 | 0.75 | 30.07 |
| 1368168_at | Slc34a2 | solute carrier family 34 member 2 | 1.5 | 87.31 |
| 1387779_at | Mybbp1a | MYB binding protein 1a | 5.25 | 59.32 |
| 1390865_at | Cadps2 | calcium dependent secretion activator 2 | 9 | 65.42 |
| 1372485_at | Pcbd1 | pterin-4 alpha-carbinolamine dehydratase 1 | 6.75 | 1.64 |
| 1372069_at | Kank1 | KN motif and ankyrin repeat domains 1 | 3.75 | 83.00 |
| 1374679_at | Higd1b | HIG1 hypoxia inducible domain family, member 1B | 8.25 | 56.08 |
| 1374323_at | Bccip | BRCA2 and CDKN1A interacting protein | 3.75 | 30.36 |
| 1375520_at | Copz1 | coatomer protein complex, subunit zeta 1 | 3.75 | 19.20 |
| 1371598_at | Adipor2 | adiponectin receptor 2 | 2.25 | 66.22 |
| 1388756_at | Ppcs | phosphopantothenoylcysteine synthetase | 12 | 39.93 |
| 1390237_at | Timm8a1 | translocase of inner mitochondrial membrane 8 homolog A1 | 9 | 69.69 |
| 1375120_at | Id4 | inhibitor of DNA binding 4, HLH protein | 11.25 | 55.74 |
| 1368574_at | Adra1b | adrenoceptor alpha 1B | 7.5 | 30.08 |
| 1373165_at | Zmat1 | zinc finger, matrin-type 1 | 1.5 | 35.21 |
| 1388483_at | Cfl2 | cofilin 2 | 3 | 14.42 |
| 1398309_at | Pigl | phosphatidylinositol glycan anchor biosynthesis, class L | 0.75 | 35.06 |
| 1399046_at | Top1 | topoisomerase I | 0.75 | 51.41 |
| 1377209_at | Klhl25 /// LOC103695026 | kelch-like family member 25 | 0.75 | 60.00 |
| 1388629_at | Impdh2 | Inosine 5-monophosphate dehydrogenase 2 | 3 | 78.60 |
| 1375224_at | Phlda3 | pleckstrin homology-like domain, family A, member 3 | 0.75 | 88.44 |
| 1389329_at | Lgals8 | galectin 8 | 3 | 64.32 |
| 1387665_at | Bhmt | betaine-homocysteine S-methyltransferase | 3 | 18.46 |
| 1374870_at | Col27a1 | collagen type XXVII alpha 1 chain | 0.75 | 56.84 |
| 1371041_at | Ndufv2 | NADH:ubiquinone oxidoreductase core subunit V2 | 0.75 | 38.14 |
| 1368552_at | Grpel1 | GrpE-like 1, mitochondrial | 0 | 51.98 |
| 1377353_a_at | Tnfsf13 | TNF superfamily member 13 | 3.75 | 59.17 |
| 1367715_at | Tnfrsf1a | TNF receptor superfamily member 1A | 12 | 29.49 |
| 1389525_at | Rnf149 | ring finger protein 149 | 0 | 59.81 |
| 1399084_at | Dhx16 | DEAH-box helicase 16 | 9.75 | 70.94 |
| 1368109_at | St3gal5 | ST3 beta-galactoside alpha-2,3-sialyltransferase 5 | 9 | 64.36 |
| 1373647_at | LOC103689968 /// LOC103690028 /// Zfp622 | zinc finger protein 622 | 9 | 50.00 |
| 1376869_at | Tle4 | transducin-like enhancer of split 4 | 3 | 11.49 |
| 1376588_at | Mtmr12 | myotubularin related protein 12 | 10.5 | 42.38 |
| 1387894_at | Gata4 | GATA Binding Protein 4 | 1.5 | 9.22 |
| 1398755_at | Atp6v0c | ATPase H+ Transporting V0 Subunit C | 3 | 7.61 |
| 1388612_at | Ociad1 | OCIA Domain Containing 1 | 6 | 45.06 |
| 1370168_at | Ywhaq | Tyrosine 3-Monooxygenase/Tryptophan 5-Monooxygenase Activation Protein Theta | 6.75 | 49.78 |

### Table D: Common genes retaining circadian expression in Muscle and Adipose. Δφ indicates their phase difference in hours and ΔΑ their amplitude difference in % change

| **ProbeID** | **Symbol** | **GeneName** | **Δφ [hr]** | **ΔΑ [%]** |
| --- | --- | --- | --- | --- |
| 1371864_at | --- | --- | 0.75 | 14.18 |
| 1375760_at | --- | -- | 2.25 | 14.89 |
| 1389085_at | --- | --- | 0.75 | 14.95 |
| 1376574_at | --- | --- | 1.5 | 15.17 |
| 1376341_at | --- | --- | 1.5 | 62.44 |
| 1373225_at | --- | --- | 0 | 29.67 |
| 1388479_at | --- | --- | 0 | 59.81 |
| 1374307_at | --- | --- | 2.25 | 24.45 |
| 1376944_at | --- | --- | 1.5 | 13.25 |
| 1389562_at | --- | --- | 0 | 2.58 |
| 1372178_at | --- | --- | 1.5 | 39.58 |
| 1375879_at | --- | --- | 4.5 | 1.24 |
| 1376848_at | --- | --- | 5.25 | 19.51 |
| 1390743_at | --- | --- | 0.75 | 37.97 |
| 1374429_at | --- | --- | 0.75 | 47.27 |
| 1372136_at | --- | --- | 2.25 | 2.37 |
| 1386185_at | --- | --- | 5.25 | 29.12 |
| 1386186_s_at | --- | --- | 0.75 | 28.74 |
| 1399082_at | --- | --- | 3.75 | 10.47 |
| 1388804_at | --- | --- | 2.25 | 14.99 |
| 1375781_at | --- | -- | 2.25 | 48.67 |
| 1368303_at | Per2 | period circadian clock 2 | 0 | 44.08 |
| 1370510_a_at | Arntl | aryl hydrocarbon receptor nuclear translocator-like | 0.75 | 15.85 |
| 1370816_at | Nr1d1 | nuclear receptor subfamily 1, group D, member 1 | 0.75 | 55.65 |
| 1387874_at | Dbp | D-box binding PAR bZIP transcription factor | 0.75 | 14.71 |
| 1370541_at | Nr1d2 | nuclear receptor subfamily 1, group D, member 2 | 0.75 | 37.83 |
| 1398246_s_at | Fcgr2a | low affinity immunoglobulin gamma Fc region receptor III-like | 0.75 | 37.93 |
| 1370912_at | Hspa1a | Heat shock 70kD protein 1B | 0.75 | 64.53 |
| 1373542_at | Sphk2 | sphingosine kinase 2 | 0 | 40.72 |
| 1367771_at | Tsc22d3 | Glucocorticoid-induced leucine zipper | 0.75 | 55.28 |
| 1374855_at | Per1 | period circadian clock 1 | 0.75 | 37.39 |
| 1370209_at | Klf9 | Kruppel-like factor 9 | 2.25 | 17.08 |
| 1388901_at | Fkbp5 | FK506 binding protein 5 | 4.5 | 41.19 |
| 1368571_at | Clip2 | CAP-GLY domain containing linker protein 2 | 0.75 | 34.20 |
| 1368304_at | Fmo3 | flavin containing monooxygenase 3 | 0.75 | 21.82 |
| 1368247_at | Hspa1a | heat shock 70kD protein 1A | 3.75 | 52.40 |
| 1368488_at | Nfil3 | nuclear factor, interleukin 3 regulated | 0 | 32.76 |
| 1398370_at | Adarb1 | adenosine deaminase, RNA-specific, B1 | 0.75 | 54.60 |
| 1370847_at | Spon2 | spondin 2 | 0.75 | 45.38 |
| 1373158_at | Gpr146 | G protein-coupled receptor 146 | 0 | 64.73 |
| 1371832_at | Leo1 | LEO1 homolog, Paf1/RNA polymerase II complex component | 3 | 1.98 |
| 1371913_at | Tgfbi | transforming growth factor, beta induced | 0.75 | 44.63 |
| 1375677_at | Tob2 | transducer of ERBB2, 2 | 1.5 | 28.64 |
| 1369919_at | Tef | TEF, PAR bZIP transcription factor | 1.5 | 3.17 |
| 1398365_at | Tppp3 | tubulin polymerization-promoting protein family member 3 | 1.5 | 55.46 |
| 1389456_at | Clock | clock circadian regulator | 0.75 | 17.66 |
| 1368025_at | Ddit4 | DNA-damage-inducible transcript 4 | 4.5 | 46.28 |
| 1370050_at | Atp2b1 | ATPase plasma membrane Ca2+ transporting 1 | 9 | 17.90 |
| 1390628_at | Cpeb2 | #N/A | 2.25 | 34.65 |
| 1370570_at | Nrp1 | Neuropilin 1 | 0 | 51.43 |
| 1370954_at | P4ha1 | prolyl 4-hydroxylase subunit alpha 1 | 3.75 | 67.76 |
| 1371583_at | Rbm3 | RNA binding motif (RNP1, RRM) protein 3 | 0 | 25.77 |
| 1368511_at | Bhlhe41 | Basic helix-loop-helix domain containing, class B, 3 | 1.5 | 7.30 |
| 1372390_at | Peg3 | paternally expressed 3 | 6 | 58.28 |
| 1398255_at | Slc15a2 | solute carrier family 15 member 2 | 2.25 | 44.97 |
| 1376071_at | LOC100909795 | colorectal mutant cancer protein-like | 2.25 | 18.13 |
| 1376435_at | Loxl4 | Lysyl Oxidase Like 4 | 1.5 | 11.76 |
| 1372091_at | Mid1ip1 | MID1 interacting protein 1 | 4.5 | 35.02 |
| 1373866_at | Coq10b | coenzyme Q10B | 2.25 | 70.21 |
| 1391808_at | Arrdc4 | arrestin domain containing 4 | 0 | 9.64 |
| 1387681_at | Ucp3 | uncoupling protein 3 | 2.25 | 13.40 |
| 1368549_at | Hbp1 | HMG-box transcription factor 1 | 1.5 | 28.23 |
| 1398819_at | Dnaja1 | DNAJ (Hsp40) homolog, subfamily A, member 1 | 0.75 | 28.28 |
| 1368249_at | Klf15 | Kruppel-like factor 15 | 2.25 | 38.08 |
| 1388525_at | Pik3ip1 | phosphoinositide-3-kinase interacting protein 1 | 0 | 28.96 |
| 1367568_a_at | Mgp | matrix Gla protein | 0.75 | 21.01 |
| 1367922_at | Adam17 | ADAM metallopeptidase domain 17 | 2.25 | 5.06 |
| 1372750_at | Fst | #N/A | 0 | 52.28 |
| 1388666_at | Enc1 | ectodermal-neural cortex 1 | 0 | 57.86 |
| 1389020_at | Islr | immunoglobulin superfamily containing leucine-rich repeat | 2.25 | 52.09 |
| 1370283_at | Hspa5 | heat shock protein family A member 5 | 3 | 17.46 |
| 1389836_a_at | Timp3 | TIMP metallopeptidase inhibitor 4 | 3.75 | 12.57 |
| 1388583_at | Cxcl12 | C-X-C motif chemokine ligand 12 | 2.25 | 8.12 |
| 1387669_a_at | Ephx1 | Epoxide hydrolase 1, | 1.5 | 12.96 |
| 1372520_at | Mcl1 | BCL2 family apoptosis regulator | 4.5 | 16.30 |
| 1371237_a_at | Mt1a | metallothionein 1 | 0.75 | 47.15 |
| 1388924_at | Angptl4 | Anigopoietin-like 4 | 0.75 | 32.01 |
| 1390171_at | Fam76a | family with sequence similarity 76, member A | 0.75 | 31.50 |
| 1376645_at | Medag | mesenteric estrogen-dependent adipogenesis | 3 | 49.54 |
| 1367802_at | Sgk1 | serum/glucocorticoid regulated kinase 1 | 5.25 | 4.95 |
| 1370268_at | Kcna5 | potassium voltage-gated channel subfamily A member 5 | 3.75 | 14.79 |
| 1398431_at | Car8 | Carbonic anhydrase viii | 1.5 | 17.57 |
| 1388949_at | Nxpe1 | neurexophilin and PC-esterase domain family, member 1 | 1.5 | 26.23 |
| 1372426_at | Adamtsl4 | ADAMTS-like 4 | 2.25 | 51.36 |
| 1370381_at | Pnrc1 | Proline-rich nuclear receptor coactivator 1, F-box coactivator 1, F-box protein 11 | 0.75 | 4.12 |
| 1374650_at | Nedd9 | neural precursor cell expressed, developmentally down-regulated 9 | 3 | 9.71 |
| 1387703_a_at | Usp2 | Ubiquitin-specific peptidase 2 | 0.75 | 33.23 |
| 1398998_at | RGD1309748 | -- | 2.25 | 77.46 |
| 1372856_at | Plin4 | perilipin 4 | 11.25 | 4.56 |
| 1388384_at | Rusc2 | Dynein light chain LC8- type 1 | 2.25 | 38.97 |
| 1391560_at | Hivep1 | human immunodeficiency virus type I enhancer binding protein 1 | 9 | 24.17 |
| 1373266_at | Fam107a | family with sequence similarity 107, member A | 1.5 | 8.55 |
| 1374636_at | Jade1 | PHD finger protein 17 | 1.5 | 27.75 |
| 1388672_at | Zcchc24 | zinc finger CCHC-type containing 24 | 1.5 | 24.33 |
| 1375658_at | Sash1 | SAM and SH3 domain containing 1 | 1.5 | 24.24 |
| 1369956_at | Ifngr1 | interferon gamma receptor 1 | 0 | 22.91 |
| 1373407_at | Gramd4 | GRAM domain containing 4 | 0.75 | 26.54 |
| 1388795_at | Tppp | tubulin polymerization promoting protein | 6 | 30.88 |
| 1387675_at | Plau | plasminogen activator, urokinase | 2.25 | 14.08 |
| 1367741_at | Herpud1 | homocysteine inducible ER protein with ubiquitin like domain 1 | 2.25 | 19.54 |
| 1367631_at | Ctgf | connective tissue growth factor | 0 | 18.24 |
| 1376770_at | Efhd1 | EF-hand domain family, member D1 | 0.75 | 20.83 |
| 1368394_at | Sfrp4 | secreted frizzled-related protein 4 | 3.75 | 68.08 |
| 1388589_at | Dot1l | DOT1 like histone lysine methyltransferase | 0.75 | 45.23 |
| 1368080_at | Rgcc | regulator of cell cycle | 3 | 18.75 |
| 1370215_at | C1qb | complement C1q B chain | 1.5 | 9.48 |
| 1372069_at | Kank1 | KN motif and ankyrin repeat domains 1 | 0.75 | 45.48 |
| 1390148_a_at | Zfp395 | zinc finger protein 395 | 0.75 | 41.78 |
| 1368144_at | Rgs2 | Regulator of g-protein signaling 2, 24kda | 0 | 3.56 |
| 1384217_at | Zhx2 | zinc fingers and homeoboxes 2 | 4.5 | 44.28 |
| 1370428_x_at | RT1-A2 | RT1 class Ib, locus Aw2 | 0 | 97.02 |
| 1392490_at | Tmem57 | Transmembrane protein 57 | 0.75 | 44.10 |
| 1371029_at | Pkd1 | polycystic kidney disease 1 | 3 | 34.35 |
| 1369200_at | Nt5e | 5' nucleotidase, ecto | 7.5 | 34.82 |
| 1369182_at | F3 | coagulation factor III, tissue factor | 0.75 | 51.79 |
| 1388686_at | Rcan1 | Regulator of calcineurin 1 | 0.75 | 58.90 |
| 1375916_at | Pcmtd2 | protein-L-isoaspartate O-methyltransferase domain containing 2 | 0 | 20.19 |
| 1376924_a_at | Palmd | palmdelphin | 5.25 | 17.00 |
| 1386987_at | Il6r | Interleukin 6 receptor | 3 | 62.93 |
| 1373092_at | Tgfbr3 | transforming growth factor beta receptor 3 | 0.75 | 17.20 |
| 1370399_at | Cyp4b1 | cytochrome P450, family 4, subfamily b, polypeptide 1 | 2.25 | 62.05 |
| 1372026_at | Atg101 | autophagy related 101 | 1.5 | 7.69 |
| 1387060_at | Klf6 | Kruppel-like factor 6 | 5.25 | 17.96 |
| 1371754_at | Slc25a25 | solute carrier family 25 member 25 | 3.75 | 58.76 |
| 1371150_at | Ccnd1 | cyclin D1 | 0.75 | 81.27 |
| 1390391_at | Pfkfb3 | 6-phosphofructo-2-kinase/fructose-2,6-biphosphatase 3 | 1.5 | 28.45 |
| 1367638_at | Mlycd | malonyl-CoA decarboxylase | 7.5 | 5.43 |
| 1389528_s_at | Jun | Jun proto-oncogene, AP-1 transcription factor subunit | 1.5 | 38.36 |
| 1371194_at | Tnfaip6 | TNF alpha induced protein 6 | 0 | 54.50 |
| 1376621_at | Wdr26 | WD repeat domain 26 | 2.25 | 36.30 |
| 1374575_at | Creb3l1 | cAMP responsive element binding protein 3-like 1 | 2.25 | 21.05 |
| 1370336_at | Osgin1 | oxidative stress induced growth inhibitor 1 | 4.5 | 26.50 |
| 1387294_at | Sh3bp5 | SH3-domain binding protein 5 | 6 | 79.98 |
| 1383169_at | LOC103691479 | Leukemia inhibitory factor receptor | 8.25 | 60.47 |
| 1368283_at | Ehhadh | Enoyl-coenzyme A, hydratase/3- hydroxyacyl coenzyme A dehydrogenase | 1.5 | 36.15 |
| 1374671_at | Traf3ip2 | Traf3 interacting protein 2 | 1.5 | 42.94 |
| 1389423_at | Ddr2 | discoidin domain receptor tyrosine kinase 2 | 4.5 | 38.32 |
| 1386897_at | Prmt1 | protein arginine methyltransferase 1-like | 0.75 | 30.84 |
| 1373644_at | Nfib | #N/A | 6.75 | 19.57 |
| 1391062_at | Elp4 | elongator acetyltransferase complex subunit 4 | 3 | 25.26 |
| 1367759_at | H1f0 | H1 histone family, member 0 | 2.25 | 28.00 |
| 1367632_at | Glul | Glutamate-ammonia | 0.75 | 67.90 |
| 1390042_at | Tmem140 | -- | 2.25 | 23.93 |
| 1389538_at | Nfkbia | NFKB inhibitor alpha | 1.5 | 38.81 |
| 1373807_at | Vegfa | vascular endothelial growth factor A | 1.5 | 42.24 |
| 1398767_at | Ubc | Ubiquitin B, C | 1.5 | 83.87 |
| 1367704_at | Ap2b1 | adaptor-related protein complex 2, beta 1 subunit | 10.5 | 74.77 |
| 1387024_at | Dusp6 | dual specificity phosphatase 6 | 9 | 24.17 |
| 1371836_at | Rab5c | RAB5C, member RAS oncogene family | 11.25 | 4.55 |
| 1387521_at | Pdcd4 | programmed cell death 4 | 0 | 29.67 |
| 1374542_at | Fam13c | family with sequence similarity 13, member C | 2.25 | 52.14 |
| 1372701_at | Hsp90aa1 | heat shock protein 1, alpha-like | 3 | 49.57 |
| 1372341_at | Slc25a36 | solute carrier family 25 member 36 | 3.75 | 46.60 |
| 1374421_at | Baz1b | bromodomain adjacent to zinc finger domain, 1B | 3.75 | 26.79 |
| 1369954_at | Idh1 | isocitrate dehydrogenase (NADP(+)) 1, cytosolic | 0.75 | 7.25 |
| 1370097_a_at | Cxcr4 | C-X-C motif chemokine receptor 4 | 2.25 | 26.73 |
| 1375037_at | Ppp6r3 | protein phosphatase 6, regulatory subunit 3 | 1.5 | 7.08 |
| 1370217_at | Ddr1 | #N/A | 1.5 | 19.70 |
| 1371537_at | B4galt5 | beta-1,4-galactosyltransferase 5 | 1.5 | 15.88 |
| 1368645_at | Ptpn1 | protein tyrosine phosphatase, non-receptor type 1 | 3.75 | 24.37 |
| 1390709_at | Trio | trio Rho guanine nucleotide exchange factor | 9 | 44.80 |
| 1377334_at | RT1-Ba | RT1 class II, locus Ba | 0 | 92.51 |
| 1371131_a_at | Txnip | thioredoxin interacting protein | 3.75 | 58.02 |
| 1390406_at | Arhgap18 | Rho GTPase activating protein 18 | 0.75 | 31.71 |
| 1367668_a_at | Scd2 | acyl-CoA desaturase 2-like | 3 | 33.06 |
| 1373240_at | Dhrs3 | Dehydrogenase/reductase 3 | 3.75 | 9.00 |
| 1369943_at | Tgm2 | Transglutaminase 2 | 2.25 | 50.58 |
| 1372835_at | Rhoj | #N/A | 2.25 | 47.65 |
| 1399022_at | Clk1 | CDC-like kinase 1 | 3.75 | 21.05 |
| 1375211_at | Rnaset2 | Ribonuclease T2 | 2.25 | 43.41 |
| 1373087_at | March7 | membrane associated ring-CH-type finger 7 | 0.75 | 5.85 |
| 1387116_at | Dnajb9 | DnaJ heat shock protein family member B9 | 1.5 | 27.01 |

### Table E: Common genes retaining circadian expression in Muscle and Lung. Δφ indicates their phase difference in hours and ΔΑ their amplitude difference in % change

| **ProbeID** | **Symbol** | **GeneName** | **Δφ [hr]** | **ΔΑ [%]** |
| --- | --- | --- | --- | --- |
| 1371864_at | --- | --- | 0.75 | 26.24 |
| 1389085_at | --- | --- | 0.75 | 14.49 |
| 1376341_at | --- | --- | 1.5 | 70.94 |
| 1373225_at | --- | --- | 1.5 | 36.54 |
| AFFX-r2-Ec-bioB-M_at | --- | --- | 12 | 27.75 |
| 1376574_at | --- | --- | 1.5 | 6.46 |
| 1390300_at | --- | --- | 2.25 | 25.57 |
| 1388527_at | --- | --- | 2.25 | 34.17 |
| 1373944_at | --- | --- | 0.75 | 63.49 |
| 1384717_at | --- | --- | 0.75 | 20.04 |
| AFFX-r2-Ec-bioC-3_at | --- | --- | 11.25 | 16.75 |
| 1376938_at | --- | --- | 4.5 | 50.57 |
| 1372011_at | --- | --- | 0.75 | 53.27 |
| 1390743_at | --- | --- | 0.75 | 7.14 |
| 1399082_at | --- | --- | 1.5 | 41.16 |
| 1376848_at | --- | --- | 0.75 | 48.26 |
| 1373873_at | --- | --- | 0 | 85.41 |
| 1377149_at | --- | --- | 2.25 | 26.59 |
| 1374307_at | --- | --- | 6 | 38.51 |
| 1390851_at | --- | --- | 2.25 | 41.98 |
| 1376966_at | --- | --- | 0.75 | 70.76 |
| 1375043_at | --- | --- | 4.5 | 18.48 |
| 1371362_at | --- | --- | 4.5 | 31.70 |
| 1389618_at | --- | --- | 3.75 | 81.99 |
| 1390215_at | --- | --- | 0.75 | 15.57 |
| 1372993_at | --- | --- | 0 | 16.52 |
| 1374089_at | --- | --- | 3.75 | 11.47 |
| 1372136_at | --- | --- | 0.75 | 30.18 |
| 1386185_at | --- | --- | 5.25 | 43.67 |
| 1389794_at | --- | --- | 9.75 | 50.82 |
| 1374521_at | --- | --- | 9 | 2.44 |
| 1372031_at | --- | --- | 0 | 48.53 |
| 1372220_at | --- | --- | 6 | 52.97 |
| 1388479_at | --- | --- | 4.5 | 25.99 |
| 1375728_at | --- | --- | 0.75 | 61.04 |
| 1372385_at | --- | --- | 0.75 | 69.51 |
| 1392566_at | --- | --- | 6.75 | 85.08 |
| 1376568_at | --- | --- | 2.25 | 3.24 |
| 1377167_at | --- | --- | 6 | 72.54 |
| 1377013_at | --- | --- | 6.75 | 5.70 |
| 1380200_at | --- | --- | 8.25 | 64.35 |
| 1380859_at | --- | --- | 3 | 27.15 |
| 1386186_s_at | --- | --- | 9.75 | 31.87 |
| 1390430_at | Nr1d2 | nuclear receptor subfamily 1, group D, member 2 | 0 | 48.52 |
| 1367771_at | Tsc22d3 | Glucocorticoid-induced leucine zipper | 3 | 64.84 |
| 1368303_at | Per2 | period circadian clock 2 | 0.75 | 41.51 |
| 1368304_at | Fmo3 | flavin containing monooxygenase 3 | 0.75 | 39.19 |
| 1370816_at | Nr1d1 | nuclear receptor subfamily 1, group D, member 1 | 0.75 | 73.50 |
| 1370510_a_at | Arntl | aryl hydrocarbon receptor nuclear translocator-like | 0.75 | 29.12 |
| 1370847_at | Spon2 | spondin 2 | 0.75 | 77.10 |
| 1389836_a_at | Timp3 | TIMP metallopeptidase inhibitor 4 | 6.75 | 11.78 |
| 1369919_at | Tef | TEF, PAR bZIP transcription factor | 1.5 | 10.98 |
| 1370209_at | Klf9 | Kruppel-like factor 9 | 1.5 | 21.17 |
| 1387874_at | Dbp | D-box binding PAR bZIP transcription factor | 0.75 | 66.65 |
| 1367741_at | Herpud1 | homocysteine inducible ER protein with ubiquitin like domain 1 | 2.25 | 49.25 |
| 1367577_at | Hspb1 | Heat shock 27kda protein 1 | 4.5 | 3.39 |
| 1368249_at | Klf15 | Kruppel-like factor 15 | 3 | 52.21 |
| 1367631_at | Ctgf | connective tissue growth factor | 1.5 | 40.47 |
| 1372752_at | Tspan4 | tetraspanin-4-like | 0 | 50.68 |
| 1388901_at | Fkbp5 | FK506 binding protein 5 | 6 | 46.23 |
| 1371583_at | Rbm3 | RNA binding motif (RNP1, RRM) protein 3 | 2.25 | 32.70 |
| 1370283_at | Hspa5 | heat shock protein family A member 5 | 3 | 42.87 |
| 1374575_at | Creb3l1 | cAMP responsive element binding protein 3-like 1 | 0.75 | 47.16 |
| 1368488_at | Nfil3 | nuclear factor, interleukin 3 regulated | 0 | 22.30 |
| 1367755_at | Cdo1 | Cysteine dioxygenase, type i | 0.75 | 81.80 |
| 1390199_at | Clock | clock circadian regulator | 3 | 28.70 |
| 1367922_at | Adam17 | ADAM metallopeptidase domain 17 | 1.5 | 33.08 |
| 1376562_at | Tnik | TRAF2 and NCK interacting kinase | 0.75 | 8.04 |
| 1371832_at | Leo1 | LEO1 homolog, Paf1/RNA polymerase II complex component | 3 | 20.63 |
| 1398773_at | Khdrbs1 | KH RNA binding domain containing, signal transduction associated 1 | 5.25 | 46.79 |
| 1386937_at | Atp1b1 | Atpase, na+/k+ transporting, β 1 | 1.5 | 65.41 |
| 1374855_at | Per1 | period circadian clock 1 | 0.75 | 27.16 |
| 1374650_at | Nedd9 | neural precursor cell expressed, developmentally down-regulated 9 | 6 | 15.53 |
| 1369943_at | Tgm2 | Transglutaminase 2 | 2.25 | 62.24 |
| 1387109_at | Por | P450 (cytochrome) oxidoreductase | 2.25 | 29.14 |
| 1370019_at | Sult1a1 | sulfotransferase family 1A member 1 | 0.75 | 16.72 |
| 1398431_at | Car8 | Carbonic anhydrase viii | 4.5 | 11.59 |
| 1374752_at | Mdfic | Myod family inhibitor domain containing | 1.5 | 19.36 |
| 1368342_at | Ampd3 | adenosine monophosphate deaminase 3 | 2.25 | 62.39 |
| 1367759_at | H1f0 | H1 histone family, member 0 | 4.5 | 41.64 |
| 1387703_a_at | Usp2 | Ubiquitin-specific peptidase 2 | 0 | 18.08 |
| 1374798_at | Tor1aip2 | torsin 1A interacting protein 2 | 1.5 | 48.81 |
| 1386946_at | Cpt1a | Carnitine palmitoyltransferase I | 0.75 | 32.46 |
| 1372480_at | Zfp462 | zinc finger protein 462 | 0 | 39.71 |
| 1388686_at | Rcan1 | Regulator of calcineurin 1 | 0.75 | 46.13 |
| 1368247_at | Hspa1a | heat shock 70kD protein 1A | 5.25 | 49.79 |
| 1387294_at | Sh3bp5 | SH3-domain binding protein 5 | 2.25 | 57.28 |
| 1373850_at | Smpdl3b | sphingomyelin phosphodiesterase, acid-like 3B | 6 | 19.93 |
| 1373595_at | Tmem43 | transmembrane protein 43 | 1.5 | 56.04 |
| 1376770_at | Efhd1 | EF-hand domain family, member D1 | 1.5 | 34.68 |
| 1372042_at | Cmtm3 | CKLF-like MARVEL transmembrane domain containing 3 | 6.75 | 54.71 |
| 1373087_at | March7 | membrane associated ring-CH-type finger 7 | 0.75 | 10.68 |
| 1370912_at | Hspa1a | Heat shock 70kD protein 1B | 3 | 51.85 |
| 1388395_at | G0s2 | G0/G1switch 2 | 0.75 | 63.61 |
| 1388426_at | Srebf1 | sterol regulatory element binding transcription factor 1 | 3 | 35.79 |
| 1370954_at | P4ha1 | prolyl 4-hydroxylase subunit alpha 1 | 2.25 | 46.89 |
| 1372565_at | Htra3 | HtrA serine peptidase 3 | 2.25 | 48.45 |
| 1391808_at | Arrdc4 | arrestin domain containing 4 | 3.75 | 8.31 |
| 1368144_at | Rgs2 | Regulator of g-protein signaling 2, 24kda | 9 | 9.82 |
| 1388792_at | Gadd45g | growth arrest and DNA-damage-inducible, gamma | 7.5 | 39.97 |
| 1388850_at | Hsp90aa1 | heat shock protein 90, alpha (cytosolic), class A member 1 | 1.5 | 10.15 |
| 1372701_at | Hsp90aa1 | heat shock protein 1, alpha-like | 1.5 | 2.61 |
| 1373364_at | Eif4g3 | eukaryotic translation initiation factor 4 gamma, 3 | 0.75 | 34.07 |
| 1375846_at | Xpr1 | #N/A | 6 | 44.32 |
| 1373672_at | Serpinb9 | serpin family B member 9 | 0.75 | 42.23 |
| 1371785_at | Tnfrsf12a | TNF receptor superfamily member 12A | 0.75 | 46.84 |
| 1373036_at | Iqgap2 | IQ motif containing GTPase activating protein 2 | 9.75 | 25.00 |
| 1373683_at | Fyn | FYN proto-oncogene, Src family tyrosine kinase | 0 | 12.61 |
| 1373887_at | Sf3b1 | #N/A | 2.25 | 3.43 |
| 1371237_a_at | Mt1a | metallothionein 1 | 1.5 | 76.88 |
| 1371554_at | Tcap | titin-cap | 0 | 88.00 |
| 1373143_at | RGD1309621 | -- | 4.5 | 46.26 |
| 1371202_a_at | Nfib | nuclear factor I/B | 3.75 | 37.61 |
| 1373092_at | Tgfbr3 | transforming growth factor beta receptor 3 | 3 | 19.89 |
| 1373282_at | Slc25a33 | solute carrier family 25 member 33 | 5.25 | 44.44 |
| 1372016_at | Gadd45b | growth arrest and DNA-damage-inducible, beta | 3 | 47.46 |
| 1372004_at | Hebp1 | Heme binding protein 1 | 0.75 | 6.09 |
| 1373542_at | Sphk2 | sphingosine kinase 2 | 0.75 | 7.18 |
| 1367609_at | Mif | Macrophage migration inhibitory factor | 0.75 | 44.36 |
| 1387060_at | Klf6 | Kruppel-like factor 6 | 3.75 | 36.41 |
| 1368511_at | Bhlhe41 | Basic helix-loop-helix domain containing, class B, 3 | 1.5 | 57.42 |
| 1370928_at | Litaf | lipopolysaccharide-induced TNF factor | 2.25 | 24.27 |
| 1369467_a_at | Pfkfb1 | 6-Phosphofructo-2-kinase/fructose-2,6-bisphosphatase 1 | 4.5 | 41.12 |
| 1373866_at | Coq10b | coenzyme Q10B | 2.25 | 72.18 |
| 1372056_at | Cmtm6 | CKLF-like MARVEL transmembrane domain containing 6 | 3 | 56.71 |
| 1372390_at | Peg3 | paternally expressed 3 | 1.5 | 9.55 |
| 1368016_at | Pecr | peroxisomal trans-2-enoyl-CoA reductase | 1.5 | 23.70 |
| 1389393_at | Abhd17c | abhydrolase domain containing 17C | 1.5 | 0.30 |
| 1368172_a_at | Lox | lysyl oxidase | 0 | 6.06 |
| 1389632_at | Rhobtb1 | Rho-related BTB domain containing 1 | 1.5 | 68.26 |
| 1373661_a_at | Cxcr4 | C-X-C motif chemokine receptor 4 | 1.5 | 26.23 |
| 1374029_at | Hrct1 | histidine rich carboxyl terminus 1 | 4.5 | 1.93 |
| 1390042_at | Tmem140 | -- | 1.5 | 11.25 |
| 1370336_at | Osgin1 | oxidative stress induced growth inhibitor 1 | 0 | 54.02 |
| 1389230_at | Arrdc3 | Arrestin domain containing 3 | 11.25 | 62.48 |
| 1375523_at | Marcks | myristoylated alanine rich protein kinase C substrate | 0 | 51.77 |
| 1388408_at | Prr13 | Proline rich 13 | 1.5 | 14.00 |
| 1368080_at | Rgcc | regulator of cell cycle | 0 | 28.28 |
| 1368571_at | Clip2 | CAP-GLY domain containing linker protein 2 | 0.75 | 31.53 |
| 1376645_at | Medag | mesenteric estrogen-dependent adipogenesis | 0 | 62.21 |
| 1375362_at | Sppl2a | signal peptide peptidase-like 2A | 3.75 | 4.22 |
| 1392490_at | Tmem57 | Transmembrane protein 57 | 2.25 | 24.26 |
| 1391428_at | Angpt1 | #N/A | 0.75 | 5.97 |
| 1369625_at | Aqp1 | Aquaporin 1 | 7.5 | 54.22 |
| 1368127_at | Neu2 | neuraminidase 2 | 3.75 | 1.66 |
| 1371729_at | Ypel5 | yippee-like 5 | 3.75 | 5.08 |
| 1367668_a_at | Scd2 | acyl-CoA desaturase 2-like | 3.75 | 15.69 |
| 1384217_at | Zhx2 | zinc fingers and homeoboxes 2 | 1.5 | 26.74 |
| 1390171_at | Fam76a | family with sequence similarity 76, member A | 5.25 | 7.88 |
| 1367950_at | Slc22a5 | solute carrier family 22 member 5 | 8.25 | 15.06 |
| 1383169_at | LOC103691479 | Leukemia inhibitory factor receptor ? | 6 | 63.74 |
| 1368025_at | Ddit4 | DNA-damage-inducible transcript 4 | 2.25 | 29.70 |
| 1388795_at | Tppp | tubulin polymerization promoting protein | 4.5 | 32.29 |
| 1367902_at | Gng11 | Guanine nucleotide binding protein , γ 11 | 4.5 | 33.13 |
| 1369044_a_at | Pde4b | phosphodiesterase 4B | 7.5 | 64.72 |
| 1373407_at | Gramd4 | GRAM domain containing 4 | 0 | 40.40 |
| 1371249_at | Xbp1 | X-box binding protein 1 | 3 | 33.71 |
| 1368549_at | Hbp1 | HMG-box transcription factor 1 | 2.25 | 55.65 |
| 1367568_a_at | Mgp | matrix Gla protein | 0 | 54.46 |
| 1398255_at | Slc15a2 | solute carrier family 15 member 2 | 7.5 | 27.63 |
| 1377457_a_at | Sorl1 | sortilin related receptor 1 | 0 | 9.74 |
| 1375916_at | Pcmtd2 | protein-L-isoaspartate O-methyltransferase domain containing 2 | 0.75 | 62.02 |
| 1372449_at | Slc8a1 | solute carrier family 8 member A1 | 1.5 | 33.91 |
| 1376646_at | Popdc2 | Popeye domain containing 2 | 3 | 43.97 |
| 1376189_at | Zmym1 | zinc finger MYM-type containing 1 | 5.25 | 28.90 |
| 1379550_a_at | Gtf2ird1 | general transcription factor II-I repeat domain-containing protein 1-like | 1.5 | 50.49 |
| 1387669_a_at | Ephx1 | Epoxide hydrolase 1, | 1.5 | 36.54 |
| 1398998_at | RGD1309748 | -- | 2.25 | 61.74 |
| 1370130_at | Rhoa | ras homolog family member A | 6 | 13.81 |
| 1386864_at | Pgam1 | phosphoglycerate mutase 1 | 5.25 | 72.56 |
| 1390217_at | Ralgapa1 | Ral GTPase activating protein catalytic alpha subunit 1 | 2.25 | 29.19 |
| 1390117_at | Ypel2 | Yippee-like 2 | 0 | 44.25 |
| 1373240_at | Dhrs3 | Dehydrogenase/reductase 3 | 0.75 | 29.42 |
| 1372144_at | Dnajb5 | DnaJ heat shock protein family member B5 | 5.25 | 14.92 |
| 1382864_at | Palmd | palmdelphin | 0.75 | 25.29 |
| 1369959_at | Zfp36l1 | zinc finger protein 36, C3H type-like 1 | 0 | 37.01 |
| 1373266_at | Fam107a | family with sequence similarity 107, member A | 0.75 | 41.71 |
| 1376861_at | Mospd1 | Motile sperm domain containing 1 | 3.75 | 32.73 |
| 1371953_at | Ccng2 | cyclin G2 | 3 | 38.34 |
| 1390391_at | Pfkfb3 | 6-phosphofructo-2-kinase/fructose-2,6-biphosphatase 3 | 2.25 | 46.37 |
| 1389003_at | Rhobtb3 | Rho-related BTB domain containing 3 | 3.75 | 39.73 |
| 1374567_at | Zfc3h1 | zinc finger, C3H1-type containing | 3.75 | 45.85 |
| 1374671_at | Traf3ip2 | Traf3 interacting protein 2 | 1.5 | 45.71 |
| 1388583_at | Cxcl12 | C-X-C motif chemokine ligand 12 | 5.25 | 22.03 |
| 1371517_at | Grb10 | growth factor receptor bound protein 10 | 9 | 30.60 |
| 1370570_at | Nrp1 | Neuropilin 1 | 0.75 | 19.16 |
| 1374204_at | Wsb1 | WD repeat and SOCS box-containing 1 | 1.5 | 73.39 |
| 1376763_at | Tet2 | tet methylcytosine dioxygenase 2 | 6.75 | 1.98 |
| 1374984_at | Epb4.1l5 | erythrocyte membrane protein band 4.1 like 5 | 7.5 | 33.17 |
| 1391560_at | Hivep1 | human immunodeficiency virus type I enhancer binding protein 1 | 1.5 | 52.79 |
| 1368347_at | Col5a3 | collagen type V alpha 3 chain | 0 | 50.94 |
| 1398759_at | Tsc22d1 | TSC22 domain family, member 1 | 4.5 | 36.15 |
| 1372835_at | Rhoj | #N/A | 0 | 53.09 |
| 1389064_at | Fem1c | fem-1 homolog C | 4.5 | 2.27 |
| 1386987_at | Il6r | Interleukin 6 receptor | 6.75 | 69.94 |
| 1369976_at | Dynll1 | Dynein, cytoplasmic, light chain 1 | 0.75 | 41.86 |
| 1367869_at | Oxr1 | oxidation resistance 1 | 3 | 63.08 |
| 1369092_at | Sec22a | Sec22 vesicle trafficking protein homolog a | 5.25 | 6.59 |
| 1367602_at | Cited2 | Cbp/p300-interacting transactivator Glu/ Asp-rich carboxy-term Dom 3 | 0 | 76.12 |
| 1373807_at | Vegfa | vascular endothelial growth factor A | 3.75 | 38.09 |
| 1399001_at | Sfr1 | SWI5-dependent homologous recombination repair protein 1 | 0.75 | 58.81 |
| 1398931_at | Tmem223 | transmembrane protein 223 | 8.25 | 5.07 |
| 1368136_at | Tmpo | Thymopoietin | 0 | 54.69 |
| 1398913_at | Numa1 | nuclear mitotic apparatus protein 1 | 3 | 40.36 |
| 1370920_at | Srpk2 | SRSF protein kinase 2 | 5.25 | 9.52 |
| 1389413_at | Evi2a | ecotropic viral integration site 2A | 6 | 64.47 |
| 1398370_at | Adarb1 | adenosine deaminase, RNA-specific, B1 | 2.25 | 40.57 |
| 1370861_at | Cox6a1 | cytochrome c oxidase subunit 6A1 | 7.5 | 46.79 |
| 1371913_at | Tgfbi | transforming growth factor, beta induced | 3 | 39.11 |
| 1367632_at | Glul | Glutamate-ammonia | 5.25 | 67.72 |
| 1388949_at | Nxpe1 | neurexophilin and PC-esterase domain family, member 1 | 4.5 | 3.53 |
| 1387351_at | Fbn1 | fibrillin 1 | 1.5 | 6.27 |
| 1370381_at | Pnrc1 | Proline-rich nuclear receptor coactivator 1, F-box coactivator 1, F-box protein 11 | 1.5 | 32.35 |
| 1399022_at | Clk1 | CDC-like kinase 1 | 2.25 | 65.10 |
| 1388384_at | Rusc2 | Dynein light chain LC8- type 1 | 2.25 | 48.59 |
| 1389570_at | Inpp5k | inositol polyphosphate-5-phosphatase K | 5.25 | 53.23 |
| 1371131_a_at | Txnip | thioredoxin interacting protein | 0.75 | 58.25 |
| 1398819_at | Dnaja1 | DNAJ (Hsp40) homolog, subfamily A, member 1 | 0.75 | 0.36 |
| 1370663_at | Wee1 | WEE1 G2 checkpoint kinase | 0.75 | 28.92 |
| 1373158_at | Gpr146 | G protein-coupled receptor 146 | 3.75 | 23.87 |
| 1368866_at | Ago2 | argonaute 2, RISC catalytic component | 4.5 | 21.26 |
| 1369973_at | Xdh | xanthine dehydrogenase(Xdh) | 0.75 | 41.69 |
| 1388471_at | Tcp11l2 | t-complex 11 like 2 | 1.5 | 48.19 |
| 1388519_at | Sec61b | Sec61 β subunit | 5.25 | 47.68 |
| 1373253_at | Acbd4 | acyl-CoA binding domain containing 4 | 2.25 | 48.90 |
| 1376610_a_at | Ttc28 | tetratricopeptide repeat domain 28 | 2.25 | 43.18 |
| 1375714_at | Erbb2ip | erbb2 interacting protein | 2.25 | 45.44 |
| 1372280_at | Asb2 | Ankyrin repeat and SOCS box-containing protein 2 | 1.5 | 46.52 |
| 1372767_at | Fam168b | family with sequence similarity 168, member B | 0 | 0.12 |
| 1369941_at | Dap | death-associated protein | 1.5 | 36.90 |
| 1386857_at | Stmn1 | stathmin 1 | 8.25 | 29.98 |
| 1376089_at | Ldlr | low density lipoprotein receptor | 3.75 | 64.16 |
| 1370867_at | Gnb2 | G protein subunit beta 2 | 8.25 | 12.82 |
| 1399101_at | LOC100910882 /// Rbm39 | RNA-binding protein 39-like | 3 | 52.05 |
| 1389538_at | Nfkbia | NFKB inhibitor alpha | 1.5 | 49.85 |
| 1398797_at | Hnrnpk | heterogeneous nuclear ribonucleoprotein K-like | 0.75 | 38.85 |
| 1368277_at | Ppp3ca | Protein phosphatase 3, catalytic subunit, alpha isoform, calcineurin A | 4.5 | 68.78 |
| 1367894_at | Insig1 | insulin induced gene 1 | 3.75 | 54.88 |
| 1389065_at | Rbm34 | RNA binding motif protein 34 | 0.75 | 34.16 |
| 1399167_a_at | Gab1 | GRB2-associated binding protein 1 | 0 | 66.96 |
| 1367795_at | Ifrd1 | interferon-related developmental regulator 1 | 0.75 | 76.27 |
| 1367850_at | Fcgr2a | low affinity immunoglobulin gamma Fc region receptor III-like | 2.25 | 84.50 |
| 1367862_at | Rrad | RRAD, Ras related glycolysis inhibitor and calcium channel regulator | 0.75 | 80.23 |
| 1370264_at | Syne1 | spectrin repeat containing nuclear envelope protein 1 | 1.5 | 40.00 |
| 1382099_at | Vps26a | VPS26 retromer complex component A | 3 | 45.58 |
| 1372597_at | Mrpl14 | mitochondrial ribosomal protein L14 | 10.5 | 44.55 |
| 1371029_at | Pkd1 | polycystic kidney disease 1 | 3.75 | 66.10 |
| 1370370_at | Hyal2 | hyaluronoglucosaminidase 2 | 3.75 | 40.58 |
| 1373841_at | Arhgef6 | #N/A | 9.75 | 35.79 |
| 1372426_at | Adamtsl4 | ADAMTS-like 4 | 2.25 | 25.98 |
| 1387053_at | Fmo1 | Flavin-containing monooxygenase 1 | 10.5 | 15.40 |
| 1369182_at | F3 | coagulation factor III, tissue factor | 3.75 | 8.25 |
| 1387181_at | Myf6 | myogenic factor 6 | 0.75 | 82.78 |
| 1374648_at | Gpr155 | G protein-coupled receptor 155 | 6 | 23.44 |
| 1388113_at | Cox8a | Cytochrome c oxidase, subunit VIIIa | 8.25 | 61.13 |
| 1390148_a_at | Zfp395 | zinc finger protein 395 | 1.5 | 65.82 |
| 1374586_at | Arl15 | ADP-ribosylation factor like GTPase 15 | 5.25 | 38.71 |
| 1391062_at | Elp4 | elongator acetyltransferase complex subunit 4 | 1.5 | 15.79 |
| 1388136_at | Timm9 | Translocase of inner mitochondrial membrane 9 | 5.25 | 20.44 |
| 1367643_at | Bsg | basigin | 4.5 | 58.43 |
| 1376805_at | Rnf2 | ring finger protein 2 | 6 | 51.23 |
| 1376587_at | Fbxo11 | F-box protein 11 | 3 | 25.31 |
| 1398365_at | Tppp3 | tubulin polymerization-promoting protein family member 3 | 0 | 30.24 |
| 1370854_at | Nexn | nexilin | 6 | 39.05 |
| 1374425_at | Tle1 | transducin like enhancer of split 1 | 10.5 | 58.08 |
| 1370575_a_at | Azin1 | antizyme inhibitor 1 | 5.25 | 54.57 |
| 1373108_at | Ppp1r3c | protein phosphatase 1, regulatory subunit 3C | 1.5 | 64.71 |
| 1373838_at | Fut4 | fucosyltransferase 4 | 3 | 56.06 |
| 1372341_at | Slc25a36 | solute carrier family 25 member 36 | 4.5 | 72.22 |
| 1367638_at | Mlycd | malonyl-CoA decarboxylase | 9.75 | 59.43 |
| 1371351_at | Rpo1-3 | RNA polymerase 1-3 | 6 | 67.49 |
| 1382223_at | Zmym4 | zinc finger MYM-type containing 4 | 0 | 14.82 |
| 1388819_at | Scamp1 | secretory carrier membrane protein 1 | 0.75 | 30.56 |
| 1369956_at | Ifngr1 | interferon gamma receptor 1 | 3 | 68.63 |
| 1371875_at | Manba | mannosidase beta | 3 | 53.56 |
| 1382059_at | Fbxo30 | F-box protein 30 | 1.5 | 47.13 |
| 1375534_at | Pum2 | Pumilio homolog 2 | 2.25 | 44.40 |
| 1371540_at | Krtcap2 | keratinocyte associated protein 2 | 7.5 | 13.55 |
| 1372651_at | Slc44a1 | #N/A | 1.5 | 40.31 |
| 1374750_at | Fnip1 | Folliculin Interacting Protein 1 | 2.25 | 48.98 |
| 1388924_at | Angptl4 | Anigopoietin-like 4 | 0.75 | 67.40 |
| 1390325_at | Cd38 | CD38 molecule | 11.25 | 10.71 |
| 1377232_at | Limch1 | LIM and calponin homology domains 1 | 0.75 | 60.89 |
| 1376884_a_at | Rpl3l | ribosomal protein L3-like | 2.25 | 92.35 |
| 1374459_at | Alg2 | ALG2, alpha-1,3/1,6-mannosyltransferase | 2.25 | 47.52 |
| 1370252_at | Avpi1 | arginine vasopressin-induced 1 | 0.75 | 34.98 |
| 1375658_at | Sash1 | SAM and SH3 domain containing 1 | 4.5 | 59.78 |
| 1376227_at | Myoz1 | myozenin 1 | 1.5 | 85.01 |
| 1371602_at | Tspan9 | Tetraspanin 9 | 0 | 33.33 |
| 1370399_at | Cyp4b1 | cytochrome P450, family 4, subfamily b, polypeptide 1 | 10.5 | 23.33 |
| 1390253_at | Ankrd9 | ankyrin repeat domain 9 | 1.5 | 51.17 |
| 1372520_at | Mcl1 | BCL2 family apoptosis regulator | 4.5 | 40.04 |
| 1389285_at | Mpp3 | membrane palmitoylated protein 3 | 11.25 | 56.33 |
| 1376015_at | Jph1 | junctophilin 1 | 8.25 | 71.14 |
| 1375037_at | Ppp6r3 | protein phosphatase 6, regulatory subunit 3 | 1.5 | 3.54 |
| 1372045_at | Ap1m1 | adaptor-related protein complex 1, mu 1 subunit | 6.75 | 11.82 |
| 1386885_at | Ech1 | enoyl-CoA hydratase 1 | 10.5 | 13.64 |
| 1387071_a_at | Mapt | microtubule-associated protein tau | 1.5 | 54.64 |
| 1387227_at | Wipf1 | WAS/WASL interacting protein family, member 1 | 3.75 | 28.92 |
| 1371492_at | Apobec2 | apolipoprotein B mRNA editing enzyme catalytic subunit 2 | 3 | 94.34 |
| 1390028_at | Dyrk2 | Dual-specificity tyrosine- (Y)-phosphorylation regulated kinase 2 | 3 | 80.87 |
| 1374872_at | Rasgrp2 | RAS guanyl releasing protein 2 | 11.25 | 14.80 |
| 1387028_a_at | Id1 | inhibitor of DNA binding 1, HLH protein | 0 | 79.73 |
| 1370215_at | C1qb | complement C1q B chain | 0.75 | 8.73 |
| 1370942_at | Rasa3 | RAS p21 protein activator 3 | 7.5 | 89.03 |
| 1377108_at | Mief1 | mitochondrial elongation factor 1 | 4.5 | 48.34 |
| 1372802_at | Zfp512 | zinc finger protein 512 | 10.5 | 33.44 |
| 1387024_at | Dusp6 | dual specificity phosphatase 6 | 9 | 29.39 |
| 1372133_at | Rras2 | related RAS viral (r-ras) oncogene homolog 2 | 3 | 42.89 |
| 1372556_at | Polr2l | DNA-directed RNA polymerases I, II, and III subunit RPABC5-like | 6.75 | 63.87 |
| 1369067_at | Nr4a3 | nuclear receptor subfamily 4, group A, member 3 | 0.75 | 66.33 |
| 1390157_at | Ube2h | ubiquitin-conjugating enzyme E2H(Ube2h) Rattus norvegicus | 4.5 | 64.31 |
| 1369050_at | Pik3c2g | Phosphatidylinositol 3-kinase, C2 domain containing, gamma | 0.75 | 50.20 |
| 1390264_at | Ncbp3 | nuclear cap binding subunit 3 | 2.25 | 55.39 |
| 1370298_at | Coa3 | cytochrome C oxidase assembly factor 3 | 7.5 | 58.91 |
| 1375378_at | Qki | Quaking homolog, KH domain RNA binding | 0.75 | 3.60 |
| 1372750_at | Fst | #N/A | 0.75 | 30.81 |
| 1388672_at | Zcchc24 | zinc finger CCHC-type containing 24 | 3 | 45.72 |
| 1389119_at | Xirp1 | xin actin-binding repeat containing 1 | 4.5 | 3.58 |
| 1369150_at | Pdk4 | pyruvate dehydrogenase kinase 4 | 4.5 | 49.42 |
| 1369590_a_at | Ddit3 | DNA-damage-inducible transcript 3 | 3.75 | 65.48 |
| 1367483_at | Tmem261 | transmembrane protein 261 | 5.25 | 68.40 |
| 1367565_a_at | Fth1 | ferritin heavy chain 1 | 0.75 | 34.09 |
| 1398923_at | Rnasek | ribonuclease K | 3 | 63.79 |
| 1399159_a_at | Vamp3 | vesicle-associated membrane protein 3 | 7.5 | 39.57 |
| 1388710_at | Rreb1 | ras responsive element binding protein 1 | 5.25 | 46.60 |
| 1389088_at | Adnp | activity-dependent neuroprotector homeobox | 5.25 | 45.63 |
| 1376435_at | Loxl4 | Lysyl Oxidase Like 4 | 0.75 | 48.67 |
| 1368063_a_at | Ythdc1 | YTH domain containing 1 | 3 | 59.55 |
| 1367463_at | Phb2 | prohibitin 2 | 7.5 | 48.85 |
| 1368785_a_at | Pitx2 | paired-like homeodomain 2 | 6 | 86.37 |
| 1370523_a_at | Ube2d2 | ubiquitin-conjugating enzyme E2D 2 | 0.75 | 56.11 |
| 1367839_at | Fdft1 | farnesyl diphosphate farnesyl transferase 1 | 10.5 | 0.86 |
| 1371537_at | B4galt5 | beta-1,4-galactosyltransferase 5 | 4.5 | 57.18 |
| 1372990_at | Creb3 | cAMP responsive element binding protein 3 | 12 | 37.70 |
| 1368394_at | Sfrp4 | secreted frizzled-related protein 4 | 3 | 59.48 |
| 1388525_at | Pik3ip1 | phosphoinositide-3-kinase interacting protein 1 | 0.75 | 37.33 |
| 1375984_at | Zfhx4 | zinc finger homeobox 4 | 7.5 | 44.11 |
| 1371754_at | Slc25a25 | solute carrier family 25 member 25 | 3.75 | 80.46 |
| 1367949_at | Penk | proenkephalin | 1.5 | 28.11 |
| 1374602_at | Tspyl1 | TSPY-like 1 | 3.75 | 33.27 |
| 1387244_at | Cgrrf1 | Cgr19 | 2.25 | 43.05 |
| 1374421_at | Baz1b | bromodomain adjacent to zinc finger domain, 1B | 2.25 | 20.33 |
| 1372929_at | Kcnma1 | potassium calcium-activated channel subfamily M alpha 1 | 11.25 | 11.44 |
| 1385236_at | LOC100363361 | zinc finger protein 687-like | 0 | 0.61 |
| 1377190_at | Fam65b | family with sequence similarity 65, member B | 3.75 | 45.35 |
| 1368910_at | Pdp1 | pyruvate dehyrogenase phosphatase catalytic subunit 1 | 10.5 | 47.29 |
| 1374449_at | Cdca3 | cell division cycle associated 3 | 0.75 | 2.65 |
| 1390284_at | Ccdc77 | coiled-coil domain containing 77 | 0.75 | 33.66 |
| 1376569_at | Klf2 | Kruppel-like factor 2 | 0 | 56.11 |
| 1374914_at | Ppard | peroxisome proliferator-activated receptor delta | 3 | 8.10 |
| 1373999_at | Clip1 | #N/A | 6.75 | 41.78 |
| 1398767_at | Ubc | Ubiquitin B, C | 4.5 | 87.69 |
| 1388607_at | Smurf2 | SMAD specific E3 ubiquitin protein ligase 2 | 1.5 | 63.37 |
| 1377275_at | Art1 | ADP-ribosyltransferase 1 | 5.25 | 73.55 |
| 1368004_at | Mrpl23 | Mitochondrial ribosomal protein L23 | 9.75 | 57.54 |
| 1399053_at | Setx | senataxin | 4.5 | 36.09 |
| 1385243_at | Maf | MAF bZIP transcription factor | 6 | 85.04 |
| 1383935_at | Ccdc88c | coiled-coil domain containing 88C | 0 | 60.12 |
| 1376673_at | Zeb1 | zinc finger E-box binding homeobox 1 | 0.75 | 52.34 |
| 1373270_at | Wipi1 | WD repeat domain, phosphoinositide interacting 1 | 1.5 | 12.76 |
| 1368983_at | Hbegf | heparin-binding EGF-like growth factor | 8.25 | 44.62 |
| 1369958_at | Rhob | ras homolog family member B | 0.75 | 58.96 |
| 1372624_at | Ano6 | Unknown | 0.75 | 74.59 |
| 1373818_at | Kctd12 | Potassium channel tetramerization domain containing 12 | 5.25 | 84.13 |
| 1376187_at | Slc35d1 | Solute Carrier Family 35 Member D1 | 9 | 40.69 |
| 1368883_at | Nov | nephroblastoma overexpressed | 3.75 | 26.71 |
| 1398925_at | Cmtr1 | cap methyltransferase 1 | 5.25 | 53.30 |
| 1373465_at | Pqlc1 | PQ loop repeat containing 1 | 8.25 | 29.56 |
| 1377021_at | Trmt6 | tRNA methyltransferase 6 | 2.25 | 44.98 |
| 1398989_at | Son | Son DNA binding protein | 1.5 | 60.09 |
| 1370050_at | Atp2b1 | ATPase plasma membrane Ca2+ transporting 1 | 0.75 | 73.74 |
| 1377334_at | RT1-Ba | RT1 class II, locus Ba | 1.5 | 95.12 |
| 1388301_at | Uqcrc1 | ubiquinol-cytochrome c reductase core protein I | 9 | 40.57 |
| 1367940_at | Ackr3 | atypical chemokine receptor 3 | 2.25 | 47.61 |
| 1377169_at | Traf5 | TNF receptor-associated factor 5 | 1.5 | 69.62 |
| 1371329_at | Eif5a | eukaryotic translation initiation factor 5A | 3.75 | 2.39 |
| 1389449_at | Fitm1 | fat storage-inducing transmembrane protein 1 | 0.75 | 83.65 |
| 1369200_at | Nt5e | 5' nucleotidase, ecto | 1.5 | 45.62 |
| 1398891_at | Mrpl15 | Mitochondrial ribosomal protein L15 | 4.5 | 11.90 |
| 1370172_at | Sod2 | superoxide dismutase 2 | 4.5 | 1.04 |
| 1371976_at | Fam195a | Unknown | 2.25 | 52.43 |
| 1375874_at | Ginm1 | glycoprotein integral membrane 1 | 9.75 | 73.72 |
| 1371955_at | Mrpl35 | mitochondrial ribosomal protein L35 | 7.5 | 6.87 |
| 1388683_at | Naa38 | LSM domain containing 1 | 9 | 54.69 |
| 1399075_at | LOC100910771 /// Map3k7 | mitogen-activated protein kinase kinase kinase 7-like | 0 | 57.89 |
| 1383321_at | Tpst1 | tyrosylprotein sulfotransferase 1 | 1.5 | 61.60 |
| 1389143_at | Pik3ca | phosphatidylinositol-4,5-bisphosphate 3-kinase, catalytic subunit alpha | 3.75 | 57.80 |
| 1370158_at | Myh10 | myosin heavy chain 10 | 5.25 | 63.45 |
| 1389251_at | Nudt7 | Nucleoside diphosphate-linked moiety X motif 7 | 9 | 66.19 |
| 1371324_at | Sf3b5 | Splicing factor 3B | 8.25 | 78.05 |
| 1399077_at | Mtx1 | Metaxin 1 | 7.5 | 32.70 |
| 1388711_at | Il13ra1 /// LOC100360218 | interleukin 13 receptor, alpha 1-like | 0 | 77.49 |
| 1368284_at | Plvap | plasmalemma vesicle associated protein | 2.25 | 58.99 |
| 1389000_at | Kdm3b | lysine demethylase 3B | 4.5 | 26.99 |
| 1373336_at | Gprc5b | G protein-coupled receptor, class C, group 5, member B | 6 | 60.56 |
| 1374006_at | Ccbl2 | kynurenine aminotransferase 3 | 2.25 | 66.54 |
| 1388335_at | Tagln2 | transgelin 2 | 0.75 | 51.20 |
| 1367788_at | Phkg2 | phosphorylase kinase catalytic subunit gamma 2 | 3.75 | 28.15 |
| 1374166_at | Adprhl1 | Unknown | 7.5 | 38.54 |
| 1374674_at | Atxn3 | ataxin 3 | 3.75 | 58.92 |
| 1378423_at | Nmrk2 | Caytaxin | 9 | 33.03 |
| 1367578_at | Prdx2 | peroxiredoxin 2 | 6 | 60.77 |
| 1373748_at | Pdzrn3 | PDZ domain containing RING finger 3 | 3.75 | 5.04 |
| 1369425_at | Cdh13 | cadherin 13 | 6.75 | 16.76 |
| 1375273_at | Trim47 | tripartite motif-containing 47 | 6 | 15.07 |
| 1376927_at | Lrrc14b | leucine rich repeat containing 14B | 6.75 | 47.89 |
| 1387675_at | Plau | plasminogen activator, urokinase | 9 | 64.88 |
| 1376700_at | Lima1 | LIM domain and actin binding 1 | 4.5 | 46.78 |
| 1372347_at | Skil | SKI-like proto-oncogene | 11.25 | 61.07 |
| 1388153_at | Acsl1 | acyl-CoA synthetase long-chain family member 1 | 4.5 | 40.22 |
| 1370166_at | Sdc2 | Syndecan 2 | 5.25 | 66.11 |
| 1399079_at | Prrc2c | proline-rich coiled-coil 2C | 4.5 | 71.36 |
| 1385548_at | Ube2d1 | ubiquitin-conjugating enzyme E2D 1 | 9.75 | 41.88 |
| 1367989_at | Slc2a4 | solute carrier family 2 member 4 | 1.5 | 57.43 |
| 1387321_at | Atp1b4 | ATPase, (Na+)/K+ transporting, beta 4 polypeptide | 3 | 73.36 |
| 1371608_at | Mrps34 | mitochondrial ribosomal protein S34 | 7.5 | 48.13 |
| 1388365_at | Atp6v0d1 | ATPase H+ transporting V0 subunit D1 | 10.5 | 42.65 |
| 1371335_at | Atp5l | ATP synthase, H+ transporting, mitochondrial Fo complex, subunit G | 5.25 | 63.68 |
| 1370428_x_at | RT1-A2 | RT1 class Ib, locus Aw2 | 0 | 96.22 |
| 1389984_at | Jarid2 | jumonji and AT-rich interaction domain containing 2 | 2.25 | 77.36 |
| 1386944_a_at | G6pc | glucose-6-phosphatase, catalytic subunit | 9.75 | 32.03 |
| 1374574_at | Fam214a | family with sequence similarity 214, member A | 5.25 | 13.87 |
| 1368971_a_at | Synj2 | synaptojanin 2 | 7.5 | 72.57 |
| 1375020_at | Rin3 | Ras and Rab interactor 3 | 3 | 41.14 |
| 1398984_at | Tm2d2 | TM2 domain containing 2 | 9 | 13.65 |
| 1389586_at | Ednrb | Endothelin Receptor Type B | 8.25 | 38.46 |
| 1374128_at | Atg7 | autophagy related 7 | 0 | 77.81 |
| 1373723_at | Gpihbp1 | glycosylphosphatidylinositol anchored high density lipoprotein binding protein 1 | 2.25 | 34.10 |
| 1372069_at | Kank1 | KN motif and ankyrin repeat domains 1 | 6 | 84.23 |
| 1372762_at | Hddc3 | HD domain containing 3 | 8.25 | 6.82 |
| 1389309_at | Sbno1 | strawberry notch homolog 1 | 3.75 | 45.92 |
| 1374061_at | Cd302 | CD302 molecule | 2.25 | 61.25 |
| 1371927_at | Ankrd23 | ankyrin repeat domain 23 | 9 | 72.11 |
| 1374904_at | Six1 | SIX homeobox 1 | 6 | 12.29 |
| 1372747_at | Slc20a2 | solute carrier family 20 member 2 | 3.75 | 52.13 |
| 1371263_a_at | Camk2d | calcium/calmodulin-dependent protein kinase II delta | 1.5 | 63.47 |
| 1371041_at | Ndufv2 | NADH:ubiquinone oxidoreductase core subunit V2 | 4.5 | 25.03 |
| 1368552_at | Grpel1 | GrpE-like 1, mitochondrial | 2.25 | 22.68 |
| 1370825_a_at | Cdc42 | cell division cycle 42 | 6 | 36.16 |
| 1373363_at | Map1b | microtubule-associated protein 1B | 3.75 | 68.32 |
| 1376869_at | Tle4 | transducin-like enhancer of split 4 | 3.75 | 74.48 |
| 1375987_at | Cerk | ceramide kinase | 2.25 | 27.80 |
| 1375009_at | Nudt14 | nudix hydrolase 14 | 7.5 | 52.78 |
| 1368379_at | Scarb2 | scavenger receptor class B, member 2 | 0.75 | 43.78 |
| 1389228_at | Fam136a | family with sequence similarity 136, member A | 5.25 | 80.61 |

### Table F: Common genes retaining circadian expression in Adipose and Lung. Δφ indicates their phase difference in hours and ΔΑ their amplitude difference in % change

| **ProbeID** | **Symbol** | **Gene Name** | **Δφ [hr]** | **ΔΑ [%]** |
| --- | --- | --- | --- | --- |
| 1381409_at | --- | --- | 2.25 | 21.56 |
| 1380306_at | --- | --- | 0 | 63.51 |
| 1392613_at | --- | --- | 1.5 | 62.19 |
| 1397225_at | --- | --- | 0.75 | 69.53 |
| 1378174_at | --- | --- | 1.5 | 73.72 |
| 1395178_at | --- | --- | 7.5 | 54.50 |
| 1371864_at | --- | --- | 0 | 14.06 |
| 1378315_at | --- | --- | 2.25 | 26.09 |
| 1377330_at | --- | --- | 2.25 | 28.20 |
| 1389085_at | --- | --- | 1.5 | 0.53 |
| 1393075_at | --- | --- | 0.75 | 32.81 |
| 1385973_at | --- | --- | 0.75 | 36.07 |
| 1398024_at | --- | --- | 0.75 | 57.70 |
| 1376341_at | --- | --- | 0 | 22.64 |
| 1392578_at | --- | --- | 3 | 27.69 |
| 1380777_at | --- | --- | 1.5 | 10.26 |
| 1378225_at | --- | --- | 2.25 | 32.66 |
| 1392675_at | --- | --- | 5.25 | 38.51 |
| 1394574_at | --- | --- | 1.5 | 19.84 |
| 1380365_at | --- | --- | 1.5 | 24.34 |
| 1381553_at | --- | --- | 2.25 | 85.48 |
| 1373225_at | --- | --- | 1.5 | 9.76 |
| 1378292_at | --- | --- | 3 | 9.34 |
| 1395030_at | --- | --- | 0.75 | 6.90 |
| 1376574_at | --- | --- | 0 | 9.31 |
| 1383642_at | --- | --- | 3 | 40.11 |
| 1390441_at | --- | --- | 2.25 | 41.03 |
| 1378098_at | --- | --- | 4.5 | 47.41 |
| 1393119_at | --- | --- | 0.75 | 43.59 |
| 1397453_at | --- | --- | 6.75 | 46.32 |
| 1373736_at | --- | --- | 6 | 37.79 |
| 1382302_at | --- | --- | 1.5 | 59.09 |
| 1374746_at | --- | --- | 6 | 22.40 |
| 1385087_at | --- | --- | 7.5 | 51.53 |
| 1375689_at | --- | --- | 7.5 | 73.26 |
| 1398635_at | --- | --- | 1.5 | 24.64 |
| AFFX-BioDn-3_at | --- | --- | 0 | 29.65 |
| 1392412_at | --- | --- | 10.5 | 6.26 |
| 1377725_at | --- | --- | 0 | 4.27 |
| 1393597_at | --- | --- | 3.75 | 29.46 |
| 1397554_at | --- | --- | 6 | 31.84 |
| 1391561_at | --- | --- | 9 | 52.63 |
| 1383919_at | --- | --- | 2.25 | 45.32 |
| 1392549_at | --- | --- | 0.75 | 16.15 |
| 1378998_at | --- | --- | 3 | 12.16 |
| 1397138_at | --- | --- | 0.75 | 46.92 |
| 1397589_at | --- | --- | 3 | 0.70 |
| 1392627_x_at | --- | --- | 6 | 47.32 |
| 1394467_at | --- | --- | 7.5 | 39.39 |
| 1381019_x_at | --- | --- | 3.75 | 12.53 |
| 1392024_at | --- | --- | 9.75 | 47.22 |
| 1393898_at | --- | --- | 0 | 19.06 |
| 1378814_at | --- | --- | 0 | 31.86 |
| AFFX-r2-Ec-bioD-3_at | --- | --- | 0 | 28.85 |
| 1389999_at | --- | --- | 3 | 2.24 |
| 1398213_at | --- | --- | 1.5 | 13.43 |
| 1378268_at | --- | --- | 8.25 | 9.26 |
| 1379915_at | --- | --- | 6.75 | 44.66 |
| 1397676_at | --- | --- | 4.5 | 70.63 |
| 1372481_at | --- | --- | 7.5 | 5.56 |
| 1384372_at | --- | --- | 7.5 | 51.26 |
| 1389986_at | --- | --- | 9 | 73.35 |
| 1391553_at | --- | --- | 6.75 | 39.40 |
| 1390743_at | --- | --- | 0 | 33.20 |
| 1376363_at | --- | --- | 0 | 28.03 |
| 1376096_a_at | --- | --- | 9.75 | 59.34 |
| 1382976_at | --- | --- | 7.5 | 43.49 |
| 1397286_at | --- | --- | 7.5 | 52.90 |
| 1397164_at | --- | --- | 6.75 | 29.24 |
| 1390277_at | --- | --- | 5.25 | 24.40 |
| 1380780_at | --- | --- | 0.75 | 16.04 |
| 1379799_at | --- | --- | 6 | 16.67 |
| 1372309_at | --- | --- | 1.5 | 6.02 |
| AFFX-r2-Ec-bioD-5_at | --- | --- | 0.75 | 24.12 |
| 1383170_at | --- | --- | 9 | 15.61 |
| 1372874_at | --- | --- | 0.75 | 16.27 |
| 1374696_at | --- | --- | 7.5 | 35.21 |
| 1395859_at | --- | --- | 3.75 | 42.91 |
| 1399082_at | --- | --- | 2.25 | 34.27 |
| 1383518_at | --- | --- | 0.75 | 34.34 |
| 1394427_at | --- | --- | 8.25 | 1.72 |
| 1376848_at | --- | --- | 4.5 | 35.71 |
| 1377589_at | --- | --- | 1.5 | 18.89 |
| AFFX-BioDn-5_at | --- | --- | 0.75 | 23.83 |
| 1379716_at | --- | --- | 1.5 | 26.08 |
| 1372648_at | --- | --- | 2.25 | 5.17 |
| 1379280_at | --- | --- | 0.75 | 38.78 |
| 1397153_at | --- | --- | 0 | 28.19 |
| 1374307_at | --- | --- | 8.25 | 18.61 |
| 1397766_at | --- | --- | 8.25 | 45.29 |
| 1390276_at | --- | --- | 8.25 | 24.92 |
| 1381496_x_at | --- | --- | 2.25 | 32.13 |
| 1394585_at | --- | --- | 9 | 20.74 |
| 1372081_at | --- | --- | 3.75 | 17.76 |
| 1393333_at | --- | --- | 9.75 | 26.59 |
| 1374687_at | --- | --- | 6.75 | 11.38 |
| 1372573_at | --- | --- | 9 | 19.49 |
| 1373488_at | --- | --- | 1.5 | 34.69 |
| 1394910_at | --- | --- | 1.5 | 32.05 |
| 1379509_at | --- | --- | 7.5 | 77.44 |
| 1385723_at | --- | --- | 0 | 81.89 |
| 1383063_a_at | --- | --- | 2.25 | 61.16 |
| 1392316_at | --- | --- | 0.75 | 19.25 |
| 1370986_s_at | --- | --- | 9.75 | 10.77 |
| 1380041_at | --- | --- | 8.25 | 62.38 |
| 1374702_at | --- | --- | 8.25 | 53.33 |
| 1372104_at | --- | --- | 4.5 | 53.41 |
| 1381157_at | --- | --- | 6.75 | 27.03 |
| 1385630_at | --- | --- | 7.5 | 25.45 |
| 1378038_at | --- | --- | 0.75 | 42.83 |
| 1380701_at | --- | --- | 10.5 | 52.44 |
| 1384003_at | --- | --- | 8.25 | 56.22 |
| 1376534_at | --- | --- | 0 | 17.83 |
| 1391674_at | --- | --- | 1.5 | 82.95 |
| 1382423_at | --- | --- | 0.75 | 34.48 |
| 1380011_at | --- | --- | 1.5 | 21.61 |
| 1380464_at | --- | --- | 10.5 | 39.71 |
| 1377298_at | --- | --- | 5.25 | 15.88 |
| 1381535_at | --- | --- | 12 | 9.44 |
| 1372136_at | --- | --- | 3 | 28.48 |
| 1397017_at | --- | --- | 4.5 | 40.06 |
| 1381066_at | --- | --- | 8.25 | 55.28 |
| 1391148_at | --- | --- | 8.25 | 7.63 |
| 1379797_at | --- | --- | 8.25 | 45.43 |
| 1397406_at | --- | --- | 2.25 | 32.61 |
| 1375868_at | --- | --- | 2.25 | 30.46 |
| 1394880_at | --- | --- | 9.75 | 28.35 |
| 1396417_at | --- | --- | 1.5 | 36.82 |
| 1383598_at | --- | --- | 1.5 | 27.92 |
| 1395303_at | --- | --- | 6 | 39.03 |
| 1386185_at | --- | --- | 10.5 | 20.53 |
| 1383862_at | --- | --- | 4.5 | 5.79 |
| 1385149_at | --- | --- | 1.5 | 49.34 |
| 1383744_at | --- | --- | 0 | 30.00 |
| 1394578_at | --- | --- | 3 | 19.97 |
| 1373285_at | --- | --- | 9 | 48.15 |
| 1382281_at | --- | --- | 0.75 | 67.47 |
| 1382142_at | --- | --- | 0.75 | 13.17 |
| 1382510_at | --- | --- | 0.75 | 27.45 |
| 1394932_at | --- | --- | 0.75 | 40.18 |
| 1394478_at | --- | --- | 7.5 | 44.67 |
| 1380590_at | --- | --- | 2.25 | 55.69 |
| 1396583_at | --- | --- | 8.25 | 50.55 |
| 1372949_at | --- | --- | 6.75 | 40.33 |
| 1397889_at | --- | --- | 6.75 | 62.00 |
| 1396855_at | --- | --- | 6.75 | 63.95 |
| 1391061_at | --- | --- | 7.5 | 78.04 |
| 1395728_at | --- | --- | 3.75 | 71.97 |
| 1388479_at | --- | --- | 4.5 | 45.69 |
| 1376840_at | --- | --- | 11.25 | 48.45 |
| 1377747_at | --- | --- | 9 | 67.14 |
| 1398083_at | --- | --- | 3 | 6.03 |
| 1377964_at | --- | --- | 3.75 | 44.67 |
| 1377445_at | --- | --- | 9.75 | 30.36 |
| 1376747_at | --- | --- | 8.25 | 41.95 |
| 1386566_at | --- | --- | 2.25 | 20.89 |
| 1386051_at | --- | --- | 6.75 | 69.88 |
| 1392304_at | --- | --- | 0.75 | 44.76 |
| 1384597_at | --- | --- | 11.25 | 46.76 |
| 1395857_at | --- | --- | 1.5 | 22.31 |
| 1382377_at | --- | --- | 0.75 | 22.90 |
| 1398345_at | --- | --- | 5.25 | 38.26 |
| 1374263_at | --- | --- | 5.25 | 33.44 |
| 1377862_at | --- | --- | 2.25 | 18.65 |
| 1381100_at | --- | --- | 10.5 | 65.71 |
| 1394047_at | --- | --- | 1.5 | 63.30 |
| 1395446_at | --- | --- | 1.5 | 61.62 |
| 1383557_at | --- | --- | 6.75 | 23.92 |
| 1396815_at | --- | --- | 2.25 | 9.21 |
| 1379410_at | --- | --- | 1.5 | 5.26 |
| 1377500_at | --- | --- | 7.5 | 76.88 |
| 1384042_at | --- | --- | 3 | 63.58 |
| 1393094_at | --- | --- | 6.75 | 46.73 |
| 1391431_at | --- | --- | 2.25 | 68.97 |
| 1377715_at | --- | --- | 9 | 57.56 |
| 1392716_at | --- | --- | 2.25 | 86.42 |
| 1391444_at | --- | --- | 2.25 | 20.64 |
| 1373676_at | --- | --- | 6 | 41.36 |
| 1389412_at | --- | --- | 3.75 | 45.04 |
| 1377800_at | --- | --- | 10.5 | 50.42 |
| 1377671_at | --- | --- | 0.75 | 46.45 |
| 1378281_at | --- | --- | 3 | 76.83 |
| 1386186_s_at | --- | --- | 10.5 | 4.40 |
| 1392483_at | --- | --- | 7.5 | 59.22 |
| 1377705_at | --- | --- | 1.5 | 52.20 |
| 1390430_at | Nr1d2 | nuclear receptor subfamily 1, group D, member 2 | 0 | 20.53 |
| 1383439_at | Npas2 | neuronal PAS domain protein 2 | 0.75 | 35.52 |
| 1382031_at | Fmo2 | flavin containing monooxygenase 2 | 3 | 41.10 |
| 1378745_at | Per3 | period circadian clock 3 | 0 | 19.56 |
| 1367771_at | Tsc22d3 | Glucocorticoid-induced leucine zipper | 2.25 | 21.37 |
| 1368303_at | Per2 | period circadian clock 2 | 0.75 | 4.40 |
| 1368304_at | Fmo3 | flavin containing monooxygenase 3 | 0 | 52.46 |
| 1370816_at | Nr1d1 | nuclear receptor subfamily 1, group D, member 1 | 0 | 40.26 |
| 1386641_at | Hlf | HLF, PAR bZIP transcription factor | 2.25 | 50.03 |
| 1370510_a_at | Arntl | aryl hydrocarbon receptor nuclear translocator-like | 0 | 15.77 |
| 1370847_at | Spon2 | spondin 2 | 1.5 | 58.08 |
| 1395753_at | Eln | Elastin | 3 | 80.06 |
| 1367982_at | Alas1 | Aminolevulinic acid synthase 1 | 11.25 | 63.76 |
| 1389836_a_at | Timp3 | TIMP metallopeptidase inhibitor 4 | 3 | 0.90 |
| 1369919_at | Tef | TEF, PAR bZIP transcription factor | 3 | 13.80 |
| 1370209_at | Klf9 | Kruppel-like factor 9 | 0.75 | 4.93 |
| 1383641_at | Ednra | Endothelin receptor type a | 8.25 | 60.71 |
| 1373114_at | Dtx4 | deltex E3 ubiquitin ligase 4 | 1.5 | 24.05 |
| 1387874_at | Dbp | D-box binding PAR bZIP transcription factor | 0 | 60.90 |
| 1367741_at | Herpud1 | homocysteine inducible ER protein with ubiquitin like domain 1 | 0 | 36.92 |
| 1376523_at | Arid4a | At rich interactive domain 4a | 4.5 | 45.94 |
| 1397409_s_at | Wee1 | WEE1 G2 checkpoint kinase | 0.75 | 45.55 |
| 1368249_at | Klf15 | Kruppel-like factor 15 | 0.75 | 22.82 |
| 1367631_at | Ctgf | connective tissue growth factor | 1.5 | 27.19 |
| 1378156_at | Cys1 | cystin 1 | 0 | 9.60 |
| 1393696_at | Fibin | fin bud initiation factor homolog (zebrafish) | 5.25 | 47.10 |
| 1391187_at | Ppl | Periplakin | 0.75 | 57.93 |
| 1388901_at | Fkbp5 | FK506 binding protein 5 | 1.5 | 8.56 |
| 1371583_at | Rbm3 | RNA binding motif (RNP1, RRM) protein 3 | 2.25 | 9.33 |
| 1370283_at | Hspa5 | heat shock protein family A member 5 | 0 | 30.78 |
| 1374575_at | Creb3l1 | cAMP responsive element binding protein 3-like 1 | 1.5 | 33.08 |
| 1368778_at | Slc6a6 | solute carrier family 6 member 6 | 3.75 | 38.34 |
| 1368488_at | Nfil3 | nuclear factor, interleukin 3 regulated | 0 | 13.46 |
| 1390199_at | Clock | clock circadian regulator | 0.75 | 30.97 |
| 1367922_at | Adam17 | ADAM metallopeptidase domain 17 | 3.75 | 36.47 |
| 1393730_at | Adamts4 | ADAM metallopeptidase with thrombospondin type 1 motif, 4 | 1.5 | 25.13 |
| 1392149_at | LOC102557354 | uncharacterized LOC102557354 | 0.75 | 67.11 |
| 1371832_at | Leo1 | LEO1 homolog, Paf1/RNA polymerase II complex component | 0 | 19.03 |
| 1393508_at | Nox4 | Nadph oxidase 4 | 7.5 | 22.51 |
| 1392203_at | Hoxb2 | Homeobox b2 | 3 | 19.87 |
| 1374855_at | Per1 | period circadian clock 1 | 0 | 54.40 |
| 1386097_at | Ankrd12 | ankyrin repeat domain 12 | 4.5 | 47.52 |
| 1393632_at | C1qtnf7 | C1q & tumor necrosis factor related protein 7 | 2.25 | 3.99 |
| 1374650_at | Nedd9 | neural precursor cell expressed, developmentally down-regulated 9 | 3 | 23.73 |
| 1392510_at | Fam180a | family with sequence similarity 180, member A | 2.25 | 2.24 |
| 1369943_at | Tgm2 | Transglutaminase 2 | 0 | 23.59 |
| 1398431_at | Car8 | Carbonic anhydrase viii | 3 | 6.76 |
| 1395986_at | Slit2 | Slit homolog 2 | 4.5 | 34.93 |
| 1381811_at | Usp2 | Ubiquitin Specific Peptidase 2 | 0.75 | 45.88 |
| 1367759_at | H1f0 | H1 histone family, member 0 | 2.25 | 18.95 |
| 1398750_at | Calr | calreticulin | 0 | 8.39 |
| 1393610_at | Fam76a | family with sequence similarity 76, member A | 3.75 | 31.31 |
| 1368486_at | Irs3 | insulin receptor substrate 3 | 0.75 | 0.75 |
| 1382199_at | Zcchc14 | Zinc finger, cchc domain containing 14 | 3.75 | 51.79 |
| 1369636_at | Sord | sorbitol dehydrogenase | 3 | 32.74 |
| 1398597_at | Rnf144a | ring finger protein 144A | 1.5 | 21.60 |
| 1373195_at | Fus | Fused in sarcoma | 1.5 | 35.41 |
| 1378168_at | Fam101b | Family with sequence similarity 101, b | 3.75 | 41.89 |
| 1369484_at | Wisp2 | Wnt1 inducible signaling pathway protein 2 | 10.5 | 14.60 |
| 1387795_at | Pola2 | Polymerase , α 2 | 6 | 26.73 |
| 1388686_at | Rcan1 | Regulator of calcineurin 1 | 0 | 23.70 |
| 1375908_at | Mpzl2 | myelin protein zero-like 2 | 0.75 | 57.19 |
| 1372213_at | LOC500300 | -- | 3 | 41.41 |
| 1368247_at | Hspa1a | heat shock 70kD protein 1A | 1.5 | 5.19 |
| 1388176_at | Cml5 | Camello-like 5 | 0 | 47.29 |
| 1372308_at | St3gal1 | ST3 beta-galactoside alpha-2,3-sialyltransferase 1 | 3.75 | 18.84 |
| 1387294_at | Sh3bp5 | SH3-domain binding protein 5 | 3.75 | 53.15 |
| 1392640_at | Cry1 | cryptochrome circadian clock 1 | 1.5 | 26.33 |
| 1389341_at | Plxnd1 | Plexin d1 | 1.5 | 27.95 |
| 1389355_at | Ier5 | immediate early response 5 | 2.25 | 5.83 |
| 1383137_at | Sox4 | SRY box 4 | 0.75 | 1.11 |
| 1388898_at | Hsph1 | heat shock protein family H (Hsp110) member 1 | 2.25 | 10.82 |
| 1378482_at | Mdfic | Myod family inhibitor domain containing | 2.25 | 29.52 |
| 1379719_at | Syde2 | Synapse Defective Rho GTPase Homolog 2 | 2.25 | 32.11 |
| 1373093_at | Errfi1 | ERBB receptor feedback inhibitor 1 | 2.25 | 62.57 |
| 1375336_at | Hsp90ab1 | Heat shock 90-kDa protein 1, beta | 1.5 | 65.04 |
| 1376770_at | Efhd1 | EF-hand domain family, member D1 | 2.25 | 48.29 |
| 1373087_at | March7 | membrane associated ring-CH-type finger 7 | 0 | 15.91 |
| 1370912_at | Hspa1a | Heat shock 70kD protein 1B | 2.25 | 26.33 |
| 1370954_at | P4ha1 | prolyl 4-hydroxylase subunit alpha 1 | 1.5 | 39.29 |
| 1370991_at | Cml3 | probable N-acetyltransferase | 0 | 20.76 |
| 1371505_at | Hnrnpc | heterogeneous nuclear ribonucleoproteins C1/C2-like | 1.5 | 41.22 |
| 1378552_at | Map3k6 | mitogen-activated protein kinase kinase kinase 6 | 0 | 7.54 |
| 1384285_at | March3 | Membrane-associated ring finger 3 | 1.5 | 47.41 |
| 1391808_at | Arrdc4 | arrestin domain containing 4 | 3.75 | 1.45 |
| 1382809_at | Cirbp | cold inducible RNA binding protein | 2.25 | 37.86 |
| 1368144_at | Rgs2 | Regulator of g-protein signaling 2, 24kda | 9 | 6.49 |
| 1388140_at | Rab13 | RAB13, member RAS oncogene family | 3.75 | 31.65 |
| 1383300_at | Klhl24 | kelch-like family member 24 | 6.75 | 9.12 |
| 1372701_at | Hsp90aa1 | heat shock protein 1, alpha-like | 4.5 | 48.21 |
| 1378544_at | Rbbp6 | Retinoblastoma binding protein 6 | 8.25 | 34.01 |
| 1391217_at | Zc3h12c | Zinc Finger CCCH-Type Containing 12C | 7.5 | 66.80 |
| 1393634_at | Nrg4 | neuregulin 4 | 3 | 41.74 |
| 1377287_at | Mars2 | methionine--tRNA ligase, mitochondrial-like | 0 | 31.89 |
| 1377163_at | Inhbb | Inhibin, β b | 12 | 65.29 |
| 1388271_at | Mt2A | metallothionein 2A | 3 | 15.43 |
| 1390107_at | Sytl2 | Synaptotagmin-like 2 | 4.5 | 36.61 |
| 1398240_at | Hspa8 | Heat shock protein 70 | 2.25 | 8.81 |
| 1371237_a_at | Mt1a | metallothionein 1 | 0.75 | 56.25 |
| 1374349_at | Ctdspl | Ctd small phosphatase-like | 0.75 | 41.38 |
| 1373147_at | Fbxl3 | F-box and leucine-rich repeat protein 3 | 0.75 | 6.94 |
| 1376911_at | Atp2b4 | ATPase plasma membrane Ca2+ transporting 4 | 7.5 | 18.58 |
| 1377369_at | Cybrd1 | Cytochrome b reductase 1 | 6 | 31.08 |
| 1373092_at | Tgfbr3 | transforming growth factor beta receptor 3 | 2.25 | 33.67 |
| 1395557_at | Klf6 | Kruppel-like factor 6 | 6 | 35.55 |
| 1393615_at | Deptor | DEP domain containing MTOR-interacting protein | 7.5 | 52.97 |
| 1377148_at | Xrn1 | 5'-3' Exoribonuclease 1 | 6.75 | 12.65 |
| 1378666_at | Arhgap21 | Rho gtpase activating protein 21 | 6.75 | 26.32 |
| 1376909_at | Rasl10a | Ras-like, family 10, a | 4.5 | 31.24 |
| 1371931_at | Gtf2i | general transcription factor II I | 9.75 | 13.47 |
| 1395157_at | Nedd4 | neural precursor cell expressed, developmentally down-regulated 4, E3 ubiquitin protein ligase | 6.75 | 6.84 |
| 1398662_at | Fam167a | family with sequence similarity 167, member A | 6.75 | 49.01 |
| 1372878_at | Zfr | zinc finger RNA binding protein | 3 | 25.46 |
| 1389651_at | Apln | Apelin | 1.5 | 3.33 |
| 1367726_at | Thra | Thyroid hormone receptor, α | 0.75 | 52.55 |
| 1373542_at | Sphk2 | sphingosine kinase 2 | 0.75 | 36.13 |
| 1395058_at | Wdr75 | Wd repeat domain 75 | 9 | 45.07 |
| 1385108_at | Fam126b | Family with sequence similarity 126, b | 6.75 | 25.09 |
| 1368511_at | Bhlhe41 | Basic helix-loop-helix domain containing, class B, 3 | 0 | 60.52 |
| 1373403_at | LOC100910163 | Chromosome 8 open reading frame 4 | 3 | 39.44 |
| 1373866_at | Coq10b | coenzyme Q10B | 0 | 6.60 |
| 1368200_at | Cx3cl1 | C-X3-C motif chemokine ligand 1 | 1.5 | 20.57 |
| 1372390_at | Peg3 | paternally expressed 3 | 4.5 | 53.87 |
| 1389587_at | Umps | uridine monophosphate synthetase | 1.5 | 14.29 |
| 1373027_at | Mllt4 | afadin, adherens junction formation factor | 4.5 | 18.88 |
| 1382831_at | Dusp18 | dual specificity phosphatase 18 | 7.5 | 38.82 |
| 1388145_at | Tnxb | tenascin XB | 0.75 | 39.69 |
| 1389432_at | Pbx2 | PBX homeobox 2 | 6 | 49.23 |
| 1395826_at | Ppp2r5e | Protein phosphatase 2, regulatory subunit b', ε | 5.25 | 5.86 |
| 1381923_at | RGD1564664 | -- | 3.75 | 3.01 |
| 1393198_at | Pcmtd1 | protein-L-isoaspartate O-methyltransferase domain containing 1 | 9 | 51.62 |
| 1390042_at | Tmem140 | Transmembrane Protein 140 | 3.75 | 14.29 |
| 1370336_at | Osgin1 | oxidative stress induced growth inhibitor 1 | 4.5 | 37.45 |
| 1398877_at | Stip1 | stress-induced phosphoprotein 1 | 1.5 | 6.89 |
| 1390383_at | Plin2 | perilipin 2 | 0 | 40.46 |
| 1379477_at | LOC100910424 | uncharacterized LOC100910424 | 0.75 | 27.56 |
| 1368080_at | Rgcc | regulator of cell cycle | 3 | 11.72 |
| 1373816_at | Ap1g1 | adaptor-related protein complex 1, gamma 1 subunit | 7.5 | 13.29 |
| 1368571_at | Clip2 | CAP-GLY domain containing linker protein 2 | 0 | 3.90 |
| 1373399_at | Wdr6 | WD repeat domain 6 | 0.75 | 18.11 |
| 1376645_at | Medag | mesenteric estrogen-dependent adipogenesis | 3 | 25.10 |
| 1389199_at | RGD1309079 | similar to Ab2-095 | 3.75 | 53.83 |
| 1391625_at | Wasl | Wiskott-Aldrich syndrome-like | 8.25 | 59.23 |
| 1392490_at | Tmem57 | Transmembrane protein 57 | 1.5 | 26.20 |
| 1393917_at | Cd163 | CD163 molecule | 0.75 | 9.94 |
| 1397627_at | Diaph1 | diaphanous-related formin 1 | 9.75 | 36.50 |
| 1392633_at | Fbxo32 | F-Box Protein 32 | 6.75 | 32.60 |
| 1395413_at | Lrp6 | LDL Receptor Related Protein 6 | 6 | 29.69 |
| 1390141_at | Mthfd1l | Methylenetetrahydrofolate dehydrogenase 1-like | 1.5 | 38.23 |
| 1373303_at | Scaf11 | Splicing factor, arginine/serine-rich 2, interacting protein | 8.25 | 41.25 |
| 1377016_at | Creld2 | cysteine-rich with EGF-like domains 2 | 1.5 | 1.69 |
| 1368991_at | Smpd3 | Sphingomyelin phosphodiesterase 3, neutral membrane | 2.25 | 37.86 |
| 1367668_a_at | Scd2 | acyl-CoA desaturase 2-like | 0.75 | 20.60 |
| 1384217_at | Zhx2 | zinc fingers and homeoboxes 2 | 3 | 23.94 |
| 1389844_at | Fkbp4 | FK506 binding protein 4 | 0 | 12.79 |
| 1384355_at | Plxna2 | Plexin a2 | 11.25 | 12.08 |
| 1379971_at | Zc3h6 | Zinc finger CCCH type containing 6 | 1.5 | 39.97 |
| 1368538_at | Exoc7 | Exocyst complex component 7 | 4.5 | 3.60 |
| 1379669_at | Arhgap42 | Rho gtpase activating protein 42 | 3.75 | 5.72 |
| 1397634_at | Anks1a | Ankyrin repeat & sterile α motif domain containing 1a | 1.5 | 10.66 |
| 1386770_x_at | Kcne2 | Potassium voltage-gated channel, isk-related family, 2 | 6 | 25.12 |
| 1370372_at | Rasd2 | RASD family, member 2 | 0 | 11.91 |
| 1383099_at | Gpbp1 | Gc-rich promoter binding protein 1 | 6.75 | 55.64 |
| 1368882_at | St6galnac3 | ST6 N-acetylgalactosaminide alpha-2,6-sialyltransferase 3 | 0 | 33.01 |
| 1368177_at | Acsl3 | acyl-CoA synthetase long-chain family member 3 | 3.75 | 70.00 |
| 1385229_at | Pcdh20 | protocadherin 20 | 2.25 | 31.90 |
| 1369754_a_at | Cast | Calpastatin | 9.75 | 42.42 |
| 1374480_at | Daam1 | dishevelled associated activator of morphogenesis 1 | 4.5 | 39.20 |
| 1375590_at | Zc3h11a | zinc finger CCCH-type containing 11A | 6.75 | 39.61 |
| 1379627_at | Tm9sf3 | Transmembrane 9 superfamily 3 | 5.25 | 13.21 |
| 1383169_at | LOC103691479 | Leukemia inhibitory factor receptor ? | 2.25 | 8.26 |
| 1368025_at | Ddit4 | DNA-damage-inducible transcript 4 | 2.25 | 23.59 |
| 1391549_at | Dcun1d4 | defective in cullin neddylation 1 domain containing 4 | 6 | 38.64 |
| 1392587_at | Pik3r1 | Phosphoinositide-3-Kinase Regulatory Subunit 1 | 1.5 | 40.37 |
| 1388795_at | Tppp | tubulin polymerization promoting protein | 1.5 | 2.05 |
| 1398522_at | Ankle2 | Ankyrin repeat & lem domain containing 2 | 7.5 | 48.42 |
| 1369063_at | Anp32a | acidic nuclear phosphoprotein 32 family member A | 4.5 | 35.10 |
| 1372301_at | Aebp1 | AE binding protein 1 | 3.75 | 44.75 |
| 1378362_at | C1ql3 | complement C1q like 3 | 6.75 | 30.99 |
| 1389787_at | Ptk7 | protein tyrosine kinase 7 | 6 | 64.23 |
| 1383494_at | Srbd1 | S1 rna binding domain 1 | 5.25 | 25.87 |
| 1372367_at | Arhgap35 | Rho GTPase Activating Protein 35 | 8.25 | 30.41 |
| 1377608_a_at | Tardbp | TAR DNA binding protein | 4.5 | 18.56 |
| 1368223_at | Adamts1 | Adam metallopeptidase with thrombospondin 1 motif, 15 | 8.25 | 7.14 |
| 1373407_at | Gramd4 | GRAM domain containing 4 | 0.75 | 18.88 |
| 1390145_at | Dmxl2 | Dmx-like 2 | 5.25 | 3.12 |
| 1368549_at | Hbp1 | HMG-box transcription factor 1 | 0.75 | 38.20 |
| 1395264_at | Rif1 | replication timing regulatory factor 1 | 8.25 | 64.78 |
| 1378111_at | Kcnd3 | potassium voltage-gated channel subfamily D member 3 | 3 | 12.89 |
| 1394706_at | Pank3 | pantothenate kinase 3 | 6 | 31.55 |
| 1385006_at | Atrx | ATRX, chromatin remodeler | 8.25 | 48.88 |
| 1395100_at | Pbrm1 | polybromo 1 | 7.5 | 6.52 |
| 1383004_at | Ahcyl1 | S adenosylhomocysteine hydrolase like 1 | 7.5 | 32.60 |
| 1382068_at | Tulp4 | Tubby Like Protein 4 | 7.5 | 64.40 |
| 1372346_at | Zfp513 | Zinc finger protein 513 | 0 | 14.37 |
| 1375396_at | Pum1 | pumilio RNA-binding family member 1 | 7.5 | 5.55 |
| 1367568_a_at | Mgp | matrix Gla protein | 0.75 | 42.34 |
| 1376762_at | Plekha1 | Pleckstrin homology domain containing, family a 1 | 8.25 | 45.95 |
| 1398255_at | Slc15a2 | solute carrier family 15 member 2 | 9.75 | 60.18 |
| 1373566_at | Irf2bp2 | Interferon Regulatory Factor 2 Binding Protein 2 | 4.5 | 31.55 |
| 1375916_at | Pcmtd2 | protein-L-isoaspartate O-methyltransferase domain containing 2 | 0.75 | 52.41 |
| 1371929_at | Mlx | MLX, MAX dimerization protein | 3 | 36.82 |
| 1375898_at | Rbpms | RNA Binding Protein With Multiple Splicing | 3 | 50.21 |
| 1369526_at | Acadsb | acyl-CoA dehydrogenase, short/branched chain | 7.5 | 31.66 |
| 1387669_a_at | Ephx1 | Epoxide hydrolase 1, | 0 | 44.76 |
| 1395246_at | Prkar2a | Protein Kinase CAMP-Dependent Type II Regulatory Subunit Alpha | 8.25 | 13.38 |
| 1398998_at | RGD1309748 | -- | 0 | 41.10 |
| 1383091_at | Appbp2 | amyloid beta precursor protein binding protein 2 | 6.75 | 0.00 |
| 1387873_at | Wfdc1 | WAP four-disulfide core domain 1 | 6.75 | 18.45 |
| 1380695_at | Zyg11b | zyg-11 family member B, cell cycle regulator | 6.75 | 37.22 |
| 1384854_at | RGD1566359 | similar to RIKEN cDNA B230219D22 | 7.5 | 18.17 |
| 1382524_at | Zbtb20 | zinc finger and BTB domain containing 20 | 7.5 | 43.49 |
| 1382847_at | Ash1l | ASH1 like histone lysine methyltransferase | 6 | 30.11 |
| 1380371_at | Nipbl | NIPBL, cohesin loading factor | 8.25 | 80.37 |
| 1398729_s_at | Nom1 | nucleolar protein with MIF4G domain 1 | 7.5 | 31.01 |
| 1373240_at | Dhrs3 | Dehydrogenase/reductase 3 | 3 | 22.44 |
| 1380242_at | Lrp5 | LDL receptor related protein 5 | 1.5 | 1.14 |
| 1384916_at | Stk35 | serine/threonine kinase 35 | 3 | 15.64 |
| 1376208_at | Dlg2 | Discs, large homolog 2 | 10.5 | 29.61 |
| 1374568_at | Cipc | CLOCK-interacting pacemaker | 2.25 | 9.55 |
| 1368173_at | Nop58 | NOP58 ribonucleoprotein | 7.5 | 52.36 |
| 1381886_at | Yy1 | YY1 transcription factor | 7.5 | 33.78 |
| 1396078_at | Krit1 | KRIT1, ankyrin repeat containing | 7.5 | 14.64 |
| 1373055_at | Tbcel | tubulin folding cofactor E-like | 0 | 5.51 |
| 1370807_at | Vmp1 | vacuole membrane protein 1 | 4.5 | 28.29 |
| 1391757_at | Ptpn4 | protein tyrosine phosphatase, non-receptor type 4 | 6.75 | 61.06 |
| 1382266_at | Gpr146 | G protein-coupled receptor 146 | 4.5 | 60.94 |
| 1382206_a_at | Akap2 | A-kinase anchoring protein 2 | 9 | 27.45 |
| 1392553_at | Adamtsl5 | Adamts-like 5 | 9 | 6.85 |
| 1393813_at | Ttc3 | Tetratricopeptide repeat domain 3 | 8.25 | 11.70 |
| 1368021_at | Adh1 | alcohol dehydrogenase 1 | 2.25 | 69.77 |
| 1383827_at | Tlk1 | tousled-like kinase 1 | 7.5 | 57.51 |
| 1370927_at | Col12a1 | Collagen, type xii, α 1 | 5.25 | 23.39 |
| 1392111_at | Exosc3 | Exosome component 3 | 1.5 | 17.74 |
| 1385852_at | Crebbp | CREB binding protein | 8.25 | 34.06 |
| 1380363_at | Klf7 | Kruppel like factor 7 | 8.25 | 70.62 |
| 1391838_at | Ankrd11 | ankyrin repeat domain 11 | 7.5 | 70.37 |
| 1375168_at | Hdac7 | Histone Deacetylase 7 | 3.75 | 21.00 |
| 1384110_at | Dock4 | dedicator of cytokinesis 4 | 8.25 | 66.31 |
| 1375692_at | Mapk1 | mitogen-activated protein kinase 1 | 4.5 | 15.36 |
| 1377848_at | Fam175a | family with sequence similarity 175, member A | 4.5 | 5.47 |
| 1373266_at | Fam107a | family with sequence similarity 107, member A | 0.75 | 46.70 |
| 1398553_at | Tmed5 | Transmembrane emp24 protein transport domain containing 5 | 7.5 | 43.15 |
| 1394566_at | Fam204a | family with sequence similarity 204, member A | 7.5 | 27.38 |
| 1390391_at | Pfkfb3 | 6-phosphofructo-2-kinase/fructose-2,6-biphosphatase 3 | 0.75 | 25.04 |
| 1390638_at | Epha4 | Eph receptor a4 | 5.25 | 25.09 |
| 1379693_at | Robo2 | roundabout guidance receptor 2 | 1.5 | 31.42 |
| 1373267_at | Sh3yl1 | SH3 and SYLF domain containing 1 | 6.75 | 23.64 |
| 1394436_at | Spag9 | sperm associated antigen 9 | 7.5 | 49.01 |
| 1368393_at | Cd93 | CD93 molecule | 1.5 | 29.78 |
| 1381244_at | Cux1 | Cut Like Homeobox 1 | 8.25 | 64.42 |
| 1374671_at | Traf3ip2 | Traf3 interacting protein 2 | 3 | 4.84 |
| 1395485_s_at | Myo1b | myosin Ib | 9 | 58.25 |
| 1388583_at | Cxcl12 | C-X-C motif chemokine ligand 12 | 3 | 15.14 |
| 1383263_at | Ogn | Osteoglycin | 6 | 18.63 |
| 1370259_a_at | Pth1r | parathyroid hormone 1 receptor | 3.75 | 46.92 |
| 1382939_at | Tpr | translocated promoter region, nuclear basket protein | 8.25 | 82.43 |
| 1381611_at | Ahctf1 | At hook containing transcription factor 1 | 1.5 | 1.23 |
| 1370570_at | Nrp1 | Neuropilin 1 | 0.75 | 39.91 |
| 1398327_at | Fermt2 | fermitin family member 2 | 3.75 | 35.04 |
| 1380079_at | LOC102553788 | sterile alpha motif domain-containing protein 9-like | 9 | 9.75 |
| 1371693_at | Ahsa1 | Activator of heat shock 90-kDa protein ATPase homolog 1 | 1.5 | 18.64 |
| 1383374_at | Galnt1 | Polypeptide N-Acetylgalactosaminyltransferase 1 | 1.5 | 12.03 |
| 1376596_at | Ddx42 | Dead box 42 | 6 | 36.26 |
| 1370266_at | Parva | Parvin, alpha | 11.25 | 20.56 |
| 1379232_at | Tbc1d12 | TBC1 domain family member 12-like | 7.5 | 68.07 |
| 1389534_at | Ube2e3 | ubiquitin-conjugating enzyme E2E 3 | 10.5 | 9.75 |
| 1382165_at | Smndc1 | survival motor neuron domain containing 1 | 9 | 25.13 |
| 1371614_at | Atg12 | Autophagy related 12 homolog | 0 | 28.16 |
| 1378074_at | Pdk4 | pyruvate dehydrogenase kinase 4 | 1.5 | 48.44 |
| 1391560_at | Hivep1 | human immunodeficiency virus type I enhancer binding protein 1 | 7.5 | 37.75 |
| 1385228_x_at | Asap1 | #N/A | 3.75 | 44.55 |
| 1391759_at | Sh3glb1 | SH3 domain -containing GRB2-like endophilin B1 | 6.75 | 40.29 |
| 1392746_x_at | Larp1 | La ribonucleoprotein domain family, member 1 | 0.75 | 10.69 |
| 1379982_at | Nrip1 | Nuclear receptor interacting protein 1 | 0 | 51.90 |
| 1382330_at | LOC103695291 | ArfGAP with SH3 domain, ankyrin repeat and PH domain 2 | 6 | 3.94 |
| 1372835_at | Rhoj | #N/A | 2.25 | 10.38 |
| 1398886_at | RGD1563348 | -- | 2.25 | 6.98 |
| 1383844_at | Arhgef15 | Rho guanine nucleotide exchange factor 15 | 2.25 | 4.41 |
| 1386987_at | Il6r | Interleukin 6 receptor | 3.75 | 18.91 |
| 1370286_at | Slc38a2 | solute carrier family 38, member 2 | 1.5 | 47.91 |
| 1373972_at | Nav1 | neuron navigator 1 | 7.5 | 59.28 |
| 1370097_a_at | Cxcr4 | C-X-C motif chemokine receptor 4 | 3 | 11.22 |
| 1374189_at | Zfp219 | Zinc finger protein 219 | 3.75 | 22.22 |
| 1376889_at | Gpr153 | #N/A | 3.75 | 5.25 |
| 1373807_at | Vegfa | vascular endothelial growth factor A | 2.25 | 6.71 |
| 1390662_at | Trim24 | tripartite motif-containing 24 | 9 | 24.73 |
| 1395298_at | Ubp1 | upstream binding protein 1 (LBP-1a) | 8.25 | 9.09 |
| 1379772_at | Aplnr | apelin receptor | 1.5 | 6.33 |
| 1383017_at | Ptprm | protein tyrosine phosphatase, receptor type, M | 6 | 17.59 |
| 1387374_at | Tcf12 | transcription factor 12 | 9 | 29.75 |
| 1370310_at | Hmgcs2 | 3-hydroxy-3-methylglutaryl-CoA synthase 2 | 3.75 | 52.77 |
| 1383013_at | Klf13 | Kruppel-like factor 13 | 0 | 50.32 |
| 1395074_at | Glt8d2 | Thymine-dna glycosylase | 0.75 | 10.61 |
| 1367539_at | Gtf2a1 | General Transcription Factor IIA Subunit 1 | 2.25 | 15.35 |
| 1377992_at | Dusp7 | dual specificity phosphatase 7 | 5.25 | 51.07 |
| 1380503_at | Fam193a | family with sequence similarity 193, member A | 9.75 | 41.03 |
| 1371684_at | Pelo | pelota mRNA surveillance and ribosome rescue factor | 10.5 | 16.53 |
| 1375444_at | Ap3d1 | adaptor-related protein complex 3, delta 1 subunit | 9 | 59.53 |
| 1382368_at | Trove2 | TROVE domain family, member 2 | 9 | 24.32 |
| 1383730_at | Ttc9c | tetratricopeptide repeat domain 9C | 3 | 24.27 |
| 1374537_at | Chsy1 | chondroitin sulfate synthase 1 | 3 | 53.67 |
| 1384728_at | Foxn3 | forkhead box N3 | 9 | 25.05 |
| 1389089_at | Slc39a7 | Solute carrier family 39 , 7 | 1.5 | 36.69 |
| 1393029_at | Celf1 | CUGBP Elav-Like Family Member 1 | 7.5 | 32.72 |
| 1373629_at | Slc7a6 | solute carrier family 7 member 6 | 1.5 | 59.44 |
| 1388932_at | Lama5 | laminin subunit alpha 5 | 9 | 43.90 |
| 1374974_at | Zak | sterile alpha motif and leucine zipper containing kinase AZK | 9 | 35.08 |
| 1387186_at | Rab9a | RAB9A, member RAS oncogene family | 6 | 59.31 |
| 1373708_at | Tut1 | Terminal uridylyl transferase 1 | 0.75 | 59.03 |
| 1369775_at | Nucks1 | nuclear ubiquitous casein and cyclin-dependent kinases substrate-like | 7.5 | 71.31 |
| 1398370_at | Adarb1 | adenosine deaminase, RNA-specific, B1 | 1.5 | 73.02 |
| 1381829_at | Zfp318 | zinc finger protein 318 | 7.5 | 57.57 |
| 1378569_at | Pcdh12 | Protocadherin 12 | 0.75 | 6.55 |
| 1379555_at | Xiap | X-linked inhibitor of apoptosis | 6.75 | 69.02 |
| 1393596_at | Atrx | ATRX, chromatin remodeler | 8.25 | 31.66 |
| 1394626_at | Hmbox1 | homeobox containing 1 | 7.5 | 49.93 |
| 1371913_at | Tgfbi | transforming growth factor, beta induced | 2.25 | 66.29 |
| 1389616_at | Whsc1 | Wolf-Hirschhorn syndrome candidate 1 | 4.5 | 33.39 |
| 1370932_at | Lrp4 | LDL receptor related protein 4 | 8.25 | 48.42 |
| 1367632_at | Glul | Glutamate-ammonia | 4.5 | 0.56 |
| 1375367_at | Pdlim2 | PDZ and LIM domain 2 | 0 | 42.12 |
| 1393368_at | Osbpl5 | Oxysterol binding protein-like 5 | 6.75 | 38.31 |
| 1388949_at | Nxpe1 | neurexophilin and PC-esterase domain family, member 1 | 3 | 28.83 |
| 1388722_at | Dnajb1 | DnaJ heat shock protein family member B1 | 0.75 | 40.22 |
| 1374232_at | Pik3ca | phosphatidylinositol-4,5-bisphosphate 3-kinase, catalytic subunit alpha | 6.75 | 47.93 |
| 1391315_at | Fnip2 | Folliculin Interacting Protein 2 | 6.75 | 36.58 |
| 1390340_a_at | Eif4g1 | eukaryotic translation initiation factor 4 gamma, 1 | 9.75 | 51.04 |
| 1374085_at | Mxd4 | Max dimerization protein 4 | 5.25 | 42.35 |
| 1375473_at | Flt1 | |  | Fms Related Tyrosine Kinase 1 | | --- | --- | | 10.5 | 41.05 |
| 1398788_at | Pdia3 | protein disulfide isomerase family A, member 3 | 5.25 | 63.18 |
| 1370381_at | Pnrc1 | Proline-rich nuclear receptor coactivator 1, F-box coactivator 1, F-box protein 11 | 0.75 | 29.45 |
| 1376917_at | Zfp292 | zinc finger protein 292 | 9 | 79.85 |
| 1370955_at | Adam10 | ADAM metallopeptidase domain 10 | 9.75 | 30.72 |
| 1375669_at | Fkbp2 | FK506 binding protein 2 | 6.75 | 46.59 |
| 1399022_at | Clk1 | CDC-like kinase 1 | 1.5 | 55.80 |
| 1383946_at | Cldn1 | claudin 1 | 3 | 51.55 |
| 1381650_at | Sephs1 | selenophosphate synthetase 1 | 6.75 | 49.32 |
| 1388384_at | Rusc2 | Dynein light chain LC8- type 1 | 0 | 68.63 |
| 1388920_at | Bmp6 | bone morphogenetic protein 6 | 8.25 | 30.52 |
| 1380726_at | Aspn | Asporin | 3 | 46.82 |
| 1375492_at | Sulf1 | sulfatase 1 | 7.5 | 80.74 |
| 1394316_a_at | Tspan5 | tetraspanin 5 | 9 | 35.01 |
| 1371887_at | Hmgb3 | high mobility group protein B3-like | 4.5 | 12.36 |
| 1373718_at | Tubb2a | tubulin, beta 2A class 2a | 3 | 78.99 |
| 1397200_at | Chd4 | chromodomain helicase DNA binding protein 4 | 8.25 | 46.64 |
| 1383867_at | Eif5a2 | eukaryotic translation initiation factor 5A2 | 0.75 | 12.20 |
| 1369177_at | Pi4k2a | phosphatidylinositol 4-kinase type 2 alpha | 9 | 47.50 |
| 1385076_at | Zbed6 | Zinc Finger BED-Type Containing 6 | 9 | 46.41 |
| 1392739_a_at | Eepd1 | Endonuclease/exonuclease family domain 1 | 6.75 | 31.08 |
| 1372530_at | Hcfc1 | host cell factor C1 | 7.5 | 21.43 |
| 1371131_a_at | Txnip | thioredoxin interacting protein | 4.5 | 0.56 |
| 1373759_at | Fosb | FosB proto-oncogene, AP-1 transcription factor subunit | 5.25 | 11.73 |
| 1399070_at | Setd5 | SET domain containing 5 | 6.75 | 19.97 |
| 1392519_at | Nktr | natural killer cell triggering receptor | 8.25 | 49.84 |
| 1387769_a_at | Id3 | inhibitor of DNA binding 3, HLH protein | 3 | 23.03 |
| 1375892_at | Elavl1 | ELAV like RNA binding protein 1 | 5.25 | 22.66 |
| 1384759_at | Jade1 | jade family PHD finger 1 | 6 | 59.96 |
| 1370141_at | Mcl1 | BCL2 family apoptosis regulator | 2.25 | 28.83 |
| 1369312_a_at | Csnk1a1 | casein kinase 1, alpha 1 | 7.5 | 15.87 |
| 1373824_at | Cfdp1 | Flavin | 9 | 9.02 |
| 1385077_at | Gbf1 | golgi brefeldin A resistant guanine nucleotide exchange factor 1 | 7.5 | 17.50 |
| 1382103_at | Pgm3 | phosphoglucomutase 3 | 9.75 | 60.85 |
| 1398819_at | Dnaja1 | DNAJ (Hsp40) homolog, subfamily A, member 1 | 0 | 28.02 |
| 1391094_at | Rnft1 | ring finger protein, transmembrane 1 | 8.25 | 43.59 |
| 1395142_at | RGD1561931 | -- | 3.75 | 64.15 |
| 1391303_at | Purb | purine rich element binding protein B | 8.25 | 84.31 |
| 1389433_at | Mkks | McKusick-Kaufman syndrome | 0.75 | 37.48 |
| 1380810_at | Zfp40 | Zinc finger protein 40 | 1.5 | 14.92 |
| 1374945_at | Trmt61a | tRNA methyltransferase 61A | 6.75 | 17.78 |
| 1395297_at | RGD1309621 | similar to hypothetical protein FLJ10652 | 6.75 | 54.16 |
| 1368703_at | Pdlim5 | PDZ and LIM domain 5 | 9 | 42.29 |
| 1381683_at | Chd2 | chromodomain helicase DNA binding protein 2 | 6.75 | 47.90 |
| 1368189_at | Dhcr7 | 7 dehydrocholesterol reductase | 0 | 52.33 |
| 1392648_at | Mrc1 | Mannose receptor, c type 1 | 0.75 | 35.75 |
| 1382721_at | Ggnbp2 | gametogenetin binding protein 2 | 3.75 | 32.42 |
| 1379469_at | Tbl1x | transducin -like 1 X-linked | 7.5 | 94.12 |
| 1397812_at | Senp6 | SUMO1/sentrin specific peptidase 6 | 7.5 | 11.92 |
| 1373014_at | B3gat3 | beta-1,3-glucuronyltransferase 3 | 0.75 | 20.43 |
| 1374947_at | Bcar3 | breast cancer anti-estrogen resistance 3 | 0 | 55.18 |
| 1370963_at | Gas7 | Growth arrest specific 7 | 6 | 58.03 |
| 1374868_at | Zfhx3 | zinc finger homeobox 3 | 8.25 | 45.30 |
| 1379885_at | Fmo4 | flavin containing monooxygenase 4 | 2.25 | 25.98 |
| 1384818_at | Mylk | myosin light chain kinase | 9 | 5.03 |
| 1372999_at | Dcun1d5 | defective in cullin neddylation 1 domain containing 5 | 9.75 | 10.78 |
| 1389468_at | Rpia | ribose 5-phosphate isomerase A | 3 | 53.29 |
| 1379704_at | Zfp143 | zinc finger protein 143 | 4.5 | 21.36 |
| 1386615_at | Chst2 | carbohydrate sulfotransferase 2 | 6.75 | 27.06 |
| 1398400_at | Strada | STE20-related kinase adaptor alpha | 6 | 42.72 |
| 1372365_at | Rin2 | Ras and Rab interactor 2 | 9.75 | 70.66 |
| 1381193_at | Lpgat1 | lysophosphatidylglycerol acyltransferase 1 | 5.25 | 50.66 |
| 1385430_at | Lims1 | LIM zinc finger domain containing 1 | 9 | 44.07 |
| 1391968_at | Pdxdc1 | pyridoxal-dependent decarboxylase domain containing 1 | 8.25 | 33.42 |
| 1375080_at | Slc25a45 | solute carrier family 25, member 45 | 3.75 | 11.14 |
| 1377124_at | Mtf1 | metal regulatory transcription factor 1-like | 4.5 | 37.13 |
| 1373031_at | Trim8 | tripartite motif-containing 8 | 0.75 | 26.09 |
| 1368116_a_at | Rps6kb1 | ribosomal protein S6 kinase B1 | 7.5 | 27.34 |
| 1367831_at | Tp53 | tumor protein p53 | 3 | 29.76 |
| 1383597_at | Dclre1c | Dna cross-link repair 1c | 5.25 | 15.92 |
| 1398273_at | Efna1 | Ephrin A1 | 3.75 | 13.48 |
| 1382045_at | Tbc1d15 | TBC1 domain family, member 15 | 8.25 | 22.41 |
| 1371885_at | Tbcb | tubulin folding cofactor B | 5.25 | 48.24 |
| 1384056_at | Fgfr3 | fibroblast growth factor receptor 3 | 9 | 3.56 |
| 1384106_at | Sgms1 | Sphingomyelin synthase 1 | 2.25 | 15.66 |
| 1374133_at | Trit1 | tRNA isopentenyltransferase 1 | 9.75 | 42.48 |
| 1385382_at | Adam19 | ADAM Metallopeptidase Domain 19 | 9.75 | 66.84 |
| 1389538_at | Nfkbia | NFKB inhibitor alpha | 0 | 18.04 |
| 1396192_at | Npat | nuclear protein, co-activator of histone transcription | 6 | 35.10 |
| 1379482_at | Tm6sf1 | Transmembrane 6 superfamily 1 | 1.5 | 17.69 |
| 1377474_at | Abhd13 | abhydrolase domain containing 13 | 0.75 | 6.57 |
| 1382192_at | Lyve1 | Lymphatic Vessel Endothelial Hyaluronan Receptor 1 | 3.75 | 69.12 |
| 1377392_at | Daam2 | dishevelled associated activator of morphogenesis 2 | 3 | 5.97 |
| 1382029_at | Fam222b | family with sequence similarity 222, member B | 10.5 | 41.55 |
| 1394724_at | Tcea1 | transcription elongation factor A (SII) 1 | 7.5 | 1.01 |
| 1379910_at | Uap1l2 | UDP-N-acteylglucosamine pyrophosphorylase 1-like 2 | 2.25 | 21.68 |
| 1375574_at | Gga3 | golgi associated, gamma adaptin ear containing, ARF binding protein 3 | 5.25 | 13.03 |
| 1396803_at | Thoc2 | THO complex 2 | 6.75 | 60.84 |
| 1371985_a_at | Abhd16a | abhydrolase domain containing 16A | 7.5 | 44.46 |
| 1393692_at | Pcf11 | PCF11 cleavage and polyadenylation factor subunit | 9.75 | 63.21 |
| 1388453_at | Myadm | myeloid-associated differentiation marker | 3 | 46.46 |
| 1367850_at | Fcgr2a | low affinity immunoglobulin gamma Fc region receptor III-like | 3 | 83.92 |
| 1376906_at | Med1 | mediator complex subunit 1 | 8.25 | 52.94 |
| 1370948_a_at | Marcks | #N/A | 3 | 27.91 |
| 1389036_at | Pisd | phosphatidylserine decarboxylase | 3 | 48.54 |
| 1390459_at | Adgrg6 | adhesion G protein-coupled receptor G6 | 10.5 | 13.67 |
| 1372872_at | Zzz3 | zinc finger, ZZ-type containing 3 | 1.5 | 27.27 |
| 1389037_at | Rit1 | Ras-like without CAAX 1 | 5.25 | 25.51 |
| 1385502_at | Trim21 | Tripartite Motif Containing 21 | 9 | 36.17 |
| 1369617_at | Ube2n | ubiquitin-conjugating enzyme E2N | 3.75 | 15.70 |
| 1377772_at | Tmeff1 | transmembrane protein with EGF-like and two follistatin-like domains 1 | 3.75 | 65.45 |
| 1392961_at | Mob1a | MOB kinase activator 1A | 8.25 | 68.00 |
| 1377262_at | Ppp4r3b | protein phosphatase 4, regulatory subunit 3B | 8.25 | 36.45 |
| 1382717_at | Fam168a | family with sequence similarity 168, member A | 6.75 | 65.64 |
| 1395146_at | Adipor2 | adiponectin receptor 2 | 2.25 | 53.97 |
| 1398960_at | Cct6a | chaperonin containing TCP1 subunit 6A | 2.25 | 69.39 |
| 1388447_at | Lbh | limb bud and heart development | 3 | 20.50 |
| 1383531_at | Crebrf | CREB3 regulatory factor | 1.5 | 31.46 |
| 1371956_at | Pja1 | praja ring finger ubiquitin ligase 1 | 3.75 | 7.00 |
| 1373239_at | Snx33 | Sorting nexin 33 | 1.5 | 53.31 |
| 1372719_at | Susd6 | sushi domain containing 6 | 5.25 | 5.81 |
| 1376924_a_at | Palmd | palmdelphin | 7.5 | 20.56 |
| 1383160_at | Chordc1 | Cysteine and histidine-rich domain (CHORD)-containing, zinc-bp1 | 1.5 | 38.26 |
| 1370445_at | Pla1a | Phosphatidylserine specific phospholipase A1 | 3.75 | 17.84 |
| 1395377_at | Bfar | Bifunctional apoptosis regulator | 5.25 | 61.38 |
| 1371029_at | Pkd1 | polycystic kidney disease 1 | 6.75 | 48.36 |
| 1397392_at | LOC102550580 | uncharacterized LOC102550580 | 2.25 | 42.81 |
| 1382846_at | Tab3 | TGF-beta activated kinase 1/MAP3K7 binding protein 3 | 2.25 | 28.63 |
| 1375622_at | Rbsn | rabenosyn, RAB effector | 10.5 | 59.62 |
| 1391297_at | Rcor1 | REST corepressor 1 | 8.25 | 76.00 |
| 1377632_at | Timp4 | Timp metallopeptidase inhibitor 4 | 0 | 14.93 |
| 1390263_at | Gnas | GNAS complex locus | 7.5 | 46.78 |
| 1372426_at | Adamtsl4 | ADAMTS-like 4 | 4.5 | 34.29 |
| 1397824_at | Wac | WW domain containing adaptor with coiled-coil | 6 | 35.98 |
| 1383533_at | LOC102551140 | REST corepressor 3 | 0.75 | 26.87 |
| 1393822_at | Tmcc3 | Transmembrane And Coiled-Coil Domain Family 3 | 9 | 31.15 |
| 1379615_at | Klhl42 | kelch-like family, member 42 | 8.25 | 32.17 |
| 1379912_at | Cnot6l | CCR4-NOT Transcription Complex Subunit 6 Like | 3 | 30.09 |
| 1373482_at | Traf3 | Tnf receptor-associated factor 3 | 3 | 40.38 |
| 1367772_at | Clns1a | chloride nucleotide-sensitive channel 1A | 7.5 | 6.58 |
| 1395053_at | Pds5b | PDS5 cohesin associated factor B | 8.25 | 11.26 |
| 1369182_at | F3 | coagulation factor III, tissue factor | 4.5 | 47.45 |
| 1383382_at | Jarid2 | jumonji and AT-rich interaction domain containing 2 | 6.75 | 19.41 |
| 1373302_at | Acer2 | alkaline ceramidase 2 | 4.5 | 67.05 |
| 1388002_at | Taok1 | TAO kinase 1 | 9 | 15.65 |
| 1378193_at | Ms4a7 | membrane spanning 4-domains A7 | 0.75 | 24.53 |
| 1374104_at | Cldn5 | claudin 5 | 9 | 68.08 |
| 1369192_at | Cdkn1b | cyclin-dependent kinase inhibitor 1B | 8.25 | 11.01 |
| 1393811_at | Ercc6l2 | ERCC excision repair 6 like 2 | 9 | 59.48 |
| 1381551_at | Alkbh1 | alkB homolog 1, histone H2A dioxygenase | 8.25 | 50.30 |
| 1390148_a_at | Zfp395 | zinc finger protein 395 | 0.75 | 41.29 |
| 1374172_at | Col8a2 | #N/A | 10.5 | 13.20 |
| 1369994_at | Crcp | CGRP receptor component | 0 | 36.62 |
| 1386959_a_at | Map2k5 | mitogen activated protein kinase kinase 5 | 8.25 | 14.97 |
| 1395535_at | Fam98a | family with sequence similarity 98, member A | 12 | 29.66 |
| 1391062_at | Elp4 | elongator acetyltransferase complex subunit 4 | 1.5 | 11.25 |
| 1373347_at | Acbd3 | acyl-CoA binding domain containing 3 | 8.25 | 83.51 |
| 1370969_at | Hoxa5 | homeo box A5 | 4.5 | 13.99 |
| 1394838_at | Aoc3 | amine oxidase, copper containing 3 | 6.75 | 14.37 |
| 1394207_at | Slc16a10 | #N/A | 8.25 | 6.19 |
| 1382967_at | Adgrg2 | adhesion G protein-coupled receptor G2 | 0 | 12.82 |
| 1398365_at | Tppp3 | tubulin polymerization-promoting protein family member 3 | 1.5 | 68.93 |
| 1398430_at | Myo6 | myosin VI | 0.75 | 33.63 |
| 1390381_at | Xpc | Xeroderma pigmentosum, | 8.25 | 61.01 |
| 1368519_at | Serpine1 | serpin family E member 1 | 1.5 | 65.76 |
| 1372618_at | Rabl6 | RAB, member RAS oncogene family-like 6 | 2.25 | 14.71 |
| 1369644_at | Adgrl2 | adhesion G protein-coupled receptor L2 | 4.5 | 26.09 |
| 1369261_at | Kcnj13 | potassium voltage-gated channel subfamily J member 13 | 9.75 | 24.60 |
| 1397708_at | LOC288978 | hypothetical LOC288978 | 1.5 | 10.03 |
| 1372341_at | Slc25a36 | solute carrier family 25 member 36 | 0.75 | 47.98 |
| 1367638_at | Mlycd | malonyl-CoA decarboxylase | 6.75 | 57.10 |
| 1370112_at | Pten | phosphatase and tensin homolog | 11.25 | 55.88 |
| 1391689_at | Kdm5a | lysine demethylase 5A | 6.75 | 75.20 |
| 1389114_at | Thnsl2 | threonine synthase-like 2 | 1.5 | 9.62 |
| 1372827_at | Ppid | peptidylprolyl isomerase D | 1.5 | 61.64 |
| 1371715_at | Sptssa | serine palmitoyltransferase, small subunit A | 5.25 | 24.39 |
| 1369956_at | Ifngr1 | interferon gamma receptor 1 | 3 | 59.31 |
| 1394565_at | Copz1 | coatomer protein complex, subunit zeta 1 | 0.75 | 4.60 |
| 1392051_at | Tmem87b | transmembrane protein 87B | 5.25 | 70.68 |
| 1388142_at | Vcan | versican | 12 | 24.92 |
| 1398339_at | Sf1 | splicing factor 1 | 6 | 13.78 |
| 1374599_at | Herc1 | HECT And RLD Domain Containing E3 Ubiquitin Protein Ligase Family Member 1 | 10.5 | 36.98 |
| 1375553_at | Spata24 | spermatogenesis associated 24 | 4.5 | 37.80 |
| 1382568_at | Rhbdd1 | rhomboid domain containing 1 | 2.25 | 50.71 |
| 1392854_at | Ddx6 | DEAD-box helicase 6 | 2.25 | 25.14 |
| 1377018_at | Pamr1 | peptidase domain containing associated with muscle regeneration 1 | 9 | 11.94 |
| 1374045_at | Cog8 | Component of oligomeric golgi complex 8 | 1.5 | 34.90 |
| 1379689_at | Rc3h2 | ring finger and CCCH-type domains 2 | 7.5 | 40.26 |
| 1393473_at | Colca2 | colorectal cancer associated 2 | 5.25 | 66.33 |
| 1382699_s_at | Hps1 | HPS1, biogenesis of lysosomal organelles complex 3 subunit 1 | 3.75 | 1.99 |
| 1387810_at | Keap1 | Kelch-like ECH-associated protein 1 | 7.5 | 3.62 |
| 1388924_at | Angptl4 | Anigopoietin-like 4 | 1.5 | 52.06 |
| 1367932_at | Hmgcs1 | 3-hydroxy-3-methylglutaryl-CoA synthase 1 | 5.25 | 38.98 |
| 1375047_at | Spata5 | spermatogenesis associated 5 | 9 | 38.35 |
| 1389778_a_at | Tceb3 | transcription elongation factor B subunit 3 | 1.5 | 32.83 |
| 1394682_at | Aff4 | AF4/FMR2 family, member 4 | 9.75 | 53.61 |
| 1367826_at | Nfe2l2 | nuclear factor, erythroid 2-like 2 | 0.75 | 32.78 |
| 1370989_at | Ret | ret proto-oncogene | 1.5 | 0.74 |
| 1375658_at | Sash1 | SAM and SH3 domain containing 1 | 3 | 46.92 |
| 1389700_at | Rasa2 | #N/A | 4.5 | 64.70 |
| 1374491_at | Cmtm8 | CKLF-like MARVEL transmembrane domain containing 8 | 1.5 | 30.40 |
| 1385350_at | Igip | IgA Inducing Protein | 10.5 | 64.36 |
| 1379226_at | Aim1l | absent in melanoma 1-like | 9 | 5.30 |
| 1379384_at | Sp1 | Sp1 transcription factor | 9 | 71.93 |
| 1370399_at | Cyp4b1 | cytochrome P450, family 4, subfamily b, polypeptide 1 | 11.25 | 50.50 |
| 1389782_at | RGD1305587 | -- | 3.75 | 59.40 |
| 1386721_at | Zfp503 | zinc finger protein 503 | 9.75 | 57.91 |
| 1375037_at | Ppp6r3 | protein phosphatase 6, regulatory subunit 3 | 3 | 3.67 |
| 1383215_at | Ttc28 | tetratricopeptide repeat domain 28 | 8.25 | 11.29 |
| 1377577_at | Gmps | guanine monophosphate synthase | 12 | 32.64 |
| 1373998_at | Specc1 | sperm antigen with calponin homology and coiled-coil domains 1 | 1.5 | 62.30 |
| 1378171_at | Nrp2 | Neuropilin 2 | 10.5 | 28.57 |
| 1377624_at | Gtpbp10 | GTP binding protein 10 | 6 | 26.97 |
| 1383328_x_at | Pdcd4 | programmed cell death 4 | 2.25 | 37.60 |
| 1373919_at | Cisd2 | CDGSH iron sulfur domain 2 | 3.75 | 13.00 |
| 1371403_at | Cct3 | Chaperonin subunit 3 (gamma) | 1.5 | 23.46 |
| 1390828_at | Npy1r | neuropeptide Y receptor Y1 | 3 | 26.92 |
| 1387786_at | Mtpn | myotrophin | 6.75 | 19.07 |
| 1370215_at | C1qb | complement C1q B chain | 2.25 | 0.82 |
| 1374558_at | Icoslg | inducible T-cell co-stimulator ligand | 3 | 30.56 |
| 1393427_s_at | Egfl7 | EGF-like-domain, multiple 7 | 3 | 12.18 |
| 1385486_at | Bnc2 | basonuclin 2 | 10.5 | 47.08 |
| 1376728_at | Rbm47 | RNA binding motif protein 47 | 3 | 52.96 |
| 1393033_at | Yars2 | tyrosyl-tRNA synthetase 2 | 5.25 | 21.69 |
| 1387805_at | Bnip3 | BCL2/adenovirus E1B 19 kDa- | 1.5 | 5.17 |
| 1382481_a_at | Adam33 | ADAM metallopeptidase domain 33 | 0.75 | 59.69 |
| 1367857_at | Fads1 | Fatty acid desaturase 1 | 3 | 70.22 |
| 1380336_at | Irak3 | interleukin-1 receptor-associated kinase 3 | 0.75 | 32.03 |
| 1387024_at | Dusp6 | dual specificity phosphatase 6 | 6 | 6.88 |
| 1399127_at | Ermard | ER membrane-associated RNA degradation | 4.5 | 34.85 |
| 1392592_at | Tcf7l2 | transcription factor 7 like 2 | 1.5 | 54.13 |
| 1376177_at | Fam117a | family with sequence similarity 117, member A | 0.75 | 65.66 |
| 1392918_at | Cct8 | chaperonin containing TCP1 subunit 8 | 3 | 61.45 |
| 1379218_at | Nlk | Nemo Like Kinase | 11.25 | 15.43 |
| 1384294_at | Cmklr1 | chemerin chemokine-like receptor 1 | 0 | 36.88 |
| 1393959_at | Sbno1 | Strawberry Notch Homolog 1 | 9.75 | 17.34 |
| 1367946_at | Pdlim1 | PDZ and LIM domain 1 | 3.75 | 25.00 |
| 1372629_at | Coro2b | coronin 2B | 0.75 | 24.04 |
| 1393758_at | Ttc14 | tetratricopeptide repeat domain 14 | 7.5 | 48.01 |
| 1392763_at | Agpat1 | 1-acylglycerol-3-phosphate O-acyltransferase 1 | 2.25 | 34.72 |
| 1374932_at | Angptl8 | angiopoietin-like 8 | 2.25 | 29.38 |
| 1371613_at | Elmo2 | engulfment and cell motility 2 | 0.75 | 32.48 |
| 1368648_at | Cox4i2 | cytochrome c oxidase subunit 4i2 | 0.75 | 41.49 |
| 1389681_at | Pvrl2 | Poliovirus receptor-related 2 | 10.5 | 18.85 |
| 1379678_at | Hmgxb4 | HMG-box containing 4 | 8.25 | 75.45 |
| 1376106_at | Tmem178a | transmembrane protein 178A | 0 | 80.22 |
| 1372256_at | Crip1 | cysteine rich protein 1 | 3.75 | 41.09 |
| 1372750_at | Fst | |  | Follistatin | | --- | --- | | 0.75 | 66.98 |
| 1388672_at | Zcchc24 | zinc finger CCHC-type containing 24 | 1.5 | 58.93 |
| 1385089_at | Grhl1 | grainyhead-like transcription factor 1 | 9.75 | 58.37 |
| 1371839_at | Srsf2 | serine and arginine rich splicing factor 2 | 9 | 60.00 |
| 1373309_at | Tmem86a | transmembrane protein 86A | 2.25 | 67.73 |
| 1387367_at | Glg1 | golgi glycoprotein 1 | 9 | 40.00 |
| 1378175_at | LOC102548514 | crooked neck-like protein 1-like | 6 | 32.85 |
| 1384262_at | Ppp1r3b | protein phosphatase 1, regulatory subunit 3B | 1.5 | 86.96 |
| 1377090_at | Dock6 | dedicator of cytokinesis 6 | 3.75 | 22.56 |
| 1391415_at | Pno1 | partner of NOB1 homolog | 6.75 | 40.18 |
| 1376593_at | Foxo3 | forkhead box O3 | 0.75 | 53.09 |
| 1367725_at | Pim3 | Pim-3 proto-oncogene, serine/threonine kinase | 0 | 85.23 |
| 1372417_at | Sertad1 | SERTA domain containing 1 | 0.75 | 54.98 |
| 1370909_at | Nup62 | nucleoporin 62 | 3 | 52.57 |
| 1381349_a_at | Ubr2 | ubiquitin protein ligase E3 component n-recognin 2 | 7.5 | 41.38 |
| 1374695_at | Cbx1 | chromobox 1 | 7.5 | 39.29 |
| 1372116_at | Mrps2 | mitochondrial ribosomal protein S2 | 1.5 | 29.94 |
| 1381804_at | Bcl6b | B-cell CLL/lymphoma 6B | 6 | 38.21 |
| 1372086_at | Fhdc1 | FH2 domain containing 1 | 2.25 | 47.39 |
| 1373340_at | Dusp9 | dual specificity phosphatase 9 | 9.75 | 19.67 |
| 1376858_at | Exoc3l2 | exocyst complex component 3-like 2 | 4.5 | 40.91 |
| 1393654_at | Neil1 | nei-like DNA glycosylase 1 | 0.75 | 30.99 |
| 1386069_at | Sp2 | Sp2 transcription factor | 6 | 32.74 |
| 1382185_at | C1qtnf2 | C1q and tumor necrosis factor related protein 2 | 6 | 63.27 |
| 1385423_at | Immt | inner membrane mitochondrial protein | 8.25 | 37.28 |
| 1376435_at | Loxl4 | Lysyl Oxidase Like 4 | 2.25 | 54.70 |
| 1374653_at | Fam73b | mitoguardin 2 | 3 | 0.44 |
| 1374013_at | C1qtnf5 | C1q and tumor necrosis factor related protein 5 | 3 | 62.75 |
| 1371537_at | B4galt5 | beta-1,4-galactosyltransferase 5 | 3 | 63.98 |
| 1389814_at | Huwe1 | HECT, UBA And WWE Domain Containing 1, E3 Ubiquitin Protein Ligase | 10.5 | 5.92 |
| 1384118_at | Zfp329 | zinc finger protein 329 | 7.5 | 72.39 |
| 1393044_at | Cmpk2 | cytidine/uridine monophosphate kinase 2 | 9 | 1.86 |
| 1377347_at | Foxj3 | Forkhead Box J3 | 6.75 | 46.65 |
| 1371425_at | Srrm1 | serine and arginine repetitive matrix 1 | 1.5 | 45.07 |
| 1368394_at | Sfrp4 | secreted frizzled-related protein 4 | 0.75 | 21.21 |
| 1371528_at | Fkbp8 | FK506 binding protein 8 | 0.75 | 54.52 |
| 1395663_at | Mall | Mal, T cell differentiation protein like | 6 | 6.06 |
| 1392557_at | Bicc1 | Bicaudal C homolog 1 | 0 | 48.04 |
| 1367945_at | Atox1 | antioxidant 1 copper chaperone | 4.5 | 2.35 |
| 1388525_at | Pik3ip1 | phosphoinositide-3-kinase interacting protein 1 | 0.75 | 55.48 |
| 1370526_at | Itgae | integrin subunit alpha E | 0.75 | 51.69 |
| 1383836_at | Retn | #N/A | 5.25 | 57.96 |
| 1389493_at | Abtb1 | ankyrin repeat and BTB domain containing 1 | 4.5 | 37.47 |
| 1371073_at | B4galt1 | beta-1,4-galactosyltransferase 1 | 8.25 | 65.53 |
| 1394816_at | Impa2 | |  | |  | Inositol Monophosphatase 2 | | --- | --- | | | --- | --- | --- | --- | | 2.25 | 27.36 |
| 1398930_at | Atp6v0b | ATPase H+ transporting V0 subunit B | 0 | 52.64 |
| 1371754_at | Slc25a25 | solute carrier family 25 member 25 | 0 | 52.63 |
| 1372340_at | Mat2b | methionine adenosyltransferase 2B | 2.25 | 33.58 |
| 1388674_at | Cdkn1a | cyclin-dependent kinase inhibitor 1A | 1.5 | 51.82 |
| 1390490_at | Mllt1 | MLLT1, super elongation complex subunit | 5.25 | 62.59 |
| 1393572_at | Zfp592 | zinc finger protein 592 | 7.5 | 50.00 |
| 1374421_at | Baz1b | bromodomain adjacent to zinc finger domain, 1B | 1.5 | 41.67 |
| 1383359_at | Lnx2 | ligand of numb-protein X 2 | 4.5 | 27.17 |
| 1387424_at | Cntn2 | contactin 2 | 0.75 | 56.01 |
| 1385250_at | Chd6 | chromodomain helicase DNA binding protein 6 | 8.25 | 18.49 |
| 1368290_at | Cyr61 | cysteine-rich, angiogenic inducer, 61 | 0 | 15.54 |
| 1384519_at | Sec63 | SEC63 homolog, protein translocation regulator | 7.5 | 33.27 |
| 1370789_a_at | Prlr | prolactin receptor | 4.5 | 29.91 |
| 1398767_at | Ubc | Ubiquitin B, C | 6 | 23.71 |
| 1376105_at | Col14a1 | collagen type XIV alpha 1 chain | 0.75 | 19.46 |
| 1388528_at | Fbl | Fibrillarin | 6.75 | 57.27 |
| 1384139_at | Gjc1 | gap junction protein, gamma 1 | 3 | 56.27 |
| 1370743_a_at | Lnpep | leucyl-cystinyl aminopeptidase-like | 9.75 | 3.71 |
| 1373904_at | Lysmd2 | LysM domain containing 2 | 6.75 | 42.82 |
| 1375174_at | Dpy19l1 | dpy-19 like 1 | 1.5 | 36.54 |
| 1394456_at | Thrb | thyroid hormone receptor beta | 7.5 | 27.52 |
| 1385397_at | Steap4 | STEAP4 metalloreductase | 12 | 18.39 |
| 1371654_at | RGD1305350 | similar to RIKEN cDNA 2510039O18 | 9.75 | 32.19 |
| 1398954_at | Dnpep | aspartyl aminopeptidase | 9.75 | 24.19 |
| 1396541_at | Jmjd1c | jumonji domain containing 1C | 9.75 | 65.68 |
| 1383587_at | Hacd3 | 3-hydroxyacyl-CoA dehydratase 3 | 9 | 26.72 |
| 1373237_at | Rufy3 | RUN And FYVE Domain Containing 3 | 6 | 85.66 |
| 1371981_at | Dscr3 | DSCR3 arrestin fold containing | 6.75 | 18.69 |
| 1368054_at | Lmna | lamin A/C | 6 | 59.49 |
| 1367499_at | Slc35c1 | solute carrier family 35 member C1 | 2.25 | 26.32 |
| 1379369_at | Prickle1 | Prickle like 1 | 11.25 | 67.75 |
| 1385701_at | Ahnak2 | AHNAK nucleoprotein 2 | 0.75 | 30.42 |
| 1373744_at | Anapc1 | anaphase promoting complex subunit 1 | 10.5 | 31.61 |
| 1368681_at | Pthlh | Parathyroid hormone like peptide | 1.5 | 65.19 |
| 1387979_at | Golgb1 | golgin B1 | 0 | 46.17 |
| 1370282_at | Csrp2 | cysteine and glycine-rich protein 2 | 5.25 | 61.58 |
| 1376971_at | Atg10 | autophagy related 10 | 1.5 | 71.78 |
| 1385494_at | Tbc1d23 | TBC1 domain family, member 23 | 6.75 | 4.50 |
| 1399048_at | Carm1 | coactivator-associated arginine methyltransferase 1 | 0.75 | 37.17 |
| 1371633_at | Ctnnbl1 | catenin, beta like 1 | 2.25 | 47.01 |
| 1371819_at | Hdac5 | histone deacetylase 5 | 5.25 | 37.96 |
| 1368298_at | Adcy5 | adenylate cyclase 5 | 8.25 | 56.07 |
| 1382946_a_at | Rab1b | RAB1B, member RAS oncogene family | 3 | 40.88 |
| 1380643_at | Pnpla3 | patatin-like phospholipase domain containing 3 | 0.75 | 45.21 |
| 1374036_at | Mcm2 | Minichromosome maintenance deficient 2 mitotin | 4.5 | 70.23 |
| 1370050_at | Atp2b1 | ATPase plasma membrane Ca2+ transporting 1 | 9.75 | 68.01 |
| 1377334_at | RT1-Ba | RT1 class II, locus Ba | 1.5 | 34.86 |
| 1387267_at | Ntf3 | neurotrophin 3 | 1.5 | 15.38 |
| 1392221_at | Greb1l | growth regulation by estrogen in breast cancer 1 like | 0.75 | 55.13 |
| 1388430_at | Ptov1 | prostate tumor overexpressed 1 | 3.75 | 13.70 |
| 1391835_at | Baz1a | bromodomain adjacent to zinc finger domain, 1A | 10.5 | 38.25 |
| 1373345_at | Amigo2 | adhesion molecule with Ig like domain 2 | 8.25 | 64.29 |
| 1367468_at | Scand1 | SCAN domain-containing 1 | 3.75 | 45.77 |
| 1388406_at | Naa60 | N-acetyltransferase 60, NatF catalytic subunit | 0 | 40.00 |
| 1376809_at | Fbrsl1 | fibrosin-like 1 | 6.75 | 42.62 |
| 1395990_at | Srcap | Snf2-related CREBBP activator protein | 9 | 32.28 |
| 1369200_at | Nt5e | 5' nucleotidase, ecto | 9 | 64.55 |
| 1393320_at | Utp15 | UTP15, small subunit processome component | 9.75 | 7.08 |
| 1395981_at | Ascc3 | activating signal cointegrator 1 complex subunit 3 | 9.75 | 49.86 |
| 1372288_at | Zmiz2 | zinc finger, MIZ-type containing 2 | 4.5 | 31.92 |
| 1390965_at | Nr5a2 | nuclear receptor subfamily 5, group A, member 2 | 4.5 | 30.41 |
| 1370012_at | Ptgis | prostaglandin I2 synthase | 3.75 | 42.23 |
| 1376380_at | Ogdh | Oxoglutarate Dehydrogenase | 3 | 41.24 |
| 1368358_a_at | Ptprr | protein tyrosine phosphatase, receptor type, R | 0.75 | 47.25 |
| 1374404_at | Jun | Jun proto-oncogene, AP-1 transcription factor subunit | 2.25 | 43.62 |
| 1376936_at | Slc8b1 | solute carrier family 8 member B1 | 3 | 46.73 |
| 1372865_at | Rnf115 | ring finger protein 115 | 9 | 65.97 |
| 1391078_at | Rfc1 | replication factor C subunit 1 | 8.25 | 79.84 |
| 1392441_at | LOC100912577 | uncharacterized LOC100912577 | 8.25 | 83.42 |
| 1370897_at | Bckdha | branched chain ketoacid dehydrogenase E1, alpha polypeptide | 0 | 43.34 |
| 1376599_at | Atad2 | ATPase family, AAA domain containing 2 | 1.5 | 58.99 |
| 1374516_at | Chtf8 | chromosome transmission fidelity factor 8 | 5.25 | 65.29 |
| 1387914_at | Cyp27a1 | cytochrome P450, family 27, subfamily a, polypeptide 1 | 6 | 28.41 |
| 1389357_at | Morc2 | MORC family CW-type zinc finger 2 | 10.5 | 8.06 |
| 1376423_at | Fbxl19 | F-box and leucine-rich repeat protein 19 | 7.5 | 50.49 |
| 1368275_at | Msmo1 | Sterol-C4-methyl oxidase-like | 7.5 | 36.39 |
| 1383114_at | Mark2 | Microtubule Affinity Regulating Kinase 2 | 6 | 29.56 |
| 1389299_at | Pcyt1a | choline-phosphate cytidylyltransferase A-like | 1.5 | 45.38 |
| 1392758_at | Tra2a | transformer 2 alpha homolog | 9.75 | 28.01 |
| 1388752_at | Bclaf1 | BCL2-associated transcription factor 1 | 3.75 | 50.59 |
| 1387262_at | Ssb | Sjogren syndrome antigen B | 7.5 | 29.76 |
| 1393375_at | Ackr2 | atypical chemokine receptor 2 | 0 | 35.57 |
| 1373728_at | RGD1359634 | similar to RIKEN cDNA 1700088E04 | 6.75 | 65.17 |
| 1387078_at | Inpp4a | inositol polyphosphate-4-phosphatase type I A | 5.25 | 54.77 |
| 1384019_a_at | Phf20 | PHD finger protein 20 | 12 | 24.26 |
| 1388582_at | Psme3 | glucose-6-phosphatase, catalytic subunit | 0.75 | 21.64 |
| 1370853_at | Camk2n1 | calcium/calmodulin-dependent protein kinase II inhibitor 1 | 6 | 4.69 |
| 1371520_at | Scaper | S-phase cyclin A-associated protein in the ER | 0.75 | 47.43 |
| 1373889_at | Igsf7 | similar to dendritic cell-derived immunoglobulin-like receptor 1, DIgR1 | 3.75 | 65.52 |
| 1387260_at | Klf4 | Kruppel like factor 4 | 3 | 56.03 |
| 1389467_at | Tmem100 | transmembrane protein 100 | 4.5 | 59.58 |
| 1391577_at | Pgam5 | Phosphoglycerate mutase family member 5 | 0.75 | 33.33 |
| 1382431_at | Abca1 | ATP binding cassette subfamily A member 1 | 1.5 | 80.00 |
| 1391005_at | Fbxl4 | F-box and leucine-rich repeat protein 4 | 6 | 44.78 |
| 1383207_at | Rrn3 | RNA polymerase I-specific transcription initiation factor RRN3-like | 9 | 72.54 |
| 1373216_at | Ccpg1os | cell cycle progression 1, opposite strand | 11.25 | 72.07 |
| 1374572_at | Gatsl3 | GATS protein-like 3 | 2.25 | 63.47 |
| 1370063_at | Nr2f2 | nuclear receptor subfamily 2, group F, member 2 | 10.5 | 69.85 |
| 1382579_at | Tox3 | TOX high mobility group box family member 3 | 0.75 | 51.56 |
| 1372590_at | C1qtnf1 | C1q tumor necrosis factor related protein 1 | 6 | 72.63 |
| 1374553_at | Fam32a | family with sequence similarity 32, member A | 4.5 | 4.03 |
| 1371898_at | Kcnk5 | potassium two pore domain channel subfamily K member 5 | 6.75 | 10.96 |
| 1392941_at | Hip1 | huntingtin interacting protein 1 | 2.25 | 60.96 |
| 1389145_at | Cdc42ep2 | CDC42 effector protein 2 | 3.75 | 63.17 |
| 1387675_at | Plau | plasminogen activator, urokinase | 11.25 | 69.83 |
| 1396225_at | Cpeb2 | Cytoplasmic Polyadenylation Element Binding Protein 2 | 11.25 | 90.70 |
| 1384316_at | Tmem63c | |  | Transmembrane Protein 63C | | --- | --- | | 0.75 | 15.99 |
| 1369664_at | Avpr1a | arginine vasopressin receptor 1A | 0 | 6.40 |
| 1379528_at | Psen1 | Presenilin 1 | 10.5 | 35.05 |
| 1397493_at | Papola | poly polymerase alpha | 12 | 17.30 |
| 1371589_at | Ubl5 | ubiquitin-like 5 | 0.75 | 16.70 |
| 1376476_at | Telo2 | telomere maintenance 2 | 6.75 | 74.63 |
| 1376134_at | Mri1 | methylthioribose-1-phosphate isomerase 1 | 6.75 | 55.90 |
| 1398598_at | Dst | dystonin | 8.25 | 68.03 |
| 1382161_at | Mphosph10 | M-phase phosphoprotein 10 | 5.25 | 66.67 |
| 1385765_at | Lin9 | lin-9 DREAM MuvB core complex component | 2.25 | 32.56 |
| 1392349_at | Slc5a3 | solute carrier family 5 member 3 | 11.25 | 59.43 |
| 1372024_at | Mafg | MAF bZIP transcription factor G | 2.25 | 7.83 |
| 1370341_at | Eno2 | gamma-enolase-like | 2.25 | 31.06 |
| 1385465_at | Siglec5 | sialic acid binding Ig-like lectin 5 | 0.75 | 51.39 |
| 1381475_at | Sdk2 | sidekick cell adhesion molecule 2 | 3.75 | 15.83 |
| 1379283_at | Braf | B-Raf Proto-Oncogene, Serine/Threonine Kinase | 10.5 | 42.99 |
| 1388514_at | Ppm1g | protein phosphatase, Mg2+/Mn2+ dependent, 1G | 0.75 | 75.00 |
| 1370428_x_at | RT1-A2 | RT1 class Ib, locus Aw2 | 0 | 21.33 |
| 1373954_at | Suds3 | SDS3 homolog, SIN3A corepressor complex component | 6 | 55.80 |
| 1397523_at | Lrp11 | LDL receptor related protein 11 | 11.25 | 48.81 |
| 1387505_at | Gnai1 | G protein subunit alpha i1 | 1.5 | 21.51 |
| 1393193_at | LOC100911166 | RNA pseudouridylate synthase domain-containing protein 2-like | 5.25 | 52.02 |
| 1397251_at | Ammecr1l | AMME chromosomal region gene 1 like | 0.75 | 73.14 |
| 1388692_at | Alkbh7 | alkB homolog 7 | 6.75 | 53.80 |
| 1372196_at | Ncor2 | nuclear receptor co-repressor 2 | 2.25 | 76.80 |
| 1383266_at | Sfrp1 | secreted frizzled-related protein 1 | 10.5 | 26.91 |
| 1380357_a_at | Lrrc8c | leucine rich repeat containing 8 family, member C | 10.5 | 53.66 |
| 1391110_at | Pde3a | Phosphodiesterase 3A | 12 | 48.71 |
| 1372223_at | Cpeb4 | Cytoplasmic Polyadenylation Element Binding Protein 4 | 7.5 | 67.30 |
| 1375536_at | Numb | NUMB, endocytic adaptor protein | 9 | 50.35 |
| 1383166_at | Ncoa1 | nuclear receptor coactivator 1 | 10.5 | 63.79 |
| 1384204_at | Srsf10 | Serine And Arginine Rich Splicing Factor 10 | 2.25 | 9.35 |
| 1373411_at | Eif1b | eukaryotic translation initiation factor 1B | 6 | 40.29 |
| 1376440_at | Rnf139 | Ring finger protein 139 | 9 | 52.13 |
| 1377045_at | LOC100363520 | -- | 1.5 | 62.21 |
| 1385299_at | Lpar4 | lysophosphatidic acid receptor 4 | 6 | 28.34 |
| 1370905_at | Dock9 | dedicator of cytokinesis 9 | 0.75 | 46.60 |
| 1372069_at | Kank1 | KN motif and ankyrin repeat domains 1 | 5.25 | 71.07 |
| 1392927_at | Paqr4 | Progestin and adipoQ receptor family member IV | 3 | 60.23 |
| 1367859_at | Tgfb3 | transforming growth factor, beta 3 | 5.25 | 56.23 |
| 1369149_at | Limk1 | LIM domain kinase 1 | 8.25 | 59.33 |
| 1380236_at | Itga9 | integrin subunit alpha 9 | 4.5 | 60.98 |
| 1377353_a_at | Tnfsf13 | TNF superfamily member 13 | 0 | 36.68 |
| 1377011_at | Fry | FRY microtubule binding protein | 3 | 51.13 |
| 1372570_at | Usp30 | ubiquitin specific peptidase 30 | 7.5 | 56.05 |
| 1371664_at | Pxn | paxillin | 4.5 | 72.90 |
| 1393090_at | LOC100361830 | translocation associated membrane protein 2 | 9.75 | 29.64 |
| 1376588_at | Mtmr12 | myotubularin related protein 12 | 4.5 | 35.54 |
| 1393875_at | Slc35d2 | solute carrier family 35 member D2 | 2.25 | 21.86 |
| 1380513_at | Rpap2 | RNA polymerase II associated protein 2 | 11.25 | 68.36 |
| 1368513_at | Sh3pxd2a | glutamyl aminopeptidase-like | 0.75 | 29.55 |
| 1388657_at | Dchs1 | dachsous cadherin-related 1 | 10.5 | 4.44 |

### Table G: Common genes retaining circadian expression in Liver, Adipose, and Lung. max Δφ indicates the maximum phase difference (in hours) and max ΔΑ the maximum amplitude difference (in % change) between two tissues (Equations 2,5).

| **ProbeID** | **Symbol** | **Gene Name** | **max Δφ [hr]** | **max ΔA [%]** |
| --- | --- | --- | --- | --- |
| 1371864_at | --- | --- | 1.5 | 42.37 |
| 1389412_at | --- | --- | 4.5 | 65.55 |
| 1399082_at | --- | --- | 3 | 72.02 |
| 1372136_at | --- | --- | 3 | 61.37 |
| 1373736_at | --- | --- | 6 | 40.73 |
| 1372949_at | --- | --- | 6.75 | 44.41 |
| 1376341_at | --- | --- | 0.75 | 22.64 |
| 1390430_at | Nr1d2 | nuclear receptor subfamily 1, group D, member 2 | 0.75 | 32.60 |
| 1372701_at | Hsp90aa1 /// LOC100362895 /// LOC103692716 | heat shock protein 1, alpha-like | 6 | 80.14 |
| 1371693_at | Ahsa1 | Activator of heat shock 90-kDa protein ATPase homolog 1 | 3 | 54.91 |
| 1387703_a_at | Usp2 | Ubiquitin-specific peptidase 2 | 0.75 | 45.30 |
| 1373718_at | Tubb2a | tubulin, beta 2A class 2a | 4.5 | 94.33 |
| 1374531_at | Slc6a6 | solute carrier family 6 member 6 | 11.25 | 86.14 |
| 1398240_at | Hspa8 /// LOC498426 /// LOC680121 /// LOC689955 | Heat shock protein 70 | 2.25 | 56.88 |
| 1387874_at | Dbp | D-box binding PAR bZIP transcription factor | 0.75 | 60.90 |
| 1371237_a_at | Mt1a /// Ttr | metallothionein 1 | 0.75 | 94.94 |
| 1370510_a_at | Arntl | aryl hydrocarbon receptor nuclear translocator-like | 0.75 | 27.08 |
| 1388898_at | Hsph1 | heat shock protein family H (Hsp110) member 1 | 3 | 31.67 |
| 1389844_at | Fkbp4 | FK506 binding protein 4 | 0.75 | 25.93 |
| 1372844_at | Efna1 | Ephrin A1 | 6 | 79.47 |
| 1375336_at | Hsp90ab1 | Heat shock 90-kDa protein 1, beta | 1.5 | 69.59 |
| 1388271_at | Mt2A | metallothionein 2A | 3 | 80.22 |
| 1389199_at | RGD1309079 | similar to Ab2-095 | 3.75 | 85.82 |
| 1373866_at | Coq10b | coenzyme Q10B | 0 | 71.22 |
| 1373158_at | Gpr146 | G protein-coupled receptor 146 | 3.75 | 53.66 |
| 1371505_at | Hnrnpc /// LOC100911576 | heterogeneous nuclear ribonucleoproteins C1/C2-like | 3 | 59.61 |
| 1367982_at | Alas1 | Aminolevulinic acid synthase 1 | 11.25 | 69.47 |
| 1371403_at | Cct3 | Chaperonin subunit 3 (gamma) | 2.25 | 78.09 |
| 1386987_at | Il6r | Interleukin 6 receptor | 3.75 | 71.76 |
| 1368303_at | Per2 | period circadian clock 2 | 3 | 28.26 |
| 1370816_at | Nr1d1 | nuclear receptor subfamily 1, group D, member 1 | 0.75 | 48.24 |
| 1383160_at | Chordc1 | Cysteine and histidine-rich domain (CHORD)-containing, zinc-bp1 | 2.25 | 65.28 |
| 1398877_at | Stip1 | stress-induced phosphoprotein 1 | 3 | 32.54 |
| 1377016_at | Creld2 | cysteine-rich with EGF-like domains 2 | 1.5 | 34.64 |
| 1390383_at | Plin2 | perilipin 2 | 2.25 | 53.08 |
| 1369636_at | Sord | sorbitol dehydrogenase | 9 | 32.74 |
| 1388722_at | Dnajb1 | DnaJ heat shock protein family member B1 | 3 | 40.22 |
| 1398819_at | Dnaja1 | DNAJ (Hsp40) homolog, subfamily A, member 1 | 3 | 32.65 |
| 1390199_at | Clock | clock circadian regulator | 0.75 | 58.96 |
| 1370381_at | Pnrc1 | Proline-rich nuclear receptor coactivator 1, F-box coactivator 1, F-box protein 11 | 0.75 | 74.44 |
| 1368275_at | Msmo1 | Sterol-C4-methyl oxidase-like | 11.25 | 76.41 |
| 1398960_at | Cct6a | chaperonin containing TCP1 subunit 6A | 3 | 86.03 |
| 1373542_at | Sphk2 | sphingosine kinase 2 | 2.25 | 36.13 |
| 1367741_at | Herpud1 | homocysteine inducible ER protein with ubiquitin like domain 1 | 0 | 53.52 |
| 1398998_at | RGD1309748 | -- | 0 | 54.40 |
| 1389355_at | Ier5 | immediate early response 5 | 2.25 | 37.01 |
| 1374709_at | Hlf | Hepatic leukemia factor | 3.75 | 56.29 |
| 1373309_at | Tmem86a | transmembrane protein 86A | 2.25 | 73.67 |
| 1390381_at | Xpc | Xeroderma pigmentosum, | 8.25 | 65.33 |
| 1367771_at | Tsc22d3 | Glucocorticoid-induced leucine zipper | 2.25 | 44.71 |
| 1388901_at | Fkbp5 | FK506 binding protein 5 | 2.25 | 58.31 |
| 1371684_at | Pelo | pelota mRNA surveillance and ribosome rescue factor | 10.5 | 50.94 |
| 1367850_at | Fcgr2a | low affinity immunoglobulin gamma Fc region receptor III-like | 3 | 83.92 |
| 1390107_at | Sytl2 | Synaptotagmin-like 2 | 4.5 | 45.57 |
| 1368486_at | Irs3 | insulin receptor substrate 3 | 3 | 3.07 |
| 1370336_at | Osgin1 | oxidative stress induced growth inhibitor 1 | 11.25 | 58.90 |
| 1372827_at | Ppid | peptidylprolyl isomerase D | 1.5 | 83.15 |
| 1390042_at | Tmem140 | Transmembrane Protein 140 | 3.75 | 29.98 |
| 1375892_at | Elavl1 | ELAV like RNA binding protein 1 | 5.25 | 26.44 |
| 1370991_at | Cml3 | probable N-acetyltransferase | 3.75 | 42.04 |
| 1387294_at | Sh3bp5 | SH3-domain binding protein 5 | 9.75 | 80.83 |
| 1374676_at | Sgms1 | Sphingomyelin Synthase 1 | 4.5 | 83.45 |
| 1387805_at | Bnip3 | BCL2/adenovirus E1B 19 kDa- | 1.5 | 33.12 |
| 1369664_at | Avpr1a | arginine vasopressin receptor 1A | 10.5 | 13.60 |
| 1368488_at | Nfil3 | nuclear factor, interleukin 3 regulated | 0 | 43.84 |
| 1370209_at | Klf9 | Kruppel-like factor 9 | 2.25 | 31.33 |
| 1368223_at | Adamts1 | Adam metallopeptidase with thrombospondin 1 motif, 15 | 8.25 | 39.93 |
| 1388686_at | Rcan1 | Regulator of calcineurin 1 | 7.5 | 45.76 |
| 1368549_at | Hbp1 | HMG-box transcription factor 1 | 0.75 | 67.07 |
| 1373093_at | Errfi1 | ERBB receptor feedback inhibitor 1 | 2.25 | 62.57 |
| 1368249_at | Klf15 | Kruppel-like factor 15 | 2.25 | 44.65 |
| 1372308_at | St3gal1 | ST3 beta-galactoside alpha-2,3-sialyltransferase 1 | 5.25 | 18.84 |
| 1389681_at | Pvrl2 | Poliovirus receptor-related 2 | 10.5 | 64.85 |
| 1373195_at | Fus | Fused in sarcoma | 1.5 | 45.17 |
| 1369644_at | Adgrl2 | adhesion G protein-coupled receptor L2 | 10.5 | 59.88 |
| 1388528_at | Fbl | Fibrillarin | 6.75 | 67.95 |
| 1371583_at | Rbm3 | RNA binding motif (RNP1, RRM) protein 3 | 2.25 | 18.35 |
| 1370283_at | Hspa5 | heat shock protein family A member 5 | 2.25 | 37.22 |
| 1389587_at | Umps | uridine monophosphate synthetase | 5.25 | 27.93 |
| 1371832_at | Leo1 | LEO1 homolog, Paf1/RNA polymerase II complex component | 0 | 19.03 |
| 1377353_a_at | Tnfsf13 | TNF superfamily member 13 | 3.75 | 59.17 |
| 1398431_at | Car8 | Carbonic anhydrase viii | 6.75 | 23.76 |
| 1388582_at | Psme3 | glucose-6-phosphatase, catalytic subunit | 6 | 26.32 |
| 1367568_a_at | Mgp | matrix Gla protein | 0.75 | 42.34 |
| 1398327_at | Fermt2 | fermitin family member 2 | 3.75 | 35.04 |
| 1398750_at | Calr | calreticulin | 0 | 31.57 |
| 1373114_at | Dtx4 | deltex E3 ubiquitin ligase 4 | 1.5 | 70.23 |
| 1388752_at | Bclaf1 | BCL2-associated transcription factor 1 | 3.75 | 62.20 |
| 1371929_at | Mlx | MLX, MAX dimerization protein | 6 | 36.82 |
| 1372340_at | Mat2b | methionine adenosyltransferase 2B | 4.5 | 43.76 |
| 1368021_at | Adh1 /// Adh6 | alcohol dehydrogenase 1 | 10.5 | 74.22 |
| 1372024_at | Mafg | MAF bZIP transcription factor G | 3 | 57.08 |
| 1383013_at | Klf13 | Kruppel-like factor 13 | 1.5 | 70.53 |
| 1389493_at | Abtb1 | ankyrin repeat and BTB domain containing 1 | 5.25 | 53.58 |
| 1398930_at | Atp6v0b | ATPase H+ transporting V0 subunit B | 7.5 | 61.57 |
| 1389538_at | Nfkbia | NFKB inhibitor alpha | 2.25 | 44.43 |
| 1372069_at | Kank1 | KN motif and ankyrin repeat domains 1 | 5.25 | 83.00 |
| 1369200_at | Nt5e | 5' nucleotidase, ecto | 9 | 64.55 |
| 1368173_at | Nop58 | NOP58 ribonucleoprotein | 9 | 54.30 |
| 1387914_at | Cyp27a1 | cytochrome P450, family 27, subfamily a, polypeptide 1 | 11.25 | 28.41 |
| 1370847_at | Spon2 | spondin 2 | 1.5 | 83.83 |
| 1374421_at | Baz1b | bromodomain adjacent to zinc finger domain, 1B | 2.25 | 59.59 |
| 1375916_at | Pcmtd2 | protein-L-isoaspartate O-methyltransferase domain containing 2 | 2.25 | 60.75 |
| 1388406_at | Naa60 | N-acetyltransferase 60, NatF catalytic subunit | 0 | 40.00 |
| 1390171_at | Fam76a | family with sequence similarity 76, member A | 4.5 | 25.65 |
| 1369919_at | Tef | TEF, PAR bZIP transcription factor | 4.5 | 62.81 |
| 1388674_at | Cdkn1a | cyclin-dependent kinase inhibitor 1A | 2.25 | 73.63 |
| 1398886_at | RGD1563348 | -- | 6.75 | 27.18 |
| 1367725_at | Pim3 | Pim-3 proto-oncogene, serine/threonine kinase | 2.25 | 85.23 |
| 1368247_at | Hspa1a /// Hspa1b | heat shock 70kD protein 1A | 1.5 | 58.15 |
| 1368177_at | Acsl3 | acyl-CoA synthetase long-chain family member 3 | 3.75 | 70.00 |
| 1374036_at | Mcm2 | Minichromosome maintenance deficient 2 mitotin | 5.25 | 89.93 |
| 1374947_at | Bcar3 | breast cancer anti-estrogen resistance 3 | 0 | 55.18 |
| 1372223_at | Cpeb4 | Cytoplasmic Polyadenylation Element Binding Protein 4 | 7.5 | 67.30 |
| 1367826_at | Nfe2l2 | nuclear factor, erythroid 2-like 2 | 12 | 32.78 |
| 1368511_at | Bhlhe41 | Basic helix-loop-helix domain containing, class B, 3 | 0 | 60.52 |
| 1376936_at | Slc8b1 | solute carrier family 8 member B1 | 3.75 | 50.86 |
| 1375536_at | Numb | NUMB, endocytic adaptor protein | 9 | 68.02 |
| 1388239_at | Per3 | period circadian clock 3 | 0.75 | 77.12 |
| 1370909_at | Nup62 | nucleoporin 62 | 3 | 66.41 |
| 1377287_at | LOC100911305 /// Mars2 | methionine--tRNA ligase, mitochondrial-like | 3 | 31.89 |
| 1374855_at | Per1 | period circadian clock 1 | 1.5 | 74.07 |
| 1383169_at | LOC103691479 | Leukemia inhibitory factor receptor ? | 3 | 60.56 |
| 1377334_at | RT1-Ba | RT1 class II, locus Ba | 2.25 | 83.00 |
| 1369943_at | Tgm2 | Transglutaminase 2 | 2.25 | 23.59 |
| 1372116_at | Mrps2 | mitochondrial ribosomal protein S2 | 4.5 | 29.94 |
| 1371986_at | Anp32a | Acidic Nuclear Phosphoprotein 32 Family Member A | 3.75 | 39.19 |
| 1370570_at | Nrp1 | Neuropilin 1 | 4.5 | 73.14 |
| 1375138_at | Timp3 | TIMP metallopeptidase inhibitor 3 | 2.25 | 57.05 |
| 1398788_at | Pdia3 | protein disulfide isomerase family A, member 3 | 5.25 | 63.18 |
| 1367946_at | Pdlim1 | PDZ and LIM domain 1 | 5.25 | 27.14 |
| 1391078_at | Rfc1 | replication factor C subunit 1 | 8.25 | 79.84 |
| 1372878_at | Zfr | zinc finger RNA binding protein | 3.75 | 52.79 |
| 1376435_at | Loxl4 | Lysyl Oxidase Like 4 | 2.25 | 54.70 |
| 1375898_at | Rbpms | RNA Binding Protein With Multiple Splicing | 7.5 | 50.21 |
| 1367932_at | Hmgcs1 | 3-hydroxy-3-methylglutaryl-CoA synthase 1 | 11.25 | 41.99 |
| 1376588_at | Mtmr12 | myotubularin related protein 12 | 10.5 | 42.38 |
| 1372390_at | Peg3 | paternally expressed 3 | 9 | 53.87 |
| 1370215_at | C1qb | complement C1q B chain | 4.5 | 48.56 |
| 1373055_at | Tbcel | tubulin folding cofactor E-like | 0.75 | 12.59 |
| 1388795_at | Tppp | tubulin polymerization promoting protein | 3.75 | 7.99 |
| 1373147_at | Fbxl3 | F-box and leucine-rich repeat protein 3 | 1.5 | 36.11 |
| 1371819_at | Hdac5 | histone deacetylase 5 | 5.25 | 37.96 |
| 1375080_at | Slc25a45 | solute carrier family 25, member 45 | 9.75 | 19.84 |
| 1387267_at | Ntf3 | neurotrophin 3 | 3 | 17.66 |
| 1377474_at | Abhd13 | abhydrolase domain containing 13 | 2.25 | 7.43 |
| 1374349_at | Ctdspl | Ctd small phosphatase-like | 0.75 | 68.97 |
| 1399022_at | Clk1 | CDC-like kinase 1 | 5.25 | 66.46 |
| 1371754_at | Slc25a25 | solute carrier family 25 member 25 | 9 | 60.18 |
| 1373824_at | Cfdp1 | Flavin | 9 | 18.98 |
| 1374568_at | Cipc | CLOCK-interacting pacemaker | 3 | 62.59 |
| 1374945_at | LOC103692719 /// Trmt61a | tRNA methyltransferase 61A | 9 | 35.43 |
| 1372213_at | LOC500300 | -- | 9 | 41.41 |
| 1373240_at | Dhrs3 | Dehydrogenase/reductase 3 | 9 | 58.22 |
| 1375658_at | Sash1 | SAM and SH3 domain containing 1 | 3 | 48.86 |
| 1373014_at | B3gat3 | beta-1,3-glucuronyltransferase 3 | 4.5 | 57.55 |
| 1387024_at | Dusp6 | dual specificity phosphatase 6 | 6 | 18.79 |
| 1387669_a_at | Ephx1 | Epoxide hydrolase 1, | 3.75 | 44.76 |
| 1368304_at | Fmo3 | flavin containing monooxygenase 3 | 3.75 | 80.60 |
| 1392490_at | Tmem57 | Transmembrane protein 57 | 6 | 52.18 |
| 1374932_at | Angptl8 | angiopoietin-like 8 | 2.25 | 29.38 |
| 1373302_at | Acer2 | alkaline ceramidase 2 | 5.25 | 67.05 |
| 1375367_at | Pdlim2 | PDZ and LIM domain 2 | 2.25 | 50.16 |
| 1371839_at | Srsf2 | serine and arginine rich splicing factor 2 | 10.5 | 60.00 |
| 1370912_at | Hspa1a | Heat shock 70kD protein 1B | 2.25 | 73.83 |
| 1367539_at | Gtf2a1 | General Transcription Factor IIA Subunit 1 | 2.25 | 60.40 |

### Table H: Common genes retaining circadian expression in Liver, Muscle, and Adipose. max Δφ indicates the maximum phase difference (in hours) and max ΔΑ the maximum amplitude difference (in % change) between two tissues (Equations 2,5).

| **Probe ID** | **Symbol** | **Gene names** | **max Δφ [hr]** | **max ΔΑ [%]** |
| --- | --- | --- | --- | --- |
| 1371864_at | --- | --- | 2.25 | 50.54 |
| 1375879_at | --- | --- | 4.5 | 53.51 |
| 1399082_at | --- | --- | 4.5 | 57.42 |
| 1372136_at | --- | --- | 2.25 | 45.99 |
| 1375781_at | --- | --- | 3 | 54.99 |
| 1375760_at | --- | --- | 2.25 | 16.20 |
| 1376341_at | --- | --- | 1.5 | 65.21 |
| 1390430_at | Nr1d2 | nuclear receptor subfamily 1, group D, member 2 | 0.75 | 56.33 |
| 1372701_at | Hsp90aa1 | heat shock protein 1, alpha-like | 6 | 80.14 |
| 1387703_a_at | Usp2 | Ubiquitin-specific peptidase 2 | 0.75 | 33.23 |
| 1387874_at | Dbp | D-box binding PAR bZIP transcription factor | 0.75 | 59.79 |
| 1371237_a_at | Mt1a /// Ttr | metallothionein 1 | 1.5 | 88.44 |
| 1370510_a_at | Arntl | aryl hydrocarbon receptor nuclear translocator-like | 0.75 | 15.85 |
| 1373866_at | Coq10b | coenzyme Q10B | 2.25 | 70.21 |
| 1373158_at | Gpr146 | G protein-coupled receptor 146 | 0.75 | 64.73 |
| 1386987_at | Il6r | Interleukin 6 receptor | 3 | 65.18 |
| 1368303_at | Per2 | period circadian clock 2 | 2.25 | 44.08 |
| 1370816_at | Nr1d1 | nuclear receptor subfamily 1, group D, member 1 | 1.5 | 55.65 |
| 1374636_at | Jade1 | PHD finger protein 17 | 3.75 | 53.77 |
| 1398819_at | Dnaja1 | DNAJ (Hsp40) homolog, subfamily A, member 1 | 3.75 | 32.65 |
| 1390199_at | Clock | clock circadian regulator | 3.75 | 58.96 |
| 1370381_at | Pnrc1 | Proline-rich nuclear receptor coactivator 1, F-box coactivator 1, F-box protein 11 | 0.75 | 63.78 |
| 1373542_at | Sphk2 | sphingosine kinase 2 | 2.25 | 40.72 |
| 1367741_at | Herpud1 | homocysteine inducible ER protein with ubiquitin like domain 1 | 2.25 | 62.60 |
| 1398998_at | RGD1309748 | -- | 2.25 | 82.55 |
| 1398246_s_at | Fcgr2a /// | low affinity immunoglobulin gamma Fc region receptor III-like | 1.5 | 43.34 |
| 1367771_at | Tsc22d3 | Glucocorticoid-induced leucine zipper | 1.5 | 55.28 |
| 1388901_at | Fkbp5 | FK506 binding protein 5 | 6.75 | 54.40 |
| 1367850_at | Fcgr2a /// | low affinity immunoglobulin gamma Fc region receptor III-like | 2.25 | 34.01 |
| 1370336_at | Osgin1 | oxidative stress induced growth inhibitor 1 | 11.25 | 34.30 |
| 1390042_at | Tmem140 | Transmembrane Protein 140 | 2.25 | 29.98 |
| 1387294_at | Sh3bp5 | SH3-domain binding protein 5 | 9.75 | 80.83 |
| 1368488_at | Nfil3 | nuclear factor, interleukin 3 regulated | 0 | 43.84 |
| 1370209_at | Klf9 | Kruppel-like factor 9 | 3.75 | 43.06 |
| 1388686_at | Rcan1 | Regulator of calcineurin 1 | 8.25 | 58.90 |
| 1368549_at | Hbp1 | HMG-box transcription factor 1 | 2.25 | 46.71 |
| 1368249_at | Klf15 | Kruppel-like factor 15 | 4.5 | 38.08 |
| 1375677_at | Tob2 | transducer of ERBB2, 2 | 2.25 | 28.64 |
| 1371583_at | Rbm3 | RNA binding motif (RNP1, RRM) protein 3 | 0.75 | 25.77 |
| 1370283_at | Hspa5 | heat shock protein family A member 5 | 5.25 | 48.19 |
| 1371832_at | Leo1 | LEO1 homolog, Paf1/RNA polymerase II complex component | 3 | 12.39 |
| 1372091_at | Mid1ip1 | MID1 interacting protein 1 | 4.5 | 45.55 |
| 1398431_at | Car8 | Carbonic anhydrase viii | 3.75 | 32.60 |
| 1367568_a_at | Mgp | matrix Gla protein | 0.75 | 23.49 |
| 1389538_at | Nfkbia | NFKB inhibitor alpha | 2.25 | 38.81 |
| 1372069_at | Kank1 | KN motif and ankyrin repeat domains 1 | 2.25 | 45.48 |
| 1369200_at | Nt5e | 5' nucleotidase, ecto | 8.25 | 34.82 |
| 1370847_at | Spon2 | spondin 2 | 0.75 | 61.43 |
| 1374421_at | Baz1b | bromodomain adjacent to zinc finger domain, 1B | 4.5 | 49.28 |
| 1375916_at | Pcmtd2 | protein-L-isoaspartate O-methyltransferase domain containing 2 | 1.5 | 20.19 |
| 1371150_at | Ccnd1 | cyclin D1 | 7.5 | 81.27 |
| 1390171_at | Fam76a | family with sequence similarity 76, member A | 2.25 | 31.50 |
| 1369919_at | Tef | TEF, PAR bZIP transcription factor | 4.5 | 58.22 |
| 1368247_at | Hspa1a /// Hspa1b | heat shock 70kD protein 1A | 5.25 | 58.15 |
| 1367802_at | Sgk1 | serum/glucocorticoid regulated kinase 1 | 9.75 | 14.67 |
| 1368511_at | Bhlhe41 | Basic helix-loop-helix domain containing, class B, 3 | 1.5 | 46.53 |
| 1374855_at | Per1 | period circadian clock 1 | 1.5 | 74.07 |
| 1383169_at | LOC103691479 | Leukemia inhibitory factor receptor ? | 9 | 60.47 |
| 1377334_at | RT1-Ba | RT1 class II, locus Ba | 0.75 | 92.51 |
| 1369943_at | Tgm2 | Transglutaminase 2 | 2.25 | 61.14 |
| 1370570_at | Nrp1 | Neuropilin 1 | 4.5 | 73.14 |
| 1375138_at | Timp3 | TIMP metallopeptidase inhibitor 3 | 1.5 | 50.88 |
| 1376435_at | Loxl4 | Lysyl Oxidase Like 4 | 1.5 | 35.19 |
| 1372390_at | Peg3 | paternally expressed 3 | 10.5 | 58.28 |
| 1370215_at | C1qb | complement C1q B chain | 4.5 | 53.05 |
| 1388795_at | Tppp | tubulin polymerization promoting protein | 6 | 36.40 |
| 1387521_at | Pdcd4 | programmed cell death 4 | 2.25 | 29.67 |
| 1399022_at | Clk1 | CDC-like kinase 1 | 7.5 | 24.11 |
| 1387116_at | Dnajb9 | DnaJ heat shock protein family member B9 | 1.5 | 38.65 |
| 1371754_at | Slc25a25 | solute carrier family 25 member 25 | 9 | 58.76 |
| 1368283_at | Ehhadh | Enoyl-coenzyme A, hydratase/3- hydroxyacyl coenzyme A dehydrogenase | 12 | 52.27 |
| 1373240_at | Dhrs3 | Dehydrogenase/reductase 3 | 9.75 | 61.98 |
| 1375658_at | Sash1 | SAM and SH3 domain containing 1 | 3 | 24.24 |
| 1387024_at | Dusp6 | dual specificity phosphatase 6 | 9.75 | 38.42 |
| 1387669_a_at | Ephx1 | Epoxide hydrolase 1, | 3.75 | 38.61 |
| 1368304_at | Fmo3 | flavin containing monooxygenase 3 | 4.5 | 68.09 |
| 1392490_at | Tmem57 | Transmembrane protein 57 | 4.5 | 52.18 |
| 1376621_at | Wdr26 | WD repeat domain 26 | 3 | 36.30 |
| 1370912_at | Hspa1a | Heat shock 70kD protein 1B | 1.5 | 73.83 |

### Table I: Common genes retaining circadian expression in Liver, Muscle, and Lung. max Δφ indicates the maximum phase difference (in hours) and max ΔΑ the maximum amplitude difference (in % change) between two tissues (Equations 2,5).

| **Probe ID** | **Symbols** | **Gene name** | **max Δφ [hr]** | **max ΔA [%]** |
| --- | --- | --- | --- | --- |
| 1390215_at | --- | --- | 1.5 | 51.85 |
| 1371864_at | --- | --- | 2.25 | 50.54 |
| 1399082_at | --- | --- | 4.5 | 72.02 |
| 1372136_at | --- | --- | 2.25 | 61.37 |
| 1372011_at | --- | --- | 5.25 | 53.46 |
| 1377149_at | --- | --- | 6 | 27.33 |
| 1372031_at | --- | --- | 2.25 | 68.41 |
| 1389618_at | --- | --- | 6 | 81.99 |
| 1372220_at | --- | --- | 6 | 52.97 |
| 1376341_at | --- | --- | 1.5 | 70.94 |
| 1390430_at | Nr1d2 | nuclear receptor subfamily 1, group D, member 2 | 0.75 | 56.33 |
| 1387109_at | Por | P450 (cytochrome) oxidoreductase | 5.25 | 67.53 |
| 1372701_at | Hsp90aa1 /// LOC100362895 /// LOC103692716 | heat shock protein 1, alpha-like | 3 | 61.64 |
| 1387703_a_at | Usp2 | Ubiquitin-specific peptidase 2 | 0 | 44.10 |
| 1387874_at | Dbp | D-box binding PAR bZIP transcription factor | 0.75 | 66.65 |
| 1371237_a_at | Mt1a /// Ttr | metallothionein 1 | 1.5 | 94.94 |
| 1370510_a_at | Arntl | aryl hydrocarbon receptor nuclear translocator-like | 0.75 | 29.12 |
| 1386946_at | Cpt1a | Carnitine palmitoyltransferase I | 2.25 | 73.27 |
| 1371976_at | Fam195a | Unknown | 2.25 | 74.78 |
| 1373108_at | Ppp1r3c | protein phosphatase 1, regulatory subunit 3C | 2.25 | 73.17 |
| 1370928_at | Litaf | lipopolysaccharide-induced TNF factor | 2.25 | 69.69 |
| 1373866_at | Coq10b | coenzyme Q10B | 2.25 | 72.18 |
| 1388850_at | Hsp90aa1 /// LOC103692716 /// LOC680491 | heat shock protein 90, alpha (cytosolic), class A member 1 | 3 | 71.47 |
| 1373158_at | Gpr146 | G protein-coupled receptor 146 | 3.75 | 63.57 |
| 1386987_at | Il6r | Interleukin 6 receptor | 6.75 | 71.76 |
| 1368303_at | Per2 | period circadian clock 2 | 3 | 41.51 |
| 1370816_at | Nr1d1 | nuclear receptor subfamily 1, group D, member 1 | 1.5 | 73.50 |
| 1372752_at | LOC100911766 /// Tspan4 | tetraspanin-4-like | 5.25 | 65.77 |
| 1398819_at | Dnaja1 | DNAJ (Hsp40) homolog, subfamily A, member 1 | 3.75 | 6.43 |
| 1390199_at | Clock | clock circadian regulator | 3.75 | 40.55 |
| 1370381_at | Pnrc1 | Proline-rich nuclear receptor coactivator 1, F-box coactivator 1, F-box protein 11 | 1.5 | 74.44 |
| 1373542_at | Sphk2 | sphingosine kinase 2 | 2.25 | 24.93 |
| 1372056_at | Cmtm6 | CKLF-like MARVEL transmembrane domain containing 6 | 3.75 | 70.67 |
| 1367741_at | Herpud1 | homocysteine inducible ER protein with ubiquitin like domain 1 | 2.25 | 62.60 |
| 1398998_at | RGD1309748 | -- | 2.25 | 82.55 |
| 1372004_at | Hebp1 | Heme binding protein 1 | 6.75 | 41.41 |
| 1369467_a_at | Pfkfb1 | 6-Phosphofructo-2-kinase/fructose-2,6-bisphosphatase 1 | 4.5 | 58.18 |
| 1390117_at | Ypel2 | Yippee-like 2 | 1.5 | 61.05 |
| 1399159_a_at | Vamp3 | vesicle-associated membrane protein 3 | 7.5 | 75.40 |
| 1370663_at | Wee1 | WEE1 G2 checkpoint kinase | 2.25 | 35.64 |
| 1371953_at | Ccng2 | cyclin G2 | 3.75 | 66.84 |
| 1367771_at | Tsc22d3 | Glucocorticoid-induced leucine zipper | 3 | 64.84 |
| 1388901_at | Fkbp5 | FK506 binding protein 5 | 6.75 | 58.31 |
| 1367850_at | Fcgr2a | low affinity immunoglobulin gamma Fc region receptor III-like | 2.25 | 84.50 |
| 1374914_at | Ppard | peroxisome proliferator-activated receptor delta | 4.5 | 79.38 |
| 1370336_at | Osgin1 | oxidative stress induced growth inhibitor 1 | 11.25 | 58.90 |
| 1390042_at | Tmem140 | Transmembrane Protein 140 | 1.5 | 18.31 |
| 1369150_at | Pdk4 | pyruvate dehydrogenase kinase 4 | 10.5 | 49.42 |
| 1387244_at | Cgrrf1 | Cgr19 | 2.25 | 63.16 |
| 1388471_at | Tcp11l2 | t-complex 11 like 2 | 2.25 | 57.04 |
| 1388395_at | G0s2 | G0/G1switch 2 | 1.5 | 63.61 |
| 1373282_at | Slc25a33 | solute carrier family 25 member 33 | 5.25 | 44.44 |
| 1387294_at | Sh3bp5 | SH3-domain binding protein 5 | 6 | 59.09 |
| 1370019_at | Sult1a1 | sulfotransferase family 1A member 1 | 6 | 41.41 |
| 1376089_at | Ldlr | low density lipoprotein receptor | 9.75 | 64.16 |
| 1368488_at | Nfil3 | nuclear factor, interleukin 3 regulated | 0 | 35.11 |
| 1369976_at | Dynll1 | Dynein, cytoplasmic, light chain 1 | 3.75 | 65.66 |
| 1388819_at | Scamp1 | secretory carrier membrane protein 1 | 6 | 30.56 |
| 1370209_at | Klf9 | Kruppel-like factor 9 | 3.75 | 43.06 |
| 1371329_at | Eif5a | eukaryotic translation initiation factor 5A | 3.75 | 11.50 |
| 1388686_at | Rcan1 | Regulator of calcineurin 1 | 8.25 | 46.13 |
| 1369973_at | Xdh | xanthine dehydrogenase(Xdh) | 4.5 | 41.69 |
| 1368549_at | Hbp1 | HMG-box transcription factor 1 | 2.25 | 67.07 |
| 1368249_at | Klf15 | Kruppel-like factor 15 | 4.5 | 52.21 |
| 1388136_at | Timm9 | Translocase of inner mitochondrial membrane 9 | 9 | 38.85 |
| 1386944_a_at | G6pc | glucose-6-phosphatase, catalytic subunit | 9.75 | 84.88 |
| 1386885_at | Ech1 | enoyl-CoA hydratase 1 | 10.5 | 19.32 |
| 1388426_at | Srebf1 | sterol regulatory element binding transcription factor 1 | 3.75 | 35.79 |
| 1386864_at | Pgam1 | phosphoglycerate mutase 1 | 5.25 | 72.56 |
| 1370575_a_at | Azin1 | antizyme inhibitor 1 | 10.5 | 65.11 |
| 1374798_at | LOC102547850 /// Tor1aip2 | torsin 1A interacting protein 2 | 3.75 | 48.81 |
| 1371249_at | Xbp1 | X-box binding protein 1 | 8.25 | 73.54 |
| 1371583_at | Rbm3 | RNA binding motif (RNP1, RRM) protein 3 | 2.25 | 32.70 |
| 1370283_at | Hspa5 | heat shock protein family A member 5 | 5.25 | 48.19 |
| 1374752_at | Mdfic | Myod family inhibitor domain containing | 2.25 | 26.35 |
| 1367839_at | Fdft1 | farnesyl diphosphate farnesyl transferase 1 | 10.5 | 8.62 |
| 1388365_at | Atp6v0d1 | ATPase H+ transporting V0 subunit D1 | 10.5 | 42.65 |
| 1371832_at | Leo1 | LEO1 homolog, Paf1/RNA polymerase II complex component | 3 | 20.63 |
| 1370172_at | Sod2 | superoxide dismutase 2 | 4.5 | 1.45 |
| 1367755_at | Cdo1 | Cysteine dioxygenase, type i | 6 | 81.80 |
| 1398431_at | Car8 | Carbonic anhydrase viii | 6.75 | 32.60 |
| 1368552_at | Grpel1 | GrpE-like 1, mitochondrial | 2.25 | 51.98 |
| 1367568_a_at | Mgp | matrix Gla protein | 0.75 | 54.46 |
| 1376861_at | LOC686087 /// Mospd1 | Motile sperm domain containing 1 | 10.5 | 44.63 |
| 1376700_at | Lima1 | LIM domain and actin binding 1 | 4.5 | 73.36 |
| 1367795_at | Ifrd1 | interferon-related developmental regulator 1 | 8.25 | 85.06 |
| 1368016_at | Pecr | peroxisomal trans-2-enoyl-CoA reductase | 4.5 | 28.67 |
| 1374750_at | Fnip1 | Folliculin Interacting Protein 1 | 2.25 | 55.70 |
| 1371729_at | Ypel5 | yippee-like 5 | 4.5 | 42.80 |
| 1369959_at | Zfp36l1 | zinc finger protein 36, C3H type-like 1 | 2.25 | 45.68 |
| 1389538_at | Nfkbia | NFKB inhibitor alpha | 2.25 | 49.85 |
| 1372069_at | Kank1 | KN motif and ankyrin repeat domains 1 | 6 | 84.23 |
| 1369200_at | Nt5e | 5' nucleotidase, ecto | 1.5 | 59.65 |
| 1389003_at | Rhobtb3 | Rho-related BTB domain containing 3 | 4.5 | 39.73 |
| 1370298_at | Coa3 | cytochrome C oxidase assembly factor 3 | 7.5 | 58.91 |
| 1398891_at | Mrpl15 | Mitochondrial ribosomal protein L15 | 4.5 | 33.93 |
| 1370847_at | Spon2 | spondin 2 | 0.75 | 83.83 |
| 1389984_at | Jarid2 | jumonji and AT-rich interaction domain containing 2 | 3.75 | 81.13 |
| 1374421_at | Baz1b | bromodomain adjacent to zinc finger domain, 1B | 4.5 | 59.59 |
| 1375916_at | Pcmtd2 | protein-L-isoaspartate O-methyltransferase domain containing 2 | 2.25 | 62.02 |
| 1372347_at | Skil | SKI-like proto-oncogene | 11.25 | 69.00 |
| 1390157_at | Ube2h | ubiquitin-conjugating enzyme E2H(Ube2h) Rattus norvegicus | 4.5 | 64.31 |
| 1367894_at | Insig1 | insulin induced gene 1 | 3.75 | 54.88 |
| 1390171_at | Fam76a | family with sequence similarity 76, member A | 5.25 | 23.03 |
| 1368136_at | Tmpo | Thymopoietin | 2.25 | 54.84 |
| 1369919_at | Tef | TEF, PAR bZIP transcription factor | 3 | 62.81 |
| 1368247_at | Hspa1a /// Hspa1b | heat shock 70kD protein 1A | 5.25 | 55.86 |
| 1389586_at | Ednrb | Endothelin Receptor Type B | 8.25 | 53.07 |
| 1388408_at | Prr13 | Proline rich 13 | 1.5 | 29.16 |
| 1368127_at | Neu2 | neuraminidase 2 | 4.5 | 61.86 |
| 1376187_at | Slc35d1 | Solute Carrier Family 35 Member D1 | 9 | 43.98 |
| 1368511_at | Bhlhe41 | Basic helix-loop-helix domain containing, class B, 3 | 1.5 | 57.42 |
| 1367609_at | LOC103694877 /// Mif | Macrophage migration inhibitory factor | 3.75 | 44.36 |
| 1367578_at | Prdx2 | peroxiredoxin 2 | 6 | 66.80 |
| 1388153_at | Acsl1 | acyl-CoA synthetase long-chain family member 1 | 12 | 50.65 |
| 1386937_at | Atp1b1 | Atpase, na+/k+ transporting, β 1 | 6.75 | 65.41 |
| 1374855_at | Per1 | period circadian clock 1 | 1.5 | 58.59 |
| 1398913_at | Numa1 | nuclear mitotic apparatus protein 1 | 3.75 | 47.98 |
| 1383169_at | LOC103691479 | Leukemia inhibitory factor receptor ? | 9 | 63.74 |
| 1377334_at | RT1-Ba | RT1 class II, locus Ba | 2.25 | 95.12 |
| 1369943_at | Tgm2 | Transglutaminase 2 | 2.25 | 62.24 |
| 1389000_at | Kdm3b | lysine demethylase 3B | 4.5 | 39.56 |
| 1374449_at | Cdca3 | cell division cycle associated 3 | 7.5 | 45.30 |
| 1370570_at | Nrp1 | Neuropilin 1 | 4.5 | 55.29 |
| 1375138_at | Timp3 | TIMP metallopeptidase inhibitor 3 | 3 | 39.48 |
| 1371041_at | Ndufv2 | NADH:ubiquinone oxidoreductase core subunit V2 | 4.5 | 53.63 |
| 1374574_at | Fam214a | family with sequence similarity 214, member A | 9.75 | 42.86 |
| 1376435_at | Loxl4 | Lysyl Oxidase Like 4 | 0.75 | 48.67 |
| 1373270_at | Wipi1 | WD repeat domain, phosphoinositide interacting 1 | 7.5 | 12.76 |
| 1377021_at | Trmt6 | tRNA methyltransferase 6 | 6 | 66.76 |
| 1374425_at | Tle1 | transducin like enhancer of split 1 | 10.5 | 58.08 |
| 1372390_at | Peg3 | paternally expressed 3 | 10.5 | 21.73 |
| 1370215_at | C1qb | complement C1q B chain | 3 | 53.05 |
| 1367602_at | Cited2 | Cbp/p300-interacting transactivator Glu/ Asp-rich carboxy-term Dom 3 | 12 | 77.96 |
| 1379550_a_at | Gtf2ird1 | general transcription factor II-I repeat domain-containing protein 1-like | 2.25 | 50.49 |
| 1388795_at | Tppp | tubulin polymerization promoting protein | 4.5 | 36.40 |
| 1376646_at | Popdc2 | Popeye domain containing 2 | 3 | 43.97 |
| 1374006_at | Ccbl2 | kynurenine aminotransferase 3 | 3 | 66.54 |
| 1371955_at | Mrpl35 | mitochondrial ribosomal protein L35 | 7.5 | 51.25 |
| 1367902_at | Gng11 /// LOC100912034 | Guanine nucleotide binding protein , γ 11 | 4.5 | 33.13 |
| 1399022_at | Clk1 | CDC-like kinase 1 | 7.5 | 66.46 |
| 1371754_at | Slc25a25 | solute carrier family 25 member 25 | 9 | 80.46 |
| 1376927_at | Lrrc14b | leucine rich repeat containing 14B | 9 | 48.52 |
| 1369092_at | Sec22a | Sec22 vesicle trafficking protein homolog a | 5.25 | 7.71 |
| 1370370_at | Hyal2 | hyaluronoglucosaminidase 2 | 3.75 | 40.58 |
| 1390028_at | Dyrk2 | Dual-specificity tyrosine- (Y)-phosphorylation regulated kinase 2 | 3 | 80.87 |
| 1373240_at | Dhrs3 | Dehydrogenase/reductase 3 | 9.75 | 61.98 |
| 1387053_at | Fmo1 | Flavin-containing monooxygenase 1 | 11.25 | 33.47 |
| 1375658_at | Sash1 | SAM and SH3 domain containing 1 | 4.5 | 59.78 |
| 1387024_at | Dusp6 | dual specificity phosphatase 6 | 9.75 | 38.42 |
| 1367463_at | Phb2 | prohibitin 2 | 7.5 | 55.18 |
| 1387669_a_at | Ephx1 | Epoxide hydrolase 1, | 3.75 | 36.54 |
| 1368304_at | Fmo3 | flavin containing monooxygenase 3 | 4.5 | 80.60 |
| 1392490_at | Tmem57 | Transmembrane protein 57 | 6 | 35.21 |
| 1389393_at | Abhd17c | abhydrolase domain containing 17C | 6 | 54.55 |
| 1369590_a_at | Ddit3 | DNA-damage-inducible transcript 3 | 3.75 | 65.74 |
| 1367577_at | Hspb1 | Heat shock 27kda protein 1 | 4.5 | 42.29 |
| 1389064_at | Fem1c | fem-1 homolog C | 4.5 | 44.70 |
| 1399077_at | Mtx1 | Metaxin 1 | 7.5 | 32.92 |
| 1374674_at | Atxn3 | ataxin 3 | 5.25 | 58.92 |
| 1376869_at | Tle4 | transducin-like enhancer of split 4 | 3.75 | 77.41 |
| 1370912_at | Hspa1a | Heat shock 70kD protein 1B | 3 | 64.47 |
| 1388519_at | Sec61b | Sec61 β subunit | 5.25 | 54.01 |
| 1389088_at | Adnp | activity-dependent neuroprotector homeobox | 5.25 | 45.63 |

### Table J: Common genes retaining circadian expression in Muscle, Adipose, and Lung. max Δφ indicates the maximum phase difference (in hours) and max ΔΑ the maximum amplitude difference (in % change) between two tissues (Equations 2,5).

| **Probe ID** | **Symbol** | **Gene name** | **max Δφ [hr[** | **max ΔΑ [%]** |
| --- | --- | --- | --- | --- |
| 1371864_at | --- | --- | 0.75 | 26.24 |
| 1376341_at | --- | --- | 1.5 | 70.94 |
| 1372136_at | --- | --- | 3 | 30.18 |
| 1374307_at | --- | --- | 8.25 | 38.51 |
| 1373225_at | --- | --- | 1.5 | 36.54 |
| 1386185_at | --- | --- | 10.5 | 43.67 |
| 1386186_s_at | --- | --- | 10.5 | 31.87 |
| 1389085_at | --- | --- | 1.5 | 14.95 |
| 1390743_at | --- | --- | 0.75 | 37.97 |
| 1376574_at | --- | --- | 1.5 | 15.17 |
| 1388479_at | --- | --- | 4.5 | 59.81 |
| 1376848_at | --- | --- | 5.25 | 48.26 |
| 1399082_at | --- | --- | 3.75 | 41.16 |
| 1387294_at | Sh3bp5 | SH3-domain binding protein 5 | 6 | 79.98 |
| 1398767_at | Ubc | Ubiquitin B, C | 6 | 87.69 |
| 1370816_at | Nr1d1 | nuclear receptor subfamily 1, group D, member 1 | 0.75 | 73.50 |
| 1367850_at | Fcgr2a | low affinity immunoglobulin gamma Fc region receptor III-like | 3 | 84.50 |
| 1390430_at | Nr1d2 | nuclear receptor subfamily 1, group D, member 2 | 0 | 48.52 |
| 1368303_at | Per2 | period circadian clock 2 | 0.75 | 44.08 |
| 1387874_at | Dbp | D-box binding PAR bZIP transcription factor | 0.75 | 66.65 |
| 1376645_at | Medag | mesenteric estrogen-dependent adipogenesis | 3 | 62.21 |
| 1388525_at | Pik3ip1 | phosphoinositide-3-kinase interacting protein 1 | 0.75 | 55.48 |
| 1370510_a_at | Arntl | aryl hydrocarbon receptor nuclear translocator-like | 0.75 | 29.12 |
| 1373866_at | Coq10b | coenzyme Q10B | 2.25 | 72.18 |
| 1368511_at | Bhlhe41 | Basic helix-loop-helix domain containing, class B, 3 | 1.5 | 60.52 |
| 1368249_at | Klf15 | Kruppel-like factor 15 | 3 | 52.21 |
| 1389456_at | Clock | clock circadian regulator | 0.75 | 17.66 |
| 1370847_at | Spon2 | spondin 2 | 1.5 | 77.10 |
| 1368488_at | Nfil3 | nuclear factor, interleukin 3 regulated | 0 | 32.76 |
| 1387703_a_at | Usp2 | Ubiquitin-specific peptidase 2 | 0.75 | 45.30 |
| 1367632_at | Glul | Glutamate-ammonia | 5.25 | 67.90 |
| 1387669_a_at | Ephx1 | Epoxide hydrolase 1, | 1.5 | 44.76 |
| 1388384_at | Rusc2 | Dynein light chain LC8- type 1 | 2.25 | 68.63 |
| 1390391_at | Pfkfb3 | 6-phosphofructo-2-kinase/fructose-2,6-biphosphatase 3 | 2.25 | 46.37 |
| 1372341_at | Slc25a36 | solute carrier family 25 member 36 | 4.5 | 72.22 |
| 1390148_a_at | Zfp395 | zinc finger protein 395 | 1.5 | 65.82 |
| 1368025_at | Ddit4 | DNA-damage-inducible transcript 4 | 4.5 | 46.28 |
| 1369956_at | Ifngr1 | interferon gamma receptor 1 | 3 | 68.63 |
| 1373807_at | Vegfa | vascular endothelial growth factor A | 3.75 | 42.24 |
| 1388686_at | Rcan1 | Regulator of calcineurin 1 | 0.75 | 58.90 |
| 1388901_at | Fkbp5 | FK506 binding protein 5 | 6 | 46.23 |
| 1389836_a_at | Timp3 | TIMP metallopeptidase inhibitor 4 | 6.75 | 12.57 |
| 1388583_at | Cxcl12 | C-X-C motif chemokine ligand 12 | 5.25 | 22.03 |
| 1388924_at | Angptl4 | Anigopoietin-like 4 | 1.5 | 67.40 |
| 1371583_at | Rbm3 | RNA binding motif (RNP1, RRM) protein 3 | 2.25 | 32.70 |
| 1373266_at | Fam107a | family with sequence similarity 107, member A | 1.5 | 46.70 |
| 1374855_at | Per1 | period circadian clock 1 | 0.75 | 54.40 |
| 1371131_a_at | Txnip | thioredoxin interacting protein | 4.5 | 58.25 |
| 1367568_a_at | Mgp | matrix Gla protein | 0.75 | 54.46 |
| 1398365_at | Tppp3 | tubulin polymerization-promoting protein family member 3 | 1.5 | 68.93 |
| 1387074_at | Rgs2 | regulator of G-protein signaling 2 | 7.5 | 24.54 |
| 1386987_at | Il6r | Interleukin 6 receptor | 6.75 | 69.94 |
| 1373092_at | Tgfbr3 | transforming growth factor beta receptor 3 | 3 | 33.67 |
| 1368549_at | Hbp1 | HMG-box transcription factor 1 | 2.25 | 55.65 |
| 1374650_at | Nedd9 | neural precursor cell expressed, developmentally down-regulated 9 | 6 | 23.73 |
| 1370209_at | Klf9 | Kruppel-like factor 9 | 2.25 | 21.17 |
| 1370912_at | Hspa1a | Heat shock 70kD protein 1B | 3 | 64.53 |
| 1371754_at | Slc25a25 | solute carrier family 25 member 25 | 3.75 | 80.46 |
| 1390171_at | Fam76a | family with sequence similarity 76, member A | 5.25 | 31.50 |
| 1372701_at | Hsp90aa1 /// LOC100362895 /// LOC103692716 | heat shock protein 1, alpha-like | 4.5 | 49.57 |
| 1390042_at | Tmem140 | Transmembrane Protein 140 | 3.75 | 23.93 |
| 1367741_at | Herpud1 | homocysteine inducible ER protein with ubiquitin like domain 1 | 2.25 | 49.25 |
| 1373158_at | Gpr146 | G protein-coupled receptor 146 | 3.75 | 64.73 |
| 1372835_at | Rhoj | #N/A | 2.25 | 53.09 |
| 1372390_at | Peg3 | paternally expressed 3 | 6 | 58.28 |
| 1388795_at | Tppp | tubulin polymerization promoting protein | 6 | 32.29 |
| 1369919_at | Tef | TEF, PAR bZIP transcription factor | 3 | 13.80 |
| 1398370_at | Adarb1 | adenosine deaminase, RNA-specific, B1 | 2.25 | 73.02 |
| 1370954_at | P4ha1 | prolyl 4-hydroxylase subunit alpha 1 | 3.75 | 67.76 |
| 1368247_at | Hspa1a /// Hspa1b | heat shock 70kD protein 1A | 5.25 | 52.40 |
| 1383169_at | LOC103691479 | Leukemia inhibitory factor receptor ? | 8.25 | 63.74 |
| 1376924_a_at | Palmd | palmdelphin | 7.5 | 34.06 |
| 1392490_at | Tmem57 | Transmembrane protein 57 | 2.25 | 44.10 |
| 1367759_at | H1f0 | H1 histone family, member 0 | 4.5 | 41.64 |
| 1391808_at | Arrdc4 | arrestin domain containing 4 | 3.75 | 9.64 |
| 1387675_at | Plau | plasminogen activator, urokinase | 11.25 | 69.83 |
| 1399022_at | Clk1 | CDC-like kinase 1 | 3.75 | 65.10 |
| 1398255_at | Slc15a2 | solute carrier family 15 member 2 | 9.75 | 60.18 |
| 1398819_at | Dnaja1 | DNAJ (Hsp40) homolog, subfamily A, member 1 | 0.75 | 28.28 |
| 1387024_at | Dusp6 | dual specificity phosphatase 6 | 9 | 29.39 |
| 1375916_at | Pcmtd2 | protein-L-isoaspartate O-methyltransferase domain containing 2 | 0.75 | 62.02 |
| 1376435_at | Loxl4 | Lysyl Oxidase Like 4 | 2.25 | 54.70 |
| 1367922_at | Adam17 | ADAM metallopeptidase domain 17 | 3.75 | 36.47 |
| 1388672_at | Zcchc24 | zinc finger CCHC-type containing 24 | 3 | 58.93 |
| 1370381_at | Pnrc1 | Proline-rich nuclear receptor coactivator 1, F-box coactivator 1, F-box protein 11 | 1.5 | 32.35 |
| 1371832_at | Leo1 | LEO1 homolog, Paf1/RNA polymerase II complex component | 3 | 20.63 |
| 1372069_at | Kank1 | KN motif and ankyrin repeat domains 1 | 6 | 84.23 |
| 1370050_at | Atp2b1 | ATPase plasma membrane Ca2+ transporting 1 | 9.75 | 73.74 |
| 1389538_at | Nfkbia | NFKB inhibitor alpha | 1.5 | 49.85 |
| 1372520_at | Mcl1 | BCL2 family apoptosis regulator | 4.5 | 40.04 |
| 1367668_a_at | Scd2 | acyl-CoA desaturase 2-like | 3.75 | 33.06 |
| 1372426_at | Adamtsl4 | ADAMTS-like 4 | 4.5 | 51.36 |
| 1374421_at | Baz1b | bromodomain adjacent to zinc finger domain, 1B | 3.75 | 41.67 |
| 1370097_a_at | Cxcr4 | C-X-C motif chemokine receptor 4 | 3 | 26.73 |
| 1371913_at | Tgfbi | transforming growth factor, beta induced | 3 | 66.29 |
| 1373542_at | Sphk2 | sphingosine kinase 2 | 0.75 | 40.72 |
| 1391560_at | Hivep1 | human immunodeficiency virus type I enhancer binding protein 1 | 9 | 52.79 |
| 1368080_at | Rgcc | regulator of cell cycle | 3 | 28.28 |
| 1398998_at | RGD1309748 | -- | 2.25 | 77.46 |
| 1375658_at | Sash1 | SAM and SH3 domain containing 1 | 4.5 | 59.78 |
| 1377334_at | RT1-Ba | RT1 class II, locus Ba | 1.5 | 95.12 |
| 1368571_at | Clip2 | CAP-GLY domain containing linker protein 2 | 0.75 | 34.20 |
| 1373240_at | Dhrs3 | Dehydrogenase/reductase 3 | 3.75 | 29.42 |
| 1388949_at | Nxpe1 | neurexophilin and PC-esterase domain family, member 1 | 4.5 | 28.83 |
| 1370336_at | Osgin1 | oxidative stress induced growth inhibitor 1 | 4.5 | 54.02 |
| 1372750_at | Fst | Follistatin | 0.75 | 66.98 |
| 1367638_at | Mlycd | malonyl-CoA decarboxylase | 9.75 | 59.43 |
| 1370215_at | C1qb | complement C1q B chain | 2.25 | 9.48 |
| 1373407_at | Gramd4 | GRAM domain containing 4 | 0.75 | 40.40 |
| 1367771_at | Tsc22d3 | Glucocorticoid-induced leucine zipper | 3 | 64.84 |
| 1375037_at | Ppp6r3 | protein phosphatase 6, regulatory subunit 3 | 3 | 7.08 |
| 1384217_at | Zhx2 | zinc fingers and homeoboxes 2 | 4.5 | 44.28 |
| 1370399_at | Cyp4b1 | cytochrome P450, family 4, subfamily b, polypeptide 1 | 11.25 | 62.05 |
| 1370428_x_at | RT1-A2 /// RT1-A3 /// RT1-EC2 | RT1 class Ib, locus Aw2 | 0 | 97.02 |
| 1368304_at | Fmo3 | flavin containing monooxygenase 3 | 0.75 | 52.46 |
| 1371537_at | B4galt5 | beta-1,4-galactosyltransferase 5 | 4.5 | 63.98 |
| 1370570_at | Nrp1 | Neuropilin 1 | 0.75 | 51.43 |
| 1373087_at | March7 | membrane associated ring-CH-type finger 7 | 0.75 | 15.91 |
| 1370283_at | Hspa5 | heat shock protein family A member 5 | 3 | 42.87 |
| 1391062_at | Elp4 | elongator acetyltransferase complex subunit 4 | 3 | 25.26 |
| 1369182_at | F3 | coagulation factor III, tissue factor | 4.5 | 51.79 |
| 1369200_at | Nt5e | 5' nucleotidase, ecto | 9 | 64.55 |
| 1374671_at | Traf3ip2 | Traf3 interacting protein 2 | 3 | 45.71 |
| 1368394_at | Sfrp4 | secreted frizzled-related protein 4 | 3.75 | 68.08 |
| 1376770_at | Efhd1 | EF-hand domain family, member D1 | 2.25 | 48.29 |
| 1374575_at | Creb3l1 | cAMP responsive element binding protein 3-like 1 | 2.25 | 47.16 |
| 1367631_at | Ctgf | connective tissue growth factor | 1.5 | 40.47 |
| 1398431_at | Car8 | Carbonic anhydrase viii | 4.5 | 17.57 |
| 1369943_at | Tgm2 | Transglutaminase 2 | 2.25 | 62.24 |
| 1371237_a_at | Mt1a /// Ttr | metallothionein 1 | 1.5 | 76.88 |
| 1387060_at | Klf6 | Kruppel-like factor 6 | 5.25 | 36.41 |
| 1371029_at | Pkd1 | polycystic kidney disease 1 | 6.75 | 66.10 |

### Table K: Common genes retaining circadian expression Liver, Muscle, Adipose, and Lung. max Δφ indicates the maximum phase difference (in hours) and max ΔΑ the maximum amplitude difference (in % change) between two tissues (Equations 3,6).

| **Probe ID** | **Symbol** | **Gene name** | **max Δφ [hr]** | **Max ΔΑ [%]** |
| --- | --- | --- | --- | --- |
| 1371864_at | --- | Kruppel-like factor 9 | 2.25 | 95.64 |
| 1399082_at | --- | Transmembrane protein 33 | 4.5 | 100.00 |
| 1372136_at | --- | --- | 3 | 95.20 |
| 1376341_at | --- | ankyrin repeat domain 12 | 1.5 | 97.77 |
| 1390430_at | Nr1d2 | nuclear receptor subfamily 1, group D, member 2 | 0.75 | 87.96 |
| 1372701_at | Hsp90aa1 /// LOC100362895 /// LOC103692716 | heat shock protein 1, alpha-like | 6 | 98.16 |
| 1387703_a_at | Usp2 | Ubiquitin-specific peptidase 2 | 0.75 | 95.15 |
| 1387874_at | Dbp | D-box binding PAR bZIP transcription factor | 0.75 | 68.49 |
| 1371237_a_at | Mt1a /// Ttr | metallothionein 1 | 1.5 | 88.44 |
| 1370510_a_at | Arntl | aryl hydrocarbon receptor nuclear translocator-like | 0.75 | 70.73 |
| 1373866_at | Coq10b | coenzyme Q10B | 2.25 | 95.77 |
| 1373158_at | Gpr146 | G protein-coupled receptor 146 | 3.75 | 96.51 |
| 1386987_at | Il6r | Interleukin 6 receptor | 6.75 | 96.93 |
| 1368303_at | Per2 | period circadian clock 2 | 3 | 96.96 |
| 1370816_at | Nr1d1 | nuclear receptor subfamily 1, group D, member 1 | 1.5 | 83.45 |
| 1398819_at | Dnaja1 | DNAJ (Hsp40) homolog, subfamily A, member 1 | 3.75 | 99.12 |
| 1390199_at | Clock | clock circadian regulator | 3.75 | 85.47 |
| 1370381_at | Pnrc1 | Proline-rich nuclear receptor coactivator 1, F-box coactivator 1, F-box protein 11 | 1.5 | 85.86 |
| 1373542_at | Sphk2 | sphingosine kinase 2 | 2.25 | 88.91 |
| 1367741_at | Herpud1 | homocysteine inducible ER protein with ubiquitin like domain 1 | 2.25 | 97.38 |
| 1398998_at | RGD1309748 | -- | 2.25 | 98.11 |
| 1367771_at | Tsc22d3 | Glucocorticoid-induced leucine zipper | 3 | 98.75 |
| 1388901_at | Fkbp5 | FK506 binding protein 5 | 6.75 | 98.65 |
| 1367850_at | Fcgr2a | low affinity immunoglobulin gamma Fc region receptor III-like | 3 | 89.28 |
| 1370336_at | Osgin1 | oxidative stress induced growth inhibitor 1 | 11.25 | 97.69 |
| 1390042_at | Tmem140 | -- | 3.75 | 95.42 |
| 1387294_at | Sh3bp5 | SH3-domain binding protein 5 | 9.75 | 100.00 |
| 1368488_at | Nfil3 | nuclear factor, interleukin 3 regulated | 0 | 50.55 |
| 1370209_at | Klf9 | Kruppel-like factor 9 | 3.75 | 95.96 |
| 1388686_at | Rcan1 | Regulator of calcineurin 1 | 8.25 | 78.88 |
| 1368549_at | Hbp1 | HMG-box transcription factor 1 | 2.25 | 98.15 |
| 1368249_at | Klf15 | Kruppel-like factor 15 | 4.5 | 98.17 |
| 1371583_at | Rbm3 | RNA binding motif (RNP1, RRM) protein 3 | 2.25 | 98.22 |
| 1370283_at | Hspa5 | heat shock protein family A member 5 | 5.25 | 100.00 |
| 1371832_at | Leo1 | LEO1 homolog, Paf1/RNA polymerase II complex component | 3 | 80.70 |
| 1398431_at | Car8 | Carbonic anhydrase viii | 6.75 | 94.31 |
| 1367568_a_at | Mgp | matrix Gla protein | 0.75 | 98.79 |
| 1389538_at | Nfkbia | NFKB inhibitor alpha | 2.25 | 98.03 |
| 1372069_at | Kank1 | KN motif and ankyrin repeat domains 1 | 6 | 100.00 |
| 1369200_at | Nt5e | 5' nucleotidase, ecto | 9 | 98.84 |
| 1370847_at | Spon2 | spondin 2 | 1.5 | 96.46 |
| 1374421_at | Baz1b | bromodomain adjacent to zinc finger domain, 1B | 4.5 | 99.09 |
| 1375916_at | Pcmtd2 | protein-L-isoaspartate O-methyltransferase domain containing 2 | 2.25 | 98.74 |
| 1390171_at | Fam76a | family with sequence similarity 76, member A | 5.25 | 98.62 |
| 1369919_at | Tef | TEF, PAR bZIP transcription factor | 4.5 | 97.88 |
| 1368247_at | Hspa1a /// Hspa1b | heat shock 70kD protein 1A | 5.25 | 98.69 |
| 1368511_at | Bhlhe41 | Basic helix-loop-helix domain containing, class B, 3 | 1.5 | 97.11 |
| 1374855_at | Per1 | period circadian clock 1 | 1.5 | 96.88 |
| 1383169_at | LOC103691479 | Leukemia inhibitory factor receptor ? | 9 | 98.77 |
| 1377334_at | RT1-Ba | RT1 class II, locus Ba | 2.25 | 95.12 |
| 1369943_at | Tgm2 | Transglutaminase 2 | 2.25 | 86.99 |
| 1370570_at | Nrp1 | Neuropilin 1 | 4.5 | 98.72 |
| 1375138_at | Timp3 | TIMP metallopeptidase inhibitor 3 | 3 | 98.37 |
| 1376435_at | Loxl4 | Lysyl Oxidase Like 4 | 2.25 | 97.00 |
| 1372390_at | Peg3 | paternally expressed 3 | 10.5 | 98.07 |
| 1370215_at | C1qb | complement C1q B chain | 4.5 | 99.59 |
| 1388795_at | Tppp | tubulin polymerization promoting protein | 6 | 99.24 |
| 1399022_at | Clk1 | CDC-like kinase 1 | 7.5 | 98.98 |
| 1371754_at | Slc25a25 | solute carrier family 25 member 25 | 9 | 98.97 |
| 1373240_at | Dhrs3 | Dehydrogenase/reductase 3 | 9.75 | 98.73 |
| 1375658_at | Sash1 | SAM and SH3 domain containing 1 | 4.5 | 97.94 |
| 1387024_at | Dusp6 | dual specificity phosphatase 6 | 9.75 | 99.54 |
| 1387669_a_at | Ephx1 | Epoxide hydrolase 1, | 3.75 | 98.95 |
| 1368304_at | Fmo3 | flavin containing monooxygenase 3 | 4.5 | 99.01 |
| 1392490_at | Tmem57 | Transmembrane protein 57 | 6 | 99.53 |
| 1370912_at | Hspa1a | Heat shock 70kD protein 1B | 3 | 99.31 |

### Table L: Genes maintaining circadian expression in Liver.

| **ProbeID** | **Symbol** | **Gene name** | **Functional group** |
| --- | --- | --- | --- |
| 1390215_at | --- | --- | --- |
| 1371864_at | --- | --- | --- |
| 1374241_at | --- | --- | --- |
| 1373513_at | --- | --- | --- |
| 1389412_at | --- | --- | --- |
| 1375879_at | --- | --- | --- |
| 1382255_at | --- | --- | --- |
| 1399082_at | --- | --- | --- |
| 1379371_at | --- | --- | --- |
| 1374613_at | --- | --- | --- |
| 1372136_at | --- | --- | --- |
| 1373854_at | --- | --- | --- |
| 1372011_at | --- | --- | --- |
| 1375781_at | --- | --- | --- |
| 1398343_at | --- | --- | --- |
| 1389256_at | --- | --- | --- |
| 1380230_at | --- | --- | --- |
| 1388942_at | --- | --- | --- |
| 1377222_at | --- | --- | --- |
| AFFX_Rat_beta-actin_3_at | --- | --- | --- |
| 1375760_at | --- | --- | --- |
| 1371699_at | --- | --- | --- |
| 1372505_at | --- | --- | --- |
| 1374265_at | --- | --- | --- |
| 1372199_at | --- | --- | --- |
| 1371507_at | --- | --- | --- |
| 1388786_at | --- | --- | --- |
| 1374483_at | --- | --- | --- |
| 1382516_at | --- | --- | --- |
| 1374721_at | --- | --- | --- |
| 1375856_at | --- | --- | --- |
| 1390530_at | --- | --- | --- |
| 1388146_at | --- | --- | --- |
| 1371854_at | --- | --- | --- |
| 1374623_at | --- | --- | --- |
| 1374611_at | --- | --- | --- |
| 1371595_at | --- | --- | --- |
| 1375672_at | --- | --- | --- |
| 1377149_at | --- | --- | --- |
| 1371735_at | --- | --- | --- |
| 1372642_at | --- | --- | --- |
| 1371903_at | --- | --- | --- |
| 1389153_at | --- | --- | --- |
| 1391485_at | --- | --- | --- |
| 1373736_at | --- | --- | --- |
| 1399015_at | --- | --- | --- |
| 1376839_at | --- | --- | --- |
| 1374748_at | --- | --- | --- |
| 1370173_at | --- | --- | --- |
| 1368046_at | --- | --- | --- |
| 1371954_at | --- | --- | --- |
| 1372949_at | --- | --- | --- |
| 1372022_at | --- | --- | --- |
| 1388509_at | --- | --- | --- |
| 1388194_at | --- | --- | --- |
| 1371796_at | --- | --- | --- |
| 1380905_at | --- | --- | --- |
| 1375676_at | --- | --- | --- |
| AFFX_Rat_beta-actin_5_at | --- | --- | --- |
| AFFX_Rat_beta-actin_M_at | --- | --- | --- |
| 1373819_at | --- | --- | --- |
| 1372031_at | --- | --- | --- |
| 1372816_at | --- | --- | --- |
| 1394117_at | --- | --- | --- |
| 1376268_at | --- | --- | --- |
| 1367611_at | --- | --- | --- |
| 1383577_at | --- | --- | --- |
| 1373569_at | --- | --- | --- |
| 1372103_at | --- | --- | --- |
| 1383161_a_at | --- | --- | --- |
| 1389618_at | --- | --- | --- |
| 1373703_at | --- | --- | --- |
| 1375027_at | --- | --- | --- |
| 1390240_at | --- | --- | --- |
| 1390457_at | --- | --- | --- |
| 1373360_at | --- | --- | --- |
| 1372381_at | --- | --- | --- |
| 1374731_at | --- | --- | --- |
| 1379429_at | --- | --- | --- |
| 1375088_at | --- | --- | --- |
| 1388985_at | --- | --- | --- |
| 1388890_at | --- | --- | --- |
| 1373626_at | --- | --- | --- |
| 1377807_a_at | --- | --- | --- |
| 1394290_at | --- | --- | --- |
| 1390466_at | --- | --- | --- |
| 1382644_at | --- | --- | --- |
| 1372261_at | --- | --- | --- |
| 1375182_at | --- | --- | --- |
| 1373740_at | --- | --- | --- |
| 1382413_at | --- | --- | --- |
| 1372220_at | --- | --- | --- |
| 1377924_at | --- | --- | --- |
| 1373355_at | --- | --- | --- |
| 1390456_at | --- | --- | --- |
| 1388966_at | --- | --- | --- |
| 1375331_at | --- | --- | --- |
| 1374060_at | --- | --- | --- |
| 1372964_at | --- | --- | --- |
| 1389488_at | --- | --- | --- |
| 1376178_at | --- | --- | --- |
| 1376544_at | --- | --- | --- |
| 1373126_at | --- | --- | --- |
| 1390694_at | --- | --- | --- |
| 1376341_at | --- | --- | --- |
| 1385273_at | --- | --- | --- |
| 1376005_at | --- | --- | --- |
| 1375045_at | --- | --- | --- |
| 1390430_at | Nr1d2 | nuclear receptor subfamily 1, group D, member 2 | Transcription/Translation regulation |
| 1387109_at | Por | P450 (cytochrome) oxidoreductase | Small molecule metabolism |
| 1387336_at | Nat8 | N-Acetyltransferase Camello 4 | Small molecule metabolism |
| 1377192_a_at | Clpx | Caseinolytic peptidase X | Transcription/Translation regulation |
| 1372701_at | Hsp90aa1 /// LOC100362895 /// LOC103692716 | heat shock protein 1, alpha-like | Transcription/Translation regulation |
| 1371693_at | Ahsa1 | Activator of heat shock 90-kDa protein ATPase homolog 1 | Transcription/Translation regulation |
| 1387703_a_at | Usp2 | Ubiquitin-specific peptidase 2 | mRNA/Protein processing |
| 1387156_at | Hsd17b2 | 17-beta Hydroxysteroid dehydrogenase type 2 | Small molecule metabolism |
| 1373515_at | Lgalsl | Galectin-5 (RL-18) | Inflammation/Immune response |
| 1373718_at | Tubb2a | tubulin, beta 2A class 2a | Cell cycle/Apoptosis |
| 1371580_at | Erlin1 | SPFH domain family, member 1 | Transcription/Translation regulation |
| 1374531_at | Slc6a6 | solute carrier family 6 member 6 | Bile acid cholesterol |
| 1398240_at | Hspa8 /// LOC498426 /// LOC680121 /// LOC689955 | Heat shock protein 70 | Transcription/Translation regulation |
| 1372177_at | Mocs2 | molybdenum cofactor synthesis 2 | mRNA/Protein processing |
| 1387874_at | Dbp | D-box binding PAR bZIP transcription factor | Transcription/Translation regulation |
| 1371763_at | RGD1309534 | Unknown | Other |
| 1371237_a_at | Mt1a /// Ttr | metallothionein 1 | Inflammation/Immune response |
| 1370510_a_at | Arntl | aryl hydrocarbon receptor nuclear translocator-like | Transcription/Translation regulation |
| 1388750_at | Tfrc | transferrin receptor | Transport |
| 1386894_at | Hspd1 | heat shock protein family D member 1 | mRNA/Protein processing |
| 1386946_at | Cpt1a | Carnitine palmitoyltransferase I | Lipid Metabolism |
| 1388874_at | Mtss1 | Metastasis suppressor 1 | Cytoskeleton/ECM |
| 1388898_at | Hsph1 | heat shock protein family H (Hsp110) member 1 | Transcription/Translation regulation |
| 1398362_at | Notch2 | Transreg | Transcription/Translation regulation |
| 1372536_at | Adck3 | Chaperone, ABC1 activity of bc1 complex like | Transcription/Translation regulation |
| 1387228_at | Slc2a2 | Solute carrier family 2 A2 | Small molecule metabolism |
| 1387203_at | Gckr | Glucokinase regulatory protein | Small molecule metabolism |
| 1371976_at | Fam195a | Unknown | Other |
| 1371012_at | Hacl1 | 2-Hydroxyphytanoyl-CoA lyase | Lipid Metabolism |
| 1389844_at | Fkbp4 | FK506 binding protein 4 | mRNA/Protein processing |
| 1389014_at | Nampt | nicotinamide phosphoribosyltransferase | Transcription/Translation regulation |
| 1393915_at | Lpcat3 | lysophosphatidylcholine acyltransferase 3 | Metabolism |
| 1386918_a_at | Sigmar1 | Opioid receptor, sigma 1 | Transcription/Translation regulation |
| 1398282_at | Kynu | Kynureninase (L-kynurenine hydrolase) | Small molecule metabolism |
| 1387848_at | Hmgcr | 3-hydroxy-3-methylglutaryl-CoA reductase | Transport |
| 1371418_at | Cct2 | Chaperonin containing TCP1, subunit 2 (beta) | Cell cycle/Apoptosis |
| 1372844_at | Efna1 | Ephrin A1 | Signaling |
| 1368692_a_at | Chka | choline kinase alpha | Metabolism |
| 1375336_at | Hsp90ab1 | Heat shock 90-kDa protein 1, beta | Transcription/Translation regulation |
| 1388271_at | Mt2A | metallothionein 2A | Metabolism |
| 1387312_a_at | Gck | glucokinase | Metabolism |
| 1389199_at | RGD1309079 | similar to Ab2-095 | -- |
| 1373108_at | Ppp1r3c | protein phosphatase 1, regulatory subunit 3C | Metabolism |
| 1389377_at | Insig2 | Insulin-induced gene 2 | Lipid Metabolism |
| 1368426_at | Crot | Carnitine octanoyltransferase | Lipid Metabolism |
| 1367838_at | Cth /// LOC103691744 | CTL target antigen (Cth) | Other |
| 1370928_at | Litaf | lipopolysaccharide-induced TNF factor | Transcription/Translation regulation |
| 1388629_at | Impdh2 | Inosine 5-monophosphate dehydrogenase 2 | Cell cycle/Apoptosis |
| 1376570_at | Cct5 | chaperonin containing TCP1 subunit 5 | mRNA/Protein processing |
| 1389408_at | LOC100359539 /// LOC103694971 /// Rrm2 | Ribonucleotide reductase M2 | Cell cycle/Apoptosis |
| 1374765_at | Bdh1 | 3-Hydroxybutyrate dehydrogenase, type 1 | Mitochondrial |
| 1373866_at | Coq10b | coenzyme Q10B | -- |
| 1386898_at | Hspe1 | heat shock protein family E member 1 | Mitochondrial |
| 1387361_s_at | Pgk1 | Phosphoglycerate kinase 1 | Small molecule metabolism |
| 1367671_at | Pcna | proliferating cell nuclear antigen | Cell cycle/Apoptosis |
| 1369635_at | Sord | sorbitol dehydrogenase | Metabolism |
| 1373986_at | Fnip2 | Tumor necrosis factor superfamily, member 11 | Inflammation/Immune response |
| 1388850_at | Hsp90aa1 /// LOC103692716 /// LOC680491 | heat shock protein 90, alpha (cytosolic), class A member 1 | mRNA/Protein processing |
| 1377821_at | Erbb3 | erb-b2 receptor tyrosine kinase 3 | Signaling |
| 1398903_at | Esd | esterase D | Other |
| 1368497_at | Abcc2 | ATP binding cassette subfamily C member 2 | Transport |
| 1373158_at | Gpr146 | G protein-coupled receptor 146 | -- |
| 1371505_at | Hnrnpc /// LOC100911576 | heterogeneous nuclear ribonucleoproteins C1/C2-like | Transcription/Translation regulation |
| 1390672_at | Rprm | Reprimo | Cell cycle/Apoptosis |
| 1370163_at | Odc1 | Ornithine decarboxylase 1 | Cell cycle/Apoptosis |
| 1367982_at | Alas1 | Aminolevulinic acid synthase 1 | Mitochondrial |
| 1367867_at | Gfer | Augmenter of liver regeneration | Cell cycle/Apoptosis |
| 1371403_at | Cct3 | Chaperonin subunit 3 (gamma) | Transcription/Translation regulation |
| 1386987_at | Il6r | Interleukin 6 receptor | Inflammation/Immune response |
| 1376788_at | Dapk1 | Death-associated protein kinase 1 | Cell cycle/Apoptosis |
| 1368563_at | Aspa | Aspartoacylase | Metabolism |
| 1388662_at | Rora | RAR related orphan receptor a | Transcription/Translation regulation |
| 1368303_at | Per2 | period circadian clock 2 | Transcription/Translation regulation |
| 1370816_at | Nr1d1 | nuclear receptor subfamily 1, group D, member 1 | Transcription/Translation regulation |
| 1383160_at | Chordc1 | Cysteine and histidine-rich domain (CHORD)-containing, zinc-bp1 | Transcription/Translation regulation |
| 1370538_at | Lama3 | laminin subunit alpha 3 | -- |
| 1368087_a_at | Ptpn21 | protein tyrosine phosphatase, non-receptor type 21 | Cytoskeleton/ECM |
| 1373963_at | Hdhd3 | haloacid dehalogenase-like hydrolase domain containing 3 | -- |
| 1372752_at | LOC100911766 /// Tspan4 | tetraspanin-4-like | Signaling |
| 1387221_at | Gch1 | GTP cyclohydrolase 1 | Metabolism |
| 1398877_at | Stip1 | stress-induced phosphoprotein 1 | Transcription/Translation regulation |
| 1374636_at | Jade1 | PHD finger protein 17 | Cell cycle/Apoptosis |
| 1372755_at | Mal2 | mal, T-cell differentiation protein 2 | Bile acid cholesterol |
| 1377016_at | Creld2 | cysteine-rich with EGF-like domains 2 | Inflammation/Immune response |
| 1368266_at | Arg1 | arginase 1 | Metabolism |
| 1370414_at | Rab38 | RAB38, member RAS oncogene family | Transport |
| 1390383_at | Plin2 | perilipin 2 | -- |
| 1389384_at | Ndufaf4 | Hormone-regulated proliferation-associated protein 20 | Cell cycle/Apoptosis |
| 1373047_at | Prkci | protein kinase C, iota | Signaling |
| 1368435_at | Cyp8b1 | cytochrome P450 family 8 subfamily B member 1 | Metabolism |
| 1388960_at | Ppa1 /// Sar1a | secretion associated, Ras related GTPase 1A | Transport |
| 1371467_at | l7Rn6 | Hikeshi, heat shock protein nuclear import factor | Inflammation/Immune response |
| 1375941_at | Baiap2l1 | BAI1-associated protein 2-like 1 | Cytoskeleton/ECM |
| 1388722_at | Dnajb1 | DnaJ heat shock protein family member B1 | Metabolism |
| 1373842_at | Wasl | Wiskott-Aldrich syndrome-like | Signaling |
| 1398819_at | Dnaja1 | DNAJ (Hsp40) homolog, subfamily A, member 1 | Transcription/Translation regulation |
| 1373312_at | Pnkd | paroxysmal nonkinesigenic dyskinesia | Other |
| 1390199_at | Clock | clock circadian regulator | Transcription/Translation regulation |
| 1372264_at | Pck1 | phosphoenolpyruvate carboxykinase 1 | Mitochondrial |
| 1377048_at | Tkfc | triokinase and FMN cyclase | Metabolism |
| 1376990_at | Khnyn | KH and NYN domain containing | -- |
| 1388348_at | Elovl5 | ELOVL family member 5,elongation of long chain fatty acids | Lipid Metabolism |
| 1370381_at | Pnrc1 | Proline-rich nuclear receptor coactivator 1, F-box coactivator 1, F-box protein 11 | Transcription/Translation regulation |
| 1368275_at | Msmo1 | Sterol-C4-methyl oxidase-like | Bile acid cholesterol |
| 1398960_at | Cct6a | chaperonin containing TCP1 subunit 6A | mRNA/Protein processing |
| 1373542_at | Sphk2 | sphingosine kinase 2 | Cell cycle/Apoptosis |
| 1388506_at | Dsp | desmoplakin | mRNA/Protein processing |
| 1390731_at | Rasgef1b | Rasgef domain family, 1b | Signaling |
| 1370062_at | Higd1a | HIG1 domain family, member 1A | Transcription/Translation regulation |
| 1374570_at | Agpat2 | 1-Acylglycerol-3-phosphate O- acyltransferase 2 | Lipid Metabolism |
| 1372452_at | Gpam | glycerol-3-phosphate acyltransferase, mitochondrial | Metabolism |
| 1372524_at | Lpin1 | lipin 1 | -- |
| 1376572_a_at | Svil | Supervillin | Cytoskeleton/ECM |
| 1372056_at | Cmtm6 | CKLF-like MARVEL transmembrane domain containing 6 | Inflammation/Immune response |
| 1387793_at | Slc9a3r1 | Solute carrier family 9 (sodium/ hydrogen exchanger) isoform 3 regulator 1 | Cytoskeleton/ECM |
| 1370045_at | Polg | DNA polymerase gamma, catalytic subunit | Mitochondrial |
| 1376868_at | Cobll1 | Cobl-like 1(Cordon-bleu) | Other |
| 1390272_at | Dph5 | diphthamide biosynthesis 5 | -- |
| 1367741_at | Herpud1 | homocysteine inducible ER protein with ubiquitin like domain 1 | mRNA/Protein processing |
| 1388642_at | Ei24 | Progesterone receptor membrane component 2 | Cell cycle/Apoptosis |
| 1387017_at | Sqle | Squalene epoxidase | Bile acid cholesterol |
| 1372602_at | Stbd1 | Genethonin 1 | Small molecule metabolism |
| 1370334_at | Plekhb1 | pleckstrin homology domain containing B1 | Other |
| 1388150_at | Xpo1 | exportin 1 | Transport |
| 1372739_at | Tspan31 | tetraspanin 31 | -- |
| 1371840_at | S1pr1 | sphingosine-1-phosphate receptor 1 | Signaling |
| 1375933_at | Cldn2 | Claudin 2 | Bile acid cholesterol |
| 1390172_at | Dhtkd1 | Dehydrogenase E1 and | Small molecule metabolism |
| 1398998_at | RGD1309748 | -- | -- |
| 1368316_at | Aqp8 | aquaporin 8 | Transport |
| 1367575_at | Eno1 | enolase 1 | Metabolism |
| 1373677_at | Slc39a10 | solute carrier family 39 member 10 | Cell cycle/Apoptosis |
| 1371251_at | Galt | Galactose-1-phosphate uridyltransferase | Small molecule metabolism |
| 1398749_at | Rpl4 | Ribosomal protein L4 | Transcription/Translation regulation |
| 1368376_at | Nr0b2 | nuclear receptor subfamily 0, group B, member 2 | Transcription/Translation regulation |
| 1372004_at | Hebp1 | Heme binding protein 1 | Inflammation/Immune response |
| 1372318_at | LOC102549542 | ELOVL family member 6 | Lipid Metabolism |
| 1372156_at | Tmem97 | transmembrane protein 97 | Metabolism |
| 1398246_s_at | Fcgr2a | low affinity immunoglobulin gamma Fc region receptor III-like | Inflammation/Immune response |
| 1369467_a_at | Pfkfb1 | 6-Phosphofructo-2-kinase/fructose-2,6-bisphosphatase 1 | Small molecule metabolism |
| 1389069_at | Rnf8 | ring finger protein 8 | Signaling |
| 1372966_at | Mfsd2a | major facilitator superfamily domain containing 2A | -- |
| 1389355_at | Ier5 | immediate early response 5 | Cell cycle/Apoptosis |
| 1399058_at | Mrpl18 | Mitochondrial ribosomal protein L18 | Mitochondrial |
| 1374709_at | Hlf | Hepatic leukemia factor | Transcription/Translation regulation |
| 1373668_at | LOC103690054 /// Polr2i | Polymerase (RNA) II polypeptide I | Transcription/Translation regulation |
| 1368536_at | Enpp2 | ectonucleotide pyrophosphatase/phosphodiesterase 2 | Cytoskeleton/ECM |
| 1390117_at | Ypel2 | Yippee-like 2 | Cell cycle/Apoptosis |
| 1387905_at | Dnajc12 | DnaJ heat shock protein family member C12 | -- |
| 1399159_a_at | Vamp3 | vesicle-associated membrane protein 3 | Transport |
| 1371833_at | Bri3 | brain protein I3 | -- |
| 1387856_at | Cnn3 | Calponin 3, acidic | Cytoskeleton/ECM |
| 1371549_at | Dcaf11 | DDB1 and CUL4 associated factor 11 | Cell cycle/Apoptosis |
| 1373625_at | Shmt1 | Serine hydroxymethyl transferase 1 (soluble) | Cell cycle/Apoptosis |
| 1371498_at | Aimp2 | tRNA synthetase cofactor p38 | Transcription/Translation regulation |
| 1371327_a_at | Actg1 /// LOC100361457 /// Potef | actin, gamma 1 | -- |
| 1375224_at | Phlda3 | pleckstrin homology-like domain, family A, member 3 | Signaling |
| 1386960_at | Slc37a4 | Solute carrier family 37 member 4 | Lipid Metabolism |
| 1387470_at | Cldn1 | Claudin 1 | Bile acid cholesterol |
| 1373309_at | Tmem86a | transmembrane protein 86A | -- |
| 1390381_at | Xpc | Xeroderma pigmentosum, | Cell cycle/Apoptosis |
| 1371853_at | LOC103691922 /// Mrpl42 | mitochondrial ribosomal protein L42 | Mitochondrial |
| 1387388_at | Chp1 /// RGD1564956 /// RGD1565588 | similar to calcium binding protein P22 | -- |
| 1367623_at | Rpl18 | ribosomal protein L18 | mRNA/Protein processing |
| 1374251_at | Kcnj15 | potassium voltage-gated channel subfamily J member 15 | Bile acid cholesterol |
| 1377375_at | Aass | Aminoadipate-semialdehyde synthase | Small molecule metabolism |
| 1367754_s_at | Asl | argininosuccinate lyase | Metabolism |
| 1368168_at | Slc34a2 | solute carrier family 34 member 2 | Transport |
| 1386927_at | Cpt2 | Carnitine palmitoyltransferase 2 | Lipid Metabolism |
| 1376168_at | Mmgt1 | membrane magnesium transporter 1 | Transport |
| 1386981_at | Slc16a1 | solute carrier family 16 member 1 | Metabolism |
| 1371031_at | Mat1a | Methionine adenosyltransferase I, | Small molecule metabolism |
| 1372401_at | Nans | N-acetylneuraminate synthase | Metabolism |
| 1370663_at | Wee1 | WEE1 G2 checkpoint kinase | Cell cycle/Apoptosis |
| 1371953_at | Ccng2 | cyclin G2 | Cell cycle/Apoptosis |
| 1367771_at | Tsc22d3 | Glucocorticoid-induced leucine zipper | Transcription/Translation regulation |
| 1389308_at | Dnajb11 | DnaJ heat shock protein family (Hsp40) member B11 | mRNA/Protein processing |
| 1367834_at | LOC100912604 /// Srm | spermidine synthase | Other |
| 1370325_at | Gorasp2 /// LOC103690018 | golgi reassembly stacking protein 2 | -- |
| 1388901_at | Fkbp5 | FK506 binding protein 5 | Transcription/Translation regulation |
| 1371074_a_at | Mcm6 | minichromosome maintenance complex component 6 | Metabolism |
| 1398950_at | Mlec /// Unc119b | Sciellin | Other |
| 1371454_at | Emc6 | ER membrane protein complex subunit 6 | -- |
| 1389420_at | Stap2 | Signal-transducing adaptor protein 2 | Transcription/Translation regulation |
| 1369270_at | Nr1i2 | Nuclear receptor subfamily 1, | Transcription/Translation regulation |
| 1371684_at | Pelo | pelota mRNA surveillance and ribosome rescue factor | Cell cycle/Apoptosis |
| 1373967_at | Sub1 | SUB1 homolog, transcriptional regulator | Transcription/Translation regulation |
| 1372051_at | Rhbdd2 | NOTFOUND | -- |
| 1371768_at | Ssu72 | SSU72 homolog, RNA polymerase II CTD phosphatase | Transcription/Translation regulation |
| 1367850_at | Fcgr2a | low affinity immunoglobulin gamma Fc region receptor III-like | Inflammation/Immune response |
| 1387105_at | Zfp422 | zinc finger protein 422 | Transcription/Translation regulation |
| 1368365_at | Aldh3a2 | Alcohol/aldehyde dehydrogenase family 3, subfamily A2 | Lipid Metabolism |
| 1370277_at | Slc25a3 | solute carrier family 25 member 3 | Transport |
| 1372715_at | Sfxn1 | Liver tricarboxylate carrier, mitochondrial | Mitochondrial |
| 1371805_at | Ankrd46 | ankyrin repeat domain 46 | Transcription/Translation regulation |
| 1368642_at | Cdh2 | cadherin 2 | Cytoskeleton/ECM |
| 1386895_at | Maged1 | MAGE family member D1 | Cell cycle/Apoptosis |
| 1390249_at | RGD1305464 | similar to human chromosome 15 open reading frame 39 | -- |
| 1375924_at | Fabp12 /// LOC103690427 | fatty acid binding protein 12 | -- |
| 1377379_at | Irf6 | interferon regulatory factor 6 | Inflammation/Immune response |
| 1371363_at | Gpd1 | Glycerol-3-phosphate dehydrogenase 1 (soluble) | Lipid Metabolism |
| 1374914_at | Ppard | peroxisome proliferator-activated receptor delta | Transcription/Translation regulation |
| 1372832_at | Stx2 | syntaxin 2 | Transport |
| 1368073_at | Irf1 | interferon regulatory factor 1 | Inflammation/Immune response |
| 1393516_at | Slc16a12 | Solute carrier family 16, member | Small molecule metabolism |
| 1377037_at | Acot4 | acyl-CoA thioesterase 4 | Metabolism |
| 1390107_at | Sytl2 | Synaptotagmin-like 2 | Transcription/Translation regulation |
| 1371542_at | Tuba4a | tubulin, alpha 4A | Cell cycle/Apoptosis |
| 1371524_at | Cfap20 | Trap locus 3, transcription factor IIB-like | Transcription/Translation regulation |
| 1388340_at | Ns5atp9 | PCNA clamp associated factor | Transcription/Translation regulation |
| 1388645_at | Ola1 | Obg-like ATPase 1 | Other |
| 1374903_at | Gcnt2 | beta-1,6- Golgi, glycoprotein synthesis Acetylglucosaminyltransferase family polypeptide 3 | Transcription/Translation regulation |
| 1398800_at | Ywhab | tyrosine 3-monooxygenase/tryptophan 5-monooxygenase activation protein, beta | Signaling |
| 1398296_at | Gde1 | glycerophosphodiester phosphodiesterase 1 | Metabolism |
| 1389169_at | Pgrmc2 | Progesterone receptor membrane component 3 | Signaling |
| 1371752_at | Rangrf | RAN guanine nucleotide release factor | -- |
| 1377921_at | Etnk2 | Ethanolamine kinase 2 | Lipid Metabolism |
| 1389113_at | Slc45a3 | solute carrier family 45, member 3 | Other |
| 1367618_a_at | Gnb2l1 | Receptor For Activated C Kinase 1 | Signaling |
| 1398349_at | Ak2 | Adenylate kinase 2 | Mitochondrial |
| 1372600_at | Fbxo31 | F-box protein 31 | Cell cycle/Apoptosis |
| 1376427_a_at | Gldc | Glycine decarboxylase | Mitochondrial |
| 1368486_at | Irs3 | insulin receptor substrate 3 | Signaling |
| 1368068_a_at | Pacsin2 | Protein kinase C and casein kinase substrate 2 | Cytoskeleton/ECM |
| 1372352_at | Manf | mesencephalic astrocyte-derived neurotrophic factor | Cell cycle/Apoptosis |
| 1374625_at | Hes6 | hes family bHLH transcription factor 6 | Transcription/Translation regulation |
| 1370336_at | Osgin1 | oxidative stress induced growth inhibitor 1 | Cell cycle/Apoptosis |
| 1372827_at | Ppid | peptidylprolyl isomerase D | mRNA/Protein processing |
| 1372437_at | Skp1 | S-phase kinase-associated protein 1 | mRNA/Protein processing |
| 1371987_at | Papd7 | poly RNA polymerase D7, non-canonical | Cell cycle/Apoptosis |
| 1387127_at | Atrn | attractin | -- |
| 1371469_at | Chp1 | calcineurin-like EF-hand protein 1 | Signaling |
| 1372847_at | Sdhaf3 | succinate dehydrogenase complex assembly factor 3 | Metabolism |
| 1372920_at | Prodh | proline dehydrogenase 1 | Metabolism |
| 1371652_at | Ccdc50 | coiled-coil domain containing 50 | Signaling |
| 1368769_at | Abcb11 | ABC transport protein, sub-family B, member 11 | Bile acid cholesterol |
| 1390042_at | Tmem140 | -- | Other |
| 1374753_at | Papd4 | PAP-associated domain containing 4 | Transcription/Translation regulation |
| 1373730_at | Rbm33 | RNA binding motif protein 33 | Transcription/Translation regulation |
| 1368460_at | Slc2a5 | Solute carrier family 2, member 5 | Small molecule metabolism |
| 1368091_at | Oplah | 5-oxoprolinase (ATP-hydrolysing) | Metabolism |
| 1376709_at | Slc39a8 | Solute carrier family 39 (metal ion transporter), member 8 | Other |
| 1374420_at | Apmap | adipocyte plasma membrane associated protein | Metabolism |
| 1375892_at | Elavl1 | ELAV like RNA binding protein 1 | Transcription/Translation regulation |
| 1390692_at | Ctps1 | CTP synthase 1 | Metabolism |
| 1389301_at | Mbnl2 | Muscle blind-like 2 isoform 1 | Transcription/Translation regulation |
| 1367590_at | Ran | RAN, member RAS oncogene family | Signaling |
| 1388410_at | Ugp2 | UDP-glucose pyrophosphorylase 2 | Metabolism |
| 1373451_at | Rnaseh2b | ribonuclease H2, subunit B | Transcription/Translation regulation |
| 1370811_at | Mpst | mercaptopyruvate sulfurtransferase | Other |
| 1370991_at | Cml3 | probable N-acetyltransferase | Transcription/Translation regulation |
| 1388331_at | Hsp90b1 | Heat shock protein 90-kDa beta | Transcription/Translation regulation |
| 1382028_at | Pex19 | peroxisomal biogenesis factor 19 | mRNA/Protein processing |
| 1371770_at | Pfdn6 | MHC class II region-expressed gene KE2 | Inflammation/Immune response |
| 1373929_at | Mrps7 | mitochondrial ribosomal protein S7 | Mitochondrial |
| 1387062_a_at | Chek1 /// Stt3a | Checkpoint kinase 1 | Cell cycle/Apoptosis |
| 1389738_at | Ung | Uracil-DNA glycosylase | Cell cycle/Apoptosis |
| 1369150_at | Pdk4 | pyruvate dehydrogenase kinase 4 | -- |
| 1371539_at | LOC103690013 /// Nhp2 | NHP2 ribonucleoprotein | mRNA/Protein processing |
| 1387892_at | Tubb5 | tubulin, beta 5 class I | mRNA/Protein processing |
| 1387244_at | Cgrrf1 | Cgr19 | Cell cycle/Apoptosis |
| 1373870_at | Fam98a | Family with sequence similarity 98, member A | Other |
| 1375296_at | Snx3 | sorting nexin 3 | Transport |
| 1371634_at | Tmem126a | transmembrane protein 126A | Mitochondrial |
| 1389658_at | Nsun2 | NOL1/NOP2/Sun domain family, | Cell cycle/Apoptosis |
| 1367521_at | Edc3 | enhancer of mRNA decapping 3 | mRNA/Protein processing |
| 1369937_at | Calm1 | calmodulin 1 | Signaling |
| 1370319_at | Ppif | peptidylprolyl isomerase F | Cell cycle/Apoptosis |
| 1368294_at | Dnase1l3 | DNase gamma; deoxyribonuclease I-like 3 | Cell cycle/Apoptosis |
| 1387963_a_at | Uox | urate oxidase | Metabolism |
| 1371372_at | Ptges3 | Prostaglandin E synthase 3 | Transcription/Translation regulation |
| 1375686_at | Ppil3 | peptidylprolyl isomerase like 3 | mRNA/Protein processing |
| 1387981_at | Olr59 | olfactory receptor 59 | -- |
| 1372871_at | Cnppd1 | cyclin Pas1/PHO80 domain containing 1 | -- |
| 1373024_at | Ap3s1 | Adaptor-related protein complex 3, sigma 1 subunit | Vesicular transport |
| 1388471_at | Tcp11l2 | t-complex 11 like 2 | Signaling |
| 1388395_at | G0s2 | G0/G1switch 2 | Cell cycle/Apoptosis |
| 1388659_at | Carhsp1 | Calcium-regulated heat stable protein 1 | Signaling |
| 1370642_s_at | Pdgfrb | platelet derived growth factor receptor beta | Signaling |
| 1399005_at | Ppp2r5a | Protein phosphatase 2, regulatory subunit B | Signaling |
| 1387852_at | Thrsp | Thyroid hormone-responsive protein | Lipid Metabolism |
| 1374176_at | Lurap1l | -- | -- |
| 1373282_at | Slc25a33 | solute carrier family 25 member 33 | Mitochondrial |
| 1372688_at | Exosc7 | Exosome component 7 | Transcription/Translation regulation |
| 1373864_at | Map4k4 | mitogen-activated protein kinase kinase kinase kinase 4(Map4k4) Rattus | Signaling |
| 1387294_at | Sh3bp5 | SH3-domain binding protein 5 | Mitochondrial |
| 1374522_at | Mia3 | MIA family member 3, ER export factor | Transport |
| 1370024_at | Fabp7 | fatty acid binding protein 7 | Metabolism |
| 1372533_at | Edem1 | ER degradation enhancer mannosidase alpha-like 1 | Transcription/Translation regulation |
| 1371519_at | Etfdh | Electron-transferring-flavoprotein dehydrogenase | Mitochondrial |
| 1367790_at | Snd1 | staphylococcal nuclease and tudor domain containing 1 | Metabolism |
| 1392929_at | RGD1565616 | -- | -- |
| 1383188_at | Ppara | peroxisome proliferator activated receptor alpha | Metabolism |
| 1370818_at | Decr2 /// Nme4 | 2,4-dienoyl-CoA reductase 2 | Metabolism |
| 1369864_a_at | Sds | serine dehydratase | Metabolism |
| 1374676_at | Sgms1 | Sphingomyelin Synthase 1 | Other |
| 1370019_at | Sult1a1 | sulfotransferase family 1A member 1 | Metabolism |
| 1389430_at | Hsd17b7 | Hydroxysteroid (17-beta) dehydrogenase 7 | Small molecule metabolism |
| 1368878_at | Idi1 | Isopentenyl-diphosphate delta isomerase | Bile acid cholesterol |
| 1387805_at | Bnip3 | BCL2/adenovirus E1B 19 kDa- | Cell cycle/Apoptosis |
| 1373015_at | Rnf11 | Ring finger protein 11 | Transcription/Translation regulation |
| 1371957_at | Imp4 | IMP4, U3 small nucleolar ribonucleoprotein | Transcription/Translation regulation |
| 1373274_at | Txnrd3 | thioredoxin reductase 3 | Metabolism |
| 1388422_at | Lims2 | LIM and senescent cell antigen like domains 2 | Cytoskeleton/ECM |
| 1388739_at | Ubr7 | ubiquitin protein ligase E3 component n-recognin 7 | mRNA/Protein processing |
| 1375775_at | Odf3b | Outer dense fiber of sperm tails 3 | Cytoskeleton/ECM |
| 1383075_at | Ccnd1 | cyclin D1 | Cell cycle/Apoptosis |
| 1367670_at | Fh | Fumarate hydratase | Mitochondrial |
| 1367808_at | Timm8b | translocase of inner mitochondrial membrane 8 homolog B | mRNA/Protein processing |
| 1376089_at | Ldlr | low density lipoprotein receptor | Metabolism |
| 1370184_at | Cfl1 | cofilin 1 | Transport |
| 1368226_at | Dnph1 | 2'-deoxynucleoside 5'-phosphate N-hydrolase 1 | Cell cycle/Apoptosis |
| 1369664_at | Avpr1a | arginine vasopressin receptor 1A | Signaling |
| 1398838_at | Rab7a | RAB7A, member RAS oncogene family | Transport |
| 1367683_at | Kpna2 /// LOC100359600 | karyopherin subunit alpha 2 | Transcription/Translation regulation |
| 1387780_at | Dnaja2 | DNAJ (Hsp40) homolog, subfamily A, member 2 | Transcription/Translation regulation |
| 1371640_at | Zfp706 | zinc finger protein 706 | -- |
| 1372170_at | Acy1 | aminoacylase 1 | Metabolism |
| 1368488_at | Nfil3 | nuclear factor, interleukin 3 regulated | Transcription/Translation regulation |
| 1369976_at | Dynll1 | Dynein, cytoplasmic, light chain 1 | Other |
| 1372141_at | Pfdn2 | Prefoldin subunit 2 | Transcription/Translation regulation |
| 1374159_at | Als2cl | ALS2 C-terminal like | mRNA/Protein processing |
| 1388819_at | Scamp1 | secretory carrier membrane protein 1 | Cytoskeleton/ECM |
| 1387375_at | Khk | ketohexokinase | Metabolism |
| 1370209_at | Klf9 | Kruppel-like factor 9 | Transcription/Translation regulation |
| 1372619_at | Mrpl49 | mitochondrial ribosomal protein L49 | -- |
| 1368458_at | Cyp7a1 | Cytochrome P450 (cholesterol hydroxylase 7 alpha) | Bile acid cholesterol |
| 1373469_at | Hacd3 | 3-hydroxyacyl-CoA dehydratase 3 | Metabolism |
| 1372331_at | Eif1ax | eukaryotic translation initiation factor 1A, X-linked | Transcription/Translation regulation |
| 1371475_at | Rnase4 | ribonuclease A family member 4 | Transcription/Translation regulation |
| 1388182_at | Prim1 | primase, DNA, polypeptide 1 | -- |
| 1371404_at | Eif4b | eukaryotic translation initiation factor 4B | Signaling |
| 1383080_at | Lamp2 | lysosomal-associated membrane protein 2 | Autophagy |
| 1370281_at | Fabp5 | fatty acid binding protein 5, epidermal | Metabolism |
| 1368223_at | Adamts1 | Adam metallopeptidase with thrombospondin 1 motif, 15 | Cytoskeleton/ECM |
| 1398994_at | Tpst2 | Protein-tyrosine sulfotransferase 2 | Transcription/Translation regulation |
| 1388108_at | Elovl6 /// LOC102549542 | Fatty acid elongase 2 | Lipid Metabolism |
| 1368049_at | Tcp1 | T-complex 1 | Transcription/Translation regulation |
| 1368051_at | Hsd17b12 | hydroxysteroid (17-beta) dehydrogenase 12 | Metabolism |
| 1371353_at | Sqstm1 | sequestosome 1 | Signaling |
| 1371329_at | Eif5a | eukaryotic translation initiation factor 5A | -- |
| 1371453_at | Farsb | phenylalanyl-tRNA synthetase, beta subunit | -- |
| 1370688_at | Gclc | glutamate-cysteine ligase, catalytic subunit | Metabolism |
| 1367983_at | Fen1 | Flap structure-specific | Cell cycle/Apoptosis |
| 1373858_at | Kpnb1 | karyopherin subunit beta 1 | Transport |
| 1371399_at | Tomm6 | translocase of outer mitochondrial membrane 6 | Transport |
| 1376765_at | Mro | maestro | -- |
| 1387865_at | Dut | deoxyuridine triphosphatase | Metabolism |
| 1371641_at | Cct7 | chaperonin containing TCP1 subunit 7 | Cell cycle/Apoptosis |
| 1367820_at | Banf1 | barrier to autointegration factor 1 | Other |
| 1371435_at | Naca | Nascent polypeptide-associated complex alpha polypeptide | Transcription/Translation regulation |
| 1368227_at | Slc28a2 | Solute carrier family 28 (sodium- coupled nucleoside transporter) a2 | Cell cycle/Apoptosis |
| 1367705_at | Glrx | glutaredoxin | Metabolism |
| 1371822_at | Polr3d | RNA polymerase III subunit D | Transcription/Translation regulation |
| 1388102_at | Ptgr1 | Dithiolethione-inducible gene-1 | Inflammation/Immune response |
| 1380262_at | Sgk2 | SGK2, serine/threonine kinase 2 | -- |
| 1373499_at | Gas5 | -- | -- |
| 1374870_at | Col27a1 | collagen type XXVII alpha 1 chain | -- |
| 1368328_at | Gys2 | Glycogen synthase 2 (liver) | Small molecule metabolism |
| 1368650_at | Klf10 | Kruppel-like factor 10 | Transcription/Translation regulation |
| 1388686_at | Rcan1 | Regulator of calcineurin 1 | Signaling |
| 1387949_at | Cyp2c22 | cytochrome P450, family 2, subfamily c, polypeptide 22 | Metabolism |
| 1369973_at | Xdh | xanthine dehydrogenase(Xdh) | -- |
| 1388185_at | Rb1 | RB transcriptional corepressor 1 | Cell cycle/Apoptosis |
| 1390577_at | Ranbp10 | RAN binding protein 10 | -- |
| 1372083_at | Polr2b | RNA polymerase II subunit B | Metabolism |
| 1370345_at | Ccnb1 | Cyclin B | Cell cycle/Apoptosis |
| 1367597_at | Rps8 | ribosomal protein S8 | Metabolism |
| 1389561_at | Tmem218 | -- | -- |
| 1367476_at | Srp14 | signal recognition particle 14 | Signaling |
| 1368549_at | Hbp1 | HMG-box transcription factor 1 | Transcription/Translation regulation |
| 1372157_at | Bola1 | bolA family member 1 | Mitochondrial |
| 1371916_at | Msrb1 | Selenoprotein X, 1 | Other |
| 1371636_at | Ankrd13c | ankyrin repeat domain 13C | -- |
| 1373043_at | Sdf2l1 | stromal cell-derived factor 2-like 1 | Metabolism |
| 1370821_at | Tpmt | thiopurine S-methyltransferase | -- |
| 1367540_at | Zfyve26 | zinc finger FYVE-type containing 26 | Cell cycle/Apoptosis |
| 1373093_at | Errfi1 | ERBB receptor feedback inhibitor 1 | -- |
| 1370280_at | Hprt1 /// LOC103689983 | hypoxanthine phosphoribosyltransferase 1 | Metabolism |
| 1374620_at | Ceacam1 | carcinoembryonic antigen related cell adhesion molecule 1 | Cytoskeleton/ECM |
| 1387271_at | Phyh | phytanoyl-CoA 2-hydroxylase | Metabolism |
| 1368249_at | Klf15 | Kruppel-like factor 15 | Metabolism |
| 1390021_at | Hist1h2bcl1 | histone cluster 1, H2bh | Transcription/Translation regulation |
| 1387365_at | Nr1h3 | nuclear receptor subfamily 1, group H, member 3 | Metabolism |
| 1371485_at | Sh3pxd2a | NOTFOUND | -- |
| 1388650_at | Top2a | topoisomerase (DNA) II alpha | Transcription/Translation regulation |
| 1371350_at | Mat2a /// RGD1560523 | methionine adenosyltransferase 2A | -- |
| 1367603_at | Tpi1 | triosephosphate isomerase 1 | Metabolism |
| 1387491_at | Gk | glycerol kinase | Metabolism |
| 1386900_at | Serp1 | Ribosome-associated membrane protein 4 | -- |
| 1369962_at | Atic /// LOC100910688 | bifunctional purine biosynthesis protein PURH-like | -- |
| 1373231_at | Otud5 | OTU deubiquitinase 5 | Inflammation/Immune response |
| 1375964_at | Psph | phosphoserine phosphatase | Metabolism |
| 1372308_at | St3gal1 | ST3 beta-galactoside alpha-2,3-sialyltransferase 1 | Metabolism |
| 1388424_at | Eif3j | Eukaryotic translation initiation factor 3, subunit 1 alpha | Transcription/Translation regulation |
| 1367798_at | Ahcy | adenosylhomocysteinase | Transport |
| 1370374_at | Steap3 | STEAP family member 3 | Cell cycle/Apoptosis |
| 1390343_at | Ccnc | cyclin C | Cell cycle/Apoptosis |
| 1399131_at | Mrps22 | mitochondrial ribosomal protein S22 | mRNA/Protein processing |
| 1371143_at | Serpina7 | serpin family A member 7 | Transport |
| 1377135_at | LOC102547797 | uncharacterized | -- |
| 1373243_at | Pmvk | phosphomevalonate kinase | Metabolism |
| 1388136_at | Timm9 | Translocase of inner mitochondrial membrane 9 | Transcription/Translation regulation |
| 1372364_a_at | LOC103690022 /// Ntan1 | N-terminal asparagine amidase | mRNA/Protein processing |
| 1398763_at | LOC100362432 /// Timm23 | translocase of inner mitochondrial membrane 23 | Mitochondrial |
| 1371888_at | mrpl24 | mitochondrial ribosomal protein L24 | Metabolism |
| 1386944_a_at | G6pc | glucose-6-phosphatase, catalytic subunit | Metabolism |
| 1373079_at | Smim14 | NOTFOUND | -- |
| 1376755_at | Rarb | retinoic acid receptor, beta | Transcription/Translation regulation |
| 1398341_at | Cisd3 | CDGSH iron sulfur domain 3 | -- |
| 1371774_at | Sat1 | spermidine/spermine N1-acetyl transferase 1 | -- |
| 1398790_at | LOC100362453 /// LOC103694903 /// Ppp2ca | protein phosphatase 2, catalytic subunit, alpha isozyme | Transport |
| 1373901_at | Cdk2ap2 | cyclin-dependent kinase 2 associated protein 2 | Cell cycle/Apoptosis |
| 1374987_at | Pank3 | pantothenate kinase 3 | Metabolism |
| 1386885_at | Ech1 | enoyl-CoA hydratase 1 | Metabolism |
| 1368336_at | Fdx1 | Ferredoxin 1 | Bile acid cholesterol |
| 1389681_at | Pvrl2 | Poliovirus receptor-related 2 | Cytoskeleton/ECM |
| 1375944_at | Acss2 | acyl-CoA synthetase short-chain family member 2 | Metabolism |
| 1371996_at | Aebp2 | AE binding protein 2 | -- |
| 1389965_at | Tgoln2 | Trans-Golgi Network Protein 2 | Transcription/Translation regulation |
| 1373722_at | Kif20a | kinesin family member 20A | Transport |
| 1388426_at | Srebf1 | sterol regulatory element binding transcription factor 1 | Transcription/Translation regulation |
| 1373195_at | Fus | Fused in sarcoma | mRNA/Protein processing |
| 1388497_at | Acot13 | acyl-CoA thioesterase 13 | Metabolism |
| 1387924_at | Ngef | neuronal guanine nucleotide exchange factor | -- |
| 1370314_at | Slc20a1 | solute carrier family 20 member 1 | Transport |
| 1386864_at | Pgam1 | phosphoglycerate mutase 1 | Metabolism |
| 1371564_at | Atp6v1e1 | ATPase, H + transporting, V1 subunit E isoform 1 | Other |
| 1388721_at | Hspb8 | heat shock protein family B member 8 | -- |
| 1373200_at | Eef1e1 | eukaryotic translation elongation factor 1 epsilon 1 | -- |
| 1369644_at | Adgrl2 | adhesion G protein-coupled receptor L2 | -- |
| 1388742_at | Bcl2l11 | BCL2 like 11 | Cell cycle/Apoptosis |
| 1389050_at | Dusp3 | dual specificity phosphatase 3 | mRNA/Protein processing |
| 1398882_at | Rps5 | ribosomal protein S5 | -- |
| 1367707_at | Fasn | fatty acid synthase | Metabolism |
| 1371598_at | Adipor2 | adiponectin receptor 2 | Metabolism |
| 1373383_at | Mterf3 | MTERF domain containing 1 | Mitochondrial |
| 1387027_a_at | Lgals9 | galectin 9 | Inflammation/Immune response |
| 1370299_at | Aldob | Aldolase B | Small molecule metabolism |
| 1374820_at | LOC691113 | l(3)mbt-like 4 (Drosophila) | Cell cycle/Apoptosis |
| 1386923_at | LOC103694902 /// Ube2b | ubiquitin-conjugating enzyme E2B | -- |
| 1370294_a_at | Cdc20 | cell division cycle 20 | Cell cycle/Apoptosis |
| 1375677_at | Tob2 | transducer of ERBB2, 2 | Metabolism |
| 1372907_at | Atp6v0e2 | ATPase, H+ transporting V0 subunit e2 | Transport |
| 1388103_at | Tmem37 | transmembrane protein 37 | Cytoskeleton/ECM |
| 1374939_at | Cyfip2 | Cytoplasmic FMR1-interacting protein 2 | Cell cycle/Apoptosis |
| 1370575_a_at | Azin1 | antizyme inhibitor 1 | -- |
| 1374798_at | LOC102547850 /// Tor1aip2 | torsin 1A interacting protein 2 | Other |
| 1388528_at | Fbl | Fibrillarin | Transcription/Translation regulation |
| 1389612_at | Rcbtb2 | RCC1 and BTB domain containing protein 2 | Cell cycle/Apoptosis |
| 1367567_at | Rpl6 | ribosomal protein L6 | Metabolism |
| 1371249_at | Xbp1 | X-box binding protein 1 | -- |
| 1370397_at | Cyp4a3 | cytochrome P450, family 4, subfamily a, polypeptide 3 | Metabolism |
| 1374010_at | Lig3 | DNA ligase 3 | Metabolism |
| 1390165_at | C2cd2 | C2 Calcium Dependent Domain Containing 2 | -- |
| 1375895_at | Tsr1 | TSR1, ribosome maturation factor | Metabolism |
| 1388484_at | Ube2c | Ubiquitin-conjugating enzyme E2C | Cell cycle/Apoptosis |
| 1384934_at | Slc41a2 | solute carrier family 41 member 2 | Transport |
| 1371437_at | Sec13 | SEC13 homolog, nuclear pore and COPII coat complex component | -- |
| 1389906_at | Fdft1 | farnesyl diphosphate farnesyl transferase 1 | -- |
| 1368877_at | Zfp354a | zinc finger protein 354A | -- |
| 1373011_at | Fam134b /// LOC103689968 /// LOC103690028 | family with sequence similarity 134, member B | -- |
| 1371583_at | Rbm3 | RNA binding motif (RNP1, RRM) protein 3 | Transcription/Translation regulation |
| 1370283_at | Hspa5 | heat shock protein family A member 5 | mRNA/Protein processing |
| 1383126_at | Akt1 | AKT Serine/Threonine Kinase 1 | -- |
| 1389480_at | Rwdd4 | RWD domain containing 4A | mRNA/Protein processing |
| 1371444_at | Cers2 | ceramide synthase 2 | Metabolism |
| 1387178_a_at | Cbs | cystathionine beta synthase | Metabolism |
| 1367599_at | Atp5g1 | ATP synthase, H+ transporting, mitochondrial Fo complex, subunit C1 | Metabolism |
| 1389587_at | Umps | uridine monophosphate synthetase | Metabolism |
| 1398319_at | Bmp2k | BMP-2 inducible kinase | -- |
| 1374752_at | Mdfic | Myod family inhibitor domain containing | Transcription/Translation regulation |
| 1367573_at | LOC100911372 /// Rps6 | ribosomal protein S6 | Signaling |
| 1392928_at | L3hypdh | Peroxisomal membrane protein 3 | Other |
| 1388325_at | Atp6v1d | ATPase, H + transporting, V1 subunit D | Other |
| 1372080_at | Immt | inner membrane mitochondrial protein | -- |
| 1386990_at | Ebp | emopamil binding protein | Metabolism |
| 1371445_at | Lrrc59 | Leucine-rich-repeat-protein superfamily | Transcription/Translation regulation |
| 1398807_at | Ppm1b | protein phosphatase, Mg2+/Mn2+ dependent, 1B | Inflammation/Immune response |
| 1370938_at | Pdxp | pyridoxal phosphatase | -- |
| 1398317_at | Bpnt1 | Bisphosphate 3’-nucleotidase 1 | Cell cycle/Apoptosis |
| 1389066_at | Rcan2 | regulator of calcineurin 2 | Transcription/Translation regulation |
| 1372890_at | Sgpl1 | Sphingosine-1-Phosphate Lyase 1 | -- |
| 1388365_at | Atp6v0d1 | ATPase H+ transporting V0 subunit D1 | Metabolism |
| 1372616_at | Trim36 | tripartite motif-containing 36 | -- |
| 1375215_x_at | Pgpep1 | Pyroglutamyl-peptidase I | Small molecule metabolism |
| 1389639_at | Shb | Protocadherin 1 (cadherin-like 1) | Cytoskeleton/ECM |
| 1372568_at | Epm2aip1 | EPM2A interacting protein 1 | Metabolism |
| 1393061_at | Ttc36 | tetratricopeptide repeat domain 36 | -- |
| 1387773_at | Cycs /// LOC100363502 | -- | -- |
| 1374747_at | Cdk14 | PFTAIRE protein kinase 1 | Cell cycle/Apoptosis |
| 1371730_at | Lonp2 | lon peptidase 2, peroxisomal | mRNA/Protein processing |
| 1387659_at | Gda | Guanine deaminase; EC 3.50.4.3 | Metabolism |
| 1372085_at | Atl2 | ADP-ribosylation factor-like 6-interacting protein 2 | Transcription/Translation regulation |
| 1398862_at | Atp2a2 | ATPase sarcoplasmic/endoplasmic reticulum Ca2+ transporting 2 | Signaling |
| 1372140_at | Ccdc28a | coiled-coil domain containing 28A | -- |
| 1398359_at | Rnf181 | ring finger protein 181 | Metabolism |
| 1376847_at | Marc1 | mitochondrial amidoxime reducing component 1 | Inflammation/Immune response |
| 1368674_at | Pygl | glycogen phosphorylase L | Metabolism |
| 1372601_at | Atf5 | activating transcription factor 5 | Transcription/Translation regulation |
| 1373777_at | Rgs16 | Regulator of G-protein signaling 16 | Signaling |
| 1372868_at | Tor3a | torsin family 3, member A | Metabolism |
| 1371970_at | Fam111a | family with sequence similarity 111, member A | -- |
| 1371969_at | Cald1 | Caldesmon 1 | Cytoskeleton/ECM |
| 1370865_at | Idh3g | isocitrate dehydrogenase 3 , gamma | -- |
| 1371832_at | Leo1 | LEO1 homolog, Paf1/RNA polymerase II complex component | Transcription/Translation regulation |
| 1369161_at | Abcb1a /// Abcb1b /// Abcb4 | ATP binding cassette subfamily B member 4 | Metabolism |
| 1372997_at | Gucd1 | guanylyl cyclase domain containing 1 | -- |
| 1371992_at | Babam1 | BRISC and BRCA1 A complex member 1 | -- |
| 1373026_at | Spc24 | SPC24, NDC80 kinetochore complex component | -- |
| 1372091_at | Mid1ip1 | MID1 interacting protein 1 | Cytoskeleton/ECM |
| 1375425_at | Ift20 | intraflagellar transport 20 | Transport |
| 1388516_at | Cdv3 | carnitine deficiency-associated gene expressed in ventricle 3 | -- |
| 1377353_a_at | Tnfsf13 | TNF superfamily member 13 | Inflammation/Immune response |
| 1370172_at | Sod2 | superoxide dismutase 2 | Signaling |
| 1368032_at | Nolc1 | nucleolar and coiled-body phosphoprotein 1 | -- |
| 1398937_at | Dhx15 | DEAH (Asp-Glu-Ala-His) box polypeptide 15 | Transcription/Translation regulation |
| 1390412_at | LOC100911874 /// Slc40a1 | solute carrier family 40 member 1 | Transport |
| 1368305_at | Casp6 | caspase 6 | Cell cycle/Apoptosis |
| 1374288_at | Ftsj3 | FtsJ homolog 3 (E. coli) | Transcription/Translation regulation |
| 1388244_s_at | Rpsa | RPSA ribosomal protein SA | mRNA/Protein processing |
| 1399013_at | Emc4 | ER membrane protein complex subunit 4 | -- |
| 1369896_s_at | Scaf8 | SR-related CTD-associated factor 8 | Transcription/Translation regulation |
| 1398386_at | Zcchc6 | zinc finger CCHC-type containing 6 | Cell cycle/Apoptosis |
| 1367755_at | Cdo1 | Cysteine dioxygenase, type i | Metabolism |
| 1371620_at | Prelid1 | PRELI domain containing 1 | Transport |
| 1398431_at | Car8 | Carbonic anhydrase viii | Other |
| 1373162_at | Tmem41a | Transmembrane protein 41a | Signaling |
| 1374508_at | LOC106631776 /// Nphp1 | nephrocystin 1 | -- |
| 1398755_at | Atp6v0c | ATPase H+ transporting V0 subunit C | Transport |
| 1374487_at | Fam96a | family with sequence similarity 96, member A | Metabolism |
| 1372132_at | Cndp2 | carnosine dipeptidase 2 | Other |
| 1372787_at | Utp3 | UTP3, small subunit processome component homolog | Metabolism |
| 1398875_at | Polr3k | RNA polymerase III subunit K | Metabolism |
| 1372230_at | Tmem147 | transmembrane protein 147 | -- |
| 1371945_at | Ube2l3 | ubiquitin-conjugating enzyme E2L 3 | Mitochondrial |
| 1388582_at | Psme3 | glucose-6-phosphatase, catalytic subunit | -- |
| 1373693_at | Gprc5c | G protein-coupled receptor, class C, group 5, member C | Signaling |
| 1374335_at | Gata6 | GATA Binding Protein 6 | -- |
| 1398906_at | LOC287274 /// Trappc2b | trafficking protein particle complex 2B | Cell cycle/Apoptosis |
| 1368036_at | Ptprf | -- | Signaling |
| 1390536_at | Cdon | Cell Adhesion Associated, Oncogene Regulated | -- |
| 1368552_at | Grpel1 | GrpE-like 1, mitochondrial | Metabolism |
| 1388361_at | Ndufb10 | NADH dehydrogenase (ubiquinone) 1 beta subcomplex, 10 | Mitochondrial |
| 1398844_at | Txn2 | thioredoxin 2 | Mitochondrial |
| 1368977_a_at | Timm10b | translocase of inner mitochondrial membrane 10B | mRNA/Protein processing |
| 1371873_at | Anp32e | Acidic (leucine-rich) nuclear phosphoprotein 32 family member E | Transcription/Translation regulation |
| 1367568_a_at | Mgp | matrix Gla protein | Cytoskeleton/ECM |
| 1368992_a_at | Srsf5 | serine and arginine rich splicing factor 5 | Metabolism |
| 1377143_at | Slc35b1 | solute carrier family 35, member B1 | Transport |
| 1376861_at | LOC686087 /// Mospd1 | Motile sperm domain containing 1 | EST/Unknown |
| 1374414_at | Fam134a | reticulophagy regulator family member 2 | -- |
| 1376700_at | Lima1 | LIM domain and actin binding 1 | Transport |
| 1388695_at | Shmt2 | serine hydroxymethyltransferase 2 | Metabolism |
| 1374072_at | Srsf1 | serine and arginine rich splicing factor 1 | Metabolism |
| 1367793_at | Ddt | D-dopachrome tautomerase | -- |
| 1371330_at | Rpl11 | ribosomal protein L11 | -- |
| 1367795_at | Ifrd1 | interferon-related developmental regulator 1 | Transcription/Translation regulation |
| 1398902_at | Cluh | MKIAA0664 protein | Other |
| 1398327_at | Fermt2 | fermitin family member 2 | -- |
| 1376288_at | Nudcd1 | NudC Domain Containing 1 | -- |
| 1372012_at | Dhcr24 | 24-Dehydrocholesterol Reductase | -- |
| 1372675_at | RGD1306954 | -- | -- |
| 1388134_at | Eef1d | eukaryotic translation elongation factor 1 delta | Transcription/Translation regulation |
| 1387665_at | Bhmt | betaine-homocysteine S-methyltransferase | Metabolism |
| 1369558_at | Inhbc | inhibin beta C subunit | Transcription/Translation regulation |
| 1388425_at | Oaf | out at first homolog | -- |
| 1398750_at | Calr | calreticulin | Metabolism |
| 1398896_at | Arcn1 | Archain 1 | Transcription/Translation regulation |
| 1374387_at | Arl6ip5 | ADP-ribosylation factor like GTPase 6 interacting protein 5 | Transport |
| 1389402_at | Csrnp1 | cysteine and serine rich nuclear protein 1 | Transcription/Translation regulation |
| 1371809_at | Mrps18b | mitochondrial ribosomal protein S18B | -- |
| 1368698_at | Atp2b2 | ATPase plasma membrane Ca2+ transporting 2 | Signaling |
| 1389988_at | Kctd2 | potassium channel tetramerization domain containing 2 | -- |
| 1388858_at | LOC103689956 /// Map2k3 | mitogen activated protein kinase kinase 3 | Inflammation/Immune response |
| 1389052_at | Ttc13 | tetratricopeptide repeat domain 13 | -- |
| 1390865_at | Cadps2 | calcium dependent secretion activator 2 | -- |
| 1374854_at | Pinx1 | PIN2/TERF1 interacting, telomerase inhibitor 1 | -- |
| 1373114_at | Dtx4 | deltex E3 ubiquitin ligase 4 | Signaling |
| 1371300_at | Rpl3 | ribosomal protein L3 | Signaling |
| 1388534_at | Slc31a1 | solute carrier family 34 member 1 | Other |
| 1368016_at | Pecr | peroxisomal trans-2-enoyl-CoA reductase | Metabolism |
| 1388900_at | LOC102549726 | uncharacterized LOC102549726 | -- |
| 1388579_at | Slc25a42 | solute carrier family 25, member 42 | -- |
| 1374750_at | Fnip1 | Folliculin Interacting Protein 1 | Signaling |
| 1388752_at | Bclaf1 | BCL2-associated transcription factor 1 | -- |
| 1371929_at | Mlx | MLX, MAX dimerization protein | -- |
| 1373387_at | Slirp | SRA stem-loop interacting RNA binding protein | Transcription/Translation regulation |
| 1393902_at | Akr1c1 | aldo-keto reductase family 1, member C1 | Signaling |
| 1375374_at | LOC102555400 | -- | -- |
| 1388772_at | Lsm8 | LSM8 homolog, U6 small nuclear RNA associated | Metabolism |
| 1387896_at | Scp2 | Sterol carrier protein 2 | Metabolism |
| 1372340_at | Mat2b | methionine adenosyltransferase 2B | mRNA/Protein processing |
| 1388810_at | Abce1 | ATP binding cassette subfamily E member 1 | mRNA/Protein processing |
| 1388117_at | Snrpb | Small nuclear ribonucleoprotein polypeptides B and B1 | Transcription/Translation regulation |
| 1368849_at | Csnk1g3 | casein kinase 1, gamma 3 | Signaling |
| 1387323_at | Klkb1 | kallikrein B1 | -- |
| 1371729_at | Ypel5 | yippee-like 5 | -- |
| 1368021_at | Adh1 /// Adh6 | alcohol dehydrogenase 1 | Metabolism |
| 1372919_at | Gpat4 | glycerol-3-phosphate acyltransferase 4 | -- |
| 1388135_at | Rpa2 | replication protein A2 | -- |
| 1390602_a_at | Tprn | taperin | -- |
| 1387093_at | Slco1a2 | solute carrier organic anion transporter family, member 1A2 | Transport |
| 1367695_at | Qdpr | quinoid dihydropteridine reductase | Metabolism |
| 1390163_at | Wdr92 | WD repeat domain 92 | -- |
| 1372024_at | Mafg | MAF bZIP transcription factor G | -- |
| 1372734_at | Smagp | small cell adhesion glycoprotein | Cytoskeleton/ECM |
| 1368967_at | Eif2b3 | eukaryotic translation initiation factor 2B, subunit 3 gamma | -- |
| 1389596_at | Cox18 | COX18, cytochrome c oxidase assembly factor | Mitochondrial |
| 1376976_at | Sectm1b | secreted and transmembrane 1B | Inflammation/Immune response |
| 1372120_at | Uba5 | ubiquitin-like modifier activating enzyme 5 | Metabolism |
| 1387836_at | Ykt6 | YKT6 v-SNARE homolog | -- |
| 1368522_at | Timeless | timeless circadian clock | Transcription/Translation regulation |
| 1369959_at | Zfp36l1 | zinc finger protein 36, C3H type-like 1 | Signaling |
| 1370245_at | Ctsl | cathepsin L | Transcription/Translation regulation |
| 1370964_at | Ass1 | argininosuccinate synthase 1 | Metabolism |
| 1389521_at | Ivns1abp | Influenza virus ns1a binding protein | mRNA/Protein processing |
| 1373248_at | Vsig10 | V-set & immunoglobulin domain containing 10 | EST/Unknown |
| 1388566_at | Lasp1 | LIM and SH3 protein 1 | Cytoskeleton/ECM |
| 1383013_at | Klf13 | Kruppel-like factor 13 | Inflammation/Immune response |
| 1369902_at | Bmf | Bcl-2 modifying factor | Cell cycle/Apoptosis |
| 1374716_at | Rrp15 | ribosomal RNA processing 15 homolog | Metabolism |
| 1385072_at | Galm | galactose mutarotase | Metabolism |
| 1367818_at | Coq3 | coenzyme Q3 methyltransferase | Other |
| 1379604_at | Apol3 | apolipoprotein L, 3 | -- |
| 1371076_at | Cyp2b1 /// Cyp2b2 | cytochrome P450, family 2, subfamily b, polypeptide 1 | -- |
| 1372473_at | Tjp1 | tight junction protein 1 | -- |
| 1389297_at | Ero1a | endoplasmic reticulum oxidoreductase 1 alpha | -- |
| 1386982_at | Mgat2 | mannosyl -glycoprotein beta-1,2-N-acetylglucosaminyltransferase | Metabolism |
| 1371576_at | Mrps36 | mitochondrial ribosomal protein S36 | -- |
| 1371680_at | Gabarapl1 | GABA type A receptor associated protein like 1 | Transcription/Translation regulation |
| 1370320_at | Pbld1 | phenazine biosynthesis-like protein domain containing 2 | -- |
| 1388682_at | Cnih1 | cornichon family AMPA receptor auxiliary protein 1 | -- |
| 1389291_at | Chchd3 | coiled-coil-helix-coiled-coil-helix domain containing 3 | -- |
| 1389493_at | Abtb1 | ankyrin repeat and BTB domain containing 1 | Other |
| 1388644_at | Mgll | -- | -- |
| 1374890_at | Evi5 | ecotropic viral integration site 5 | Metabolism |
| 1375161_at | Mrpl55 | mitochondrial ribosomal protein L55 | Mitochondrial |
| 1386976_at | Cd82 | Cd82 molecule | -- |
| 1398799_at | Eif4e | Eukaryotic translation initiation factor 4e | Transcription/Translation regulation |
| 1374677_at | Adssl1 | adenylosuccinate synthase like 1 | Metabolism |
| 1376860_at | Slc22a23 | solute carrier family 22, member 23 | Transport |
| 1371463_at | Phf5a | PHD finger protein 5A | Transcription/Translation regulation |
| 1371409_at | RGD1562987 | -- | -- |
| 1372512_at | Stx18 | Syntaxin 18 | Vesicular transport |
| 1398930_at | Atp6v0b | ATPase H+ transporting V0 subunit B | -- |
| 1372685_at | Cdkn3 | cyclin-dependent kinase inhibitor 3 | Cell cycle/Apoptosis |
| 1389538_at | Nfkbia | NFKB inhibitor alpha | Inflammation/Immune response |
| 1372028_at | Nop16 | NOP16 nucleolar protein | -- |
| 1386978_at | Bnip3l | BCL2/adenovirus E1B interacting protein 3-like | -- |
| 1375726_at | Lmo7 | LIM domain 7 | -- |
| 1377042_at | Pcgf5 | Polycomb group ring finger 5 | Transcription/Translation regulation |
| 1388616_at | Prelid3b | PRELI domain containing 3B | Transport |
| 1372030_at | Zfyve21 | zinc finger FYVE-type containing 21 | Cytoskeleton/ECM |
| 1388401_at | Flnb /// LOC100911261 | filamin-B-like | -- |
| 1371638_at | Rnf7 | ring finger protein 7 | Cell cycle/Apoptosis |
| 1377058_at | Slc37a2 | solute carrier family 37 member 2 | Transport |
| 1368490_at | Cd14 | CD14 molecule | -- |
| 1367583_at | Tpt1 | tumor protein, translationally-controlled 1 | Cell cycle/Apoptosis |
| 1388364_at | Ndufs3 | NADH-ubiquinone oxidoreductase Fe-S protein 3 | Mitochondrial |
| 1371565_at | Kti12 | KTI12 chromatin associated homolog | -- |
| 1371656_at | Cct4 | chaperonin containing TCP1 subunit 4p | Cell cycle/Apoptosis |
| 1398979_at | Angel2 | Angel Homolog 2 | -- |
| 1374231_at | Klf16 | Kruppel-like factor 16 | Metabolism |
| 1372069_at | Kank1 | KN motif and ankyrin repeat domains 1 | -- |
| 1369746_a_at | Slco1b2 | solute carrier organic anion transporter family, member 1B2 | Transport |
| 1374941_at | N4bp2 | NEDD4 binding protein 2 | -- |
| 1389391_at | Slc35e3 | solute carrier family 35, member E3 | -- |
| 1371795_at | Cyb5b | cytochrome b5 type B | Metabolism |
| 1367767_at | Hmgcl | 3-hydroxymethyl-3-methylglutaryl-CoA lyase | Metabolism |
| 1390455_at | Abhd2 | abhydrolase domain containing 2 | mRNA/Protein processing |
| 1398835_at | Actb | actin, beta | Other |
| 1372908_at | Spsb3 | splA/ryanodine receptor domain and SOCS box containing 3 | Inflammation/Immune response |
| 1367927_at | Phb | prohibitin | Cell cycle/Apoptosis |
| 1372431_at | Mrpl12 | mitochondrial ribosomal protein L12 | Mitochondrial |
| 1369629_at | Adk | adenosine kinase | Metabolism |
| 1388651_at | Rchy1 | ring finger and CHY zinc finger domain containing 1 | Metabolism |
| 1367718_at | Chkb | Choline kinase-like | Lipid Metabolism |
| 1367657_at | Btg1 | BTG anti-proliferation factor 1 | Cell cycle/Apoptosis |
| 1371632_at | Coro1c | Coronin, actin-binding protein 1C | Cell cycle/Apoptosis |
| 1369934_at | Ppib | peptidylprolyl isomerase B | -- |
| 1369200_at | Nt5e | 5' nucleotidase, ecto | Other |
| 1368596_at | Sik1 | Salt-inducible kinase 1 | Signaling |
| 1372150_at | Usp10 | ubiquitin specific peptidase 10 | -- |
| 1389003_at | Rhobtb3 | Rho-related BTB domain containing 3 | Signaling |
| 1372953_at | Ncald | neurocalcin delta | Signaling |
| 1368706_at | Tm4sf4 | transmembrane 4 L six family member 4 | Cell cycle/Apoptosis |
| 1372078_at | Strap | Serine/Threonine Kinase Receptor Associated Protein | -- |
| 1369013_a_at | Mrpl17 | mitochondrial ribosomal protein L17 | -- |
| 1371254_at | Uqcrfs1 | ubiquinol-cytochrome c reductase, Rieske iron-sulfur polypeptide 1 | -- |
| 1388458_at | Rfc4 | replication factor C subunit 4 | Transcription/Translation regulation |
| 1388976_at | Bola3 | bolA family member 3 | -- |
| 1373711_at | Orc5 | origin recognition complex, subunit 5 | Metabolism |
| 1389010_at | Lta4h | leukotriene A4 hydrolase | -- |
| 1372770_at | Sri | sorcin | -- |
| 1371789_at | Clpp | caseinolytic mitochondrial matrix peptidase proteolytic subunit | -- |
| 1368173_at | Nop58 | NOP58 ribonucleoprotein | mRNA/Protein processing |
| 1375858_at | Nprl2 | NPR2-like, GATOR1 complex subunit | Signaling |
| 1372790_at | Mdh1 | malate dehydrogenase 1 | Metabolism |
| 1374441_at | Spryd4 | SPRY domain containing 4 | -- |
| 1376038_at | Tex2 | testis expressed 2 | -- |
| 1368009_at | Gne | glucosamine -2-epimerase/N-acetylmannosamine kinase | Metabolism |
| 1398810_at | Pdap1 | PDGFA associated protein 1 | -- |
| 1389567_at | Scap | SREBF chaperone | -- |
| 1399034_at | Pcnx | pecanex homolog 1 | Transcription/Translation regulation |
| 1370298_at | Coa3 | cytochrome C oxidase assembly factor 3 | Transcription/Translation regulation |
| 1371848_at | Snrpd3 | small nuclear ribonucleoprotein D3 polypeptide | Metabolism |
| 1372704_at | Ostc | Oligosaccharyltransferase complex subunit | mRNA/Protein processing |
| 1388758_at | Ogt | O-linked N-acetylglucosamine (GlcNAc) transferase | Metabolism |
| 1367527_at | LOC686774 | similar to Importin-8 | -- |
| 1387036_at | Hes1 | hes family bHLH transcription factor 1 | Transcription/Translation regulation |
| 1367886_at | Pcsk7 | proprotein convertase subtilisin/kexin type 7 | Inflammation/Immune response |
| 1389229_at | Pxylp1 | 2-phosphoxylose phosphatase 1 | Metabolism |
| 1370808_at | Cyb5r3 | cytochrome b5 reductase 3 | Signaling |
| 1374591_at | Ptprd | Protein tyrosine phosphatase, receptor type, d | Signaling |
| 1370975_at | Kdm3a | lysine demethylase 3A | Transcription/Translation regulation |
| 1389048_at | Bmp1 | bone morphogenetic protein 1 | Signaling |
| 1389072_at | Mtmr4 | myotubularin related protein 4 | Transport |
| 1387914_at | Cyp27a1 | cytochrome P450, family 27, subfamily a, polypeptide 1 | Metabolism |
| 1372607_at | Nubp2 | nucleotide binding protein 2 | -- |
| 1388163_at | Slc25a5 | ADP/ATP translocase 3-like | -- |
| 1371812_at | Rtcb | RNA 2',3'-cyclic phosphate and 5'-OH ligase | -- |
| 1371745_at | Cwc15 | CWC15 spliceosome-associated protein | Metabolism |
| 1372763_at | Nus1 | NUS1 dehydrodolichyl diphosphate synthase subunit | -- |
| 1373913_at | Pnpt1 | Polyribonucleotide nucleotidyltransferase 1 | Transcription/Translation regulation |
| 1375423_at | LOC689959 | hypothetical protein LOC689959 | -- |
| 1370531_a_at | Pld1 | phospholipase D1 | Transport |
| 1371971_at | Txnl4a | thioredoxin like 4A | mRNA/Protein processing |
| 1398891_at | Mrpl15 | Mitochondrial ribosomal protein L15 | Mitochondrial |
| 1371851_at | Psmd6 | proteasome 26S subunit, non-ATPase 6 | mRNA/Protein processing |
| 1372242_at | Ddx3x | DEAD-box helicase 3, X-linked | Transcription/Translation regulation |
| 1370847_at | Spon2 | spondin 2 | Cytoskeleton/ECM |
| 1388681_at | Sar1b | secretion associated, Ras related GTPase 1B | Transport |
| 1389476_at | RGD1307100 | similar to RIKEN cDNA D630029K19 | -- |
| 1377156_at | Tcf7l2 | transcription factor 7 like 2 | -- |
| 1371926_at | Il6st | interleukin 6 signal transducer | -- |
| 1371724_at | Rexo2 | RNA exonuclease 2 | Metabolism |
| 1388669_at | Sf3a3 | splicing factor 3a, subunit 3 | Metabolism |
| 1389919_at | Parvb | parvin, beta | Cytoskeleton/ECM |
| 1373557_at | Mcm4 | minichromosome maintenance complex component 4 | Cell cycle/Apoptosis |
| 1376997_at | Fzd4 | frizzled class receptor 4 | Signaling |
| 1389984_at | Jarid2 | jumonji and AT-rich interaction domain containing 2 | Metabolism |
| 1367870_at | Glrx3 | glutaredoxin 3 | Signaling |
| 1389525_at | Rnf149 | ring finger protein 149 | -- |
| 1367644_at | Adcy6 | adenylate cyclase 6 | Metabolism |
| 1389197_at | Poglut1 | protein O-glucosyltransferase 1 | Metabolism |
| 1370893_at | Acaca | acetyl-CoA carboxylase alpha | Metabolism |
| 1372497_at | Nbr1 | NBR1, autophagy cargo receptor | Signaling |
| 1398267_at | Slc22a7 | solute carrier family 22 member 7 | Transport |
| 1372450_at | Cmc2 | C-x-C motif containing 2 | -- |
| 1390448_at | Abhd13 | Abhydrolase domain containing 13 | Lipid Metabolism |
| 1374936_at | Amdhd2 | amidohydrolase domain containing 2 | Metabolism |
| 1370305_at | Yif1a | Yip1 interacting factor homolog A, membrane trafficking protein | Transport |
| 1388953_at | Gnl3 | G protein nucleolar 3 | -- |
| 1389156_at | Kxd1 | Hypothetical protein LOC498606 | Other |
| 1389344_at | Usp39 | Ubiquitin-specific protease 39 | Transcription/Translation regulation |
| 1372241_at | Oaz1 | ornithine decarboxylase antizyme 1 | Cell cycle/Apoptosis |
| 1388333_at | Rbx1 | -- | -- |
| 1374421_at | Baz1b | bromodomain adjacent to zinc finger domain, 1B | -- |
| 1389329_at | Lgals8 | galectin 8 | -- |
| 1370057_at | Csrp1 | cysteine and glycine-rich protein 1 | Cell cycle/Apoptosis |
| 1371543_at | Mtmr2 | myotubularin related protein 2 | Transport |
| 1368230_a_at | Ndufaf3 | Nuclear protein E3–3 | Other |
| 1370365_at | Gss | glutathione synthetase | Metabolism |
| 1375916_at | Pcmtd2 | protein-L-isoaspartate O-methyltransferase domain containing 2 | Metabolism |
| 1374156_at | Mpp5 | Membrane protein, palmitoylated 5 | Other |
| 1389459_at | LOC500028 /// Yae1d1 | Yae1 domain containing 1 | Metabolism |
| 1373862_at | Tmed7 | transmembrane p24 trafficking protein 7 | mRNA/Protein processing |
| 1374055_at | Erf | Ets2 repressor factor | Transcription/Translation regulation |
| 1388449_at | Eef1b2 | eukaryotic translation elongation factor 1 beta 2 | Transcription/Translation regulation |
| 1383654_a_at | Fn3k | fructosamine 3 kinase | Cytoskeleton/ECM |
| 1372115_at | Ubr2 | ubiquitin protein ligase E3 component n-recognin 2 | -- |
| 1367606_at | Rps3a | Ribosomal protein S3a | Transcription/Translation regulation |
| 1371626_at | Srp68 | signal recognition particle 68 | -- |
| 1376062_at | Sdc1 | syndecan 1 | Transport |
| 1371820_at | Mesdc2 | -- | Other |
| 1398867_at | Prpf19 | pre-mRNA processing factor 19 | Metabolism |
| 1371301_at | LOC100360449 /// LOC100364457 /// Rpl9 | ribosomal protein L9-like | Metabolism |
| 1372347_at | Skil | SKI-like proto-oncogene | Signaling |
| 1372255_at | Rars | arginyl-tRNA synthetase | mRNA/Protein processing |
| 1390591_at | Slc17a3 | solute carrier family 17 member 3 | Transport |
| 1372088_at | Ppfibp1 | PPFIA binding protein 1 | -- |
| 1367561_at | Rpl27 | ribosomal protein L27 | mRNA/Protein processing |
| 1388406_at | Naa60 | N-acetyltransferase 60, NatF catalytic subunit | mRNA/Protein processing |
| 1371316_at | Fau /// LOC100360647 /// LOC687780 | ubiquitin-like protein fubi and ribosomal protein S30-like | -- |
| 1387214_at | Ces1e | Carboxylesterase 1 | Metabolism |
| 1376722_at | Nup205 | nucleoporin 205 | -- |
| 1388640_at | Sumo3 | Smt3 suppressor of mif two 3 homolog 3 | mRNA/Protein processing |
| 1370313_at | Acot7 | acyl-CoA thioesterase 7 | Metabolism |
| 1390157_at | Ube2h | ubiquitin-conjugating enzyme E2H(Ube2h) Rattus norvegicus | -- |
| 1367894_at | Insig1 | insulin induced gene 1 | Metabolism |
| 1389287_at | Tead1 | Tea domain family 1 | Transcription/Translation regulation |
| 1373071_at | Lin37 | lin-37 DREAM MuvB core complex component | -- |
| 1372558_at | Naa15 | N-acetyltransferase 15, NatA auxiliary subunit | Metabolism |
| 1388723_at | Bre | brain and reproductive organ-expressed | -- |
| 1389040_at | Pcnp | PEST proteolytic signal containing nuclear protein | -- |
| 1390171_at | Fam76a | family with sequence similarity 76, member A | Transcription/Translation regulation |
| 1368136_at | Tmpo | Thymopoietin | Transcription/Translation regulation |
| 1372612_at | Dynll2 | dynein light chain LC8-type 2 | Transcription/Translation regulation |
| 1372406_at | Mcm3 | minichromosome maintenance complex component 3 | Cell cycle/Apoptosis |
| 1389380_at | Fpgs | folylpolyglutamate synthase | -- |
| 1390532_at | Slc13a4 | solute carrier family 13 member 4 | Transport |
| 1398812_at | Psmb1 | proteasome subunit beta 1 | Metabolism |
| 1373692_at | Ankhd1 /// Eif4ebp3 | eukaryotic translation initiation factor 4E binding protein 3 | -- |
| 1377209_at | Klhl25 /// LOC103695026 | kelch-like family member 25 | -- |
| 1391510_at | Ebag9 | estrogen receptor binding site associated, antigen, 9 | -- |
| 1390607_at | Zdhhc23 | zinc finger, DHHC-type containing 23 | mRNA/Protein processing |
| 1389668_at | Spc25 | SPC25, NDC80 kinetochore complex component | mRNA/Protein processing |
| 1367495_at | Pfdn4 | prefoldin subunit 4 | -- |
| 1367580_at | LOC100363537 /// RGD1566137 /// Rpl10a | ribosomal protein L10A | mRNA/Protein processing |
| 1387867_at | Aldh9a1 | aldehyde dehydrogenase 9 family, member A1 | Metabolism |
| 1371311_at | Sdhc | succinate dehydrogenase complex subunit C | Transport |
| 1368132_at | Tob1 | transducer of ErbB-2.1 | -- |
| 1373835_at | Fbxo8 | F-box protein 8 | -- |
| 1389518_at | Tomm5 | translocase of outer mitochondrial membrane 5 | -- |
| 1390109_at | LOC100910069 /// LOC102554001 /// LOC680724 | uncharacterized LOC100910069 | -- |
| 1375120_at | Id4 | inhibitor of DNA binding 4, HLH protein | -- |
| 1387018_at | Sorbs2 | sorbin and SH3 domain containing 2 | Cell cycle/Apoptosis |
| 1373936_at | Rnf44 | ring finger protein 44 | -- |
| 1388759_at | Clic4 | chloride intracellular channel 4 | Transport |
| 1369919_at | Tef | TEF, PAR bZIP transcription factor | Transcription/Translation regulation |
| 1388754_at | LOC100911177 | uncharacterized LOC100911177 | -- |
| 1387434_at | Slc22a4 | solute carrier family 22 member 4 | -- |
| 1372409_at | Mad2l1bp | MAD2L1 binding protein | -- |
| 1369220_at | Dnm1l | dynamin 1-like | Transcription/Translation regulation |
| 1376837_at | H6pd | Hexose-6-Phosphate Dehydrogenase/Glucose 1-Dehydrogenase | Metabolism |
| 1372783_at | Larp4b | La ribonucleoprotein domain family, member 4B | Cell cycle/Apoptosis |
| 1376196_a_at | Med4 | mediator complex subunit 4 | Metabolism |
| 1382048_at | Atp6v1a | Myosin ID | Other |
| 1388674_at | Cdkn1a | cyclin-dependent kinase inhibitor 1A | Cell cycle/Apoptosis |
| 1375060_at | Apitd1 | phosphogluconate dehydrogenase | Metabolism |
| 1398817_at | Arf1 | ADP-ribosylation factor 1 | Signaling |
| 1398928_at | Cuta | cutA divalent cation tolerance homolog | -- |
| 1398886_at | RGD1563348 | -- | -- |
| 1374858_at | Dhx29 | DExH-box helicase 29 | Transcription/Translation regulation |
| 1367725_at | Pim3 | Pim-3 proto-oncogene, serine/threonine kinase | Metabolism |
| 1374632_at | Jmjd6 | jumonji domain containing 6 | -- |
| 1387942_at | Slc35e4 | solute carrier family 35, member E4 | -- |
| 1375185_at | Ipo7 | importin 7 | Signaling |
| 1368129_at | Sfmbt1 | Scm-like with four mbt domains 1 | Cell cycle/Apoptosis |
| 1367586_at | Ldha | lactate dehydrogenase A | Metabolism |
| 1371455_at | Pmm1 | phosphomannomutase 1 | mRNA/Protein processing |
| 1376252_at | Srsf3 | Splicing factor, arginine/serine-rich 3 (SRp20)(Sfrs3) | Transcription/Translation regulation |
| 1389296_at | Emc2 | ER membrane protein complex subunit 2 | Metabolism |
| 1367456_at | Ube2d2 /// Ube2d3 | ubiquitin-conjugating enzyme E2D 2 | Metabolism |
| 1383347_at | Rev1 | REV1, DNA directed polymerase | Cell cycle/Apoptosis |
| 1398756_at | LOC300303 /// Npm1 | nucleophosmin 1 | Metabolism |
| 1372829_at | Rbm8a | RNA binding motif protein 8A | Transcription/Translation regulation |
| 1371877_at | Dexi /// LOC102553347 | uncharacterized LOC102553347 | -- |
| 1371968_at | Tmbim4 | transmembrane BAX inhibitor motif containing 4 | -- |
| 1373885_at | Cbx5 | chromobox 5 | -- |
| 1368247_at | Hspa1a /// Hspa1b | heat shock 70kD protein 1A | Metabolism |
| 1371392_at | Gpi | glucose-6-phosphate isomerase | Metabolism |
| 1379252_at | LOC102556753 | -- | -- |
| 1377937_at | Mrps14 | mitochondrial ribosomal protein S14 | Metabolism |
| 1368574_at | Adra1b | adrenoceptor alpha 1B | Metabolism |
| 1367905_at | Enpp3 | Ectonucleotide pyrophosphatase/phosphodiesterase 3 | Metabolism |
| 1370322_at | Stk16 | serine/threonine kinase 16 | -- |
| 1370188_at | Tra2b | transformer 2 beta homolog | Metabolism |
| 1374359_at | Ccne2 | cyclin E2 | Cell cycle/Apoptosis |
| 1370020_at | Slc25a10 | solute carrier family 25 member 10 | Transport |
| 1368062_at | Ap3m1 | adaptor-related protein complex 3, mu 1 subunit | Metabolism |
| 1373867_at | Kdm5a | lysine demethylase 5A | Metabolism |
| 1389717_at | Fam175b | EST | Other |
| 1389586_at | Ednrb | Endothelin Receptor Type B | -- |
| 1398324_at | Rpl18a | ribosomal protein L18A | mRNA/Protein processing |
| 1371415_at | Uqcrh | ubiquinol-cytochrome c reductase hinge protein | Metabolism |
| 1388587_at | Ier3 | immediate early response 3 | -- |
| 1367775_at | Amacr | alpha-methylacyl-CoA racemase | -- |
| 1368177_at | Acsl3 | acyl-CoA synthetase long-chain family member 3 | Metabolism |
| 1374281_at | Ino80 | INO80 complex subunit | Transcription/Translation regulation |
| 1388408_at | Prr13 | Proline rich 13 | Cell cycle/Apoptosis |
| 1388321_at | Imp3 | IMP3, U3 small nucleolar ribonucleoprotein | Transcription/Translation regulation |
| 1389655_at | Ptrh2 | peptidyl-tRNA hydrolase 2 | -- |
| 1372791_at | Wnk1 | WNK lysine deficient protein kinase 1 | Transcription/Translation regulation |
| 1386871_at | Gpx4 | glutathione peroxidase 4 | Inflammation/Immune response |
| 1388909_at | Oxnad1 | oxidoreductase NAD-binding domain containing 1 | -- |
| 1372722_at | Dnajb4 | DnaJ heat shock protein family member B4 | Metabolism |
| 1373640_at | Pycr2 | pyrroline-5-carboxylate reductase family, member 2 | -- |
| 1367969_at | Prdx6 | peroxiredoxin 6 | Metabolism |
| 1387038_at | Ccs | Copper chaperone for superoxide dismutase | Other |
| 1371734_at | Maea | macrophage erythroblast attacher | Metabolism |
| 1374036_at | Mcm2 | Minichromosome maintenance deficient 2 mitotin | Cell cycle/Apoptosis |
| 1387395_at | Adora2b | Adenosine a2b receptor | Signaling |
| 1371900_at | Celf1 | CUGBP, Elav-like family member 1 | Transcription/Translation regulation |
| 1372902_at | Alg12 | ALG12, alpha-1,6-mannosyltransferase | Metabolism |
| 1373784_at | Cct8 | chaperonin containing TCP1 subunit 8 | Metabolism |
| 1373449_at | LOC690283 /// Taf5l | TAF5-like RNA polymerase II, p300/CBP-associated factor -associated factor | -- |
| 1372323_at | Sardh | sarcosine dehydrogenase | Metabolism |
| 1369546_at | Bbox1 | gamma-butyrobetaine hydroxylase 1 | Metabolism |
| 1377155_at | LOC102549803 /// LOC102556148 | probable N-acetyltransferase CML2-like | Metabolism |
| 1375093_at | Rbks | ribokinase | Metabolism |
| 1370180_at | Nudt4 | nudix hydrolase 4 | Signaling |
| 1389685_at | LOC103690102 /// Zfp655 | zinc finger protein 655 | -- |
| 1376376_at | Zbtb44 | Zinc Finger And BTB Domain Containing 44 | -- |
| 1374947_at | Bcar3 | breast cancer anti-estrogen resistance 3 | -- |
| 1373603_at | Tsr3 | TSR3, 20S rRNA accumulation | -- |
| 1398929_at | Eif3l | eukaryotic translation initiation factor 3, subunit L | Metabolism |
| 1367506_at | mrpl11 | Mitochondrial ribosomal | Mitochondrial |
| 1376796_at | Rab14 | RAB14, member RAS oncogene family | Signaling |
| 1389218_at | Uggt1 | UDP-Glucose Glycoprotein Glucosyltransferase 1 | -- |
| 1398885_at | RGD1561618 /// Rpl23 | Ribosomal protein L23 | Transcription/Translation regulation |
| 1372223_at | Cpeb4 | Cytoplasmic Polyadenylation Element Binding Protein 4 | -- |
| 1398295_at | Slc29a1 | solute carrier family 29 member 1 | Transport |
| 1398919_at | RGD1304704 | similar to Hypothetical protein CGI-99 | -- |
| 1389555_at | Tcf19 | transcription factor 19 | -- |
| 1367627_at | Gatm | glycine amidinotransferase | -- |
| 1367814_at | Atp1b1 | Atpase, na+/k+ transporting, β 1 | Transport |
| 1372650_at | Dnmbp | dynamin binding protein | -- |
| 1368184_at | Psmd9 | Proteasome (prosome, macropain) 26S subunit, non-ATPase, 9 | mRNA/Protein processing |
| 1388104_at | Lgr4 | leucine-rich repeat-containing G protein-coupled receptor 4 | Signaling |
| 1371936_at | Eif4a1 | eukaryotic translation initiation factor 4A1 | -- |
| 1368127_at | Neu2 | neuraminidase 2 | Signaling |
| 1367713_at | Eif2s1 | eukaryotic translation initiation factor 2 subunit 1 alpha | Metabolism |
| 1369291_at | Agtr1a | angiotensin II receptor, type 1a | Metabolism |
| 1388297_at | Eef1g | eukaryotic translation elongation factor 1 gamma | Metabolism |
| AFFX_Rat_GAPDH_M_at | Gapdh | glyceraldehyde-3-phosphate dehydrogenase | Metabolism |
| 1383539_at | Prrg1 | proline rich and Gla domain 1 | Metabolism |
| 1387950_at | Nip7 | NIP7, nucleolar pre-rRNA processing protein | -- |
| 1367802_at | Sgk1 | serum/glucocorticoid regulated kinase 1 | -- |
| 1388709_at | Wdr43 | WD repeat domain 43 | -- |
| 1368870_at | Id2 | inhibitor of DNA binding 2, HLH protein | Cell cycle/Apoptosis |
| 1389033_at | Uqcc2 | ubiquinol-cytochrome c reductase complex assembly factor 2 | -- |
| 1367826_at | Nfe2l2 | nuclear factor, erythroid 2-like 2 | -- |
| 1390224_at | Ikbkg | NOTFOUND | -- |
| 1386877_at | Ap2s1 | adaptor-related protein complex 2, sigma 1 subunit | Transport |
| 1373002_at | Mrps9 | mitochondrial ribosomal protein S9 | Metabolism |
| 1368720_at | Tdo2 | tryptophan 2,3-dioxygenase | Metabolism |
| 1370881_at | Tst | thiosulfate sulfurtransferase | Transport |
| 1372654_at | Eps8l2 | EPS8-like 2 | -- |
| 1371332_at | LOC684681 | -- | EST/Unknown |
| 1388059_a_at | Slc11a2 | solute carrier family 11 member 2 | Inflammation/Immune response |
| 1386862_at | Anxa5 | annexin A5 | Metabolism |
| 1372496_at | Fdx1l | ferredoxin 1-like | -- |
| 1375220_at | Ndufa11 | NADH:ubiquinone oxidoreductase subunit A11 | -- |
| 1387209_at | Sec16b | SEC16 homolog B, endoplasmic reticulum export factor | mRNA/Protein processing |
| 1367980_at | Rabep1 | rabaptin, RAB GTPase binding effector protein 1 | -- |
| 1389549_at | Prosc | proline synthetase co-transcribed homolog | -- |
| 1388568_at | Eif3d | eukaryotic translation initiation factor 3, subunit D | Metabolism |
| 1372534_at | Polr2k | polymerase II polypeptide K | Metabolism |
| 1376824_at | Ddi2 | DNA damage inducible 1 homolog 2 | -- |
| 1374652_at | Lrig1 | leucine-rich repeats and immunoglobulin-like domains 1 | -- |
| 1376585_at | Mrpl50 | mitochondrial ribosomal protein L50 | -- |
| 1376187_at | Slc35d1 | Solute Carrier Family 35 Member D1 | -- |
| 1372731_at | Rhot1 | ras homolog family member T1 | -- |
| 1373632_at | Ak6 /// Taf9 | adenylate kinase 6 | -- |
| 1372274_at | Kmt2e | lysine methyltransferase 2E | Cell cycle/Apoptosis |
| 1388380_at | Samm50 | SAMM50 sorting and assembly machinery component | -- |
| 1367541_at | Mettl5 | Methyltransferase-like 5 | Transcription/Translation regulation |
| 1390421_at | Hoga1 | 4-hydroxy-2-oxoglutarate aldolase 1 | Metabolism |
| 1387887_at | Rpl14 | ribosomal protein L14 | mRNA/Protein processing |
| 1371657_at | Uba2 | ubiquitin-like modifier activating enzyme 2 | -- |
| 1370168_at | Ywhaq | tyrosine 3-monooxygenase/tryptophan 5-monooxygenase activation protein, theta | Signaling |
| 1375977_at | Cetn2 | centrin 2 | -- |
| 1367591_at | Prdx3 | peroxiredoxin 3 | Signaling |
| 1372741_at | LOC103689999 /// Sccpdh | saccharopine dehydrogenase | Metabolism |
| 1368511_at | Bhlhe41 | Basic helix-loop-helix domain containing, class B, 3 | Transcription/Translation regulation |
| 1367609_at | LOC103694877 /// Mif | Macrophage migration inhibitory factor | Inflammation/Immune response |
| 1398417_at | Fem1a | fem-1 homolog A | -- |
| 1390149_at | Tacc2 | transforming, acidic coiled-coil containing protein 2 | -- |
| 1373319_at | Ddx1 | DEAD (Asp-Glu-Ala-Asp) box polypeptide 1 | Transcription/Translation regulation |
| 1371428_at | Sec61g | Sec61 translocon gamma subunit | -- |
| 1398766_at | Rpn1 | ribophorin I | -- |
| 1371342_at | Cyc1 | cytochrome c-1 | Transport |
| 1388344_at | Sarnp | SAP domain containing ribonucleoprotein | mRNA/Protein processing |
| 1398883_at | Hnrnpa2b1 | heterogeneous nuclear ribonucleoprotein A2/B1 | Transcription/Translation regulation |
| 1398871_at | Rpl17 | Ribosomal protein L17 | Transcription/Translation regulation |
| 1388978_at | Tmem30a | Transmembrane Protein 30A | -- |
| 1374505_at | Lyrm5 | electron transfer flavoprotein regulatory factor 1 | -- |
| 1371592_at | Csk | c-src tyrosine kinase | Signaling |
| 1375452_at | LOC100912115 | cutaneous T-cell lymphoma-associated antigen 5 homolog | -- |
| 1376936_at | Slc8b1 | solute carrier family 8 member B1 | Mitochondrial |
| 1388778_at | Ppm1k | protein phosphatase, Mg2+/Mn2+ dependent, 1K | Metabolism |
| 1389710_at | Sos1 | SOS Ras/Rac guanine nucleotide exchange factor 1 | Signaling |
| 1368617_at | Serpina5 | serpin family A member 5 | -- |
| 1373044_at | Eif3m | eukaryotic translation initiation factor 3, subunit M | Metabolism |
| 1367730_at | Uso1 | USO1 vesicle transport factor | Bile Acid cholesterol |
| 1374513_at | septin-7-like | septin-7-like | Cytoskeleton/ECM |
| 1371482_at | Ndufs2 | NADH dehydrogenase Fe-S protein 2 | Metabolism |
| 1373778_at | Acacb | Acetyl-CoA Carboxylase Beta | -- |
| 1375536_at | Numb | NUMB, endocytic adaptor protein | Metabolism |
| 1370803_at | Zwint | ZW10 interacting kinetochore protein | -- |
| 1387085_at | Prps1 | phosphoribosyl pyrophosphate synthetase 1 | Metabolism |
| 1367596_at | Rps26 | ribosomal protein S26 | mRNA/Protein processing |
| 1370878_at | Urod | uroporphyrinogen decarboxylase | Cytoskeleton/ECM |
| 1386473_at | Dcaf6 | DDB1 and CUL4 associated factor 6 | Metabolism |
| 1369473_at | Pgm1 | phosphoglucomutase 1 | Cell cycle/Apoptosis |
| 1373935_at | Pold2 | DNA polymerase delta 2, accessory subunit | Metabolism |
| 1368839_at | Wfs1 | Wolframin ER Transmembrane Glycoprotein | -- |
| 1367578_at | Prdx2 | peroxiredoxin 2 | -- |
| 1388239_at | Per3 | period circadian clock 3 | Transcription/Translation regulation |
| 1370906_at | Bckdhb | branched chain keto acid dehydrogenase E1 subunit beta | Metabolism |
| 1371544_at | Erh | enhancer of rudimentary homolog (Drosophila) | -- |
| 1389318_at | Daam1 | dishevelled associated activator of morphogenesis 1 | Transport |
| 1370351_at | Tdrd7 | tudor domain containing 7 | Metabolism |
| 1398795_at | Dars | aspartyl-tRNA synthetase | Metabolism |
| 1375506_at | Adck5 | aarF domain containing kinase 5 | -- |
| 1388381_at | Eif3g | eukaryotic translation initiation factor 3, subunit G | Metabolism |
| 1374161_at | Ipo11 /// LOC314407 | importin 11 | Transport |
| 1399109_at | Nhlrc2 | NHL repeat containing 2 | -- |
| 1368507_at | LOC100361067 /// Psma3 /// Psma3l | proteasome subunit alpha type-3-like | -- |
| 1390020_at | LOC103693780 /// Ogdh | oxoglutarate dehydrogenase | -- |
| 1392731_at | Tnfrsf1b | TNF receptor superfamily member 1B | -- |
| 1390097_at | Tspyl4 | TSPY-like 4 | Cell cycle/Apoptosis |
| 1376865_at | Onecut2 | one cut homeobox 2 | -- |
| 1371646_at | Pgd | phosphogluconate dehydrogenase | Metabolism |
| 1379361_at | Pex11a | Peroxisomal biogenesis factor 11 α | Other |
| 1373719_at | Map4k3 | mitogen-activated protein kinase kinase kinase kinase 3 | Cell cycle/Apoptosis |
| 1398827_at | Cd81 | Cd81 molecule | Metabolism |
| 1367662_at | Hsd17b10 | hydroxysteroid dehydrogenase 10 | Metabolism |
| 1388404_at | Rpo1-3 | RNA polymerase I subunit D | -- |
| 1388153_at | Acsl1 | acyl-CoA synthetase long-chain family member 1 | Inflammation/Immune response |
| 1389566_at | Ccnb2 /// LOC100364016 | cyclin B2 | -- |
| 1373388_at | Sppl3 | signal peptide peptidase like 3 | -- |
| 1370243_a_at | LOC100359583 /// Ptma | hypothetical protein LOC100359583 | -- |
| 1373106_at | LOC100911319 /// Zfp36l2 | zinc finger protein 36, C3H type-like 2 | Metabolism |
| 1368662_at | Rnf39 | ring finger protein 39 | -- |
| 1367736_at | Rraga | Ras-related GTP binding A | Signaling |
| 1370909_at | Nup62 | nucleoporin 62 | mRNA/Protein processing |
| 1388883_at | Pold4 | Polymerase , δ 4 | Cell cycle/Apoptosis |
| 1374333_at | Card19 | caspase recruitment domain family, member 19 | -- |
| 1377675_at | LOC100302372 | hypothetical protein LOC100302372 | -- |
| 1375451_at | Pcnxl3 | pecanex homolog 3 | Transcription/Translation regulation |
| 1398832_at | Ncl | Nucleolin | Transcription/Translation regulation |
| 1371856_at | Pnrc2 | proline-rich nuclear receptor coactivator 2 | -- |
| 1399056_at | Pdss2 | prenyl diphosphate synthase, subunit 2 | -- |
| 1373984_at | Slc39a14 | solute carrier family 39 member 14 | -- |
| 1386886_at | Cd164 | CD164 molecule | -- |
| 1374346_at | Aifm2 | Apoptosis-inducing factor, mitochondrion-associated, 2 | Cell cycle/Apoptosis |
| 1388747_at | Lcmt1 | leucine carboxyl methyltransferase 1 | -- |
| 1389869_at | Sssca1 | Sjogren syndrome/scleroderma autoantigen 1 | -- |
| 1368109_at | St3gal5 | ST3 beta-galactoside alpha-2,3-sialyltransferase 5 | -- |
| 1373433_at | Hmgn5 /// Hmgn5b | high mobility group nucleosome binding domain 5 | Cell cycle/Apoptosis |
| 1399085_at | Zmpste24 | zinc metallopeptidase STE24 | Metabolism |
| 1377287_at | LOC100911305 /// Mars2 | methionine--tRNA ligase, mitochondrial-like | Metabolism |
| 1368106_at | Plk2 | polo-like kinase 2 | Signaling |
| 1388789_at | Tmem263 | transmembrane protein 263 | -- |
| 1388576_at | Eif3b | eukaryotic translation initiation factor 3, subunit B | Metabolism |
| 1375887_at | LOC100910807 /// Zbtb33 | -- | -- |
| 1399084_at | Dhx16 | DEAH-box helicase 16 | Metabolism |
| 1372267_at | Psmd5 | proteasome 26S subunit, non-ATPase 5 | -- |
| 1369863_at | Adh4 /// Adh5 | alcohol dehydrogenase 4 , pi polypeptide | -- |
| 1368871_at | LOC100912399 /// Map3k1 | mitogen-activated protein kinase kinase kinase 1-like | -- |
| 1370353_at | Timm22 | translocase of inner mitochondrial membrane 22 | mRNA/Protein processing |
| 1398943_at | Lage3 | L antigen family, member 3 | -- |
| 1388359_at | Eif4g2 /// LOC501116 | eukaryotic translation initiation factor 4, gamma 2 | -- |
| 1367537_at | Eif4enif1 | Eukaryotic translation initiation | Transcription/Translation regulation |
| 1388867_at | Tfdp1 | transcription factor Dp-1 | Metabolism |
| 1390387_at | Sh3d19 | SH3 domain containing 19 | -- |
| 1373138_at | Nudt5 | nudix hydrolase 5 | Metabolism |
| 1371305_at | Rpl8 | ribosomal protein L8-like | Metabolism |
| 1376623_at | Tmem204 | Transmembrane protein 204 | Other |
| 1375520_at | Copz1 | coatomer protein complex, subunit zeta 1 | Transport |
| 1398798_at | Metap2 /// RGD1560341 | similar to Methionine aminopeptidase 2 | -- |
| 1389940_at | Tmed2 | transmembrane p24 trafficking protein 2 | mRNA/Protein processing |
| 1375645_at | Szrd1 | -- | EST/Unknown |
| 1374855_at | Per1 | period circadian clock 1 | Transcription/Translation regulation |
| 1374962_at | Rtn4ip1 | reticulon 4 interacting protein 1 | -- |
| 1374775_at | Mki67 | marker of proliferation Ki-67 | Cell cycle/Apoptosis |
| 1374953_at | Tstd3 | thiosulfate sulfurtransferase (rhodanese)-like domain containing 3 | -- |
| 1374876_at | Leprotl1 | leptin receptor overlapping transcript-like 1 | Metabolism |
| 1371449_at | Pin1 | peptidylprolyl cis/trans isomerase, NIMA-interacting 1 | Cell cycle/Apoptosis |
| 1373397_at | Mapre1 | microtubule-associated protein, RP/EB family, member 1 | Signaling |
| 1371623_at | Sdhaf4 | succinate dehydrogenase complex assembly factor 4 | -- |
| 1388468_at | Cdc42se1 | CDC42 small effector 1 | -- |
| 1389202_at | Rpe | ribulose-5-phosphate-3-epimerase | Metabolism |
| 1388857_at | Sec23b | Sec23 homolog B, coat complex II component | Signaling |
| 1389006_at | Mpeg1 | macrophage expressed 1 | -- |
| 1367776_at | Cdk1 | cyclin-dependent kinase 1 | Cell cycle/Apoptosis |
| 1398913_at | Numa1 | nuclear mitotic apparatus protein 1 | Cell cycle/Apoptosis |
| 1377166_at | Als2 | ALS2, Alsin Rho Guanine Nucleotide Exchange Factor | -- |
| 1398315_at | LOC100362479 /// Rpl15 | Ribosomal protein L15 | Transcription/Translation regulation |
| 1389519_at | Psmd8 | Proteasome 26s subunit, non-atpase, 8 | mRNA/Protein processing |
| 1367698_a_at | Sept9 | septin 9 | mRNA/Protein processing |
| 1388744_at | Mcm7 | minichromosome maintenance complex component 7 | Cell cycle/Apoptosis |
| 1379240_at | LOC100911854 /// Mbl2 | mannose-binding protein C-like | -- |
| 1372334_at | Opa1 | OPA1, Mitochondrial Dynamin Like GTPase | -- |
| 1398348_at | Ddah1 | Dimethylarginine Dimethylaminohydrolase 1 | -- |
| 1392607_at | Epb4.1 | erythrocyte membrane protein band 4.1 | -- |
| 1367536_at | Apip | APAF1 interacting protein | Metabolism |
| 1373447_at | Hn1l | hematological and neurological expressed 1-like | -- |
| 1383169_at | LOC103691479 | Leukemia inhibitory factor receptor ? | Signaling |
| 1371480_at | Cks1b | CDC28 protein kinase regulatory subunit 1B | Cell cycle/Apoptosis |
| 1370940_at | Tjp2 | Tight junction protein 2 (zona occludens 2) | Bile acid cholesterol |
| 1398901_at | Nup98 | nucleoporin 98 | mRNA/Protein processing |
| 1373064_at | Dnajc15 | DnaJ heat shock protein family member C15 | Metabolism |
| 1388639_at | Bcas3 | BCAS3, microtubule associated cell migration factor | -- |
| 1369950_at | Cdk4 | cyclin-dependent kinase 4 | Cell cycle/Apoptosis |
| 1374943_at | Fam210b | family with sequence similarity 210, member B | -- |
| 1371355_at | Ndufa8 | NADH:ubiquinone oxidoreductase subunit A8 | -- |
| 1368947_at | Gadd45a | growth arrest and DNA-damage-inducible, alpha | -- |
| 1368378_at | Aldh1l1 | aldehyde dehydrogenase 1 family, member L1 | -- |
| 1377102_at | Tmem63a | transmembrane protein 63a | -- |
| 1374644_at | Gpr180 | NOTFOUND | -- |
| 1372246_at | Ostf1 | osteoclast stimulating factor 1 | Signaling |
| 1370087_at | Rab2a | RAB2A, member RAS oncogene family | Signaling |
| 1388119_at | Hnrnpa3 | heterogeneous nuclear ribonucleoprotein A3 | -- |
| 1377334_at | RT1-Ba | RT1 class II, locus Ba | -- |
| 1375673_at | LOC100912399 | mitogen-activated protein kinase kinase kinase 1-like | -- |
| 1370366_at | Timm10 | translocase of inner mitochondrial membrane 10 | mRNA/Protein processing |
| 1375246_at | LOC100910678 /// LOC100911762 /// Tma7 | coiled-coil domain-containing protein 72-like | -- |
| 1387063_at | Ip6k2 | inositol hexakisphosphate kinase 2 | Metabolism |
| 1373746_at | Wdr91 | WD repeat domain 91 | -- |
| 1369943_at | Tgm2 | Transglutaminase 2 | mRNA/Protein processing |
| 1371705_at | Vps26a | VPS26 retromer complex component A | mRNA/Protein processing |
| 1390385_at | Glce | glucuronic acid epimerase | -- |
| 1389832_at | Gsto1 | glutathione S-transferase omega 1 | -- |
| 1389969_at | Tomm40 | Translocase Of Outer Mitochondrial Membrane 40 | -- |
| 1388647_at | Chchd7 | coiled-coil-helix-coiled-coil-helix domain containing 7 | -- |
| 1371707_at | Tnpo2 | transportin 2 | mRNA/Protein processing |
| 1371967_at | Mrpl16 | mitochondrial ribosomal protein L16 | Mitochondrial |
| 1371997_at | Akr1e2 | aldo-keto reductase family 1, member E2 | -- |
| 1387809_at | Map2k6 | mitogen-activated protein kinase kinase 6 | Cell cycle/Apoptosis |
| 1389000_at | Kdm3b | lysine demethylase 3B | -- |
| 1370276_at | Atp5o | ATP synthase, H+ transporting, mitochondrial F1 complex, O subunit | Metabolism |
| 1374791_at | Snx21 | Sorting Nexin Family Member 21 | -- |
| 1370465_at | Abcb1a | ATP binding cassette subfamily B member 1A | -- |
| 1372876_at | Sephs2 | selenophosphate synthetase 2 | -- |
| 1387185_at | Apbb3 | amyloid beta precursor protein binding family B member 3 | -- |
| 1370327_at | Commd5 /// LOC100910472 | COMM domain containing 5 | Cell cycle/Apoptosis |
| 1390035_at | Diexf | digestive organ expansion factor homolog | Metabolism |
| 1398784_at | C1qbp | complement C1q binding protein | Transport |
| 1367723_a_at | Sh2b3 | SH2B adaptor protein 3 | Inflammation/Immune response |
| 1371410_at | Wdr83os | WD repeat domain 83 opposite strand | -- |
| 1373155_at | Mrpl46 | mitochondrial ribosomal protein L46 | -- |
| 1373121_at | Mrps28 | mitochondrial ribosomal protein S28 | -- |
| 1388397_at | Ebna1bp2 /// LOC100912375 | probable rRNA-processing protein EBP2-like | -- |
| 1376112_a_at | Nutf2 | nuclear transport factor 2 | Transport |
| 1373501_at | Nek7 | NIMA-related kinase 7 | Metabolism |
| 1388442_at | Canx | calnexin | Transport |
| 1388499_at | Timmdc1 | translocase of inner mitochondrial membrane domain containing 1 | -- |
| 1389479_at | Klf3 | Kruppel like factor 3 | Metabolism |
| 1368668_at | Plaa | phospholipase A2, activating protein | Metabolism |
| 1370001_at | Rab8a | RAB8A, member RAS oncogene family | Transport |
| 1368422_at | Meox2 | Mesenchyme homeobox 2 | Cell cycle/Apoptosis |
| 1376579_at | Lap3 | leucine aminopeptidase 3 | -- |
| 1374449_at | Cdca3 | cell division cycle associated 3 | -- |
| 1386971_at | Ppp1r10 | protein phosphatase 1, regulatory subunit 10 | -- |
| 1373479_at | Ppp3ca | protein phosphatase 3 catalytic subunit alpha | Signaling |
| 1372667_at | RGD1359158 | hypothetical protein LOC100362345 | -- |
| 1372947_at | Pls3 | plastin 3 | -- |
| 1373472_at | Actr6 | ARP6 actin-related protein 6 homolog | Transcription/Translation regulation |
| 1374754_at | Srgap2 | SLIT-ROBO Rho GTPase activating protein 2 | Transport |
| 1387041_at | Ubqln1 | ubiquilin 1 | Metabolism |
| 1398308_at | Rpa3 | replication protein A3 | Transcription/Translation regulation |
| 1388756_at | Ppcs | phosphopantothenoylcysteine synthetase | -- |
| 1388431_at | Ss18 | SS18, nBAF chromatin remodeling complex subunit | Metabolism |
| 1368321_at | Egr1 | early growth response 1 | Signaling |
| 1367589_at | Aco2 | aconitase 2 | Metabolism |
| 1370487_a_at | Kalrn | kalirin, RhoGEF kinase | Signaling |
| 1371661_at | Mrps23 | mitochondrial ribosomal protein S23 | -- |
| 1373898_at | Pan3 | PAN3 Poly(A) Specific Ribonuclease Subunit | -- |
| 1372116_at | Mrps2 | mitochondrial ribosomal protein S2 | -- |
| 1370311_at | Eif2b1 | eukaryotic translation initiation factor 2B subunit 1 alpha | -- |
| 1388548_at | Fam126b | family with sequence similarity 126, member B | -- |
| 1389569_at | Brix1 | BRX1, biogenesis of ribosomes | -- |
| 1372320_at | Msl3 | Male-specific lethal-3 homolog 1 | Transcription/Translation regulation |
| 1370910_at | Rfc2 | replication factor C subunit 2 | Metabolism |
| 1370375_at | Gls2 | glutaminase 2 | Metabolism |
| 1371348_at | Psmb5 | proteasome subunit beta 5 | Metabolism |
| 1376407_a_at | Lsm7 | LSM7 homolog, U6 small nuclear RNA and mRNA degradation associated | Metabolism |
| 1370193_at | LOC100365697 /// LOC103693189 /// Ptp4a1 | protein tyrosine phosphatase 4a1-like | Metabolism |
| 1390699_at | RGD1311595 | similar to KIAA2026 protein | -- |
| 1389654_at | Pls1 | plastin 1 | -- |
| 1389200_at | Bysl | bystin-like | Metabolism |
| 1370285_at | Calcoco1 /// LOC100912282 | calcium-binding and coiled-coil domain-containing protein 1-like | -- |
| 1373206_at | Fndc3b | Fibronectin Type III Domain Containing 3B | -- |
| 1370317_at | Alg10 | ALG10, alpha-1,2-glucosyltransferase | Transport |
| 1373738_at | Mphosph6 | M phase phosphoprotein 6 | Metabolism |
| 1367761_at | Ndel1 | nudE neurodevelopment protein 1-like 1 | Cell cycle/Apoptosis |
| 1373104_at | Specc1l | sperm antigen with calponin homology and coiled-coil domains 1-like | -- |
| 1371986_at | Anp32a | Acidic Nuclear Phosphoprotein 32 Family Member A | -- |
| 1370348_at | Ninj1 | ninjurin 1 | Metabolism |
| 1389450_at | Wbscr22 | Williams Beuren syndrome chromosome region 22 | Metabolism |
| 1392484_at | LOC100233176 | GON7, KEOPS complex subunit homolog | -- |
| 1375056_at | Ociad2 | -- | EST/Unknown |
| 1370570_at | Nrp1 | Neuropilin 1 | Signaling |
| 1367873_at | Atp6ap1 | ATPase H+ transporting accessory protein 1 | Metabolism |
| 1373815_at | Lman2 | lectin, mannose-binding 2 | Transport |
| 1374303_at | Alkbh2 | alkB homolog 2, alpha-ketoglutarate-dependent dioxygenase | -- |
| 1390255_at | Ablim3 | Actin binding lim protein family, 3 | Other |
| 1368446_at | Spink3 | serine peptidase inhibitor, Kazal type 3 | -- |
| 1367854_at | Acly | ATP citrate lyase | Metabolism |
| 1374318_at | Brcc3 | BRCA1/BRCA2-containing complex, subunit 3 | Metabolism |
| 1387243_at | Cyp1a2 | cytochrome P450, family 1, subfamily a, polypeptide 2 | -- |
| 1371027_at | Cblb | Cbl proto-oncogene B | Metabolism |
| 1384417_at | Apoc4 | apolipoprotein C4 | -- |
| 1398860_at | Nedd8 | neural precursor cell expressed, developmentally down-regulated 8 | Metabolism |
| 1398300_at | Atp1b3 | ATPase Na+/K+ transporting subunit beta 3 | Metabolism |
| 1375138_at | Timp3 | TIMP metallopeptidase inhibitor 3 | Cytoskeleton/ECM |
| 1370376_a_at | Ybx3 | Y box binding protein 3 | -- |
| 1374767_at | Npepo | aminopeptidase O | -- |
| 1388393_at | Plp2 | proteolipid protein 2 | -- |
| 1371983_at | Josd1 | Josephin domain containing 1 | Metabolism |
| 1398788_at | Pdia3 | protein disulfide isomerase family A, member 3 | Signaling |
| 1390435_at | Smim4 | small integral membrane protein 4 | -- |
| 1389625_at | Chchd4 /// LOC100361898 | coiled-coil-helix-coiled-coil-helix domain containing 4 | -- |
| 1388523_at | Txndc12 | thioredoxin domain containing 12 | Transcription/Translation regulation |
| 1368600_at | Slc26a1 | solute carrier family 26 member 1 | -- |
| 1373475_at | Ccdc58 | coiled-coil domain containing 58 | -- |
| 1388643_at | Fut8 | Fucosyltransferase 8 | -- |
| 1367789_at | Slc27a1 | solute carrier family 27 member 1 | -- |
| 1391437_at | Cptp | ceramide-1-phosphate transfer protein | Transport |
| 1376627_at | C1galt1 | core 1 synthase, glycoprotein-N-acetylgalactosamine 3-beta-galactosyltransferase, 1 | Metabolism |
| 1371629_at | Cxxc5 /// LOC681300 | CXXC finger protein 5 | Transcription/Translation regulation |
| 1388822_at | Scoc | short coiled-coil protein | -- |
| 1368142_at | Anxa7 | annexin A7 | Metabolism |
| 1377049_at | Pnpla7 | patatin-like phospholipase domain-containing protein 7-like | -- |
| 1368074_at | Gale | UDP-galactose-4-epimerase | Metabolism |
| 1388612_at | Ociad1 | OCIA domain containing 1 | -- |
| 1386904_a_at | Cyb5a | cytochrome b5 type A | Metabolism |
| 1387503_at | Cpn1 | carboxypeptidase N subunit 1 | Metabolism |
| 1368835_at | Stat1 /// Stat4 | signal transducer and activator of transcription 1 | -- |
| 1388619_at | Rdx | radixin | -- |
| 1387557_s_at | Vps33a | VPS33A CORVET/HOPS core subunit | -- |
| 1387048_at | Ddx39a | DExD-box helicase 39A | -- |
| 1374476_at | LOC100366044 | FMR1 autosomal homolog 1 | Transport |
| 1372215_at | Mrps11 | mitochondrial ribosomal protein S11 | Metabolism |
| 1386910_a_at | Apex1 | apurinic/apyrimidinic endodeoxyribonuclease 1 | Metabolism |
| 1374692_at | Snx14 | sorting nexin 14 | -- |
| 1387958_at | Akr1c3 | aldo-keto reductase family 1, member C3 | Metabolism |
| 1389336_at | Pop5 | POP5 homolog, ribonuclease P/MRP subunit | Metabolism |
| 1373650_at | Cmas | cytidine monophosphate N-acetylneuraminic acid synthetase | Metabolism |
| 1367473_at | Tomm22 | translocase of outer mitochondrial membrane 22 | Metabolism |
| 1371041_at | Ndufv2 | NADH:ubiquinone oxidoreductase core subunit V2 | Transport |
| 1372290_at | Nelfe | negative elongation factor complex member E | Transcription/Translation regulation |
| 1367946_at | Pdlim1 | PDZ and LIM domain 1 | Cytoskeleton/ECM |
| 1373152_at | Prss23 | protease, serine, 23 | -- |
| 1374574_at | Fam214a | family with sequence similarity 214, member A | -- |
| 1390237_at | Timm8a1 | translocase of inner mitochondrial membrane 8 homolog A1 | -- |
| 1391078_at | Rfc1 | replication factor C subunit 1 | -- |
| 1374690_at | Gle1 | GLE1 RNA export mediator | Transcription/Translation regulation |
| 1375504_at | Polg2 | DNA polymerase gamma 2, accessory subunit | Metabolism |
| 1370304_at | Timm17a /// Timm17al1 | Translocase of inner mitochondrial membrane 17a | Transport |
| 1367642_at | Suclg1 | succinate-CoA ligase, alpha subunit | Metabolism |
| 1388946_at | Rbfa | ribosome binding factor A | -- |
| 1388387_at | Ubac1 | UBA domain containing 1 | -- |
| 1367715_at | Tnfrsf1a | TNF receptor superfamily member 1A | Metabolism |
| 1367727_at | Cyth2 | cytohesin 2 | -- |
| 1372562_at | MGC94207 | similar to RIKEN cDNA C030006K11 | -- |
| 1370870_at | Me1 | malic enzyme 1 | Metabolism |
| 1372950_at | Bet1l | Bet1 golgi vesicular membrane trafficking protein-like | Transport |
| 1368934_at | Cyp4a1 | cytochrome P450, family 4, subfamily a, polypeptide 1 | Metabolism |
| 1398976_at | Ncor1 | nuclear receptor co-repressor 1 | -- |
| 1388217_a_at | Calu | calumenin | Transcription/Translation regulation |
| 1373609_at | Mrps21 | mitochondrial ribosomal protein S21 | -- |
| 1372260_at | Rogdi | rogdi homolog | -- |
| 1372878_at | Zfr | zinc finger RNA binding protein | -- |
| 1372105_at | Micu2 | mitochondrial calcium uptake 2 | -- |
| 1369772_at | Slc6a9 | solute carrier family 6 member 9 | Signaling |
| 1375844_at | Ptbp2 | polypyrimidine tract binding protein 2 | -- |
| 1374642_at | Zfp64 | zinc finger protein 64 | Metabolism |
| 1367579_a_at | LOC100909441 /// Tuba1a /// Tuba1b /// Tuba1c | tubulin alpha-1C chain-like | Cell cycle/Apoptosis |
| 1398753_at | Akr1a1 | aldo-keto reductase family 1 member A1 | -- |
| 1398917_at | Rpl7 | ribosomal protein L7 | -- |
| 1389545_at | Tbl1xr1 | transducin -like 1 X-linked receptor 1 | Metabolism |
| 1376501_at | Arhgap8 | Rho GTPase activating protein 8 | Signaling |
| 1374912_at | Kif2c | kinesin family member 2C | Metabolism |
| 1367770_at | Degs1 | delta-desaturase, sphingolipid 1 | Metabolism |
| 1389032_at | Mcoln1 | mucolipin 1 | Transport |
| 1376435_at | Loxl4 | NOTFOUND | -- |
| 1373264_at | Zbed3 | zinc finger, BED-type containing 3 | -- |
| 1373224_at | St3gal4 | ST3 beta-galactoside alpha-2,3-sialyltransferase 4 | -- |
| 1375898_at | Rbpms | -- | EST/Unknown |
| 1367807_at | Plod1 | procollagen-lysine, 2-oxoglutarate 5-dioxygenase 1 | -- |
| 1390382_at | Hypk | Huntingtin interacting protein K | -- |
| 1372681_at | Stxbp6 | syntaxin binding protein 6 | mRNA/Protein processing |
| 1386996_at | Myl12b | myosin light chain 12B | Transcription/Translation regulation |
| 1374897_at | Alyref | Aly/REF export factor | Transport |
| 1372413_at | Mcrs1 | microspherule protein 1 | Metabolism |
| 1389303_at | Mif4gd | MIF4G domain containing | Metabolism |
| 1372066_at | Fam103a1 | family with sequence similarity 103, member A1 | -- |
| 1369969_at | Parp1 | poly polymerase 1 | Metabolism |
| 1387779_at | Mybbp1a | MYB binding protein 1a | Cell cycle/Apoptosis |
| 1374840_at | Ppih | peptidylprolyl isomerase H | -- |
| 1373270_at | Wipi1 | WD repeat domain, phosphoinositide interacting 1 | Cytoskeleton/ECM |
| 1370947_at | Setd4 | SET domain containing 4 | Metabolism |
| 1371813_at | Hirip3 | HIRA interacting protein 3 | -- |
| 1387826_at | RGD1566085 | similar to pyridoxal kinase | -- |
| 1369690_at | Nsf | N-ethylmaleimide sensitive factor, vesicle fusing ATPase | mRNA/Protein processing |
| 1377021_at | Trmt6 | tRNA methyltransferase 6 | -- |
| 1367932_at | Hmgcs1 | 3-hydroxy-3-methylglutaryl-CoA synthase 1 | Metabolism |
| 1371317_at | Ldb1 | LIM domain binding 1 | Transcription/Translation regulation |
| 1388622_at | Nop56 | NOP56 ribonucleoprotein | -- |
| 1371299_at | Rps3 | ribosomal protein S3 | Metabolism |
| 1371798_at | Gna12 | G protein subunit alpha 12 | Metabolism |
| 1372674_at | Rybp | RING1 and YY1 binding protein | -- |
| 1398792_at | Psmc1 | proteasome 26S subunit, ATPase 1 | Metabolism |
| 1388542_at | Use1 | unconventional SNARE in the ER 1 | -- |
| 1388414_at | LOC100363268 /// Ndufs5 | rCG31129-like | -- |
| 1374793_at | Wdr3 | WD repeat domain 3 | Metabolism |
| 1368117_at | Gphn | gephyrin | Metabolism |
| 1368467_at | Cyp4f1 | cytochrome P450, family 4, subfamily f, polypeptide 1 | -- |
| 1369304_at | Pts | 6-pyruvoyl-tetrahydropterin synthase | Metabolism |
| 1398758_at | Arf4 | ADP-ribosylation factor 4 | mRNA/Protein processing |
| 1376588_at | Mtmr12 | myotubularin related protein 12 | Transport |
| 1389648_at | Ripk4 | receptor-interacting serine-threonine kinase 4 | -- |
| 1368115_at | Cldn3 | claudin 3 | -- |
| 1372101_at | Plpp3 | phospholipid phosphatase 3 | Cytoskeleton/ECM |
| 1390501_at | Scrn3 | secernin 3 | Transport |
| 1372885_at | Cebpg | CCAAT/enhancer binding protein gamma | -- |
| 1371982_at | Dpy30 | dpy-30 histone methyltransferase complex regulatory subunit | mRNA/Protein processing |
| 1370888_at | Cox5a | Cytochrome c oxidase subunit 5A, mitochondrial-like | -- |
| 1374227_at | Ttc27 | tetratricopeptide repeat domain 27 | Signaling |
| 1388734_at | LOC102546572 | zinc finger protein 709-like | -- |
| 1367906_at | Acp2 | acid phosphatase 2, lysosomal | -- |
| 1370066_at | Keap1 | Kelch-like ECH-associated protein 1 | -- |
| 1375297_at | Apoo | apolipoprotein O | -- |
| 1371723_at | Rragc | Ras-related GTP binding C | Signaling |
| 1373185_at | Ssr2 | signal sequence receptor subunit 2 | Signaling |
| 1368038_at | Synj2bp | synaptojanin 2 binding protein | Signaling |
| 1387857_at | LOC100910446 /// Stx7 | syntaxin-7-like | -- |
| 1371791_at | LOC102553670 /// Surf4 | Surfeit 4 | Vesicular transport |
| 1376496_at | Apol9a | apolipoprotein L 9a | -- |
| 1374375_at | RGD1560925 | similar to 2610034M16Rik protein | -- |
| 1376660_at | Manea | mannosidase, endo-alpha | -- |
| 1372192_at | Rprd1a | regulation of nuclear pre-mRNA domain containing 1A | -- |
| 1374805_at | Dscc1 | DNA replication and sister chromatid cohesion 1 | -- |
| 1387962_at | Zfp180 | zinc finger protein 180 | Metabolism |
| 1390697_at | Gemin8 | Gem Nuclear Organelle Associated Protein 8 | Transcription/Translation regulation |
| 1367502_at | Mrpl21 | mitochondrial ribosomal protein L21 | -- |
| 1373214_at | Kdelc1 | KDEL motif containing 1 | Metabolism |
| 1388372_at | Rpl35 | ribosomal protein L35 | Metabolism |
| 1371939_at | Caprin1 | cell cycle associated protein 1 | -- |
| 1388469_at | Igf1 | NOTFOUND | -- |
| 1371515_at | Elof1 | elongation factor 1 homolog | Transcription/Translation regulation |
| 1374425_at | Tle1 | transducin like enhancer of split 1 | Metabolism |
| 1376347_at | Kdm5b | lysine demethylase 5B | -- |
| 1371352_at | Hmgn2 /// LOC100360316 | high mobility group nucleosomal binding domain 2 | -- |
| 1388454_at | Oaz2 | ornithine decarboxylase antizyme 2 | Metabolism |
| 1371660_at | Znhit1 | zinc finger, HIT-type containing 1 | Cell cycle/Apoptosis |
| 1369414_at | Stxbp3 | syntaxin binding protein 3 | Signaling |
| 1367465_at | Dad1 | defender against cell death 1 | Metabolism |
| 1388945_at | Pxdc1 | PX domain containing 1 | -- |
| 1372390_at | Peg3 | paternally expressed 3 | Cell cycle/Apoptosis |
| 1370030_at | Gclm | glutamate cysteine ligase, modifier subunit | Metabolism |
| 1372027_at | Spata13 | spermatogenesis associated 13 | Cytoskeleton/ECM |
| 1369663_at | Ephx2 | epoxide hydrolase 2 | -- |
| 1399143_at | LOC100909892 /// Ube2n | ubiquitin-conjugating enzyme E2N | -- |
| 1374007_at | Slu7 | SLU7 Homolog, Splicing Factor | -- |
| 1367832_at | Lypla1 | lysophospholipase I | -- |
| 1370946_at | Nfix | nuclear factor 1 X | Metabolism |
| 1398808_at | Impa1 | inositol monophosphatase 1 | Signaling |
| 1367588_a_at | LOC100363296 /// Rpl13a | ribosomal protein L13A | -- |
| 1389941_at | Arl2bp | ADP-ribosylation factor like GTPase 2 binding protein | Metabolism |
| 1376849_at | LOC100911993 /// Usp48 | Ubiquitin-specific protease 48 | mRNA/Protein processing |
| 1399033_at | Cbfb | core-binding factor, beta subunit | Metabolism |
| 1370215_at | C1qb | complement C1q B chain | Metabolism |
| 1373830_at | LOC619574 | hypothetical protein LOC619574 | -- |
| 1389571_at | Stat2 | signal transducer and activator of transcription 2 | Cell cycle/Apoptosis |
| 1375915_at | Irak1bp1 | interleukin-1 receptor-associated kinase 1 binding protein 1 | Metabolism |
| 1388860_at | Mrpl22 | mitochondrial ribosomal protein L22 | -- |
| 1367602_at | Cited2 | Cbp/p300-interacting transactivator Glu/ Asp-rich carboxy-term Dom 3 | Transcription/Translation regulation |
| 1371755_at | RGD1566359 | similar to RIKEN cDNA B230219D22 | -- |
| 1369554_at | Syngr2 | synaptogyrin 2 | -- |
| 1368158_at | Scfd1 | sec1 family domain containing 1 | Transport |
| 1370475_at | Cyp2b3 | cytochrome P450, family 2, subfamily b, polypeptide 3 | Metabolism |
| 1390326_at | Ang | angiogenin | -- |
| 1387947_at | Mafb | MAF bZIP transcription factor B | -- |
| 1380546_at | Fggy | FGGY carbohydrate kinase domain containing | Metabolism |
| 1383618_at | Mrpl38 | mitochondrial ribosomal protein L38 | -- |
| 1398809_at | Nde1 | nudE neurodevelopment protein 1 | Cell cycle/Apoptosis |
| 1373055_at | Tbcel | tubulin folding cofactor E-like | Transport |
| 1373249_at | Ubl4a | ubiquitin-like 4A | -- |
| 1372661_at | Tbl3 | transducin -like 3 | Metabolism |
| 1376676_a_at | Mphosph8 | M-phase phosphoprotein 8 | -- |
| 1367815_at | Slc5a6 | solute carrier family 5 member 6 | Transport |
| 1371780_at | Kdelr2 | KDEL endoplasmic reticulum protein retention receptor 2 | Transport |
| 1386874_at | Rps15 | ribosomal protein S15 | Transport |
| 1367620_at | Atp5g3 | ATP synthase, H+ transporting, mitochondrial Fo complex, subunit C3 | Metabolism |
| 1376065_at | Rrs1 | ribosome biogenesis regulator homolog | mRNA/Protein processing |
| 1372476_at | Fads3 | fatty acid desaturase 3 | -- |
| 1373510_at | Vamp1 | vesicle-associated membrane protein 1 | -- |
| 1379550_a_at | Gtf2ird1 | general transcription factor II-I repeat domain-containing protein 1-like | Transcription/Translation regulation |
| 1380158_at | Tpd52l2 | Tumor Protein D52 Like 2 | -- |
| 1368102_at | Hsd11b2 | hydroxysteroid 11-beta dehydrogenase 2 | Transcription/Translation regulation |
| 1373653_at | Mtpap | mitochondrial poly polymerase | Cell cycle/Apoptosis |
| 1390279_at | Tssc4 | tumor suppressing subtransferable candidate 4 | -- |
| 1399132_at | Cul3 | cullin 3 | Metabolism |
| 1388574_at | Wars | tryptophanyl-tRNA synthetase | Metabolism |
| 1386859_at | Tkt | transketolase | Metabolism |
| 1399154_at | Kdm2a | lysine demethylase 2A | -- |
| 1383698_at | Pdha1 | pyruvate dehydrogenase alpha 1 | -- |
| 1371297_at | Rpl7a | ribosomal protein L7a | Metabolism |
| 1387765_at | Mbl1 | mannose-binding lectin 1 | Cell cycle/Apoptosis |
| 1388795_at | Tppp | tubulin polymerization promoting protein | -- |
| 1389128_at | Wdfy3 | WD repeat and FYVE domain containing 3 | -- |
| 1380547_at | Clcn3 | chloride voltage-gated channel 3 | -- |
| 1373955_at | Ipo5 | importin 5 | mRNA/Protein processing |
| 1375896_at | Stradb | STE20-related kinase adaptor beta | Cell cycle/Apoptosis |
| 1387188_at | Slc17a1 | solute carrier family 17 member 1 | -- |
| 1370606_at | P2ry1 | purinergic receptor P2Y1 | -- |
| 1398912_at | mrpl9 | mitochondrial ribosomal protein L9 | Mitochondrial |
| 1372375_at | Atg16l1 | autophagy related 16-like 1 | Signaling |
| 1371980_at | Atad3a | ATPase family, AAA domain containing 3A | Metabolism |
| 1398921_at | Mrpl37 | mitochondrial ribosomal protein L37 | -- |
| 1371765_at | Hist3h2a | histone cluster 3, H2a | Transcription/Translation regulation |
| 1389126_at | Chchd1 | coiled-coil-helix-coiled-coil-helix domain containing 1 | Other |
| 1374169_at | Cdip1 | cell death-inducing p53 target 1 | Inflammation/Immune response |
| 1374612_at | Papd5 | poly RNA polymerase D5, non-canonical | -- |
| 1367604_at | Crip2 | cysteine-rich protein 2 | Cell cycle/Apoptosis |
| 1368030_at | Gnai3 | G protein subunit alpha i3 | Metabolism |
| 1372061_at | Rabepk | Rab9 effector protein with kelch motifs | -- |
| 1398326_at | Chchd10 /// LOC103694872 | coiled-coil-helix-coiled-coil-helix domain containing 10 | -- |
| 1398303_s_at | Tpm3 | tropomyosin 3 | Transcription/Translation regulation |
| 1376118_at | Otub2 | OTU deubiquitinase, ubiquitin aldehyde binding 2 | Metabolism |
| 1367756_at | Gfm1 | G elongation factor, mitochondrial 1 | mRNA/Protein processing |
| 1390278_at | Tra2a | transformer 2 alpha homolog | -- |
| 1368671_at | Srpx | sushi-repeat-containing protein, X-linked | -- |
| 1388190_at | Apob | apolipoprotein B | -- |
| 1371725_at | Myh9 /// Myh9l1 | -- | -- |
| 1371489_at | Rnf4 | ring finger protein 4 | Metabolism |
| 1389245_at | Psmd7 | proteasome 26S subunit, non-ATPase 7 | Metabolism |
| 1372535_at | Tceanc2 | transcription elongation factor A N-terminal and central domain containing 2 | Metabolism |
| 1377014_at | Nim1k | NIM1 serine/threonine protein kinase | Signaling |
| 1376646_at | Popdc2 | Popeye domain containing 2 | Other |
| 1371310_s_at | Serpinh1 | serpin family H member 1 | -- |
| 1382944_at | Ero1b | endoplasmic reticulum oxidoreductase 1 beta | -- |
| 1372372_at | Cmbl | carboxymethylenebutenolidase homolog | -- |
| 1376636_at | Tgfbr1 | transforming growth factor, beta receptor 1 | Transcription/Translation regulation |
| 1398752_at | Sep15 | selenoprotein 15 | -- |
| 1367587_at | LOC103689989 /// Prl3d4 | prolactin family 3, subfamily d, member 4 | Signaling |
| 1373147_at | Fbxl3 | F-box and leucine-rich repeat protein 3 | mRNA/Protein processing |
| 1376275_at | Arl5b | ADP Ribosylation Factor Like GTPase 5B | -- |
| 1389351_at | Lrrfip1 | LRR binding FLII interacting protein 1 | Metabolism |
| 1376437_at | Derl2 | derlin 2 | -- |
| 1369927_at | Mdh2 | malate dehydrogenase 2 | Metabolism |
| 1388700_at | Foxn3 | forkhead box N3 | -- |
| 1371596_at | Rnps1 | RNA binding protein S1 | Metabolism |
| 1373685_at | Ankrd37 | ankyrin repeat domain 37 | -- |
| 1389096_at | Mical2 | microtubule associated monooxygenase, calponin and LIM domain containing 2 | -- |
| 1369679_a_at | Nfia | nuclear factor I/A | Metabolism |
| 1387870_at | Zfp36 | zinc finger protein 36 | Metabolism |
| 1375612_at | Hnrnpa1 | Heterogeneous nuclear ribonucleoprotein a1 | mRNA/Protein processing |
| 1368002_at | Msh2 | mutS homolog 2 | Cell cycle/Apoptosis |
| 1374006_at | Ccbl2 | kynurenine aminotransferase 3 | Metabolism |
| 1374416_at | Coa4 | Coiled-coil-helix-coiled-coil-helix domain containing 8 | EST/Unknown |
| 1373983_at | Rsrc2 | arginine and serine rich coiled-coil 2 | -- |
| 1398938_at | Acp1 /// LOC102549052 | acid phosphatase 1, soluble | Signaling |
| 1371089_at | Gsta3 | glutathione S-transferase alpha 3 | Metabolism |
| 1369421_at | LOC499179 /// Top1 | topoisomerase I | mRNA/Protein processing |
| 1369654_at | Prkaa2 | protein kinase AMP-activated catalytic subunit alpha 2 | Signaling |
| 1372484_at | Ddx5 | DEAD-Box Helicase 5 | -- |
| 1375412_at | Arsb | arylsulfatase B | -- |
| 1371819_at | Hdac5 | histone deacetylase 5 | -- |
| 1387145_at | Gjb1 | Gap junction protein, β 1, 32kda | Other |
| 1389577_at | Cirh1a | UTP4 small subunit processome component | -- |
| 1372769_at | Eif2d | eukaryotic translation initiation factor 2D | Metabolism |
| 1376567_at | Mad1l1 | MAD1 mitotic arrest deficient like 1 | Cell cycle/Apoptosis |
| 1387982_at | Tlr4 | toll-like receptor 4 | Metabolism |
| 1371972_at | Sun2 | Sad1 and UNC84 domain containing 2 | mRNA/Protein processing |
| 1373006_at | Tmem171 | transmembrane protein 171 | -- |
| 1373244_at | Tmub2 | transmembrane and ubiquitin-like domain containing 2 | -- |
| 1367724_a_at | Atp6v0e1 | ATPase H+ transporting V0 subunit e1 | Transport |
| 1374512_at | Glyctk | glycerate kinase | Metabolism |
| 1377194_a_at | Ccdc90b | Coiled-coil domain containing 90b | EST/Unknown |
| 1387521_at | Pdcd4 | programmed cell death 4 | -- |
| 1371790_at | Mrpl45 | mitochondrial ribosomal protein L45 | -- |
| 1373610_at | Sec24d | SEC24 homolog D, COPII coat complex component | Transport |
| 1388294_at | Sdhd | succinate dehydrogenase complex subunit D | Transport |
| 1372517_at | Ppil1 | peptidylprolyl isomerase like 1 | Metabolism |
| 1372198_at | LOC100910540 /// Mepce | 7SK snRNA methylphosphate capping enzyme-like | Metabolism |
| 1370462_at | Hmmr | hyaluronan-mediated motility receptor | -- |
| 1371488_at | LOC100912030 /// RGD1562079 | succinate dehydrogenase assembly factor 1, mitochondrial-like | -- |
| 1370316_at | Hspbp1 | HSPA binding protein, cytoplasmic cochaperone 1 | Metabolism |
| 1389358_at | Lpgat1 | Lysophosphatidylglycerol Acyltransferase 1 | -- |
| 1375181_at | Rpl12 | Ribosomal protein L12 | Transcription/Translation regulation |
| 1389286_at | Glrx5 | glutaredoxin 5 | -- |
| 1371384_at | Btf3 | basic transcription factor 3 | -- |
| 1389682_at | Chac2 | ChaC cation transport regulator 2 | Metabolism |
| 1399141_at | Clk4 | CDC-like kinase 4 | -- |
| 1375080_at | Slc25a45 | solute carrier family 25, member 45 | Transport |
| 1372433_at | Ngdn | neuroguidin | Metabolism |
| 1367908_at | Gcsh | glycine cleavage system protein H | -- |
| 1371764_at | Ube2j2 | ubiquitin-conjugating enzyme E2, J2 | -- |
| 1398760_at | LOC100359498 /// LOC103690996 /// Rpl35a | ribosomal protein L35a-like | -- |
| 1387912_at | Ddx46 | DEAD-box helicase 46 | mRNA/Protein processing |
| 1387267_at | Ntf3 | neurotrophin 3 | Cell cycle/Apoptosis |
| 1376098_a_at | LOC102547700 | uncharacterized LOC102547700 | -- |
| 1372462_at | Acat2 | acetyl-CoA acetyltransferase 2 | Metabolism |
| 1388132_at | Sfpq | splicing factor proline and glutamine rich | -- |
| 1388314_at | Hmgn1 /// LOC100911295 /// LOC691642 | non-histone chromosomal protein HMG-14-like | Cell cycle/Apoptosis |
| 1372526_at | Flcn | folliculin | -- |
| 1398751_at | Rps7 | ribosomal protein S7-like | Metabolism |
| 1375357_at | Tor1a | torsin family 1, member A | Metabolism |
| 1389017_at | Antxr2 | Anthrax toxin receptor 2 | Signaling |
| 1392900_at | Capza1 | Capping protein muscle z-line, α 1 | Cytoskeleton/ECM |
| 1377935_at | Cldnd1 | claudin domain containing 1 | -- |
| 1373598_at | Ubn1 | ubinuclein 1 | -- |
| 1370200_at | Glud1 | glutamate dehydrogenase 1 | Metabolism |
| 1373970_at | Il33 | interleukin 33 | Transcription/Translation regulation |
| 1376100_at | Tubb6 | tubulin, beta 6 class V | Cell cycle/Apoptosis |
| 1371147_at | Serpina3m | serine proteinase inhibitor, clade A, member 3M | -- |
| 1388684_at | Fnbp4 | formin binding protein 4 | -- |
| 1371427_at | Spcs1 | signal peptidase complex subunit 1 | -- |
| 1367833_at | Psmc5 | proteasome 26S subunit, ATPase 5 | -- |
| 1367676_at | Hmgb2 /// Hmgb2l1 /// RGD1559962 | high mobility group protein B2-like | -- |
| 1377302_a_at | Mmaa | methylmalonic aciduria cblA type | -- |
| 1373165_at | Zmat1 | zinc finger, matrin-type 1 | -- |
| 1368228_at | Zranb2 | Zinc finger, ran-binding domain containing 2 | mRNA/Protein processing |
| 1388741_at | Cmya5 | cardiomyopathy associated 5 | -- |
| 1374349_at | Ctdspl | Ctd small phosphatase-like | Transcription/Translation regulation |
| 1371709_at | Mrpl3 | mitochondrial ribosomal protein L3 | -- |
| 1388114_at | Myl12a | myosin light chain 12A | Transcription/Translation regulation |
| 1371955_at | Mrpl35 | mitochondrial ribosomal protein L35 | -- |
| 1373037_at | Ube2l6 | ubiquitin-conjugating enzyme E2L 6 | Metabolism |
| 1375463_at | Ski | SKI Proto-Oncogene | -- |
| 1372709_at | Bcap29 | B-cell receptor-associated protein 29 | mRNA/Protein processing |
| 1372218_at | Wdr12 | WD repeat domain 12 | Metabolism |
| 1374396_at | Atp6v1c1 | ATPase H+ transporting V1 subunit C1 | Metabolism |
| 1375663_at | Ube2f | Ubiquitin-conjugating enzyme e2f | mRNA/Protein processing |
| 1388105_at | Cdc123 | cell division cycle 123 | Transcription/Translation regulation |
| 1373279_at | Ctnnbip1 | catenin, beta-interacting protein 1 | -- |
| 1389057_at | Arv1 | ARV1 homolog, fatty acid homeostasis modulator | Transport |
| 1390454_at | Nipsnap1 | nipsnap homolog 1 | -- |
| 1371973_at | Eif3e | eukaryotic translation initiation factor 3, subunit 6 48kDa-like | -- |
| 1372522_at | Arglu1 | arginine and glutamate rich 1 | Transcription/Translation regulation |
| 1398889_at | Myzap /// Polr2m | RNA polymerase II subunit M | Signaling |
| 1388770_at | Ufm1 | ubiquitin-fold modifier 1 | -- |
| 1371189_x_at | Rpsa | ribosomal protein SA | mRNA/Protein processing |
| 1374973_at | Med25 | mediator complex subunit 25 | Metabolism |
| 1367902_at | Gng11 /// LOC100912034 | Guanine nucleotide binding protein , γ 11 | Signaling |
| 1370549_at | Vps45 | vacuolar protein sorting 45 | Transport |
| 1373467_at | Btaf1 | B-TFIID TATA-box binding protein associated factor 1 | -- |
| 1371599_at | LOC294154 | similar to chromosome 6 open reading frame 106 isoform a | -- |
| 1367728_at | Tsn | translin | -- |
| 1388805_at | LOC103694903 | protein phosphatase 2, catalytic subunit, alpha isozyme | Signaling |
| 1372175_at | Reep3 | receptor accessory protein 3 | -- |
| 1372754_at | Appl2 | adaptor protein, phosphotyrosine interacting with PH domain and leucine zipper 2 | -- |
| 1373380_at | Zc3h15 | zinc finger CCCH-type containing 15 | Metabolism |
| 1398872_at | LOC683961 /// LOC684988 /// Rps13 | ribosomal protein S13 | Signaling |
| 1376754_at | Cars | cysteinyl-tRNA synthetase | Metabolism |
| 1373452_at | Rcl1 | RNA terminal phosphate cyclase-like 1 | -- |
| 1367656_at | Psmb7 | proteasome subunit beta 7 | Metabolism |
| 1399022_at | Clk1 | CDC-like kinase 1 | -- |
| 1373041_at | Ndufb3 | NADH:ubiquinone oxidoreductase subunit B3 | Mitochondrial |
| 1388352_at | Naa20 | N-acetyltransferase 20, NatB catalytic subunit | Metabolism |
| 1388526_at | Gstz1 | glutathione S-transferase zeta 1 | Metabolism |
| 1371683_at | Lsm4 | LSM4 homolog, U6 small nuclear RNA and mRNA degradation associated | Transcription/Translation regulation |
| 1374704_at | Kdelc2 | KDEL motif containing 2 | Metabolism |
| 1376001_at | Polr1e | RNA polymerase I subunit E | Metabolism |
| 1387116_at | Dnajb9 | DnaJ heat shock protein family member B9 | -- |
| 1371529_at | Ripk1 | receptor interacting serine/threonine kinase 1 | Metabolism |
| 1389372_at | Dennd4c /// LOC102555797 | uncharacterized LOC102555797 | -- |
| 1378016_at | Eml4 | echinoderm microtubule associated protein like 4 | -- |
| 1388341_at | Rangap1 | RAN GTPase activating protein 1 | -- |
| 1388370_at | Ccni | cyclin I | Cell cycle/Apoptosis |
| 1372197_at | Rictor | RPTOR independent companion of MTOR, complex 2 | Cytoskeleton/ECM |
| 1370167_at | Sdc2 | syndecan 2 | Transport |
| 1389170_at | Casp7 | caspase 7 | Signaling |
| 1370111_at | Kcnn2 | Potassium Calcium-Activated Channel Subfamily N Member 2 | -- |
| 1374065_at | Met | Met proto-oncogene | Signaling |
| 1371855_at | Camk2g /// NEWGENE_621802 | -- | -- |
| 1388450_at | Synrg | Synergin Gamma | -- |
| 1369435_at | Ttpa | alpha-tocopherol transfer protein-like | -- |
| 1389293_at | Cpsf2 | cleavage and polyadenylation specific factor 2 | -- |
| 1370809_at | Tubg1 /// Tubg2 | tubulin, gamma 1 | Cell cycle/Apoptosis |
| 1375432_at | Exosc1 | exosome component 1 | -- |
| 1373664_at | Pigc | phosphatidylinositol glycan anchor biosynthesis, class C | -- |
| 1368762_at | Ubd | ubiquitin D | Transcription/Translation regulation |
| 1376737_at | LOC100912041 | uncharacterized LOC100912041 | -- |
| 1390026_at | Bag3 | Bcl2-associated athanogene 3 | Cell cycle/Apoptosis |
| 1373441_at | Dctn5 | dynactin subunit 5 | -- |
| 1372324_at | Znhit3 | zinc finger, HIT-type containing 3 | -- |
| 1371843_at | Yipf5 | Yip1 domain family, member 5 | Transcription/Translation regulation |
| 1373686_at | Serpina6 | serpin family A member 6 | -- |
| 1373269_at | Smim13 | Small Integral Membrane Protein 13 | -- |
| 1398953_at | Tsta3 | tissue specific transplantation antigen P35B | Signaling |
| 1372501_at | Sf3b3 | splicing factor 3b, subunit 3 | Metabolism |
| 1369430_at | Bco1 | beta-carotene oxygenase 1 | Signaling |
| 1371754_at | Slc25a25 | solute carrier family 25 member 25 | Transport |
| 1373530_at | Ccne1 | cyclin E1 | -- |
| 1371606_at | Wdtc1 | WD and tetratricopeptide repeats 1 | Metabolism |
| 1387894_at | Gata4 | GATA binding protein 4 | Transcription/Translation regulation |
| 1375530_at | Gnpnat1 | glucosamine-phosphate N-acetyltransferase 1 | Metabolism |
| 1371356_at | Tns2 | tensin 2 | Cytoskeleton/ECM |
| 1374705_at | Col4a5 | collagen type IV alpha 5 chain | Cytoskeleton/ECM |
| 1398446_at | Fam189b | family with sequence similarity 189, member B | -- |
| 1372036_at | Cd2bp2 | Cd2 binding protein 2 | -- |
| 1371876_at | Psmg2 | proteasome assembly chaperone 2 | -- |
| 1398894_at | Commd3 | Comm domain containing 3 | EST/Unknown |
| 1369275_s_at | Cyp2a1 /// Cyp2a2 /// Cyp2a3 | cytochrome P450, family 2, subfamily a, polypeptide 1 | Metabolism |
| 1376016_at | Get4 | golgi to ER traffic protein 4 | mRNA/Protein processing |
| 1370563_at | Akr1c14 | aldo-keto reductase family 1, member C14 | Metabolism |
| 1367673_at | LOC103689947 /// Selenbp1 | selenium binding protein 1 | -- |
| 1367933_at | Amd1 | adenosylmethionine decarboxylase 1 | Metabolism |
| 1368141_at | Cnbp | CCHC-type zinc finger, nucleic acid binding protein | -- |
| 1368064_a_at | Ddc | dopa decarboxylase | Metabolism |
| 1367492_at | Dnajc8 | DnaJ heat shock protein family member C8 | -- |
| 1373824_at | Cfdp1 | Flavin | Other |
| 1372489_at | Slmap | Sarcolemma-associated protein | Transcription/Translation regulation |
| 1375922_at | Cox15 | COX15 cytochrome c oxidase assembly homolog | Mitochondrial |
| 1372516_at | Kif22 | kinesin family member 22 | Transport |
| 1367813_at | Ppp1r14a | protein phosphatase 1, regulatory subunit 14A | Metabolism |
| 1368283_at | Ehhadh | Enoyl-coenzyme A, hydratase/3- hydroxyacyl coenzyme A dehydrogenase | Mitochondrial |
| 1376272_s_at | Tmem167a | transmembrane protein 167A | -- |
| 1374848_at | Gtf3c6 | general transcription factor 3C subunit 6 | Metabolism |
| 1376927_at | Lrrc14b | leucine rich repeat containing 14B | -- |
| 1390717_at | Crls1 | cardiolipin synthase 1 | Metabolism |
| 1372306_at | Ethe1 | ETHE1, persulfide dioxygenase | Metabolism |
| 1389013_at | Rpl7l1 | ribosomal protein L7-like 1 | -- |
| 1398868_at | Timm13 | Translocase of inner | Mitochondrial |
| 1371014_at | Plcb1 | Phospholipase c, β 1 | Signaling |
| 1388343_at | Ndufb7 | NADH:ubiquinone oxidoreductase subunit B7 | Metabolism |
| 1399091_at | Capzb | capping actin protein of muscle Z-line beta subunit | -- |
| 1374568_at | Cipc | CLOCK-interacting pacemaker | -- |
| 1390185_at | Dcps | decapping enzyme, scavenger | Metabolism |
| 1373647_at | LOC103689968 /// LOC103690028 /// Zfp622 | zinc finger protein 622 | -- |
| 1388488_at | Lsm3 | LSM3 homolog, U6 small nuclear RNA and mRNA degradation associated | Metabolism |
| 1372155_at | Trim28 | tripartite motif-containing 28 | -- |
| 1388870_at | Msi2 | musashi RNA-binding protein 2 | -- |
| 1371548_at | Mrps25 | mitochondrial ribosomal protein S25 | -- |
| 1367479_at | Nop10 | NOP10 ribonucleoprotein | -- |
| 1372901_at | Nhlrc3 | NHL repeat containing 3 | -- |
| 1374945_at | LOC103692719 /// Trmt61a | tRNA methyltransferase 61A | Metabolism |
| 1373621_at | Gmpr2 | guanosine monophosphate reductase 2 | Metabolism |
| 1372009_at | Yars | tyrosyl-tRNA synthetase | Metabolism |
| 1398252_at | Mecr | mitochondrial trans-2-enoyl-CoA reductase | Cytoskeleton/ECM |
| 1398957_at | Cdk11b | cyclin-dependent kinase 11B | -- |
| 1367472_at | Uba1 | ubiquitin-like modifier activating enzyme 1 | Metabolism |
| 1375453_at | Gpatch8 | G patch domain containing 8 | -- |
| 1376921_at | Foxred1 | FAD-dependent oxidoreductase domain containing 1 | -- |
| 1369092_at | Sec22a | Sec22 vesicle trafficking protein homolog a | Vesicular transport |
| 1386899_at | Ctsh | cathepsin H | Metabolism |
| 1370824_at | Slc38a3 | solute carrier family 38, member 3 | Transport |
| 1368863_at | Nme3 | NME/NM23 nucleoside diphosphate kinase 3 | -- |
| 1376492_at | Unkl | unkempt family like zinc finger | -- |
| 1372213_at | LOC500300 | -- | EST/Unknown |
| 1368354_at | Gstt1 | glutathione S-transferase theta 1 | Metabolism |
| 1388171_at | Cdk7 | cyclin-dependent kinase 7 | -- |
| 1373277_at | Tm2d3 | TM2 domain containing 3 | Signaling |
| 1374323_at | Bccip | BRCA2 and CDKN1A interacting protein | -- |
| 1374693_at | Parp16 | poly polymerase family, member 16 | Metabolism |
| 1370370_at | Hyal2 | hyaluronoglucosaminidase 2 | -- |
| 1376101_at | Lrp6 | NOTFOUND | -- |
| 1387372_at | Slc6a13 | solute carrier family 6 member 13 | Signaling |
| 1390028_at | Dyrk2 | Dual-specificity tyrosine- (Y)-phosphorylation regulated kinase 2 | Other |
| 1377051_at | LOC100362572 /// LOC103693261 | Mpv17 transgene, kidney disease mutant-like -like | -- |
| 1368220_at | Gtf2b | general transcription factor IIB | Metabolism |
| 1376653_at | Phtf1 | putative homeodomain transcription factor 1 | Metabolism |
| 1389235_at | Icam2 | intercellular adhesion molecule 2 | -- |
| 1368608_at | Cyp2f4 | cytochrome P450, family 2, subfamily f, polypeptide 4 | Metabolism |
| 1372774_at | Entpd5 | ectonucleoside triphosphate diphosphohydrolase 5 | Metabolism |
| 1373240_at | Dhrs3 | Dehydrogenase/reductase 3 | Metabolism |
| 1389866_at | LOC100912557 | 6.8 kDa mitochondrial proteolipid | -- |
| 1375551_at | Ccdc86 | coiled-coil domain containing 86 | Metabolism |
| 1374804_at | Gnl2 | G protein nucleolar 2 | -- |
| 1370144_at | Gtpbp4 /// LOC103690178 /// LOC299312 | GTP binding protein 4 | Inflammation/Immune response |
| 1388937_at | Rnf19a | ring finger protein 19A, RBR E3 ubiquitin protein ligase | Metabolism |
| 1387187_a_at | Nat1 | N-acetyltransferase 1 | -- |
| 1367758_at | Afp | alpha-fetoprotein | -- |
| 1372720_at | Btbd1 | BTB domain containing 1 | Transcription/Translation regulation |
| 1372164_at | Wbp1l | WW domain binding protein 1-like | -- |
| 1372302_at | LOC100911313 /// Rmdn3 | regulator of microtubule dynamics protein 3-like | -- |
| 1376007_at | LOC103690067 /// Tprkb | Tp53rk binding protein | -- |
| 1367706_at | Vdac1 | similar to voltage-dependent anion channel 1 | -- |
| 1367484_at | Ube2e1 /// Ube2e2 | ubiquitin-conjugating enzyme E2E 1 | -- |
| 1368096_at | Rab29 | RAB29, member RAS oncogene family | Signaling |
| 1374764_at | Nop14 | NOP14 nucleolar protein | Metabolism |
| 1389578_at | Yrdc | yrdC N-threonylcarbamoyltransferase domain containing | Transcription/Translation regulation |
| 1373884_at | Klhl2 | kelch-like family member 2 | -- |
| 1387053_at | Fmo1 | Flavin-containing monooxygenase 1 | Small molecule metabolism |
| 1376731_at | Pign | NOTFOUND | -- |
| 1373201_at | Dbt | dihydrolipoamide branched chain transacylase E2 | Metabolism |
| 1388155_at | Krt18 /// LOC683212 | keratin 18 | -- |
| 1390101_at | Ccdc107 | coiled-coil domain containing 107 | -- |
| 1372339_at | Cdc26 | cell division cycle 26 | -- |
| 1389167_at | Mapkap1 | mitogen-activated protein kinase associated protein 1 | -- |
| 1388931_at | Mrpl13 | mitochondrial ribosomal protein L13 | Inflammation/Immune response |
| 1369932_a_at | Raf1 | Raf-1 proto-oncogene, serine/threonine kinase | Metabolism |
| 1367630_at | Rps11 | ribosomal protein S11 | -- |
| 1373597_at | Pop7 | POP7 homolog, ribonuclease P/MRP subunit | -- |
| 1399062_at | Smim15 | small integral membrane protein 15 | -- |
| 1388767_at | Pdcd6 | Programmed cell death | Cell cycle/Apoptosis |
| 1375658_at | Sash1 | SAM and SH3 domain containing 1 | -- |
| 1373533_at | RGD1562037 | similar to OTTHUMP00000046255 | -- |
| 1372867_at | Rnmt | RNA methyltransferase | Metabolism |
| 1375517_at | Tp53inp2 | tumor protein p53 inducible nuclear protein 2 | -- |
| 1371379_at | Mpc2 | mitochondrial pyruvate carrier 2 | Transport |
| 1373014_at | B3gat3 | beta-1,3-glucuronyltransferase 3 | Metabolism |
| 1368625_at | Prap1 | proline-rich acidic protein 1 | -- |
| 1375442_at | Mphosph10 | M-phase phosphoprotein 10 | -- |
| 1389617_at | Elk3 | ELK3, ETS-domain protein | Transcription/Translation regulation |
| 1371535_at | Pmm2 | phosphomannomutase 2 | mRNA/Protein processing |
| 1388487_at | Add1 | adducin 1 | Metabolism |
| 1377060_at | Mccc2 | Methylcrotonoyl-coenzyme a carboxylase 2 | Metabolism |
| 1375338_at | Rab10 | RAB10, member RAS oncogene family | -- |
| 1390027_at | Usp8 | ubiquitin specific peptidase 8 | -- |
| 1387024_at | Dusp6 | dual specificity phosphatase 6 | Metabolism |
| 1398945_at | Ddx6 | DEAD-box helicase 6 | -- |
| 1375363_at | Asxl2 | additional sex combs like 2, transcriptional regulator | Transcription/Translation regulation |
| 1375914_at | Krba1 | KRAB-A domain containing 1 | -- |
| 1373849_at | Actl6a | actin-like 6A | Cell cycle/Apoptosis |
| 1399046_at | Top1 | topoisomerase I | mRNA/Protein processing |
| 1368433_at | Sacm1l | SAC1 suppressor of actin mutations 1-like | -- |
| 1371547_at | Grcc10 /// LOC100911713 | gene rich cluster, C10 gene | -- |
| 1390124_at | RGD1560464 | similar to hypothetical protein FLJ38426 | -- |
| 1372187_at | Prkd3 | protein kinase D3 | -- |
| 1369656_at | Pcyt1a | choline-phosphate cytidylyltransferase A-like | -- |
| 1367480_at | Eif4a3 | DEAD (Asp-Glu-Ala-Asp) box polypeptide 48 | Transcription/Translation regulation |
| 1386478_at | Slc25a52 | solute carrier family 25, member 51 | -- |
| 1367463_at | Phb2 | prohibitin 2 | Cell cycle/Apoptosis |
| 1389559_at | RGD1564420 | similar to Hypothetical protein MGC31278 | Transport |
| 1367454_at | Copb2 | coatomer protein complex subunit beta 2 | mRNA/Protein processing |
| 1373797_at | Nat6 | hyaluronoglucosaminidase 3 | Metabolism |
| 1387669_a_at | Ephx1 | Epoxide hydrolase 1, | Small molecule metabolism |
| 1373758_at | Flad1 | flavin adenine dinucleotide synthetase 1 | Metabolism |
| 1371697_at | LOC100911615 /// Pnpla2 | patatin-like phospholipase domain-containing protein 2-like | Metabolism |
| 1373376_at | Sun1 | Sad1 and UNC84 domain containing 1 | mRNA/Protein processing |
| 1371378_at | Eif1 | eukaryotic translation initiation factor 1 | -- |
| 1389319_at | Ergic1 | Endoplasmic reticulum-golgi intermediate compartment 1 | Vesicular transport |
| 1368232_at | Mvk | mevalonate kinase | Metabolism |
| 1370916_at | Tec | tec protein tyrosine kinase | -- |
| 1372108_at | Ptcd3 | Pentatricopeptide repeat domain 3 | -- |
| 1373097_at | Ercc6 | ERCC excision repair 6, chromatin remodeling factor | -- |
| 1370098_at | Vamp7 | vesicle-associated membrane protein 7 | Transport |
| 1374517_at | Recql | RecQ like helicase | Transcription/Translation regulation |
| 1389918_at | LOC100360205 | palladin | -- |
| 1374679_at | Higd1b | HIG1 hypoxia inducible domain family, member 1B | -- |
| 1374789_at | Dcun1d2 /// NEWGENE_1582994 | defective in cullin neddylation 1 domain containing 2 | Transcription/Translation regulation |
| 1369249_at | Ankh | ANKH inorganic pyrophosphate transport regulator | -- |
| 1390563_at | Abca3 | ATP binding cassette subfamily A member 3 | Transport |
| 1371341_at | Snrpd2 | small nuclear ribonucleoprotein D2 polypeptide | Metabolism |
| 1388358_at | Etfb | electron transfer flavoprotein beta subunit | -- |
| 1371377_at | Rps19l1 | ribosomal protein S19 | mRNA/Protein processing |
| 1390481_a_at | Ube2t | ubiquitin-conjugating enzyme E2T | -- |
| 1372001_at | Fam96b | family with sequence similarity 96, member B | Metabolism |
| 1388164_at | RT1-S3 | RT1 class Ib, locus S3 | Inflammation/Immune response |
| 1368304_at | Fmo3 | flavin containing monooxygenase 3 | Other |
| 1390373_at | Smad5 | SMAD family member 5 | Metabolism |
| 1376140_at | Abcd1 | ATP binding cassette subfamily D member 1 | Transport |
| 1368428_at | Xpnpep2 | X-prolyl aminopeptidase 2, membrane-bound | Cytoskeleton/ECM |
| 1373321_at | Myrf | myelin regulatory factor | Metabolism |
| 1371783_at | Selk | selenoprotein K | Signaling |
| 1371738_at | Ano10 | anoctamin 10 | -- |
| 1392490_at | Tmem57 | Transmembrane protein 57 | EST/Unknown |
| 1387855_at | Gdi1 | GDP dissociation inhibitor 1 | Signaling |
| 1373372_at | Mydgf | myeloid-derived growth factor | -- |
| 1376222_at | Sdr39u1 | short chain dehydrogenase/reductase family 39U, member 1 | Metabolism |
| 1376573_at | Rab34 | RAB34, member RAS oncogene family | Signaling |
| 1377966_at | Paqr7 | progestin and adipoQ receptor family member 7 | Inflammation/Immune response |
| 1390392_at | Tmem185b | transmembrane protein 185B | -- |
| 1387775_at | Gtf2a2 /// LOC103695118 | general transcription factor IIA, 2 | Transcription/Translation regulation |
| 1367903_at | Hmox2 | heme oxygenase 2 | Metabolism |
| 1374109_at | Dgkq | diacylglycerol kinase, theta | -- |
| 1388313_at | Rps25 | 40S ribosomal protein S25-like | -- |
| 1370804_at | Gabarap | GABA type A receptor-associated protein | Signaling |
| 1374932_at | Angptl8 | angiopoietin-like 8 | -- |
| 1370675_at | Trpv1 | transient receptor potential cation channel, subfamily V, member 1 | -- |
| 1376649_at | Sik2 | salt-inducible kinase 2 | Signaling |
| 1390274_at | Plxna2 | Plexin a2 | Signaling |
| 1398761_at | Rpl5 | Ribosomal protein L5 | Transcription/Translation regulation |
| 1370295_at | Nme1 | NME/NM23 nucleoside diphosphate kinase 1 | Transcription/Translation regulation |
| 1376807_at | Rabl3 | RAB, member of RAS oncogene family-like 3 | Signaling |
| 1371422_at | Morf4l2 | mortality factor 4 like 2 | Transcription/Translation regulation |
| 1375965_at | Ube2k | Ubiquitin-conjugating enzyme e2k | mRNA/Protein processing |
| 1389363_at | Adi1 | acireductone dioxygenase 1 | Metabolism |
| 1372160_at | Blcap | Bladder cancer associated protein | Cell cycle/Apoptosis |
| 1369669_at | Nln | neurolysin | Metabolism |
| 1398908_at | Stoml2 | stomatin like 2 | -- |
| 1370213_at | Ybx1 /// Ybx1-ps3 | Y box protein 1 related, pseudogene 3 | -- |
| 1372368_at | Hspa13 | NOTFOUND | -- |
| 1388492_at | Tnip1 | TNFAIP3 interacting protein 1 | -- |
| 1389393_at | Abhd17c | abhydrolase domain containing 17C | -- |
| 1367629_at | Cox7a2 /// Cox7a2l2 | cytochrome c oxidase subunit VIIa polypeptide 2 | Metabolism |
| 1385922_at | Prrc2b | proline-rich coiled-coil 2B | -- |
| 1379321_at | LOC102548944 | uncharacterized LOC102548944 | -- |
| 1388338_at | Ppp2r4 | protein phosphatase 2 phosphatase activator | mRNA/Protein processing |
| 1389964_at | Ndufab1 | NADH dehydrogenase 1, alpha/beta subcomplex, 1 | Metabolism |
| 1368042_a_at | Hmg1l1 /// Hmgb1 /// Hmgb1-ps4 | high mobility group box 1 | Metabolism |
| 1376621_at | Wdr26 | WD repeat domain 26 | -- |
| 1390115_at | Sec63 | SEC63 homolog, protein translocation regulator | mRNA/Protein processing |
| 1371577_at | Ndufs1 | NADH dehydrogenase Fe-S protein 1 | Metabolism |
| 1368436_at | LOC100911422 /// Nudc | nuclear migration protein nudC-like | -- |
| 1370233_at | Fgf12 | fibroblast growth factor 12 | Signaling |
| 1388799_at | Klhl7 | kelch-like family member 7 | -- |
| 1372779_at | B3gnt2 | UDP-GlcNAc:betaGal beta-1,3-N-acetylglucosaminyltransferase 2 | Metabolism |
| 1369590_a_at | Ddit3 | DNA-damage-inducible transcript 3 | Transcription/Translation regulation |
| 1370871_at | Hnrnpa3 /// LOC100361479 /// LOC100911361 | heterogeneous nuclear ribonucleoprotein A3 | -- |
| 1371371_at | Ndufb4 | NADH:ubiquinone oxidoreductase subunit B4 | Metabolism |
| 1367780_at | Pttg1 | pituitary tumor-transforming 1 | Cell cycle/Apoptosis |
| 1386956_at | Scarb1 | scavenger receptor class B, member 1 | Cytoskeleton/ECM |
| 1368452_at | Abcc6 | ATP binding cassette subfamily C member 6 | Metabolism |
| 1372185_at | Ntmt1 | N-terminal Xaa-Pro-Lys N-methyltransferase 1 | Metabolism |
| 1369402_at | Adnp | activity-dependent neuroprotector homeobox | -- |
| 1369485_at | Acot12 | acyl-CoA thioesterase 12 | Metabolism |
| 1372243_at | Cab39 | calcium binding protein 39 | -- |
| 1388790_at | Coq5 | coenzyme Q5, methyltransferase | -- |
| 1373039_at | Tomm70a | translocase of outer mitochondrial membrane 70 | -- |
| 1388366_at | Mrpl4 | mitochondrial ribosomal protein L4 | -- |
| 1367856_at | G6pd | glucose-6-phosphate dehydrogenase | Metabolism |
| 1372676_at | Fahd1 | fumarylacetoacetate hydrolase domain containing 1 | Metabolism |
| 1370300_at | Preb | prolactin regulatory element binding | Metabolism |
| 1388141_at | Cetn3 /// LOC100912538 | centrin-3-like | -- |
| 1398854_at | Rpl24 | ribosomal protein L24 | Transcription/Translation regulation |
| 1381970_at | Lsm14a | LSM14A mRNA processing body assembly factor | Metabolism |
| 1398874_at | Atxn10 | Ataxin 10 | Signaling |
| 1388562_at | Stard7 | StAR Related Lipid Transfer Domain Containing 7 | -- |
| 1370272_x_at | Grpca | glutamine/glutamic acid-rich protein A | -- |
| 1388565_at | Spg21 | spastic paraplegia 21 homolog | Cell cycle/Apoptosis |
| 1373302_at | Acer2 | alkaline ceramidase 2 | Transcription/Translation regulation |
| 1367525_at | Thrap3 | thyroid hormone receptor associated protein 3 | Metabolism |
| 1388716_at | Ehmt2 | euchromatic histone lysine methyltransferase 2 | -- |
| 1371295_at | Rps20 | ribosomal protein S20-like | -- |
| 1390531_at | Helz2 | helicase with zinc finger 2, transcriptional coactivator | -- |
| 1386573_a_at | RGD735065 | NOTFOUND | -- |
| 1389733_at | Mars | methionyl-tRNA synthetase | -- |
| 1368695_at | C4bpb | complement component 4 binding protein, beta | -- |
| 1372067_at | Tmx1 | thioredoxin-related transmembrane protein 1 | Signaling |
| 1376628_at | Zfp189 | zinc finger protein 189 | -- |
| 1371904_at | Smyd2 | SET and MYND domain containing 2 | Metabolism |
| 1376214_at | Mmachc | Methylmalonic aciduria cblc type, with homocystinuria | Transport |
| 1372521_at | Rnd2 | Rho family GTPase 2 | Signaling |
| 1373074_at | Timm21 | translocase of inner mitochondrial membrane 21 | mRNA/Protein processing |
| 1374067_at | Cacul1 | CDK2-associated, cullin domain 1 | -- |
| 1371965_at | Mmadhc | methylmalonic aciduria and homocystinuria, cblD type | -- |
| 1368244_at | As3mt | arsenite methyltransferase | -- |
| 1372916_at | Mrps27 | mitochondrial ribosomal protein S27 | -- |
| 1375186_at | Dph3 | diphthamide biosynthesis 3 | Metabolism |
| 1372830_at | Smg1 | SMG1, Nonsense Mediated MRNA Decay Associated PI3K Related Kinase | -- |
| 1377118_at | Cep44 | centrosomal protein 44 | -- |
| 1388974_at | Srp19 | signal recognition particle 19 | -- |
| 1373829_at | Fgfr2 | Fibroblast growth factor receptor 2 | Signaling |
| 1367685_at | Rps27a /// Rps27a-ps12 /// Rps27a-ps28 | ribosomal protein S27a | Metabolism |
| 1376880_at | Tbk1 | Tank-binding kinase 1 | Signaling |
| 1367577_at | Hspb1 | Heat shock 27kda protein 1 | mRNA/Protein processing |
| 1375367_at | Pdlim2 | PDZ and LIM domain 2 | Transcription/Translation regulation |
| 1398256_at | Il1b | interleukin 1 beta | -- |
| 1388720_at | Rnf144b | ring finger protein 144B | Metabolism |
| 1372854_at | Ttc17 | tetratricopeptide repeat domain 17 | -- |
| 1376312_a_at | Desi2 | desumoylating isopeptidase 2 | -- |
| 1367711_at | Psmc2 | proteasome 26S subunit, ATPase 2 | Metabolism |
| 1367645_at | LOC100362366 /// LOC100365810 /// Rps17 | ibosomal protein S17 | mRNA/Protein processing |
| 1373859_at | Yipf6 | NOTFOUND | -- |
| 1372485_at | Pcbd1 | pterin-4 alpha-carbinolamine dehydratase 1 | Metabolism |
| 1388483_at | Cfl2 | cofilin 2 | -- |
| 1389064_at | Fem1c | fem-1 homolog C | -- |
| 1389046_at | Cnnm4 | cyclin and CBS domain divalent metal cation transport mediator 4 | -- |
| 1372613_at | Bdh2 | 3-hydroxybutyrate dehydrogenase, type 2 | Metabolism |
| 1369725_at | Adap2 | Arfgap with dual ph domains 2 | Signaling |
| 1387045_at | Atp6v0a1 | ATPase H+ transporting V0 subunit a1 | Metabolism |
| 1373135_at | Aarsd1 | alanyl-tRNA synthetase domain containing 1 | Transcription/Translation regulation |
| 1373605_at | Trak1 | trafficking kinesin protein 1 | Transport |
| 1367659_s_at | Eci1 | enoyl-CoA delta isomerase 1 | -- |
| 1371839_at | Srsf2 | serine and arginine rich splicing factor 2 | Metabolism |
| 1399077_at | Mtx1 | Metaxin 1 | -- |
| 1398309_at | Pigl | phosphatidylinositol glycan anchor biosynthesis, class L | -- |
| 1372468_at | Adgre5 | adhesion G protein-coupled receptor E5 | -- |
| 1375886_at | Ipp | intracisternal A particle-promoted polypeptide | -- |
| 1388820_at | Sltm | SAFB-like, transcription modulator | -- |
| 1398851_at | Ywhae | tyrosine 3-monooxygenase/tryptophan 5-monooxygenase activation protein, epsilon | Metabolism |
| 1372531_at | Ppfibp2 | PPFIA binding protein 2 | -- |
| 1375974_at | G3bp2 | G3BP stress granule assembly factor 2 | -- |
| 1368654_at | Npap60 | nucleoporin 50 | Transcription/Translation regulation |
| 1388882_at | Fkbp3 | FK506 binding protein 3 | Inflammation/Immune response |
| 1390104_at | Irgq | immunity-related GTPase Q | Metabolism |
| 1388641_at | Gart | phosphoribosylglycinamide formyltransferase | Metabolism |
| 1374842_at | Cebpz | CCAAT/enhancer binding protein zeta | -- |
| 1389038_at | Etf1 | eukaryotic translation termination factor 1 | -- |
| 1371622_at | Dph1 /// Ovca2 | diphthamide biosynthesis 1 | mRNA/Protein processing |
| 1377000_at | Whamm | WAS protein homolog associated with actin, golgi membranes and microtubules | Cell cycle/Apoptosis |
| 1376091_at | Adsl | adenylosuccinate lyase | Metabolism |
| 1375870_a_at | Rbms1 | RNA binding motif, single stranded interacting protein 1 | -- |
[truncated: 795,775 more chars]
