# Supplementary figures and images for: Daily variation of gene expression in diverse rat tissues
Source: PLoS One. 2018 May 10;13(5):e0197258. doi: 10.1371/journal.pone.0197258 (PMC5945012; doi:10.1371/journal.pone.0197258)

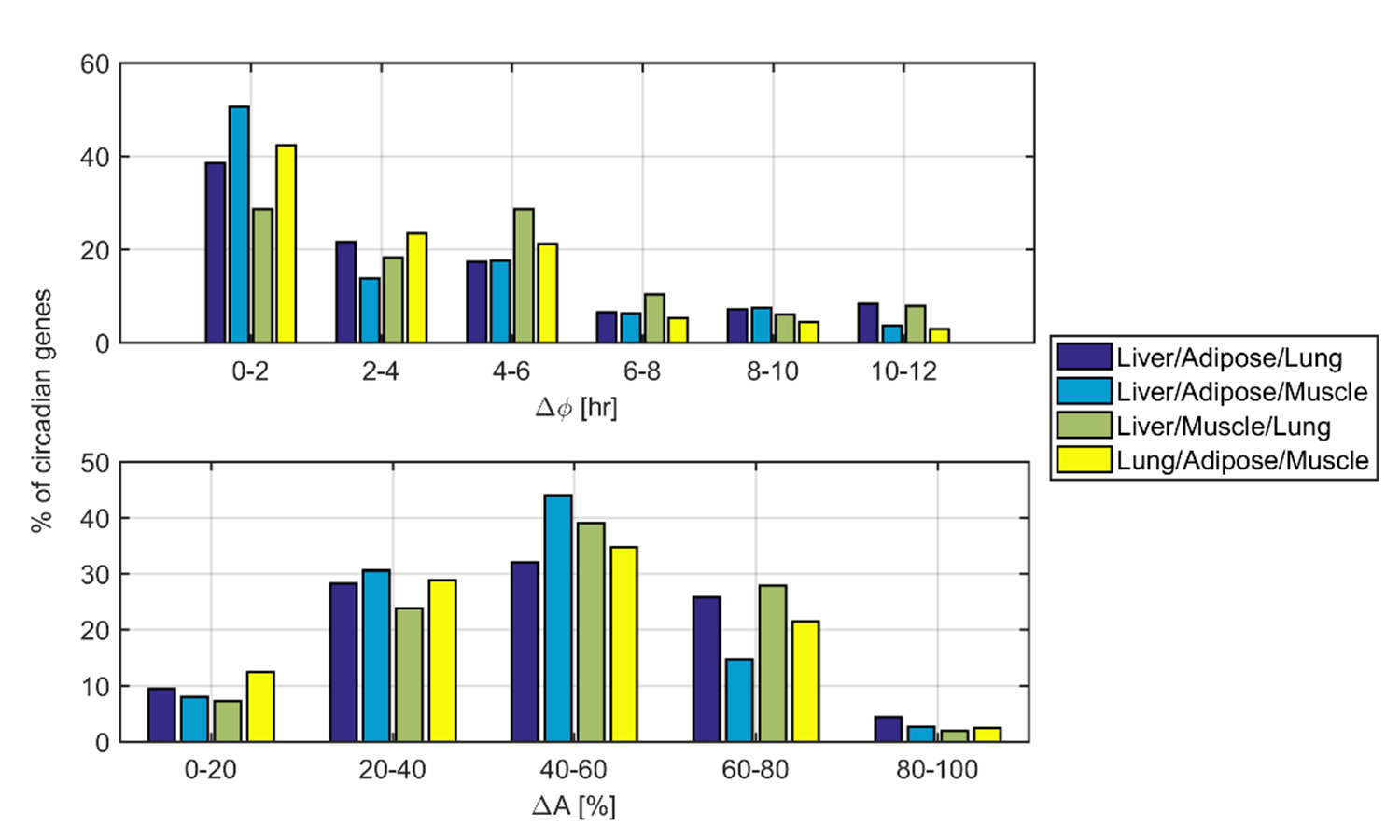

Supplement: S1 Fig — A: Histogram of phase lags of common genes in 3 tissues combinations. Different colors represent the various tissue combinations. Phase lags are separated to 6 groups on the x-axis, and represent genes that have a phase difference between 0 to 2, 2 to 4, 4 to 6, 6 to 8, 8 to 10, and 10 to 12 hours. The y-axis shows the percentage of circadian genes retaining a certain phase lag for combinations of certain tissues. B: Histogram of % amplitude difference of common genes in 3 tissues combinations. Different colors reflect the various tissue combinations. The % amplitude differences are separated in 5 groups on the x-axis, and represent genes that have a % amplitude differences of 0 to 20, 20 to 40, 40 to 60, 60 to 80, 80 to 100. The y-axis depicts the percentage of circadian genes retaining a certain amplitude difference for combinations of certain tissues. C: Scatterplot of phase lag (x-axis) versus % amplitude difference (y-axis). Different colors show different combination of 3 tissues. Lower panel boxplots indicate distributions of phase lags in the different combinations of tissues. Upper panel boxplots indicate distributions of % amplitude difference in the different combination of tissues. (TIF) [file pone.0197258.s002.tif]

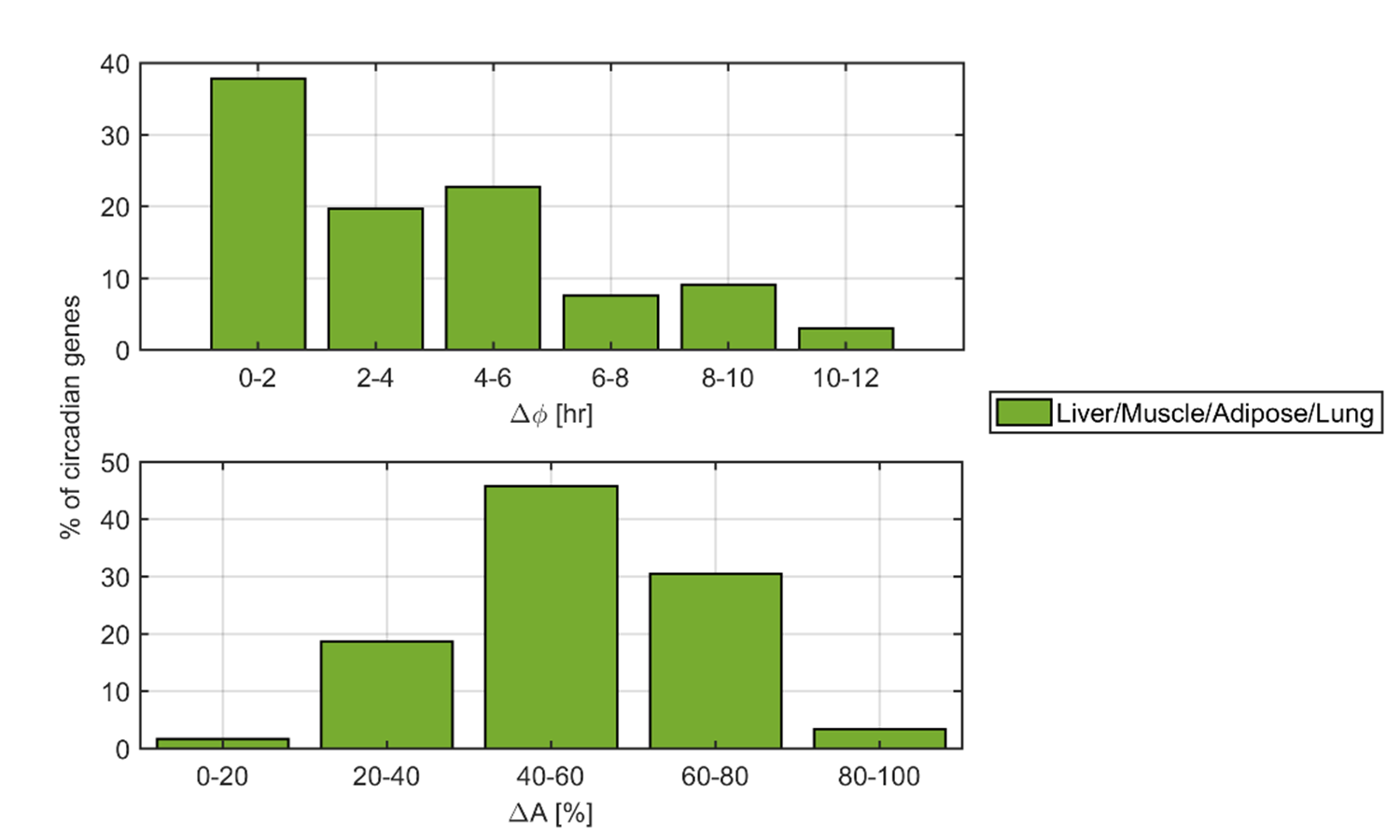

Supplement: S2 Fig — A: Histogram of phase lags of common genes in 4 tissues. Phase lags are separated to 6 groups on the x-axis, and represent genes that have a phase difference between 0 to 2, 2 to 4, 4 to 6, 6 to 8, 8 to 10, and 10 to 12 hours. The y-axis represents the percentage of circadian genes retaining a certain phase lag. B: Histogram of % amplitude difference of common genes in 4 tissues. % amplitude differences are separated in 5 groups on the x-axis, and represent genes that have a % amplitude different 0 to 20, 20 to 40, 40 to 60, 60 to 80, 80 to 100. The y-axis indicates the percentage of circadian genes retaining a certain amplitude difference. C: Scatterplot of phase lag (x-axis) versus % amplitude difference (y-axis). Lower panel boxplot shows the distribution of phase lags and upper panel boxplot the distribution of % amplitude. (TIF) [file pone.0197258.s003.tif]

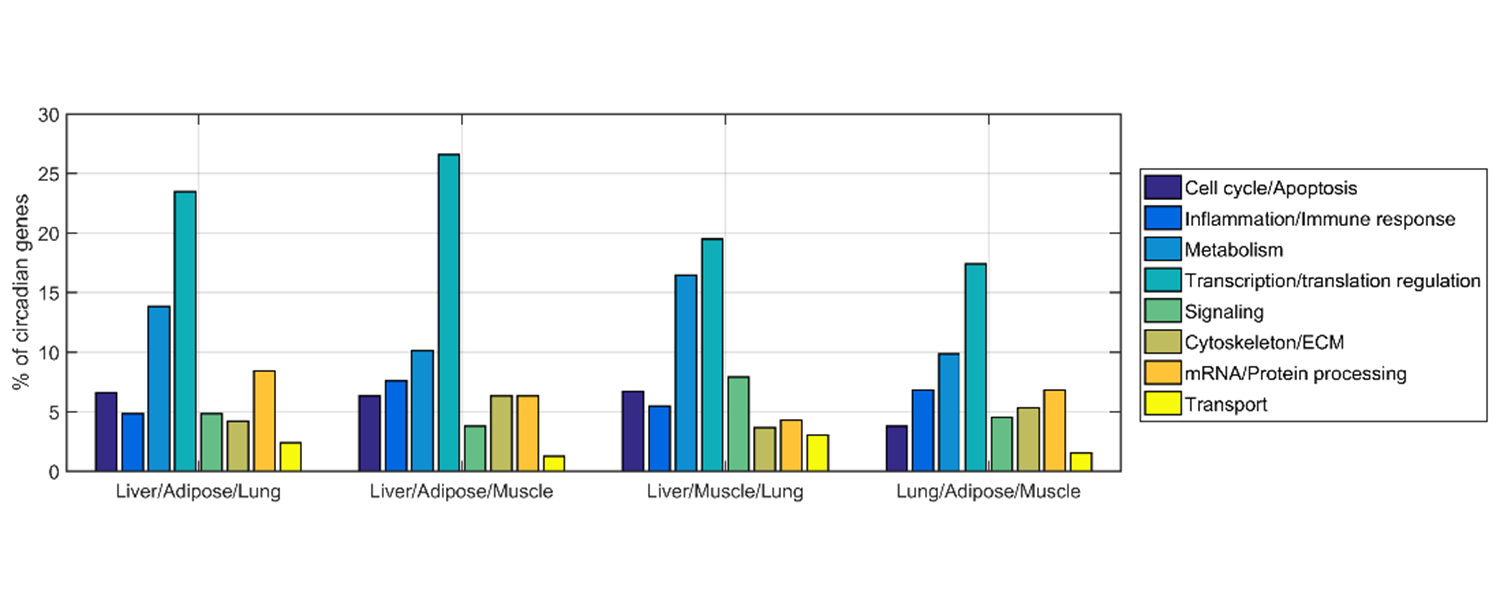

Supplement: S3 Fig — Different colors indicate the different functional groups. x-axis represents the different combination of tissues and the y-axis the percentage of circadian genes that belong to a certain functional group. (TIF) [file pone.0197258.s004.tif]

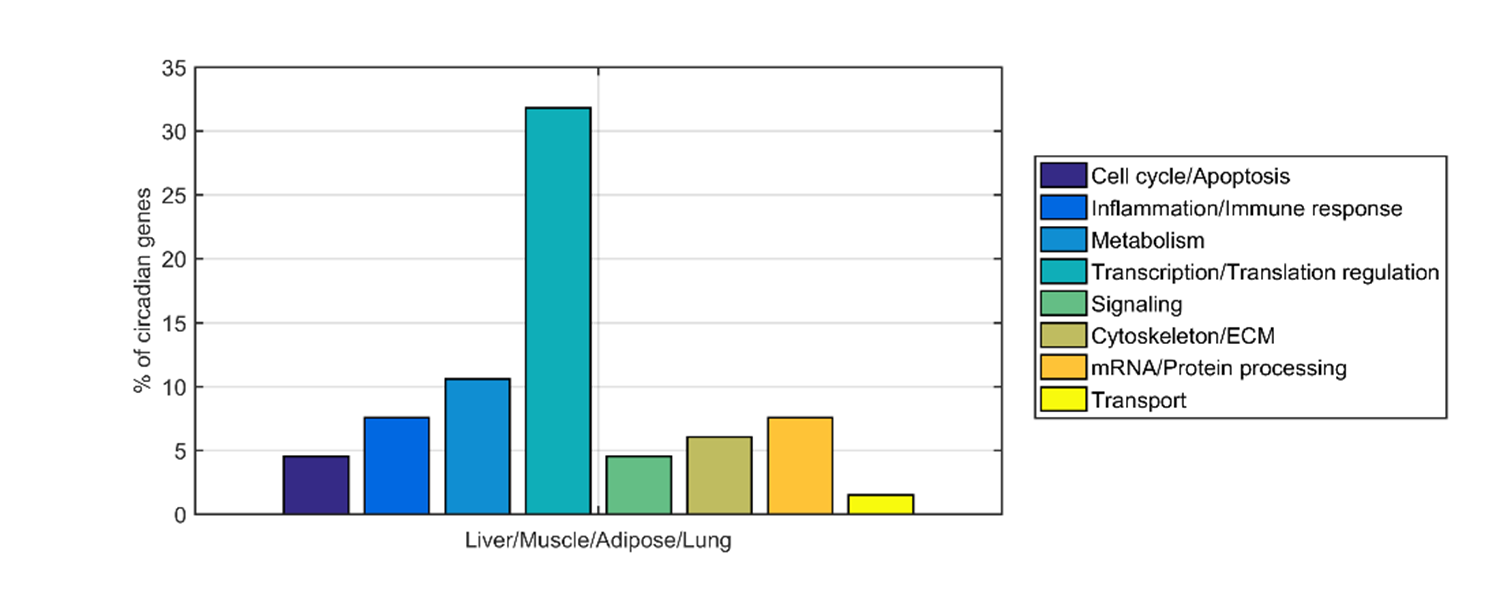

Supplement: S4 Fig — Different colors indicate the different functional groups. x-axis represents the different combination of tissues and the y-axis the percentage of circadian genes that belong to a certain functional group. (TIF) [file pone.0197258.s005.tif]
